# Reconfiguring interfacial hydrogen-bond networks to enhance anodic oxidation for efficient wastewater treatment

**DOI:** 10.1038/s41467-026-78208-2

**Published:** 2026-10-05

**Authors:** Tong Liu, Shuang Zhong, Xiaotong Liang, Hongrui Zhu, Ye Du, Bo Lai

**Affiliations:** 1 State Key Laboratory of Hydraulics and Mountain River Engineering, College of Architecture and Environment, Sichuan University, Chengdu, China; 2 Sino-German Centre for Water and Health Research, Sichuan University, Chengdu, China; 3 Key Laboratory of Green Chemistry and Technology of Ministry of Education, College of Chemistry, Sichuan University, Chengdu, China; 4 College of Materials Science and Engineering, Sichuan University, Chengdu, China

**Keywords:** Pollution remediation, Electrocatalysis, Pollution remediation

## Abstract

Anodic oxidation is a pivotally electrochemical technology for wastewater treatment containing emerging contaminants such as isothiazolinone antimicrobials, but sluggish interfacial water-splitting kinetics for hydroxyl radical (•OH) formation and a limited oxidation range, constrain its industrial scalability. This study leverages Fe^3+^ to strengthen interfacial water hydrogen bond, accelerating •OH formation and high-valent iron production, thus enhancing oxidation kinetics and scope. [Fe(OH)(H_2_O)_5_]^2+^ enhances the hydrogen-bond (H-bond) population at the electrode surface—particularly 4-HB-H_2_O motifs increase from 21% to 37% and weakens O–H bonds, due to its strong hydration and polarization ability. This reconfigured interfacial water H-bond structure markedly enhances water dissociation, leading to a 7-fold increase in the •OH concentration. Ultimately, Fe^3+^-mediated precise tuning of the H-bond network of water molecules enables non-selective degradation of diverse emerging contaminants, delivering stable long-term flow operation of 500 hours with ~95% removal efficiency maintained for contaminant. The system achieves a 44.3% cost reduction relative to conventional boron-doped diamond electrodes, establishing a convenient approach for industrial-scale electrochemical water treatment.

## Abstract (web summary)

This study reports Fe^3+^-mediated anodic oxidation strengthening interfacial water H-bonds to accelerate hydroxyl radical and high-valent iron production, expanding oxidation scope for contaminants and enabling stable low-cost operation.

## Introduction

**Fig. 1:**
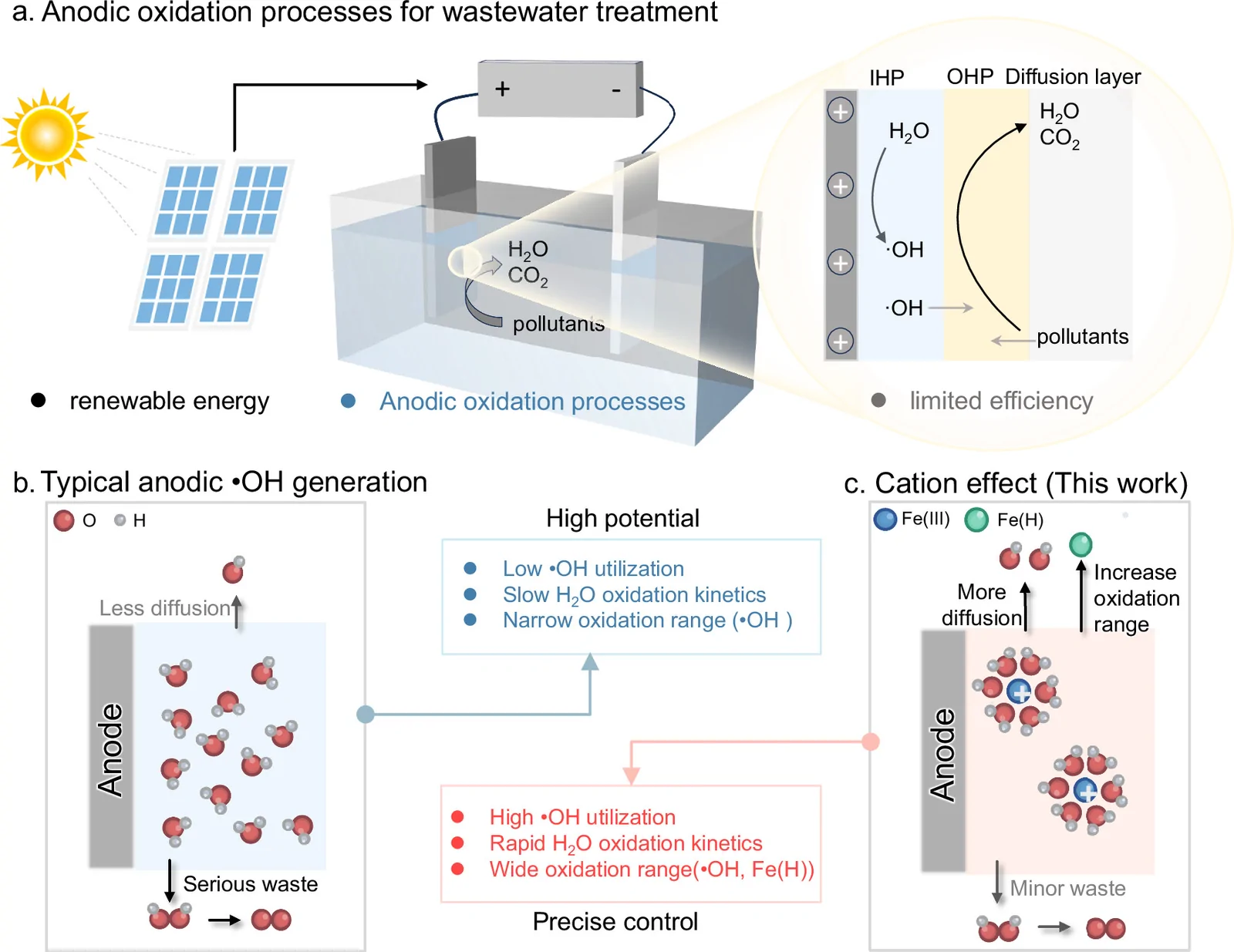
Schematic illustration of anode oxidation (AO) wastewater treatment technology. **a** The processes of AO wastewater treatment technology. (IHP: Inner Helmholtz Plane, OHP: Outer Helmholtz Plane). **b** A summary of the limitations of the traditional AO system. **c** Fe^3+^ coupled AO system for efficient and sustainable wastewater treatment. AO, an electrochemical advanced oxidation process that employs high-oxygen-overpotential anodes to generate reactive species for the degradation and mineralization of organic pollutants.

Anodic oxidation (AO) process has shown great potential in the field of wastewater treatment^1–4^. The core of this technology lies in its ability to directly activate water molecules at the anode interface, generating highly reactive oxidative species such as hydroxyl radicals (•OH)^3–6^. The rapid development of renewable energy sources enables electrocatalytic anodic oxidation to emerge as a green and sustainable technology for water treatment (Fig. 1a). However, the key bottleneck for the realization of this technology in large-scale industrial applications lies in the limitations of its oxidation capacity and scope^7^. The electrochemical decomposition of water molecules at the anode surface exhibits a slow kinetics^7–9^, and •OH dimerization occurs at the anode surface to produce hydrogen peroxide (H_2_O_2_)^10,11^. Furthermore, the lifetime of the •OH generated is extremely short (~1 ns)^6^, making it difficult for them to react sufficiently with the pollutants in the bulk phase^12,13^, resulting in low utilization efficiency (Fig. 1b). These result in low efficiency of pollutant degradation during the AO water treatment process and high energy consumption, making it difficult to optimize the balance between treatment efficiency and energy consumption, thus becoming a deep challenge for its large-scale promotion. Hence, there is an urgent need to practical solutions to address these challenges.

To address the issue of low oxidation capacity, traditional methods employ material design to alter the pathway of water decomposition (from the four-electron transfer oxygen evolution reaction (OER) to the single-electron water oxidation)^4,14–16^. For example, Ni single-atom-driven “H-pulling effect” coupled with the electronic metal support interactions (EMSI) effect promotes the selective generation of •OH^3^. These methods can indeed increase the production of •OH to a certain extent. However, these methods are essentially limited by the classical *Sabatier* principle and the scaling relationship between reaction intermediates, resulting in an unsatisfactory •OH yield^17,18^. Moreover, the dimer quenching that occurs on the electrode surface shortly after the generation of •OH further restricts its diffusion and utilization into the solution phase, constituting another bottleneck that is difficult to be solved by material design alone. Regarding the issue of limited oxidation range, some have suggested that using UV-assisted anode H_2_O oxidation can expand the diffusion range of •OH^19^. However, it is limited by the light transmittance of wastewater, and it can also cause adverse side reactions, resulting in low controllability and difficulty in large-scale application. Therefore, we urgently need a paradigm shift in research, shifting the focus from the intrinsic properties of the electrode to the active design and manipulation of the microenvironment of the electrode-electrolyte interface, which is the key reaction region^20^.

Compared to material regulation and coupling processes, electrolyte engineering is easier to implement and offers greater controllability, yet it is often overlooked by researchers^21^. By precisely adjusting the composition of the electrolyte to shape the microenvironment at the electrode interface (including the electric double layer (EDL), electric field and interface water structure, etc.), it is possible to directly control the pathways, rates and selectivity of electrochemical reactions^20,22^. The water molecules that serve as key reactants during the anodization process have been newly evidenced to play a crucial role^22,23^. The latest research indicates that the structure and dynamics of the interface water are jointly governed by the electric field and ionic hydration, and can profoundly affect the activation energy barrier of water oxidation^22,24–27^. The metal cations can act on the Outer Helmholtz Plane (OHP) at the anode and form strong non-covalent interactions with the chemically adsorbed intermediates, which affect the adsorption energy and kinetics of the anodic oxidation intermediates^21,25,28^. Furthermore, the strong cationic hydration effect can reconstruct the hydrogen bond (H-bond) network to modify the microenvironment of water molecules, thereby altering the activity of anode water oxidation^21,29–31^. However, how to conduct microscopic regulation of the water environment at the anode solid-liquid interface, and increase the production and utilization rate of •OH, remains a scientific challenge that has not yet been resolved.

Herein, we achieved a double benefit through the Fe^3+^-mediated anodic oxidation system. The aim is to achieve efficient electro-production of •OH through precise engineering of the interface H_2_O H-bond networks (Fig. 1c). The Fe^3+^ restructured the H-bond network of water molecules on the electrode surface, accelerating the kinetics of the generation of •OH. At the same time, the Fe^3+^ is oxidized to high-valent iron (Fe(H), including Fe(IV), Fe(V), and Fe(VI)), thereby expanding the oxidation range of the system. *Operando* electrochemical characterizations indicate that Fe^3+^ promotes interfacial charge transfer and the kinetics of water oxidation. In situ electrochemical spectroscopy and ab initio molecular dynamics (AIMD) demonstrate that [Fe(OH)(H_2_O)_5_]^2+^ enhances the 4-HB-H_2_O and H-bond population at the electrode surface^32^, thereby guiding the reaction path towards a lower energy barrier^24,33,34^. Meanwhile, the Fe^3+^ is oxidized to Fe(H), thereby expanding the oxidation range of the indirect oxidation at the anode. The probe experiment and the COMSOL simulation reveal that Fe^3+^ increases the diffusion range of •OH at the interface and provide a more extensive diffusion range of Fe(H). This study establishes a convenient approach for regulating the H-bond network of water molecules to enhance the kinetics of •OH generation. It both provides guidance for efficient and energy-saving water treatment technologies and has broad applicability to other electrocatalytic processes involving water oxidation or the generation of reactive radicals.

## Results

### Pollutant degradation and identification of dominant reactive species in the Fe^3+^/BDD system

**Fig. 2:**
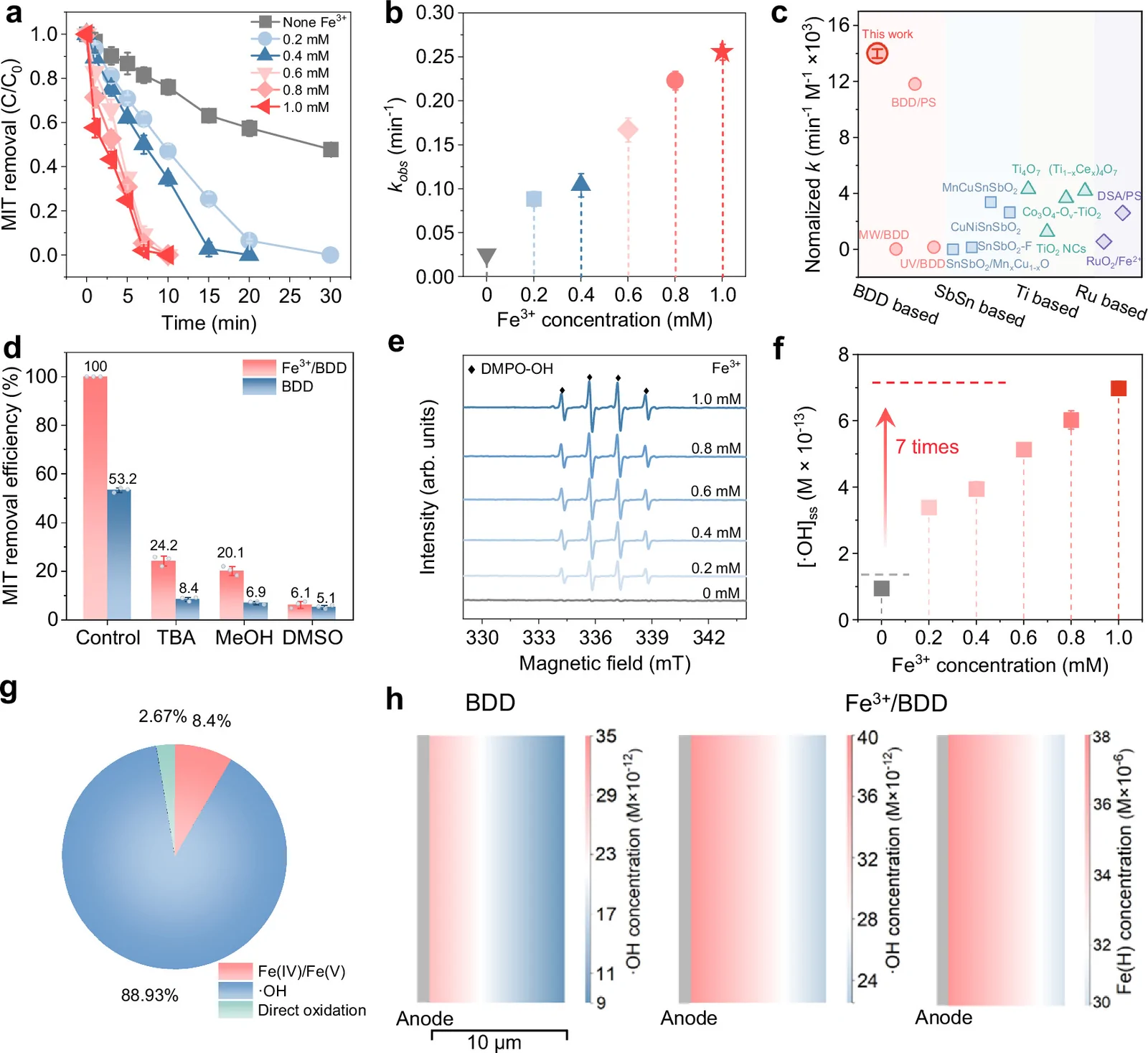
Pollutant degradation and identification of dominant reactive species in the Fe^3+^/boron-doped diamond (BDD) system. **a**, **b** Effect of Fe^3+^ concentration on the removal efficiency of methyl isothiazolinone (MIT) and the observed rate constant (*k*_obs_) in Fe^3+^/BDD system. **c** Comparison of the pollutant normalization degradation kinetics in state-of-the-art anodic oxidation studies. Detailed data are given in Supplementary Table 3. **d** Effect of scavenger (TBA, MeOH and DMSO) on the removal of MIT in the BDD and Fe^3+^/BDD systems. **e** Effect of Fe^3+^ concentration on the DMPO-OH signal intensity in the EPR test. **f** Effect of Fe^3+^ concentration on the generated concentration of •OH in Fe^3+^/BDD. **g** Relative contributions of reactive species to MIT removal in the Fe^3+^/BDD system. **h** Concentration distribution of •OH and Fe(IV)/Fe(V) at the anode interface were simulated using COMSOL in BDD and Fe^3+^/BDD systems. Error bars represent the standard deviation, obtained by repeating the experiment thrice (*n* = 3). Experimental conditions: current density = 7.5 mA cm^−2^, [Na_2_SO_4_]  = 50 mM, [MIT] = 20 µM, [DMPO] = 20 µM, [TBA] = [MeOH] =  500 mM, [DMSO] = 50 mM, [Fe^3+^] = 0.2 mM. Source data are provided as a Source Data file.

To evaluate the impact of Fe^3+^ introduction on anodic oxidation for pollutant degradation, a commercial boron-doped diamond (BDD) electrode was employed, with methyl isothiazolinone (MIT) selected as a representative emerging organic pollutant. As shown in Fig. 2a, the pristine BDD electrode achieved only 53.2% removal of MIT within 30 min. Notably, the addition of only 0.2 mM Fe^3+^ achieved complete MIT removal and increased the observed rate constant (*k*_obs_) by 3.5 times (Fig. 2b). With increasing Fe^3+^ concentration, the degradation efficiency further improved, with *k*_obs_ increasing from 0.089 to 0.26 and reaching a maximum at 1 mM. The 0.2 mM Fe^3+^ was selected over 1 mM to enhance degradation kinetics while reducing chemical use and iron sludge—avoiding reduced energy efficiency and secondary pollution at higher concentrations. These results demonstrate that the introduction of Fe^3+^ can substantially enhance the pollutant degradation efficiency of BDD anodic oxidation.

The effect of current density was also systematically investigated, showing enhanced removal efficiency with increasing current density. However, the performance plateaued when the current density exceeded 7.5 mA cm^−2^ (Supplementary Fig. 1). Considering the trade-off between operational cost, energy consumption, post-treatment and degradation efficiency, 0.2 mM Fe^3+^ and 7.5 mA cm^−2^ were selected as the optimal conditions for subsequent experiments. At this parameter, the Fe^3+^/BDD system maintained complete removal of MIT for 15 cycles, demonstrating excellent stability (Supplementary Fig. 2). As shown in Fig. 2c and Supplementary Table 3, the Fe^3+^/BDD system outperforms other representative anodic oxidation materials and combined processes in pollutant degradation.

A combination of quenching, probe and electron paramagnetic resonance (EPR) experiments was employed to identify the dominant reactive species in the Fe^3+^/BDD system. As shown in Fig. 2d, Supplementary Figs. 3 and 4, in the Fe^3+^/BDD system, the addition of tert-butanol (TBA) and methanol (MeOH) significantly quenched the degradation efficiency from 100% to 24.2% and 20.1%, respectively, indicating •OH was the main reactive species. After quenching with MeOH, the MIT removal rate in the Fe^3+^/BDD system decreased by 75.8%, whereas that of BDD alone decreased by only 44.8%. This 31% difference was attributed to the enhanced generation of •OH resulting from the introduction of Fe^3+^. Comparisons with a sodium perchlorate electrolyte ruled out the contribution of sulfate radicals (SO_4_^•−^) to pollutant removal (Supplementary Fig. 5). Nitrobenzene (NB), a common probe for •OH^3^, showed progressively enhanced degradation with increasing Fe^3+^ concentration (Supplementary Fig. 6). Through kinetic calculations, it was shown that the generated •OH concentration could be increased by 7-fold (Fig. 2f and Supplementary Text 7). Consistently, EPR spectra (Fig. 2e) revealed a gradual increase in the DMPO–OH adduct signal as the Fe^3+^ concentration increased. The DMPO–OH signal was effectively quenched by •OH scavengers or excess TBA (Supplementary Fig. 7)^35^, confirming •OH as the dominant reactive species responsible for pollutant degradation.

**Fig. 3:**
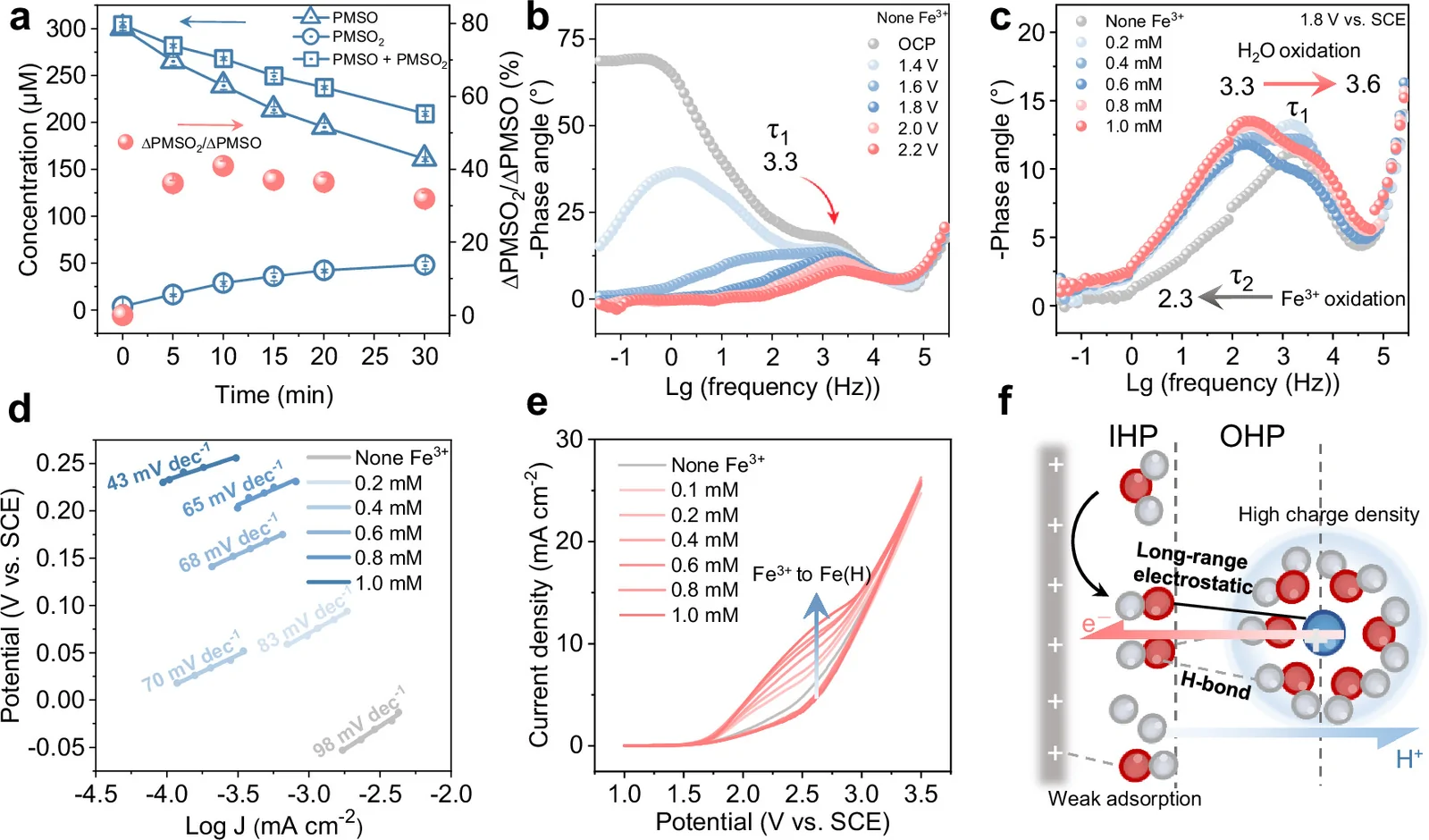
*Operando* electrochemical characterization to monitor the effect of Fe^3+^ on the anodic oxidation interface. **a** Changes in PMSO consumption, PMSO_2_ generation, and conversion rate over time. Error bars represent the standard deviation, obtained by repeating the experiment thrice (*n* = 3). **b** Bode phase angle plots at different potentials without Fe^3+^. **c** Bode phase angle plots at different Fe^3+^ concentrations at 1.8 V vs. SCE (saturated calomel electrode). *τ*_1_ and *τ*_2_ represent the time constants of two different relaxation processes. **d** Tafel slopes at different Fe^3+^ concentrations. **e** Cyclic voltammogram (CV) curves at different Fe^3+^ concentrations. Blue arrow: the oxidation peak of Fe^3+^ oxidized to Fe(H) increases with rising Fe^3+^ concentration. **f** Schematic diagram of the mechanism of the Fe^3+^-regulated electric double layer (EDL) at the BDD anode (IHP: Inner Helmholtz Plane, OHP: Outer Helmholtz Plane). Source data are provided as a Source Data file. The red spheres represent oxygen atoms, the gray spheres represent hydrogen atoms, and the blue spheres represent Fe^3+^.

DMSO, a representative quencher for both •OH and Fe(H)^36^, exhibited a slightly stronger inhibitory effect on pollutant degradation than MeOH and TBA in the Fe^3+^/BDD system, indicating the involvement of Fe(H). The conversion rate of PMSO and the yield of product PMSO_2_ both increased linearly with the Fe^3+^ concentration (Fig. 3a, Supplementary Figs. 8 and 9), providing compelling evidence for the formation of Fe(IV)/Fe(V)^36^. In situ electrochemical Raman spectroscopy showed that the characteristic signals of Fe(VI) increased with anodic potential, confirming Fe(VI) as the electrochemically generated species during the reaction (Supplementary Fig. 10). However, the reaction rates of Fe(IV)/Fe(V) with pollutants were 2–6 orders of magnitude higher than those of Fe(VI), making them the dominant active high-valent iron species^37^. Moreover, no ozone was detected in the BDD anode oxidation system under our experimental conditions (Supplementary Fig. 11). Collectively, •OH and Fe(IV)/Fe(V) are identified as the dominant reactive species in the Fe^3+^/BDD system.

Quenching analysis and kinetic calculations (Fig. 2g, Supplementary Fig. 12, and Supplementary Text 6) indicated that •OH and Fe(IV)/Fe(V) together dominated pollutant degradation, contributing 88.9% and 8.4%, respectively, whereas direct electron transfer played a negligible role (2.7%). According to literature and experimental results, the second-order reaction rate constant of •OH with MIT (*k*_•OH, MIT_ = 2.09 × 10^9^ M^−1^ s^−1^) was significantly higher than that of Fe(V)/Fe(IV) (*k*_Fe(V)/Fe(IV), MIT_ = 1.08 × 10^3^ M^−1^ s^−1^) (Supplementary Text 8 and Supplementary Fig. 13)^38^, but the steady-state concentration of •OH (3.39 × 10^−13^ M) was lower than that of Fe(V)/Fe(IV) (1.20 × 10^−7^ M) (Supplementary Text 7). Therefore, the *k*_obs, MIT_ of •OH was 5.5 times that of Fe(V)/Fe(IV) (Supplementary Text 9 and Supplementary Fig. 13), consistent with their relative contributions to MIT removal.

However, another inherent limitation of anodic oxidation lies in the short lifetime of •OH (~1 ns)^6^, which restricts the effective oxidation range. The coexistence of increased •OH generation and the formation of longer-lived Fe(H) (e.g. half-life time of Fe(IV) is 7 s at pH 3^39^) was expected to synergistically expand the oxidation range. COMSOL simulations were therefore employed to visualize the mass-transfer behavior of reactive species and to provide an intuitive comparison of the spatial distribution of oxidation zones. Finite element simulation were conducted to model the interfacial concentration distribution of •OH and Fe(H) at the micrometer scale^12^. COMSOL simulation visualized the mass transfer of active species at the anode interface (Fig. 2h and Supplementary Text 10)^40^. Within the simulation domain, •OH concentration decayed rapidly, with a ~75% decrease on the pristine BDD anode but only ~45% on the Fe^3+^/BDD anode. In contrast, Fe(H) exhibited much slower decay, decreasing by only ~20% on the Fe^3+^/BDD anode.

In conclusion, the introduction of Fe^3+^ substantially enhances the oxidative capacity of the Fe^3+^/BDD system by simultaneously increasing •OH generation yield and inducing the formation of Fe(H) species. The coexistence of abundant •OH and longer-lived Fe(H) species significantly extends the diffusion and effective reaction range of reactive oxidants, thereby expanding the overall oxidation scope. Consequently, the Fe^3+^-mediated BDD anodic oxidation system enables rapid, efficient, and stable pollutant degradation.

### Charge and mass transfer in Fe^3+^-mediated anodic oxidation processes

To further elucidate the interfacial charge and mass transfer changes induced by Fe^3+^ introduction in the BDD anodic oxidation system, *operando* electrochemical characterizations were employed to probe the electrode surface and interface, providing mechanistic insights into the origin of the altered reactive species^41^.

In the absence of Fe^3+^, the Bode phase plots (Fig. 3b and Supplementary Fig. 14) showed a dominant time constant (*τ*_1_) at lg 3.3, corresponding to the charge transfer during H_2_O oxidation to •OH on the BDD anode. As the Fe^3+^ concentration increased from 0 to 1 mM, the high-frequency characteristic peak shifted from lg 3.3 to lg 3.6 (Fig. 3c and Supplementary Fig. 15), indicating a shorter relaxation time and accelerated interfacial charge-transfer kinetics. This shift confirmed that Fe^3+^ significantly expedited the kinetics of interfacial water oxidation on the BDD anode. A new relaxation process (*τ*_2_) appeared at lg 2.3 and became more pronounced with increasing Fe^3+^ concentration, reflecting an additional Fe^3+^-related interfacial reaction. The coexistence of *τ*_1_ (H_2_O oxidation) and *τ*_2_ (Fe^3+^ oxidation) strongly proved that Fe^3+^ introduced a parallel and efficient Faraday reaction pathway at the interface. When the 1 mM Fe^3+^ system was subjected to potential scanning (Supplementary Fig. 16a), the high-frequency peak remained stable at lg 3.6, indicating that the reaction path of •OH was stable throughout the potential window.

Tafel analysis provided direct insight into changes in electrochemical reaction kinetics. As shown in Supplementary Fig. 18, increasing Fe^3+^ concentration led to a rightward shift of the Tafel curves, accompanied by a pronounced decrease in the Tafel slope value from 98 to 43 mV dec^−1^ (Fig. 3d), indicating the introduction of Fe^3+^ significantly enhanced interfacial charge-transfer efficiency, facilitating both H_2_O oxidation and Fe^3+^ oxidation at the electrode surface. These results are fully consistent with the accelerated interfacial charge-transfer behavior revealed by EIS measurements.

To directly resolve potential-dependent reaction changes and pathway, cyclic voltammetry (CV) tests were performed^36,42^. As shown in Fig. 3e, in the potential range of 1.8 to 2.8 V vs. SCE, a distinct oxidation peak linearly increased with the increase in Fe^3+^ concentration. This peak corresponds to the new relaxation process (*τ*_2_) as indicated by the Bode phase angle diagram, precisely corresponding to the electrochemical oxidation process of Fe^3+^ to Fe(H). The CV at low potentials showed a linear dependence of the Fe^2+^/Fe^3+^ redox peak current on the square root of the scan rate (Supplementary Fig. 19), indicating diffusion-controlled of Fe^3+^ at the BDD surface and no penetration into the IHP. At pH 3, the main form of Fe^3+^ was (Fe(OH))^2+^ (Supplementary Fig. 20), it acting on OHP, enhanced the reaction rate of interface water oxidation^21^, thereby strengthening the generation of •OH, and simultaneously rapidly oxidizing to form Fe(H).

The in situ spectroscopy and *operando* electrochemical characterization results mentioned above (Fig. 3f) indicated that in the anodic oxidation system, the introduction of (Fe(OH))^2+^ can significantly enhance the charge transfer efficiency of H_2_O, thereby accelerating the kinetics of •OH generation. At the same time, (Fe(OH))^2+^ acted on the OHP of the BDD anode, promoting the desorption of •OH through long-range electrostatic interactions and oxidizing it to generate Fe(H), thereby increasing the oxidation range. This interface reaction behavior was highly consistent with the aforementioned pollutant degradation performance and the identification results of the main reactive species. However, in order to reveal the reaction mechanism at a more microscopic level, further exploration is still needed: how does the introduction of Fe^3+^ change the structural state and intrinsic reactivity of the interface water molecules, thereby enhancing the efficiency of charge transfer of water molecules? After the generation of •OH and Fe(H), can these reactive species be efficiently utilized and continuously participate in subsequent oxidation reactions?

### Iron-induced reconfiguration of the hydrogen-bond network at the anodic surface

**Fig. 4:**
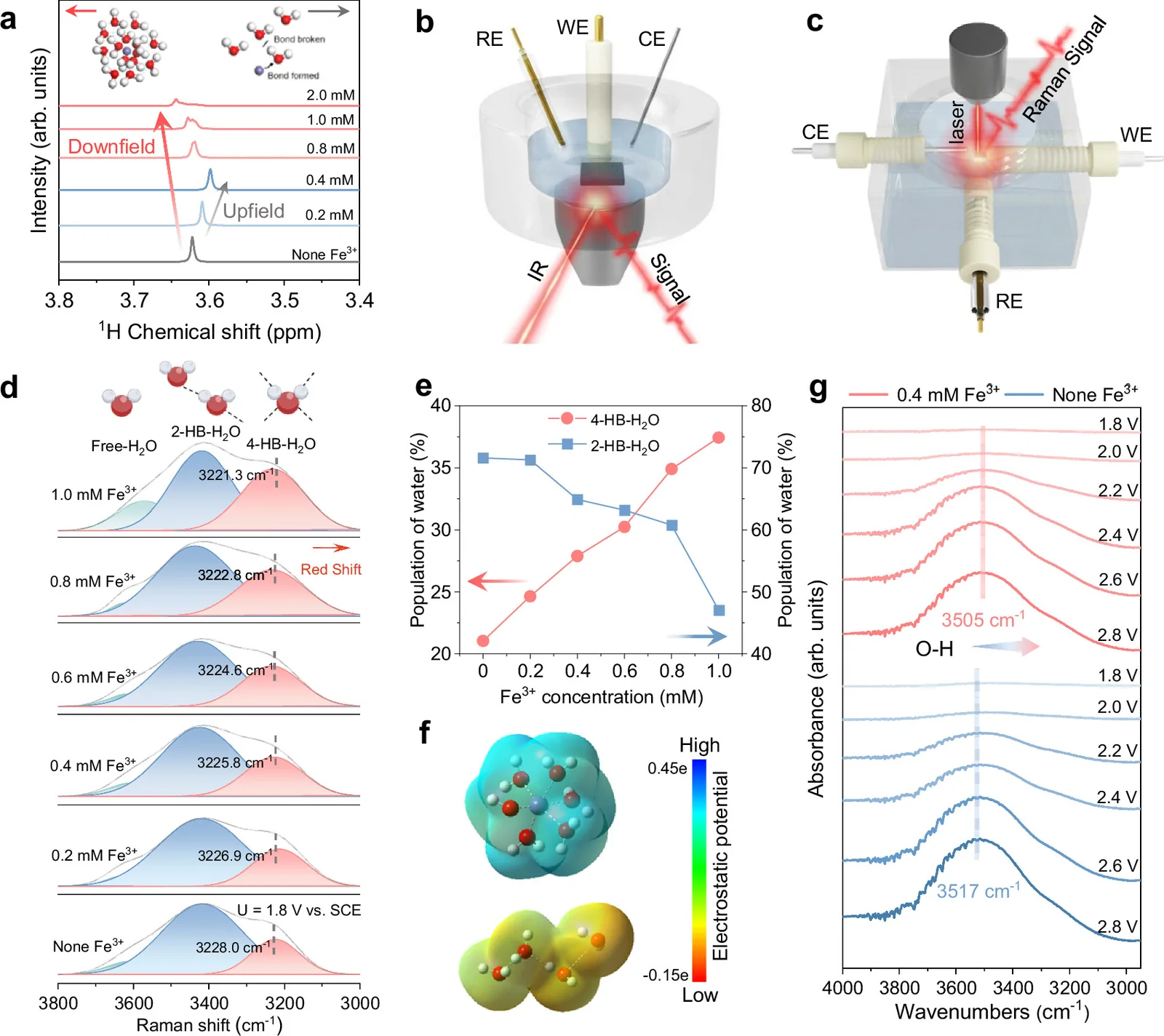
In situ spectroscopic analysis of hydrogen-bond network in the Fe^3+^/BDD system. **a** ^1^H NMR chemical shifts for water at different Fe^3+^ concentration. Red arrow indicates a shifted downfield, grey arrow indicates a shifted upfield. **b**, **c** Schematic illustrations of the principles of in situ electrochemical Raman and Fourier transform infrared (FTIR) spectroscopy. (WE: working electrode, RE: reference electrode, CE: counter electrode). **d** In situ Raman spectra at 1.8 V vs. SCE with Fe^3+^ concentrations ranging from 0 to 1 mM. (4-HB-H_2_O: ~3200 cm^−1^, 2-HB-H_2_O: ~3400 cm^−1^ free H_2_O: ~3600 cm^−1^). **e** Concentration-dependent population changes of 4-HB-H_2_O (two H-bond donors, two acceptors) and 2-HB-H_2_O (one H-bond donor, one acceptor). **f** Electrostatic potential distribution of water molecules and [Fe(OH)(H_2_O)_5_]^2+^ hydrated clusters. **g** In situ FTIR O–H stretching bands on the BDD surface at high potentials (1.8–2.8 V vs. SCE) with and without Fe^3+^. The red spheres represent oxygen atoms, the gray spheres represent hydrogen atoms, and the purple spheres represent Fe^3+^. Source data are provided as a Source Data file.

At pH 3, Fe^3+^ mainly exists in the solvated structure of [Fe(OH)(H_2_O)_5_]^2+^ (Supplementary Fig. 20)^32^. To clarify the positive effect of Fe^3+^ on the reconfiguration of the H-bond network of water molecules, we conducted nuclear magnetic resonance hydrogen spectrum (^1^H NMR) tests^31,43,44^. The results showed an interesting non-monotonic change (Fig. 4a). First, low concentrations of Fe^3+^ forcibly disrupted water molecules to form the local hydration shells (0.2–0.4 mM). Although local hydrogen bonds were formed, this solvation process disrupted the original bulk water H-bond network. The strong local H-bond network formed by the Fe^3+^ hydrated sphere ([Fe(OH)(H_2_O)_5_]^2+^) was masked by paramagnetic effects, causing the overall NMR signal to appear as weakly H-bond water shifted toward upfield. As concentration increases (0.4–2 mM), more Fe^3+^ hydrated spheres formed, and the strong local polarization induced by Fe^3+^ within these spheres propagates through chemical bonds to the surface coordinated water molecules, weakening their O–H bonds. This allowed the coordinated water molecules to form tighter H-bond network with outer water molecules, shifting the NMR signal toward downfield. Additionally, due to the paramagnetism of Fe^3+^, water exchange within the hydrated spheres became slow, and multiple Fe^3+^ paramagnetic centers accumulated under the strong H-bond network. According to nuclear magnetic relaxation theory, this led to a shortened transverse relaxation time, resulting in broadening of the NMR peak shapes.

Additionally, 2D DOSY NMR also indicated the rigidity of this strong H-bond network (Supplementary Fig. 21). After adding Fe^3+^, the diffusion coefficient decreased, indicating slower water exchange and the formation of a more tightly bound H-bond network^45^. This phenomenon confirmed the process of the H-bond network of water molecules reconfigured by Fe^3+^. In the Fe^3+^-introduced BDD system, water remained the primary reactant in anodic oxidation, while variations in interfacial polarization can markedly alter the structure and polarity of interfacial water^21,30,46^. Owing to its strong hydration and polarization capabilities (Fig. 4f), Fe^3+^ can interact with interfacial water to form ordered hydration structures, thereby modulating the physicochemical properties of water and potentially influencing the kinetics of anodic H_2_O oxidation toward •OH generation. However, the ^1^H NMR spectrum was an exogenous characterization and cannot represent the conditions at the electrode interface and the working potential. Therefore, to elucidate this effect at the molecular level, interfacial water microenvironmental changes were probed by electrochemical in situ Raman and FTIR spectroscopy (Fig. 4b, c, Supplementary Figs. 22 and 23).

H_2_O can be generally classified into three typically structural states: 4-HB-H_2_O (two H-bond donors and two acceptors), 2-HB-H_2_O (one H-bond donor and one acceptor), and free H_2_O. These distinct water configurations can be identified by electrochemical in situ Raman spectroscopy^47^. From Fig. 4d, with increasing Fe^3+^ concentration, the characteristic band at ~3200 cm^−1^ assigned to 4-HB-H_2_O exhibited both enhanced intensity and a pronounced red shift (from 3228.0 cm^−1^ at 0 mM to 3221.3 cm^−1^ at 1.0 mM)^48,49^, indicating strengthened hydrogen bonding. In general, a redshift of the O–H stretching band is associated with a longer and weaker O–H bond, which often results from stronger hydrogen bonding interactions^50^. Meanwhile, the increased intensity of this band suggested a higher population of the 4-HB-H_2_O species. Therefore, the observed spectral changes were consistent with a strengthening of the interfacial H-bond network and a weakening of the O–H covalent bonds induced by the presence of Fe^3+^ species. This weakening and polarization of O–H bonds may facilitate subsequent bond cleavage, i.e., a pre-activated state of water.

As shown in Fig. 4e, the quantitative analysis of the spectral graph using Gaussian fitting revealed that the relative proportion of 4-HB-H_2_O increased sharply from 21 to 37%, while the proportion of 2-HB-H_2_O corresponding to the weak H-bond interaction decreased from 72 to 47%. The quantitative analysis of hydrogen bonding further confirmed that [Fe(OH)(H_2_O)_5_]^2+^ strengthened the interfacial H-bond network. A linear relationship (*R*^2^ > 0.96) between the content of 4-HB-H_2_O and the concentration of •OH was established through quantitative analysis (Supplementary Fig. 24), thereby clarifying the connection between the structural changes of hydrogen bonds and the reaction kinetics.

[Fe(OH)(H_2_O)_5_]^2+^ exhibited a high charge density and a unique OH⁻/H_2_O ligand arrangement on its surface and extending outward through H-bond interactions to form a hydration cluster (Fig. 4f)^51^. The strong electron-withdrawing ability of Fe^3+^ caused the electron cloud of the O–H bond of water molecules to be greatly pulled towards the O atom, severely weakening the O–H bond, thereby causing the Raman vibration frequency to red shift (Fig. 4d). Its strong electrostatic field can extend to the second, third, and even further hydration layers, inducing the relatively disordered water molecules into a highly structured 4-HB-H_2_O configuration. The 4-HB-H_2_O structure of water molecules provided a rapid proton transfer pathway at the interface, facilitating charge transfer at the interface.

In situ FTIR spectroscopy further provided direct evidence for the O–H changes of water molecules^52–54^. As shown in Fig. 4g, upon applying an anodic potential from 1.8 to 2.8 V vs. SCE, the BDD system without Fe^3+^ exhibited a gradual increase in the intensity of the O–H stretching band at ~3517 cm^−1^, while the peak position remained unchanged. This reflected that under a strong electric field, the orientation of polar water molecules was significantly altered (i.e., the negative oxygen end facing the electrode and the positive hydrogen end facing the solution), while the O–H bond energy remained unchanged. In the in situ FTIR spectrum, the signal intensity was associated with both the number of vibrating bonds and their orientation. As the potential increased, a larger fraction of water molecules adopted infrared-active orientations, resulting in a systematic enhancement of the O–H stretching signal intensity. However, in the presence of Fe^3+^, the O–H peak position of water molecules shifted to 3505 cm^−1^, and this shift was independent of the potential^55^.

In the [Fe(OH)(H_2_O)_5_]^2+^ induced water network, the electron cloud of the O–H bond was strongly polarized by the H-bond interactions with both acceptors (the O atom of the adjacent water molecule or the OH⁻) and the H-bond donors (H···O–H···O). As a result, the electron cloud of the O–H bond was displaced away from the H atom toward the O atom and the H-bond acceptor, leading to a reduced electron density within the O–H bond and a consequent decrease in bond energy^54^. Combined with the above ^1^H NMR and in situ Raman spectra, the H-bond network reconfiguration triggered by [Fe(OH)(H_2_O)_5_]^2+^ increased the number of 4-HB-H_2_O and hydrogen bonds, weakened O–H, and created an extremely favorable state for the anodic water oxidation reaction.

### AIMD simulation of the interface water structure and anodic oxidation mechanisms

**Fig. 5:**
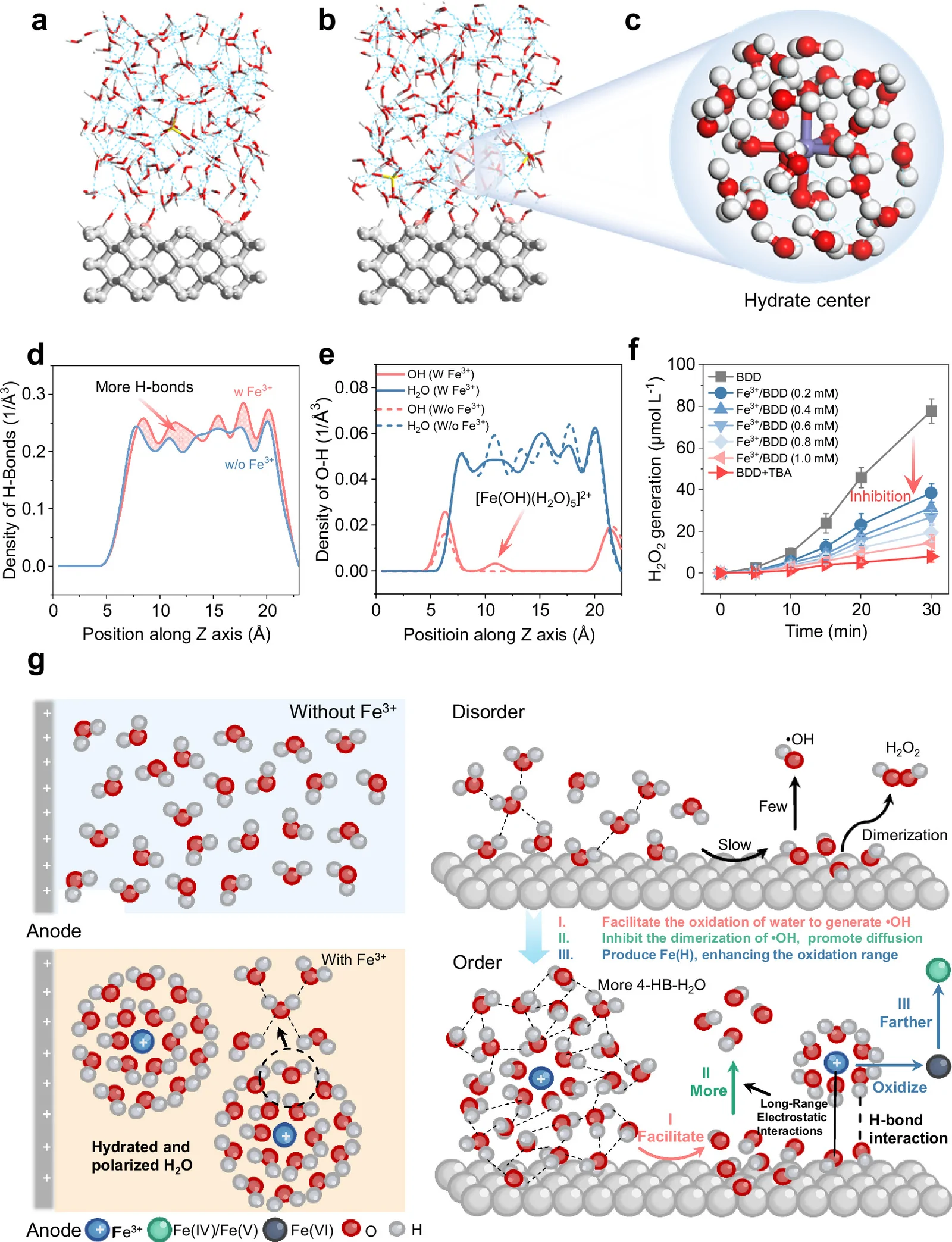
AIMD simulation of H-bond network reconfiguration and anodic oxidation mechanisms in Fe^3+^/BDD system. **a**, **b** Representative AIMD snapshots of the electrolyte with and without Fe^3+^. **c** Structure of the Fe^3+^ hydrated cluster. **d** H-bond density distribution along the electrode normal direction (Z axis). **e** Radial distribution functions of O–H bonds along the electrode normal direction. **f** H_2_O_2_ concentration during AO processes. Red arrow indicates that H_2_O_2_ production is inhibited. Error bars represent the standard deviation, obtained by repeating the experiment thrice (*n* = 3). **g** Schematic illustration of Fe^3+^-induced H-bond network reconfiguration and anodic oxidation mechanism processes. (The above shows the BDD anode oxidation process without Fe³⁺, while the below illustrates the interfacial reaction process with Fe^3+^. I, II, and III represent the three roles played after the introduction of Fe^3+^.) All atoms in the models are the same as those listed in (**g**). Experimental conditions: current density = 7.5 mA cm^−2^, [Na_2_SO_4_] = 50 mM, [TBA] = 500 mM, [Fe^3+^] = 0.2 mM. Source data are provided as a Source Data file.

To obtain direct evidence of the H-bond network of water molecules at the Fe^3+^-reconfigured interface on an atomic scale, we conducted ab initio molecular dynamics simulations (AIMD)^56–58^. The snapshots of the system provided highly intuitive images (Fig. 5a, b). In the BDD system without Fe^3+^, water molecules at the surface of the BDD electrode surface exhibited preferential orientation, but the H-bond network remained relatively loose and dynamic disorder. After introducing Fe^3+^, its dominant species [Fe(OH)(H_2_O)_5_]^2+^ acted as a powerful hydration center, inducing a highly ordered and dense secondary solvation layer of surrounding water molecules, whose structural extension far exceeded that of the first hydration shell (Fig. 5c). These visual structural differences were confirmed by quantitative statistics. The number of hydrogen bonds in the simulation system was calculated (Fig. 5d), showing that the average H-bond density per unit volume was higher in the presence of Fe^3+^ than in its absence.

The calculation results are consistent with those of ^1^H NMR, in situ Raman and FTIR spectra, showing an increase in the number of hydrogen bonds. It quantitatively confirmed that the strong hydration effect of Fe^3+^ increased the number of hydrogen bonds in the interface region. The O–H radial distribution function showed that [Fe(OH)(H_2_O)_5_]^2+^ was located 11 Å from the anode, within the electrochemical reaction zone, enabling analysis of its effect on the interfacial H-bond network (Fig. 5e). The AIMD simulations provided a solid support for the proposed mechanism of Fe^3+^-induced H-bond reconfiguration of water molecules at the atomic molecular dynamics level. It visualized the increased number of hydrogen bonds by the [Fe(OH)(H_2_O)_5_]^2+^ hydration center into a clear image. [Fe(OH)(H_2_O)_5_]^2+^ promoted the formation of 4-HB-H_2_O, weakening the O–H bonds of water molecules, laying the foundation for the subsequent efficient and low-energy barrier water oxidation process.

We observed that the pristine BDD system inherently generated 0.078 mM H_2_O_2_ during the reaction process (Fig. 5f). Upon the addition of excess TBA to scavenge •OH, H_2_O_2_ formation was almost completely suppressed, indicating that H_2_O_2_ originated from the recombination of interfacial •OH. This side reaction inevitably consumed reactive •OH, thereby reducing its effective utilization for pollutant oxidation in the BDD system. In contrast, in the Fe^3+^/BDD system, the concentration of H_2_O_2_ gradually decreased with increasing Fe^3+^ dosage. When the pH was approximately 3, Fe^3+^ and Fe(H) cannot effectively activate H_2_O_2_ to generate strong oxidizing active species (Supplementary Table 2). The removal efficiencies of MIT in the Fe^3+^/H_2_O_2_, Fe(VI)/H_2_O_2_ and Fe(VI)/Fe^3+^/H_2_O_2_ systems were all less than 20%, and the H_2_O_2_ concentration did not decline within 30 min (Supplementary Fig. 25). In addition, during the degradation of MIT by the Fe^3+^/BDD system, no significant consumption of the by-product H_2_O_2_ was observed within 5 min after the power supply was cut off (Supplementary Fig. 26). These suggested that Fe^3+^ effectively inhibited H_2_O_2_ generation, accelerating water dissociation and •OH generation. Fe^3+^ was further oxidized into Fe(H) species at the electrode surface, and suppressed the undesired •OH recombination pathway.

Based on the in situ characterization and AIMD results described above, we proposed a schematic mechanism to illustrate how Fe^3+^-induced H-bond network reconfiguration induced enhances the anodic oxidation ability and range, as depicted in Fig. 5g. The [Fe(OH)(H_2_O)_5_]^2+^ regulated the interfacial reaction at the OHP site of the BDD anode through hydrogen bonding and long-range electrostatic interactions. Its strong hydration effect can reconfigure the interfacial microenvironment of the electrode surface, significantly forming more 4-HB-H_2_O, thereby reducing the bond energy of the O–H bond in water molecules. A large number of water molecules around it were also more easily oxidized by the BDD anode directly, thereby significantly promoting the generation of •OH. Additionally, [Fe(OH)(H_2_O)_5_]^2+^ on OHP significantly inhibited the dimerization of •OH through long-range electrostatic interactions and hydrogen bonding, enhancing •OH utilization efficiency, and promoted the formation of Fe(H) species, collectively improving oxidation range, and enabling highly efficient and stable pollutant removal in the Fe^3+^/BDD system.

More importantly, the addition of Fe^3+^ significantly enhanced the pollutant removal performance of inactive anodes (such as Ti_4_O_7_), but was ineffective on active anodes (such as DSA). This suggested that the Fe^3+^-mediated H-bond network modulation strategy may also be effective for other inactive anodes, indicating potential universality (Supplementary Fig. 27).

### Application prospects of the Fe^3+^/BDD system

**Fig. 6:**
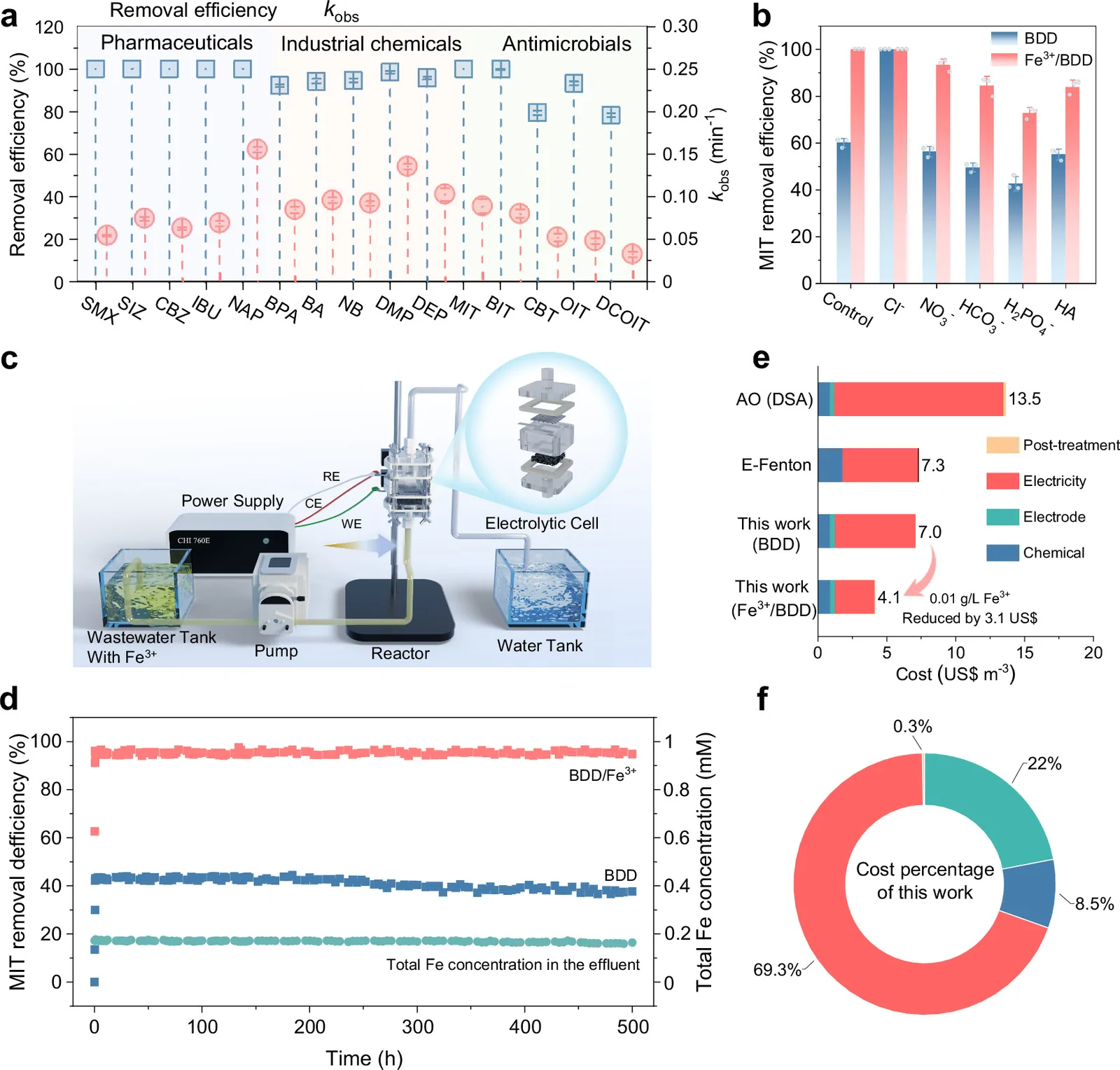
Practical application investigations of Fe^3+^/BDD system. **a** A comparison of removal efficiency and observed rate constant (*k*_obs_) for different types of pollutant in the Fe^3+^/BDD system. (SMX, sulfamethoxazole; SIZ, sulfanilamide; CBZ, carbamazepine; IBU, ibuprofen; NAP, naproxen; BPA, bisphenol A; BA, benzoic acid; NB, nitrobenzene; DMP, dimethyl phthalate; DEP, diethyl phthalate; MIT, methyl isothiazolinone; BIT, benzothiazolinone; CBT, chlorinated benzothiazolinone; OIT, 2-octyl-isothiazolinone; DCOIT, dichloro-octyl isothiazolinone). **b** Effect of anions and humic acids on the removal of MIT in the BDD and Fe^3+^/BDD systems. Error bars represent the standard deviation, obtained by repeating the experiment thrice (*n* = 3). **c** Schematic diagram and detailed enlarged view of the electrolytic cell used for the degradation of long-term flowing pollutants in the Fe^3+^/BDD system. **d** Stability tests of different systems. **e** Treatment cost per cubic meter of wastewater using four different AOP systems. **f** Proportion of each cost in this system. Detailed data are given in Supplementary Table 4. Experimental conditions: current density = 7.5 mA cm^−2^, [Na_2_SO_4_] = 50 mM, [pollution] = 20 µM, [Fe^3+^] = 0.2 mM, [Cl⁻] =  [NO_3_⁻] = [HCO_3_⁻] = [H_2_PO_4_⁻] = 5 mM, [HA] = 10 mg C/L. Source data are provided as a Source Data file.

To assess the broad application potential of the Fe^3+^/BDD system, we systematically investigated its degradation performance towards 15 types of organic pollutants, encompassing pharmaceuticals, industrial chemicals, and antimicrobials. As depicted in Fig. 6a and Supplementary Figs. 28–35, all contaminants were effectively eliminated within 30 min, even for difficult to degrade pollutants such as SMX, SIZ, CBZ, IBU, NPX, and MIT. The proportion of reactive species that were removed by BIT, OIT, CBT and DCOI was almost the same as that of MIT in the Fe^3+^/BDD system (Supplementary Figs. 29–34). Furthermore, the TOC removal rates of the five isothiazolinones by BDD anode oxidation increased by more than double after the addition of Fe^3+^ (Supplementary Fig. 35). These results demonstrate that the Fe^3+^-induced BDD system remarkably enhances the mineralization ability and achieves the efficient and stable removal of diverse emerging organic pollutants.

We systematically investigated the impact of typical ions (e.g., Cl⁻, HCO_3_⁻, NO_3_⁻ and H_2_PO_4_⁻) and humic acid (HA) in actual wastewater on the degradation of MIT, in order to assess the potential for practical application (Fig. 6b). In the BDD system, Cl⁻ was the sole species identified to promote MIT removal, and all others exerted a slight inhibitory effect. Because Cl⁻ can be oxidized to active chlorine species at the anode to promote pollutant degradation. In the Fe^3+^/BDD system, although other anions and HA still had some inhibitory effects, their tolerance was significantly stronger than that of the BDD system. This is due to the fact that the oxidation process of Fe(H) is less affected by quenching agents in the solution, maintaining a high selective oxidation ability^42^. The Fe^3+^/BDD system achieved stable MIT degradation efficiency (more than 80% in 30 min) across pH 1–10 (Supplementary Fig. 36), demonstrating broad pH tolerance. Collectively, these findings indicate that the Fe^3+^/BDD system not only has a broad range of pollutant degradation capability but also exhibits strong resistance to interference from complex water matrices, laying a solid foundation for its treatment of actual wastewater.

To promote the practical application of this technology, we designed a laboratory-scale long-term flow reactor (Fig. 6c) and assessed its long-term performance for MIT removal. The concentration range of MIT in actual sewage is quite wide, ranging from ~µg/L to nearly 200 mg/L^59,60^. Here, a MIT concentration at a moderate level (2.3 mg/L) was selected. Over continuous operation for 500 h, the system maintained a stable MIT removal efficiency exceeding 90% on average (Fig. 6d). After the Fe^3+^ was oxidized to Fe(H) at the anode, its reduction product was Fe^3+^, enabling a rapid Fe^3+^-Fe(H)-Fe^3+^ cycle (Supplementary Fig. 37). Fe^3+^ addition did not trigger passivation of the BDD electrode. Moreover, the BDD electrode itself has extremely high physical and chemical stability, with strong corrosion resistance, ensuring the durability of the reaction platform.

We conducted a further economic analysis of the Fe^3+^/BDD system to assess its practical application prospects. As shown in Fig. 6e and Supplementary Table 4, compared with traditional water treatment processes (AO (DSA), E-Fenton, and AO (BDD)), the Fe^3+^/BDD process proposed in this work significantly reduces the treatment cost. Based on the cost calculation of achieving the same degradation standard for each unit of wastewater, the BDD/Fe^3+^ system only introduced 0.01 g/L of Fe^3+^ and reduced the operating cost by 44.3% compared to the traditional BDD system. This significant cost advantage mainly stems from the substantial reduction in power consumption. As shown in Fig. 6f, the cost composition of the Fe^3+^/BDD system mainly included electricity consumption (69.3%), electrode loss (6.5%), chemical reagents (22%), and post-treatment (0.3%). Although the initial fabrication cost of the BDD electrode remained relatively high, its extremely long service life has spread the single treatment cost. The addition of trace Fe^3+^ has brought about significant energy-saving effects, resulting in a significant reduction in the overall cost.

The Fe^3+^/BDD system constructed through the water molecule engineering strategy successfully overcame the key bottlenecks faced by the anodic oxidation technology, such as high energy consumption and weak anti-interference ability, providing a convenient technical paradigm and solid scientific foundation for achieving efficient, stable, and low-cost treatment of refractory organic wastewater.

In summary, we introduced Fe^3+^ into the BDD anodic oxidation system to regulate the H-bond network of water molecules at the anode interface, significantly enhancing the kinetics of water oxidation and expanding the anodic oxidation range. The *operando* electrochemical and in situ spectroscopic results indicated that the strong hydration and polarization ability of [Fe(OH)(H_2_O)_5_]^2+^ restructured the H-bond network of water molecules, increasing the proportion of 4-HB-H_2_O by 16% and weakened the O–H bond, thereby significantly reducing the energy barrier for the generation of •OH during the anodic water oxidation process. Through long-range electrostatic interactions and hydrogen bonding, [Fe(OH)(H_2_O)_5_]^2+^ inhibited the dimerization of •OH to produce H_2_O_2_, promoting the diffusion of •OH. As a low-energy barrier oxidation center, it generated Fe(H), broadening the oxidation range of the anode. This interface microenvironmental regulation engineering was able to increase the *k*_obs_ of MIT removal by three times, reduced the operating cost by 44.3%, and achieved continuous operation for 500 h. This study proposed a convenient approach for regulating the hydrogen bond network of water molecules at the interface, addressing the inherent limitations in the anodic oxidation process and providing a new perspective for enhancing the anodic oxidation performance of non‑active anodes.

## Methods

### Chemicals and reagents

A complete list of chemicals and reagents is provided in Supplementary Text 1.

### Oxidation experiments

The experiment was conducted in a 250 mL H-type electrolyzer equipped with a proton exchange membrane (Nafion NR211, Dupont, U.S.A.) and magnetic stirring. A predetermined concentration of contaminants and electrolyte solution was introduced into the anode chamber, while an identical concentration of electrolyte was added to the cathode chamber. The BDD (20 × 20 × 1 mm, New Frontier Technology, China) anode and the stainless-steel cathode are connected to a regulated direct current (DC, HSPY-60-02, China) power supply. During the reaction process, the current density remains constant. Samples were collected at specified time intervals and transferred into 1 mL sample vials for subsequent analysis. The initial pH of the reaction mixture was adjusted using 0.1 M H_2_SO_4_ and 0.1 M NaOH. All experiments were conducted in triplicate from distinct samples, and the results are presented as means with standard deviations. All tests were conducted under pH 3 conditions.

### Analytical methods

The concentration of total organic carbon (TOC) was detected by a TOC analyzer (TOC-L, SHIMADZU, Japan). The pH was measured using a pH meter (pHS-3C, Lei-ci, China). The organic compounds were quantified by high‒performance liquid chromatography (HPLC 1260, Agilent, U.S.A.) equipped with a G1316A variable wavelength UV detector and an Eclipse XDBC-18 (5 μm, 4.6 × 250 mm) column (detailed conditions for the HPLC tests are provided in Table S1). The concentration of Fe^3+^ and H_2_O_2_ was quantified via ultraviolet–visible spectrophotometry (UV1800, SHIMADZU, Japan) (Supplementary Texts 2 and 3). Electron paramagnetic resonance (EPR) spectra were acquired using a Bruker EMX plus X-band CWEPR spectrometer (BRUKER, Germany) (Supplementary Text 4). Nuclear magnetic resonance hydrogen (^1^H NMR and 2D DOSY NMR) spectra were recorded at room temperature on an AMX-400 (BRUKER, Germany) spectrometer (400 MHz for ¹H NMR).

### Electrochemical measurements

All electrochemical performance tests were conducted on an electrochemical workstation (CHI 760E, CH Instrument Company, Shanghai Chenhua, China). A three-electrode configuration was employed, with a platinum wire as the working electrode, a saturated calomel electrode as the reference electrode, and BDD as the counter electrode. Unless otherwise specified, all potentials were referenced to the saturated calomel electrode. All electrochemical measurements were carried out at room temperature (approximately 25 °C). The cyclic voltammogram (CV) was recorded under a scan rate of 50 mV/s. The electrochemical impedance spectroscopy (EIS) measurement was conducted under a frequency range of 0.01 to 10^6^ Hz. The Tafel plot shows the logarithm of the absolute current density versus applied potential, fully displaying the entire measured potential range without truncation. iR-compensation was not applied in this study due to the negligible ohmic drop estimated from the solution resistance.

### Electrochemical in situ Raman spectroscopy

In situ Raman spectroscopy characterizations were carried out using a inVia confocal Raman microscope (HORIBA Xplora Plus, Renishaw, England). The laser power was set at 532 nm with 1% power at a grating of 1800 1/mm. The silicon peak was calibrated before testing. Platinum wires and a saturated calomel electrode were used as the counter electrode and reference electrode, respectively.

### Electrochemical in situ Fourier transform infrared (FTIR) spectroscopy

The instrument was equipped with an electrochemical in situ cell accessory with a silicon internal reflection element (IRE), and used the Nicolet iS50 Fourier transform infrared spectrometer (Thermo Scientific, U.S.A.). The test mode was the internal reflection Otto thin layer mode. The electrodes were square with a side length of 10 mm, and were firmly pressed onto the silicon IRE using glass carbon electrodes as the working electrode. Platinum wires and a saturated calomel electrode were used as the counter electrode and reference electrode, respectively.

### Ab initio molecular dynamics (AIMD) theoretical simulation

We apply molecule dynamics simulation to simulate the electric double layers that were mode by combining the Boron-dopped diamond surface and explicit water molecules, Fe^3+^, H^+^, SO_4_^2^⁻ and Na^+^. For reducing the simulation cost, the molecular dynamic simulations were firstly performed using extended tight binding method (XTB) implemented in CP2K package for 50 ps to equilibrate the water solution^61^. Furthermore, we applied AIMD simulations to equilibrate the system for 20 ps to better simulate physical-chemical interface between the solution and surface. The AIMD simulations were performed using the CP2K package as well. The exchange-correlation energy was computed by using generalized-gradient approximation in the parameterization of Perdew, Burke, and Ernzerhof (PBE)^62^. The dispersion interaction was considered by using the empirical parameterized Grimme (D3) method^63^. The valence-shell electrons of all elements in simulation are described as 1*s*^1^ for H, 2*s*^2^2*p*^2^ for C, 2*s*^2^2*p*^4^ for O, 2*s*^2^2*p*^6^3*s*^1^ for Na, 3*s*^2^3*p*^4^ for S, and 3*s*^2^3*p*^6^3*d*^6^4*s*^2^ for Fe by using hybrid Gaussian and plane-wave (GPW) basis sets at the level of triple-ζ valence plus polarization that were optimized to minimize basis set superposition errors^64^. The cutoff energy of 600 Rydberg of auxiliary plane wave basis sets was adopted. The Core electrons were described with scalar relativistic norm-conserving pseudopotentials. Brillouin zone integration was performed with a reciprocal space mesh consisting of only the gamma point. In the simulations, we used the Nose–Hoover thermostat (NVT) to simulate the dynamic evolution of the model of electric double layers^65^. A time step of 1.0 fs was used to simulate the system for both the DFT and DFTB method. The statistical sampling was performed at temperature of 298 K.

### Reporting summary

Further information on research design is available in the Nature Portfolio Reporting Summary linked to this article.

## Supplementary information

Supplementary Information

Reporting Summary

## Source data

Source Data

Transparent Peer Review File

## Data Availability

The data supporting the findings of the study are included in the main text and Supplementary Information files. Additional data are available from the corresponding author upon request. Source data are provided with this paper.
