# Supplementary material for: Reconfiguring interfacial hydrogen-bond networks to enhance anodic oxidation for efficient wastewater treatment
Source: Nat Commun. 2026 Oct 5;17:11322. doi: 10.1038/s41467-026-78208-2 (PMC13724709; doi:10.1038/s41467-026-78208-2)
Supplement: Supplementary file 1 [file 41467_2026_78208_MOESM1_ESM.pdf]

Reconfiguring Interfacial Hydrogen-Bond Networks to Enhance Anodic Oxidation for Efficient Wastewater Treatment

Tong Liu[a,b 1], Shuang Zhong[a,b 1], Xiaotong Liang[c], Hongrui Zhu[d], Ye Du[a,b*] & Bo Lai[a,b*]

[a] *State Key Laboratory of Hydraulics and Mountain River Engineering, College of Architecture and Environment, Sichuan University, Chengdu 610065, China*

[b] *Sino-German Centre for Water and Health Research, Sichuan University, Chengdu 610065, China*

[c] *Key Laboratory of Green Chemistry and Technology of Ministry of Education, College of Chemistry, Sichuan University, Chengdu, 610065, China*

[d] *College of Materials Science and Engineering, Sichuan University, Chengdu 610065, China*

[1] *These authors contributed equally: Tong Liu and Shuang Zhong*

[*] *Corresponding authors: Ye Du and Bo Lai*

[*] Corresponding authors.
E-mail address: duyeah@scu.edu.cn (Ye Du)
[*] Corresponding authors.
E-mail address: laibo@scu.edu.cn (Bo Lai)

Content

Supplementary Methods……………………………………..………..………………...3

Supplementary Fig. 1 to 36……………………………………………..………………14

Supplementary Tables 1 to 4…………………………………………………………51

Supplementary References……………………………………………………….….57

Supplementary Text 1

Chemicals and Reagents.

Potassium dihydrogen phosphate (KH_2PO_4, 98%), sodium chloride (NaCl, 99.5%), sodium nitrate ($NaNO_3$), sodium bicarbonate ($NaHCO_3$, 99.5%), sodium sulfate (Na_2SO_4), hydrogen peroxide (H_2O_2, 30%), and ferric sulfate ($Fe_2(SO_4)_3$) were offered by kelong chemical co., LTD (Chengdu, China). Tertiary butanol (TBA, 99%) and methanol (MeOH, 99.5%) were provided by Macklin Biochemical Technology Co., Ltd (Shanghai, China). Diphenyl sulfoxide (PMSO, 98%), phenyl methyl sulfone ($PMSO_2$, 97%), humic acid (HA, fulvic acid ≥ 90%), 2-Methyl-4-isothiazolin-3-one (MIT, 95%), 1,2-Benzisothiazolin-3-one (BIT, 99%), 5-Chloro-2-benzothiazolinone (CBT, 98%), 2-Octyl-4-isothiazolin-3-one (OIT, 98%), 4,5-Dichloro-2-octyl-4-isothiazolin-3-one (DCOIT, 98%), Sulfamethoxazole (SMX, 98%), Sulfisoxazole (SIX, 98%), Carbamazepine (CBZ, 98%), Ibuprofen (NAP, 98%), Naproxen (NAP, 99%), Bisphenol A (BPA, 99%), Diethyl phthalate (DEP, 99%), Dimethyl phthalate (DMP, 99%), 5,5-Dimethyl-1-Pyrrolin-N-Oxide (DMPO, 97%), dimethyl sulfoxide (DMSO, 99.9%), nitrobenzene (NB, 99%), benzoic acid (BA, 99.5%) and were bought from Aladdin Biochemical Technology Co., Ltd (Shanghai, China). Methanol and acetonitrile were purchased from Anpu Experimental Technology Co., Ltd (Shanghai, China).

Supplementary Text 2

Methods for analyzing the concentration of iron species.

The concentrations of iron species were analyzed by spectrophotometric methods. For the analysis, 10 mM phenanthroline and 100 mM ascorbic acid were prepared before reaction. Samples (6 mL) were withdrawn from the reaction solution at specific time intervals. 3 mL filtered sample was mixed with 0.1 mL 10 mM phenanthroline and 0.1 mL 100 mM ascorbic acid. After shaking for 30 s, the absorbance of mixed solution was detected by an UV-vis spectrometer at 510 nm for measuring the concentrations of total dissolved iron. The other sample (3 mL) was mixed with 0.1 mL 10 mM phenanthroline. Detection method was same as above to measure the dissolved Fe(II).

Supplementary Text 3

Methods for analyzing the concentration of H_2O_2.

The H_2O_2 concentration was detected by spectrophotometric method using an UV-vis spectrophotometer (UV2355, UNICO, Shanghai, China). Firstly, 50 mM potassium titanium oxalate (PTO) and 3 M sulfuric acid were prepared before reactions. Pre-added 0.5 mL PTO and 0.5 mL sulfuric acid in 5 mL sampling tubes. Samples were withdrawn from the reaction solution at specific time intervals. Then, 3 mL sample was mixed with 1 mL mixture as quickly as possible. After 30 ± 2s, the absorbance of mixed solution was detected by UV-vis spectrophotometer at 396 nm to detect the concentration of H_2O_2.

Supplementary Text 4

Method for detecting reactive species using EPR.

2 mL sample to be tested and 20 μL of the spin-trapping agent (DMPO) were thoroughly mixed to form a relatively stable adduct. Then, the sample was placed into a capillary (0.95 mm×1.3 mm×125 mm, RS-CP-50) for EPR detection. The test parameters were as follows: modulation = 0.1 mT, modulation frequency = 100 kHz, center field = 336.5 mT, microwave power = 2.5 mW, sweep width = 16 mT, sweep time = 30 s.

Supplementary Text 5

Calculation of apparent reaction rate constant.

The reaction rate was evaluated by a first-order kinetics model as the following equation:

$$\ln \frac{C_0}{C_t} = -k_{obs} t \qquad \text{(S1)}$$

where C_0 is the initial pollutant concentration, C_t is the concentration at a specific time t during the degradation process, and k_{obs} is the apparent reaction rate constant.

Supplementary Text 6

Contribution of the active species calculated from the results of the quenching experiment.

The contribution of active species is calculated according to the pseudo-first-order rate constant of quenching experiments, and the equation is as follows:

$$CR_{\cdot OH} = \frac{k_0 - k_{MeOH}}{k_0} \times 100\% \tag{S5}$$

$$CR_{Fe(H)} = \frac{k_{MeOH} - k_{DMSO}}{k_0} \times 100\% \tag{S6}$$

$$CR_{DO} = 100 - CR_{\cdot OH} - CR_{Fe(H)} \tag{S7}$$

where k_0 is the pseudo-first-order rate constant of ITOs degradation without quenching agent, k_{MeOH} or k_{DMSO} is the pseudo-first-order rate constant of ITOs degradation when adding quenching agent, CR is the contribution rate.

Supplementary Text 7

Determination of the steady-state concentrations of •OH and Fe (H) by probe experiments.

In order to evaluate the enhancing effect of Fe^{3+} addition on the •OH in the BDD system, NB was used as a •OH probe to estimate the concentration of •OH. It is reported that in the oxidation system involving Fe(H), Fe(H) is not the cause of NB degradation. Moreover, the reaction activity of NB with •OH is very high, and the second-order rate constant is 3×10^{9} M^{-1} s^{-1} [1]. Therefore, in the Fe^{3+}/BDD system, the degradation of NB is caused by •OH rather than Fe(H). The degradation kinetics of NB in the Fe^{3+}/BDD system were described in Equations S1. The steady-state concentration of •OH can be reliably evaluated according to the following equation.

$$\frac{d[NB]}{dt} = -k_{NB,\cdot OH}[\cdot OH]_{ss}[NB] \quad \text{(S2)}$$

$$\ln\frac{[NB]}{[NB]_0} = -k_{NB,\cdot OH}\int_0^t[\cdot OH]dt = -k_{obs,NB}t \quad \text{(S3)}$$

$$[\cdot OH]_{SS} = \frac{\int_0^t[\cdot OH]dt}{t} = \frac{k_{obs,NB}}{k_{NB}} \quad \text{(S4)}$$

Where [NB] denote the molar concentrations of NB at time t after reacting with the oxidant; $[NB]_0$ represent the initial molar concentration of NB; $k_{NB,\cdot OH}$ are the second-order rate constants for the reactions of NB with •OH (M^{-1} s^{-1}); $[\cdot OH]_{ss}$ denote to the steady-state concentrations of •OH at time t; the term $\int_0^t[\cdot \mathrm{OH}]\,dt$ represents the cumulative exposure to •OH, i.e. the time-integrated •OH concentration; $k_{obs,NB}$ refers to the pseudo-first-order rate constants of NB in the

Fe^{3+}/BDD system.

To evaluate the role of Fe^{3+} addition in generating Fe(IV)/Fe(V) in the BDD system, PMSO was used as a probe for Fe(IV)/Fe(V) to estimate their concentrations. Due to pH effects, Fe(V) rapidly deactivates under acidic conditions; therefore, Fe(IV) was considered the primary active iron species for estimation. Additionally, the second-order rate constant between Fe(IV) and PMSO was determined to be 2.58 × 10^3 M^{-1} s^{-1}. In the Fe^{3+}/BDD system, after adding excess MeOH to completely quench •OH, the degradation of PMSO was primarily driven by Fe(IV). The steady-state concentration of Fe(IV) could thus be reliably estimated using the following equation.

$$\frac{d[PMSO]}{dt} = -k_{PMSO,Fe(IV)}[Fe(IV)]\,[PMSO] \tag{S5}$$

$$\ln\frac{[PMSO]}{[PMSO]_0} = -k_{PMSO,Fe(IV)}\int_0^t [Fe(IV)]dt = -k_{obs,PMSO}t \tag{S6}$$

$$[Fe(IV)] = \frac{\int_0^t [Fe(IV)]dt}{t} = \frac{k_{obs,PMSO}}{k_{PMSO}} \tag{S7}$$

Where [PMSO] denote the molar concentrations of PMSO at time t after reacting with the oxidant; $[PMSO]_0$ represent the initial molar concentration of PMSO; $k_{PMSO,Fe(IV)}$ are the second-order rate constants for the reactions of PMSO with Fe(IV) (M^{-1} s^{-1}); [•OH]ss denote to the steady-state concentrations of Fe(IV) at time t; the term $\int_0^t [Fe(IV)]dt$ represents the cumulative exposure to Fe(IV), i.e. the time-integrated Fe(IV) concentration; $k_{obs,PMSO}$ refers to the pseudo-first-order rate constants of PMSO in the Fe^{3+}/BDD system.

Supplementary Text 8

Calculation of the second-order rate constants for the reactions of Fe(IV)/Fe(V) with MIT in the Fe^{3+}/BDD system

Determine the k_{obs} of Fe(IV)/Fe(V) toward MIT in the Fe^{3+}/BDD system through competitive kinetics experiments. Adding excess MeOH completely quenches the •OH in the system. Equal amounts of MIT and PMSO were added to the system for degradation experiments, and the degradation of both was attributed to Fe(IV)/Fe(V). Calculations were performed according to formula S8.

$$k_{Fe(IV),MIT} = \frac{\ln(\frac{[MIT]_0}{[MIT]})}{\ln(\frac{[PMSO]_0}{[PMSO]})} \times k_{Fe(IV),PMSO} \quad \text{(S8)}$$

Where [PMSO] and [MIT] denote the molar concentrations of PMSO and MIT at time t after reacting with the oxidant; $[PMSO]_0$ and $[MIT]_0$ represent the initial molar concentration of MIT and PMSO; $k_{Fe(IV),MIT}$ and $k_{Fe(IV),PMSO}$ are the second-order rate constants for the reactions of MIT and PMSO with Fe(IV) (M^{-1} s^{-1}).

Supplementary Text 9

Calculation of the $k_{obs,MIT}$ for •OH and Fe(IV)/Fe(V) in the Fe^{3+}/BDD system

The $k_{obs,MIT}$ of •OH and Fe(IV)/Fe(V) can be obtained through formula S9 and formula S10.

$$k_{obs,MIT} = [\cdot OH] \times k_{\cdot OH,MIT} \quad \text{(S9)}$$

$$k'_{obs,MIT} = [Fe(IV)] \times k_{Fe(IV),MIT} \quad \text{(S10)}$$

Where [•OH] and [Fe(IV)] denote the molar concentrations of •OH and Fe(IV); $k_{\cdot OH,MIT}$ and $k_{Fe(IV),MIT}$ are the second-order rate constants for the reactions of •OH and Fe(IV) with MIT (M^{-1} s^{-1}); $k_{obs,MIT}$ and $k'_{obs,MIT}$ represent the apparent reaction rates of •OH and Fe(IV) with MIT in Fe^{3+}/BDD system.

Supplementary Text 10

Finite Element Analysis (FEA) Simulation.

The concentration distribution of •OH and Fe(H) on the surface of the BDD electrode was simulated using COMSOL Multiphysics 6.2 software. A two-dimensional geometry "Transient" module along with a "Cube Current Distribution" module were employed to address the material distribution issue on the BDD electrode surface[2, 3]. The dynamic simulation of the reaction process was conducted utilizing the Butler-Volmer and Nernst-Planck equations[4]. The electrode double layer thickness was set to approximately 10 μm, which is a typical steady-state Nernst electrical double layer for a non-stirred system. The bulk concentration of Fe^{3+} was set to the experimentally optimized value of 0.2 mM. The current density is set at 7.5 mA cm^{-2}. The diffusion coefficient of •OH in aqueous solution was set to about 2.0×10^{-9} m^2 s^{-1}, which is an approximation commonly adopted in electrochemical models for small molecules. For the high valent iron species, a slightly lower value of about 0.6×10^{-9} m^2 s^{-1} has been used, which is comparable to the reported diffusion coefficients of iron cyanide complexes in aqueous electrolytes. This choice accounts for the larger size of the Fe(H) compared to the •OH.

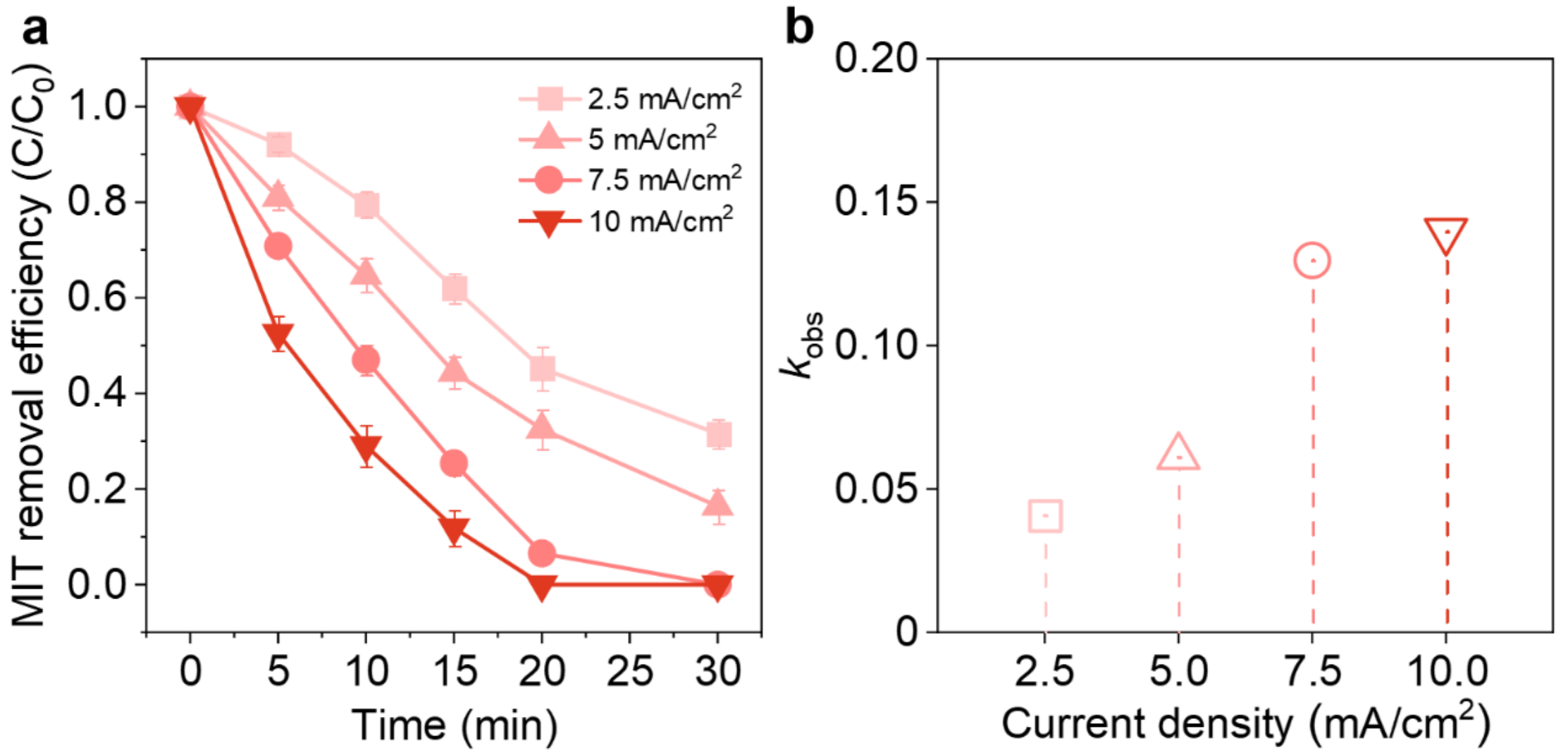

Supplementary Fig. 1. a. Efficiency of different current density on the oxidation removal of MIT by Fe^{3+}/BDD. **b.** k_{obs} of different current density on MIT removal by Fe^{3+}/BDD. Experimental conditions: [Na_2SO_4] = 50 mM, [MIT] = 20 μM, [Fe^{3+}] = 0.2 mM. Source data are provided as a Source Data file.

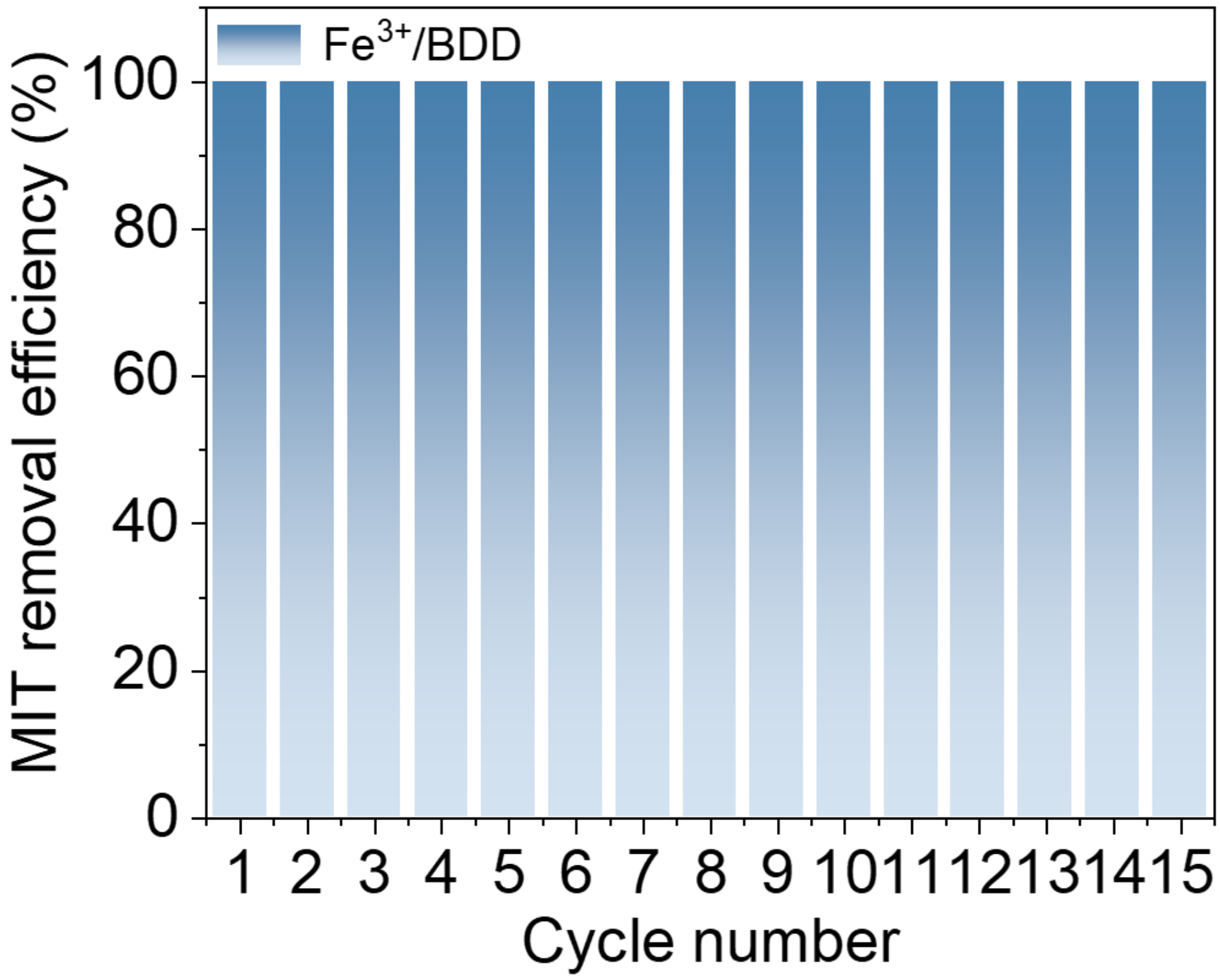

Supplementary Fig. 2. Cyclic stability test of the Fe^{3+}/BDD system for the removal of MIT. Experimental conditions: [Na_2SO_4] = 50 mM, [MIT] = 20 μM, [Fe^{3+}] = 0.2 mM, current density = 7.5 mA cm^{-2}. Source data are provided as a Source Data file.

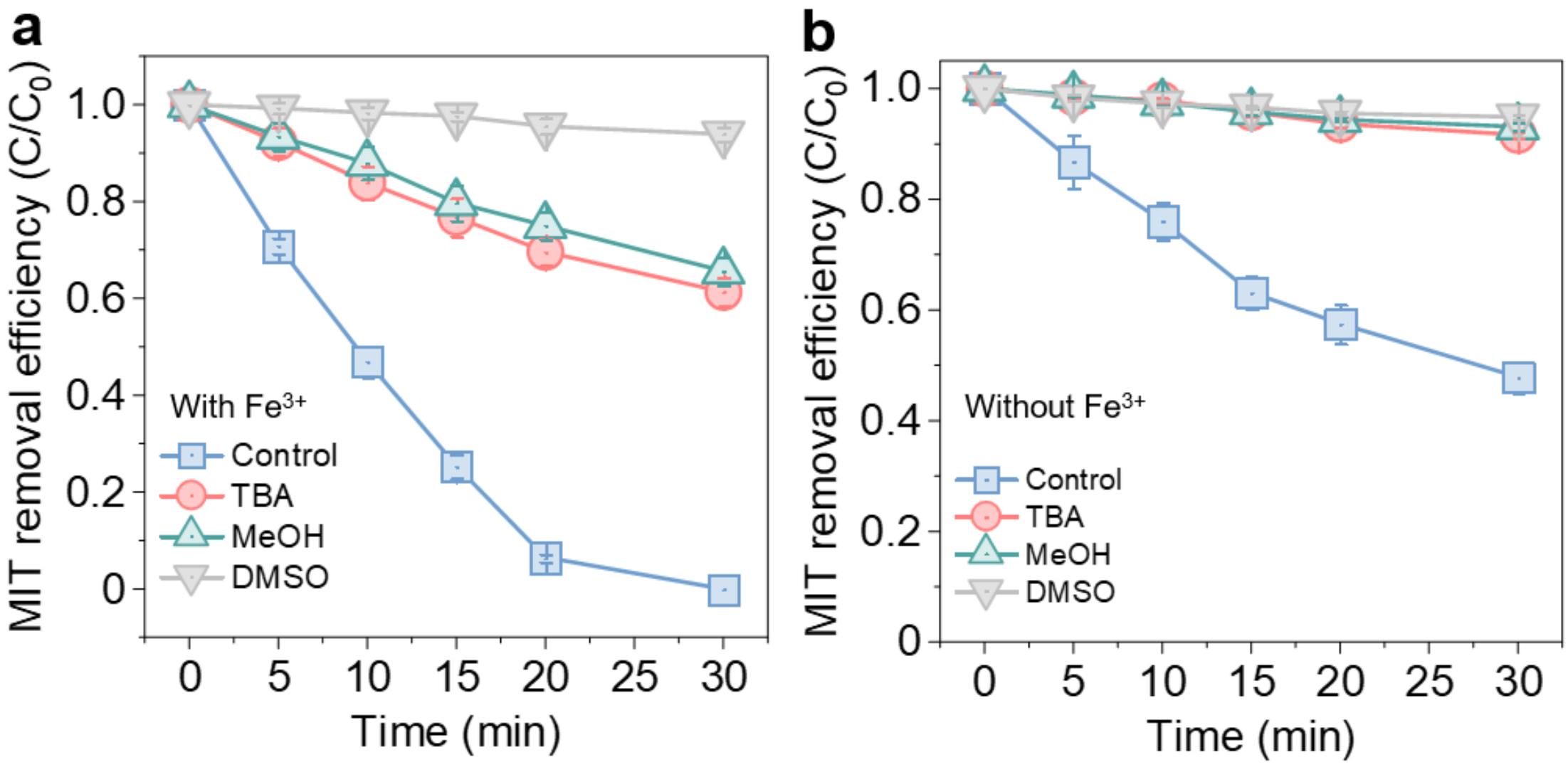

Supplementary Fig. 3. a. Effects of various quenchers (TBA, MeOH and DMSO) on the elimination of MIT in Fe^{3+}/BDD system. **b.** Effects of various quenchers (TBA, MeOH and DMSO) on the elimination of MIT in BDD system. Experimental conditions: [Na_2SO_4] = 50 mM, [MIT] = 20 μM, [Fe^{3+}] = 0.2 mM, [TBA] = [MeOH] = 200 mM, [DMSO] = 50 mM, current density = 7.5 mA cm^{-2}. Source data are provided as a Source Data file.

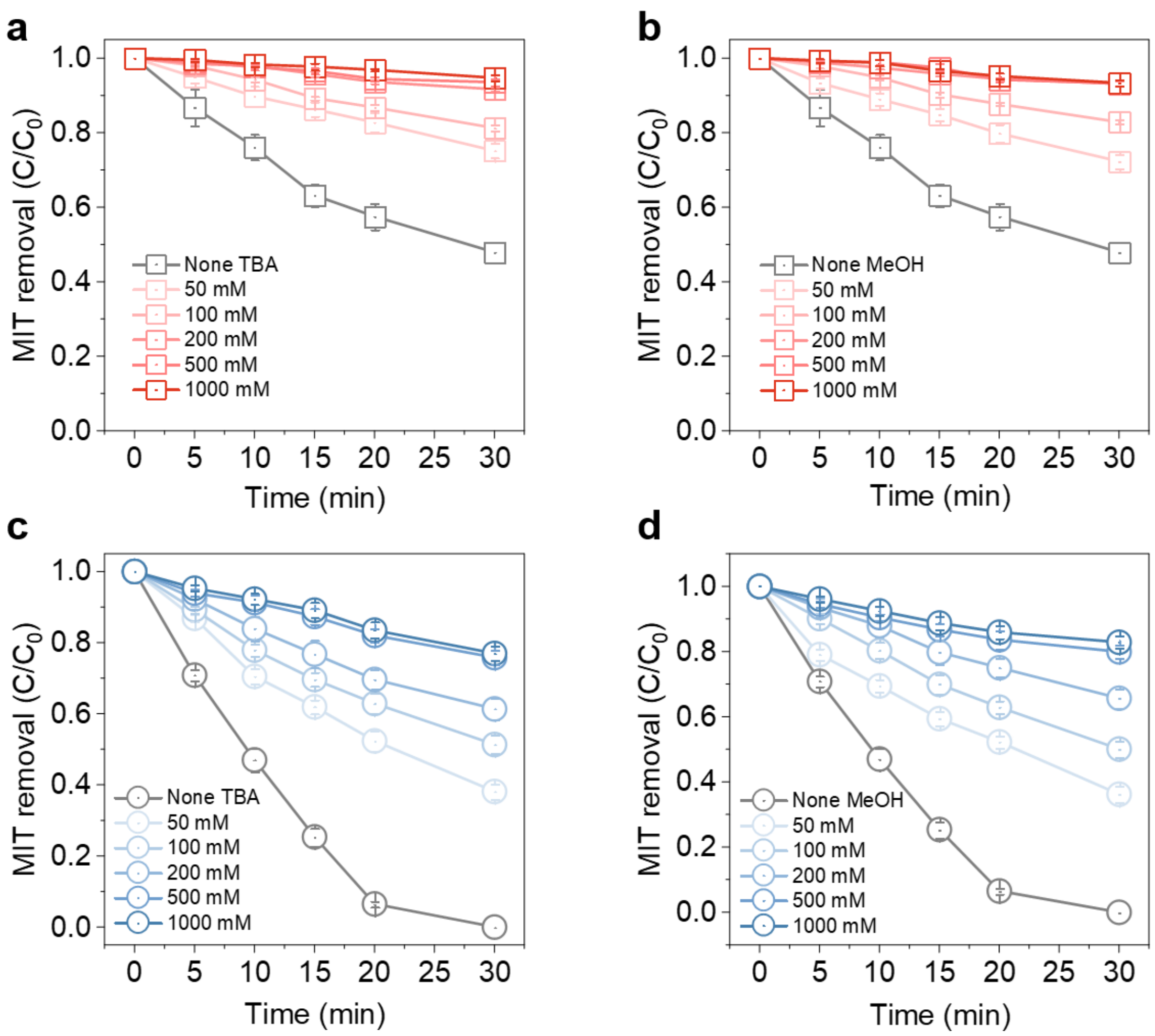

Supplementary Fig. 4. a and b. Effect of different TBA and MeOH concentrations on the attenuation of MIT in the BDD system. **c and d.** Effect of different TBA and MeOH concentrations on the attenuation of MIT in the Fe^{3+}/BDD system. Error bars represent the standard deviation, obtained by repeating the experiment thrice (n = 3). Experimental conditions: [Na_2SO_4] = 50 mM, [MIT] = 20 μM, [Fe^{3+}] = 0.2 mM, current density = 7.5 mA cm^{-2}. Source data are provided as a Source Data file.

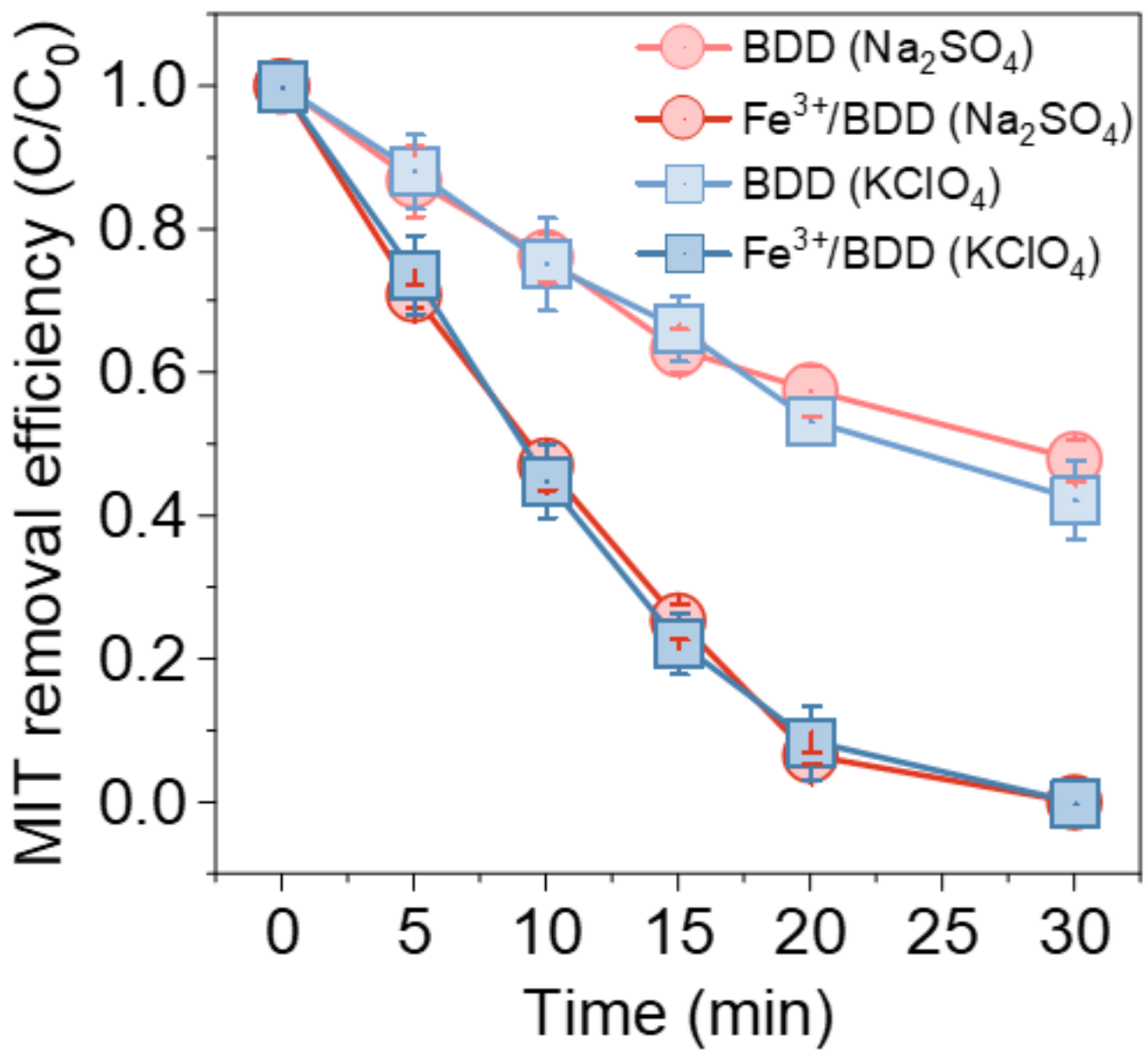

Supplementary Fig. 5. Effect of different electrolytes (Na_2SO_4 and $KClO_4$) on MIT degradation efficiency in BDD and Fe^{3+}/BDD system. Error bars represent the standard deviation, obtained by repeating the experiment thrice (n = 3). Experimental conditions: [Na_2SO_4] = [$KClO_4$] =50 mM, [MIT] = 20 μM, [Fe^{3+}] = 0.2 mM, current density = 7.5 mA cm^{-2}. Source data are provided as a Source Data file.

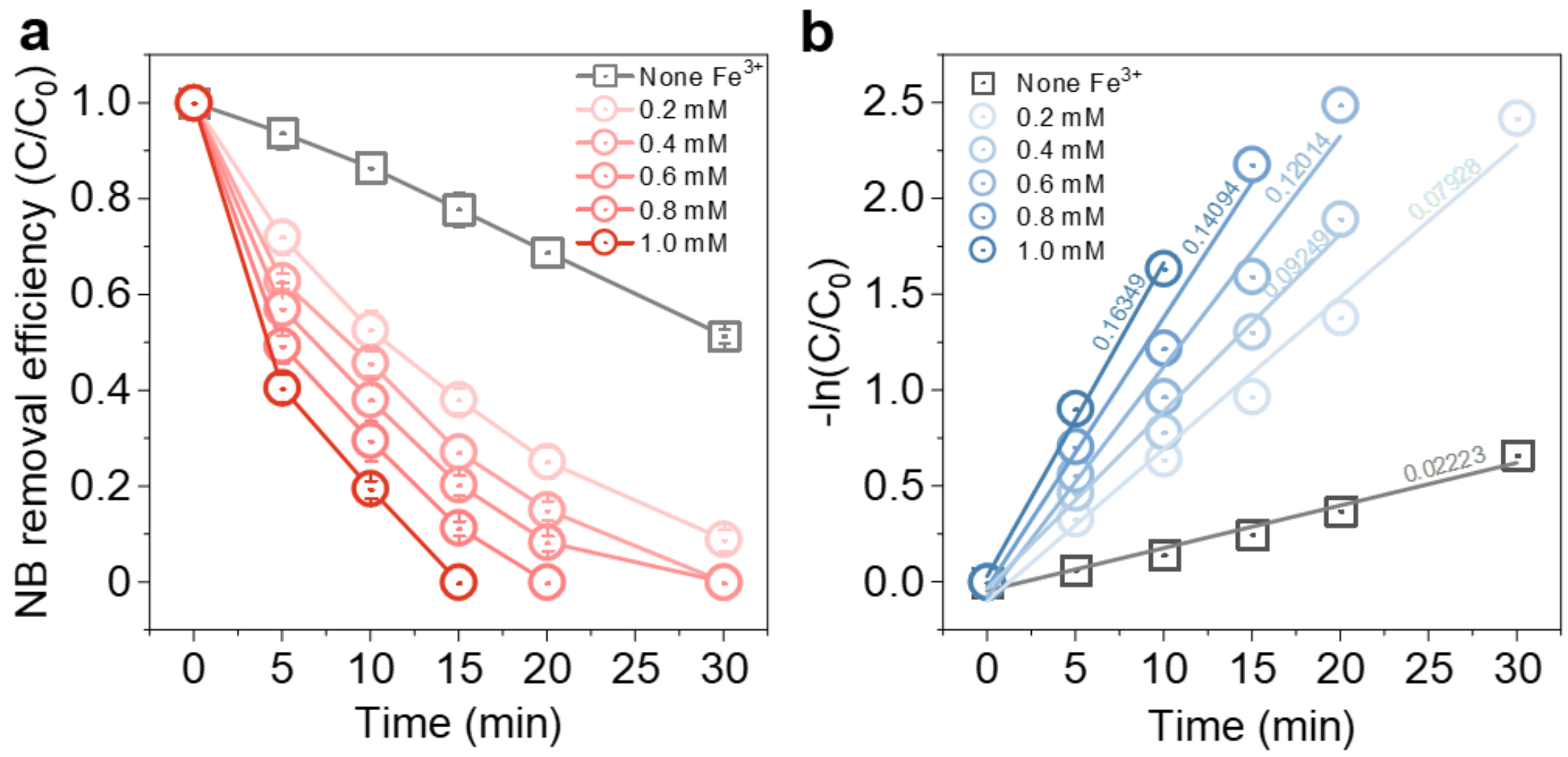

Supplementary Fig. 6. Probe experiments of NB as an •OH probe in Fe^{3+}/BDD system with different Fe^{3+} concentrations: **a.** the decay situation of the probe compound, **b.** the corresponding first-order kinetic curve. Error bars represent the standard deviation, obtained by repeating the experiment thrice (n = 3). Experimental conditions: [Na_2SO_4] = [$KClO_4$] =50 mM, [NB] = 20 μM, current density = 7.5 mA cm^{-2}. Source data are provided as a Source Data file.

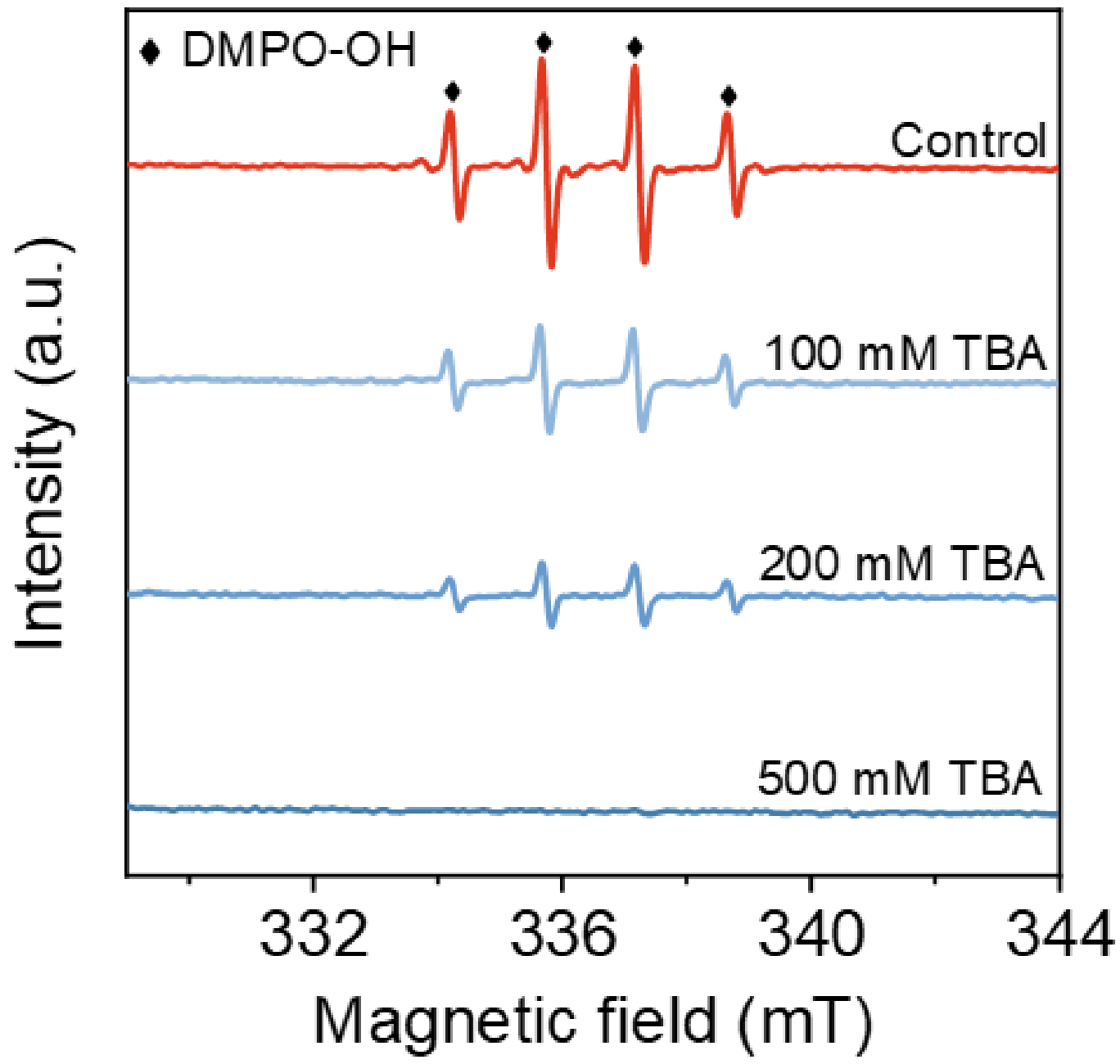

Supplementary Fig. 7. EPR spectra of DMPO-OH after adding TBA to the Fe^{3+}/BDD system. Experimental conditions: [Na_2SO_4] = 50 mM, [DMPO] = 20 μM, [Fe^{3+}] = 0.2 mM, current density = 7.5 mA cm^{-2}. Source data are provided as a Source Data file.

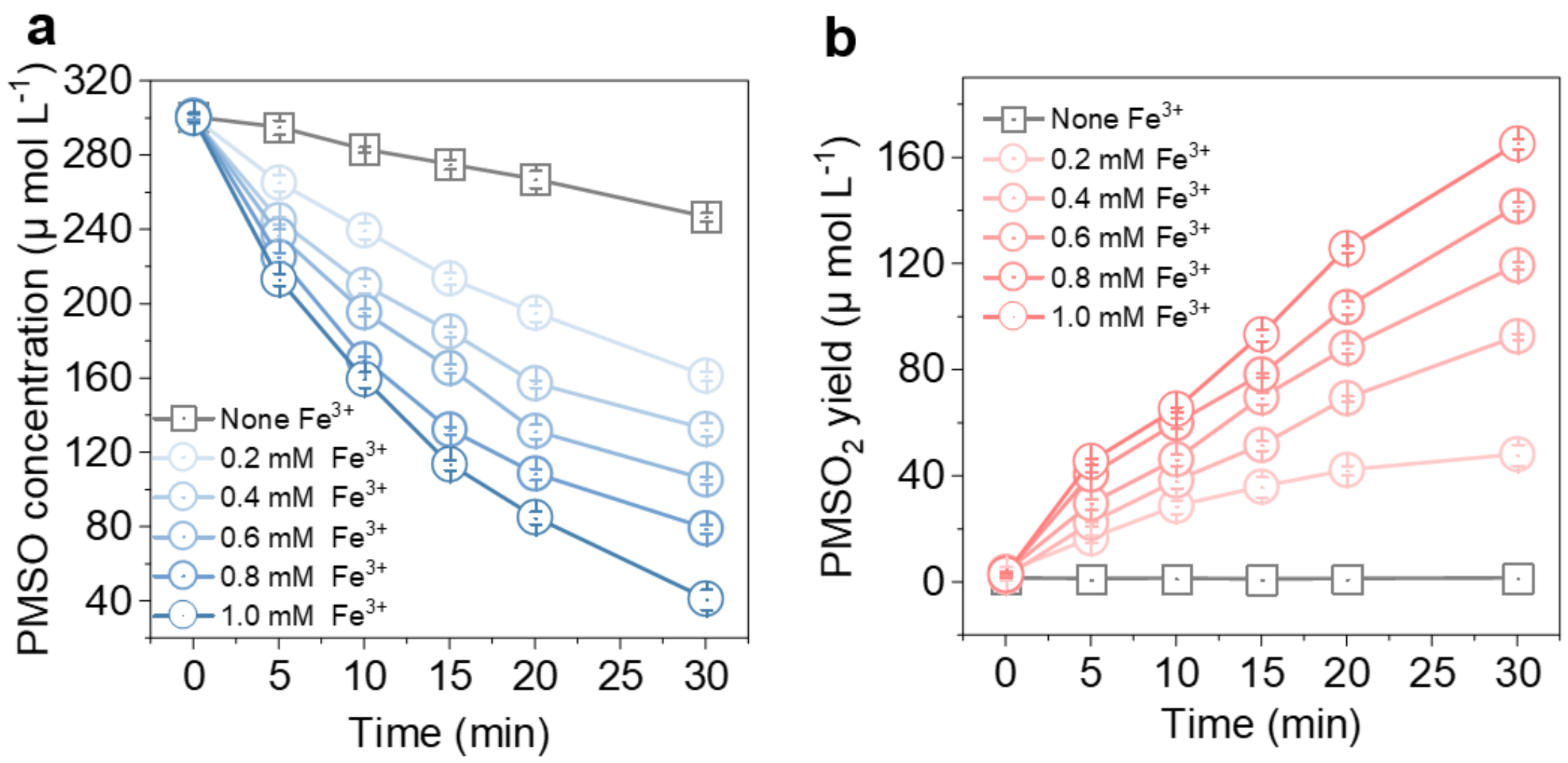

Supplementary Fig. 8. a. b. Effect of different Fe^{3+} concentrations on the consumption amount of PMSO and the generation amount of $PMSO_2$ in the BDD system. Error bars represent the standard deviation, obtained by repeating the experiment thrice (n = 3). Experimental conditions: [Na_2SO_4] = 50 mM, [PMSO] = 300 μM, current density = 7.5 mA cm^{-2}. Source data are provided as a Source Data file.

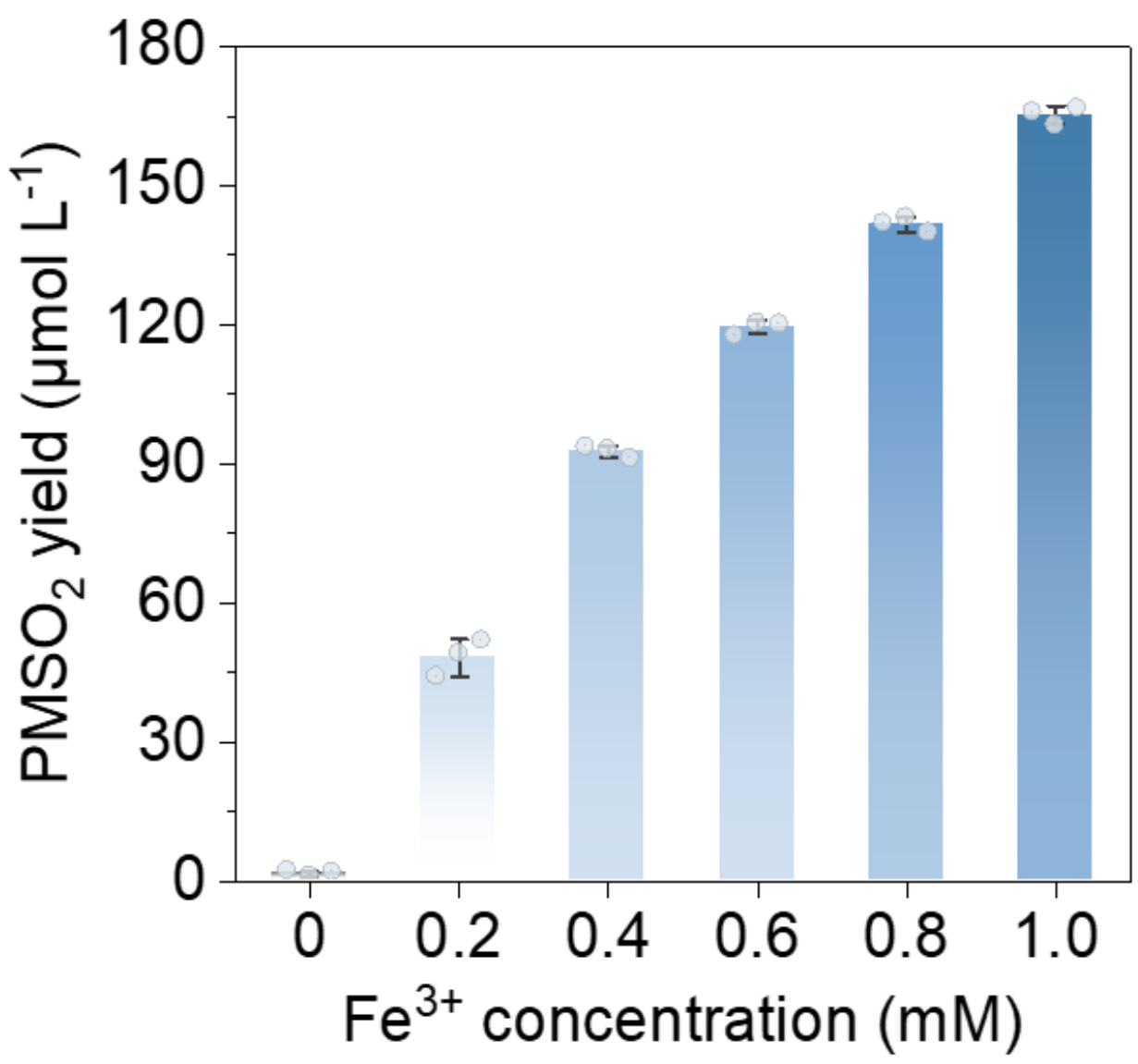

Supplementary Fig. 9. The amount of $PMSO_2$ generated corresponding to different Fe^{3+} concentrations. Error bars represent the standard deviation, obtained by repeating the experiment thrice ($n = 3$). Experimental conditions: $[Na_2SO_4]$ = 50 mM, [PMSO] = 300 μM, current density = 7.5 mA cm^{-2}. Source data are provided as a Source Data file.

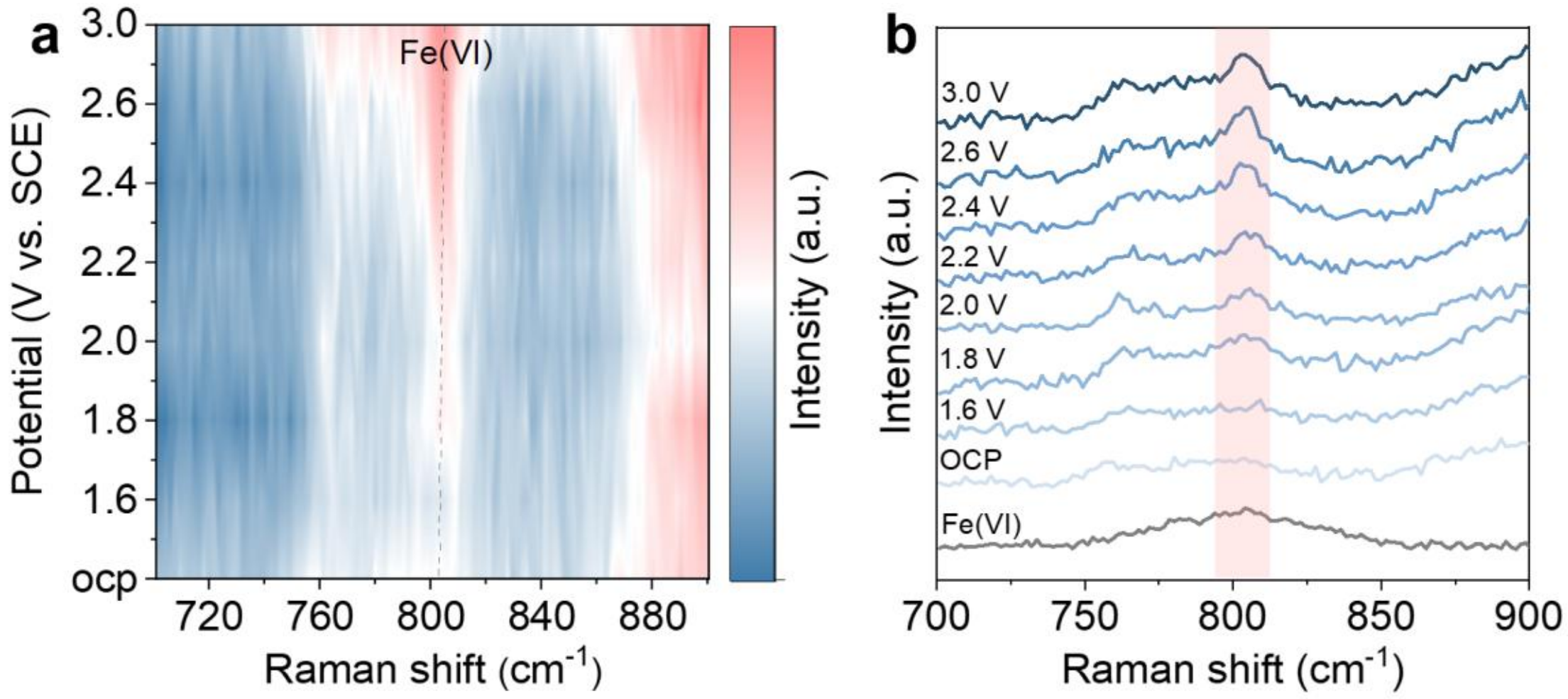

Supplementary Fig. 10. *In situ* electrochemical Raman spectroscopy of the electrode under applied potentials ranging from OCP to 3.0 V, acquired over the spectral window of 700–900 cm^{-1}. **a.** Contour heatmap showing the potential-dependent evolution of Raman scattering intensity as a function of wavenumber, with color gradient encoding normalized signal intensity. **b.** Representative line spectra extracted at selected potentials, illustrating the emergence, growth, and shift of characteristic vibrational bands with increasing anodic potential. Experimental conditions: [Na_2SO_4] = 50 mM, [Fe^{3+}] = 2 mM, [Fe(VI)] = 2 mM. Source data are provided as a Source Data file.

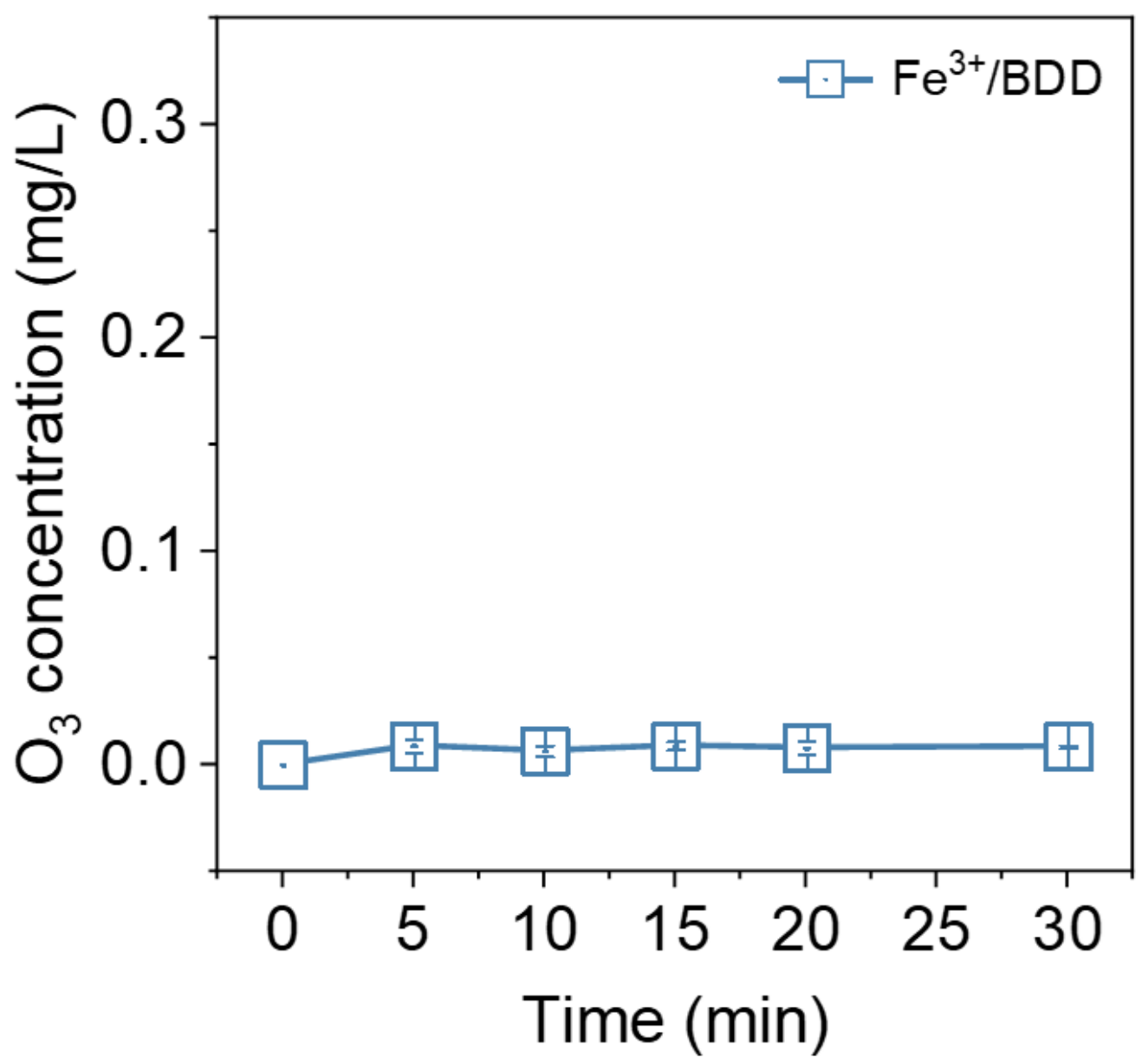

Supplementary Fig. 11. The variation of ozone generation concentration in the BDD system with reaction time was detected by the indigo method. Error bars represent the standard deviation, obtained by repeating the experiment thrice (n = 3). Experimental conditions: [Na_2SO_4] = 50 mM, current density = 7.5 mA cm^{-2}. Source data are provided as a Source Data file.

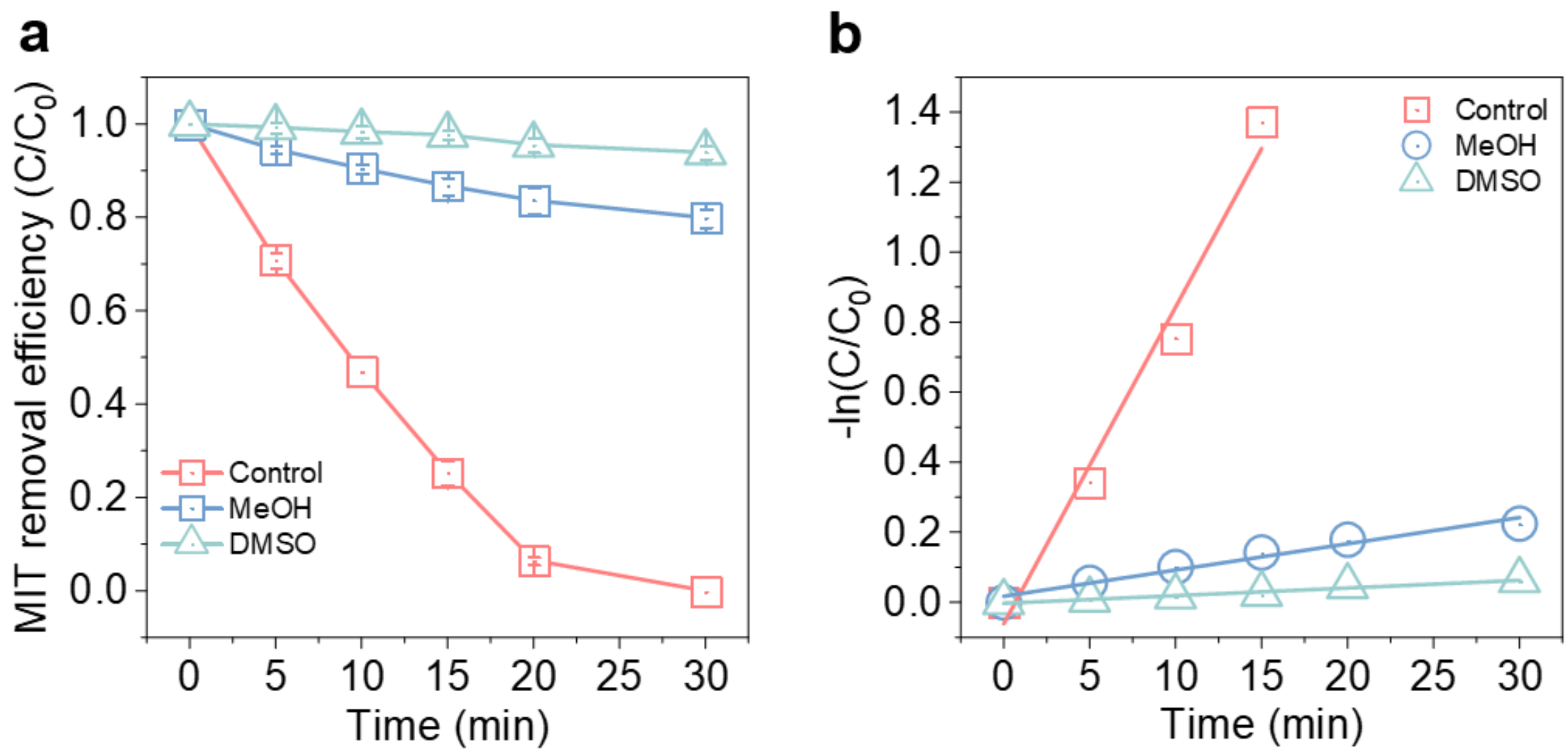

Supplementary Fig. 12. a. and b. Decay of MIT and corresponding first-order kinetic curves of MIT in quenching reaction processes in Fe^{3+}/BDD system. Error bars represent the standard deviation, obtained by repeating the experiment thrice (n = 3). Experimental conditions: [Na_2SO_4] = 50 mM, [MIT] = 20 μM, [Fe^{3+}] = 0.2 mM, [MeOH] = 500 mM, [DMSO] = 50 mM, current density = 7.5 mA cm^{-2}. Source data are provided as a Source Data file.

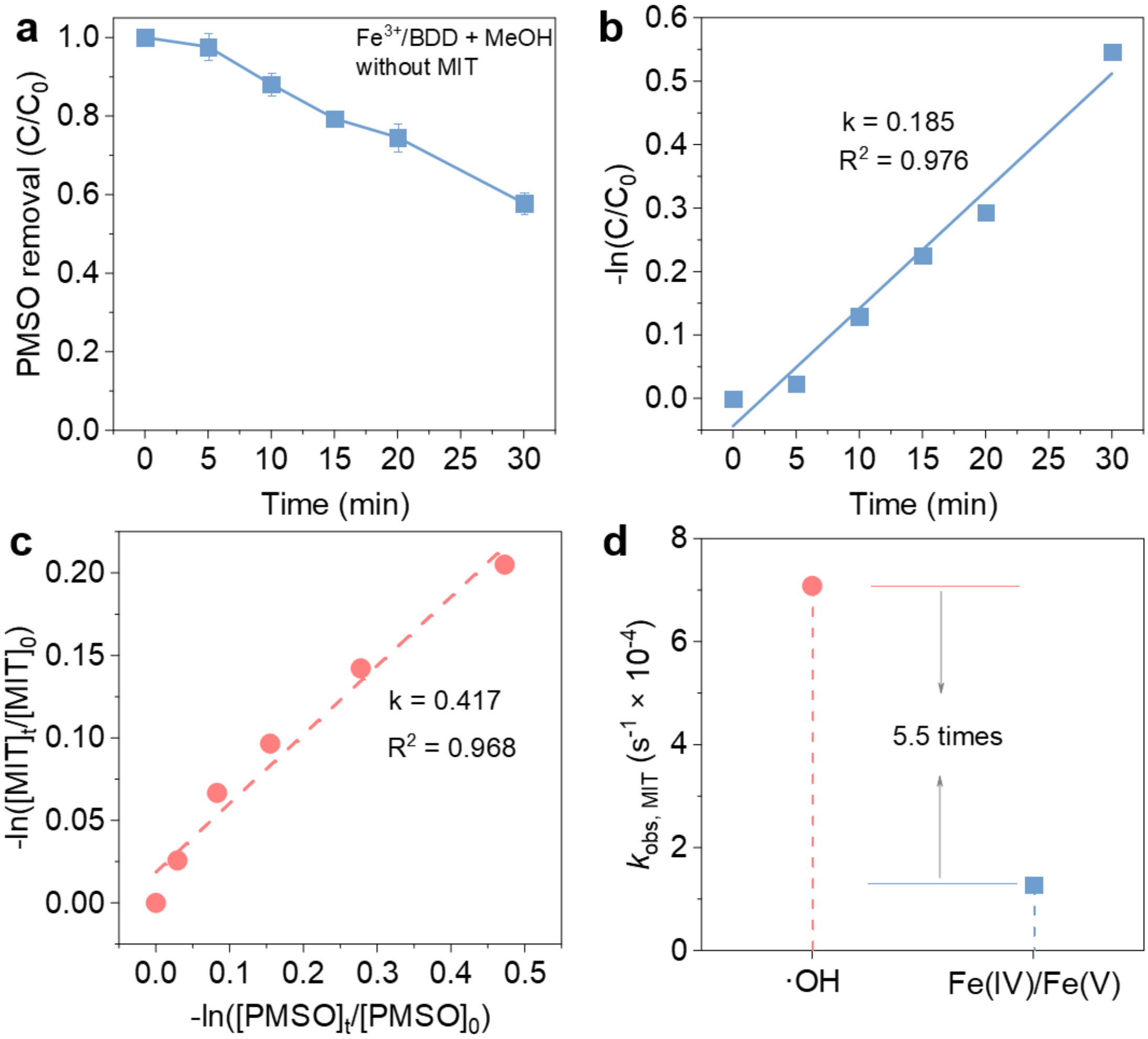

Supplementary Fig. 13. a. and b. Degradation of PMSO in the Fe^{3+}/BDD system during MeOH quenching and its corresponding first-order kinetic curves. **c.** Linear fitting of -ln([MIT]$_t$/[MIT]$_0$) and -ln([PMSO]$_t$/[PMSO]$_0$) obtained from the MeOH quenching process in the Fe^{3+}/BDD system with MIT and PMSO at the same concentration. **d.** The $k_{obs,MIT}$ of with •OH and Fe(IV)/Fe(V) in the Fe^{3+}/BDD system. Error bars represent the standard deviation, obtained by repeating the experiment thrice (n = 3). Experimental conditions: [Na_2SO_4] = 50 mM, [MIT] = [PMSO] =20 μM, [Fe^{3+}] = 0.2 mM, [MeOH] = 1000 mM, current density = 7.5 mA cm^{-2}. Source data are provided as a Source Data file.

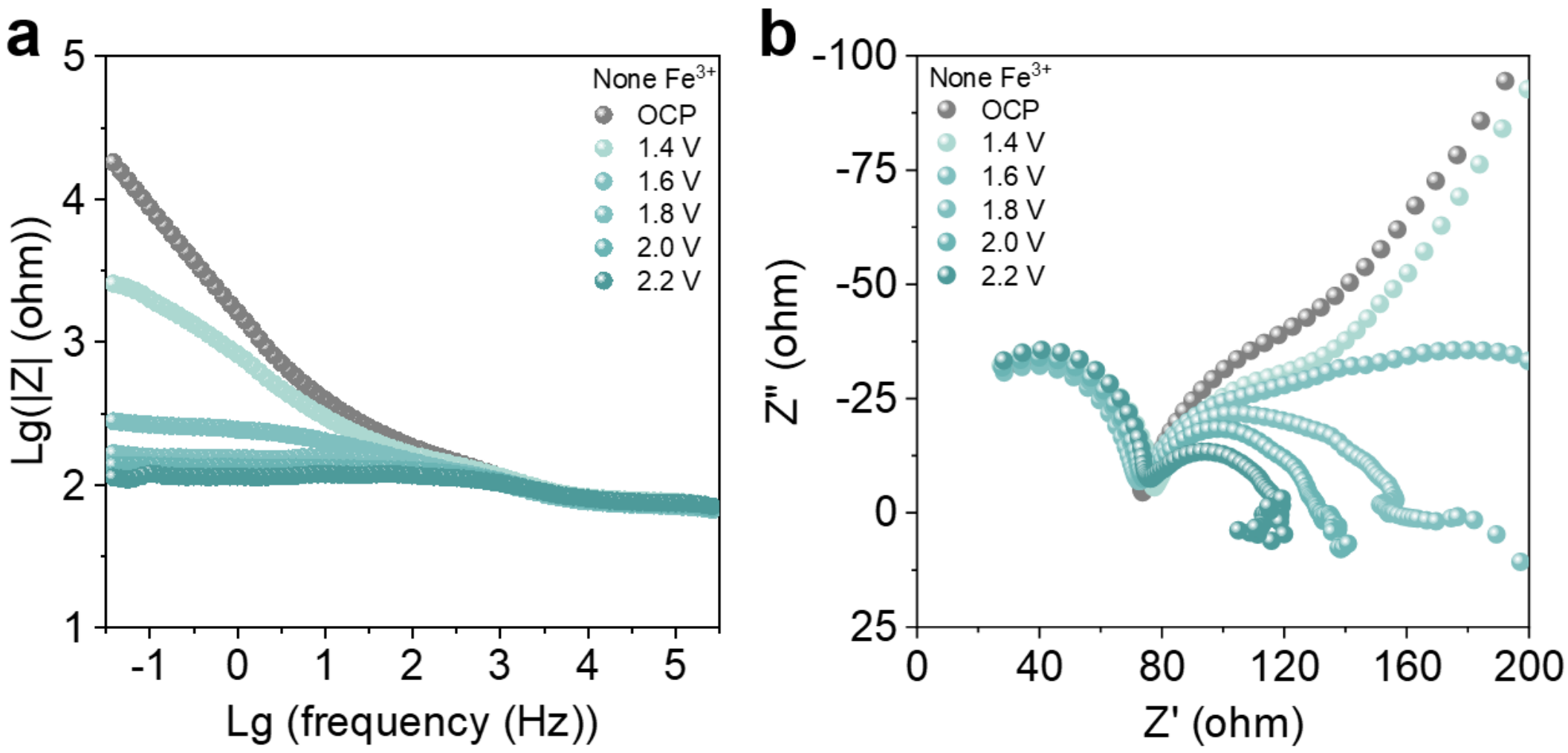

Supplementary Fig. 14. a and b. Bode magnitude plots and Nyquist plots at different potentials. Experimental conditions: $[Na_2SO_4]$ = 50 mM, without Fe^{3+}. Source data are provided as a Source Data file.

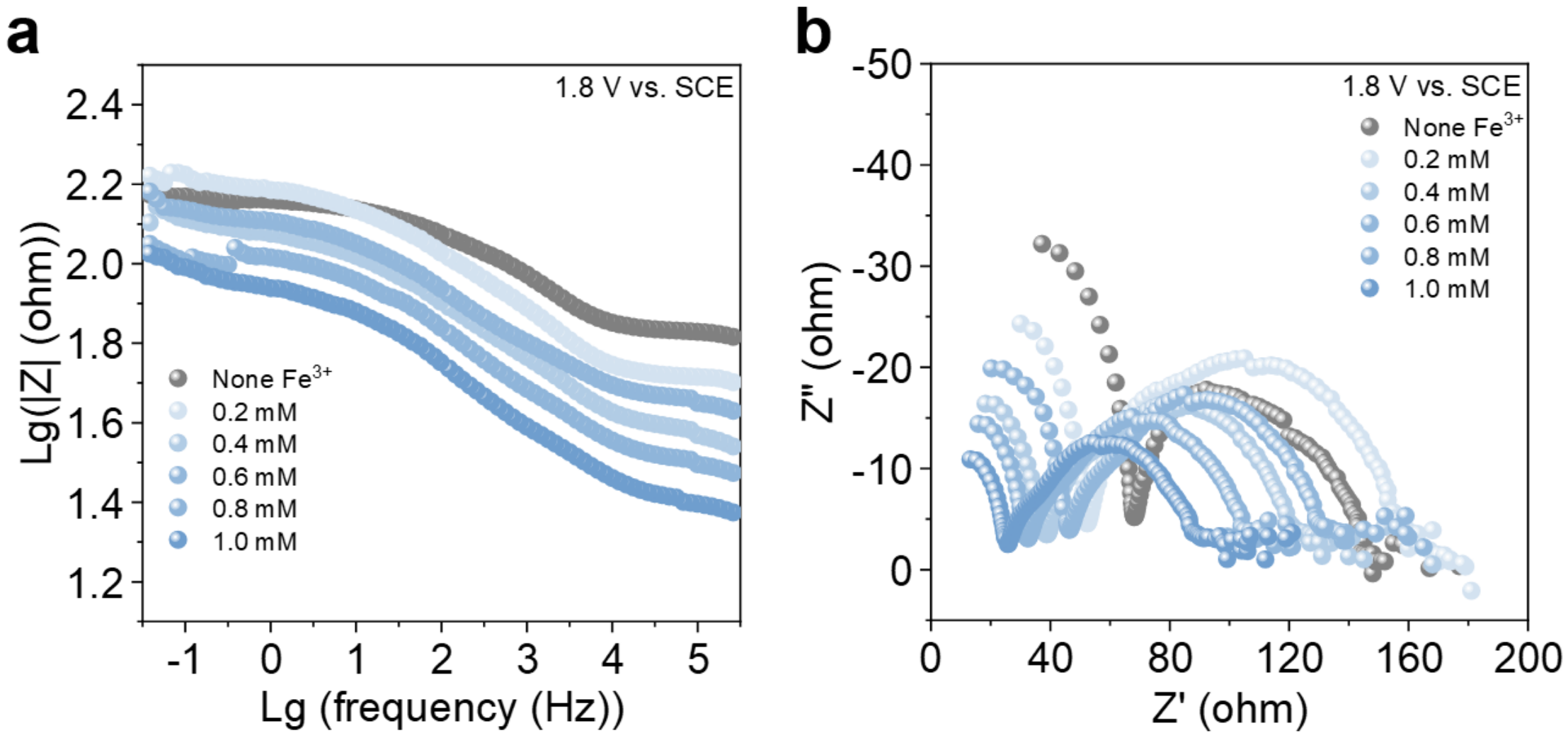

Supplementary Fig. 15. a and b. Bode magnitude plots and Nyquist plots at different Fe^{3+} concentrations. Experimental conditions: [Na_2SO_4] = 50 mM, potential = 1.8 V vs. SCE. Source data are provided as a Source Data file.

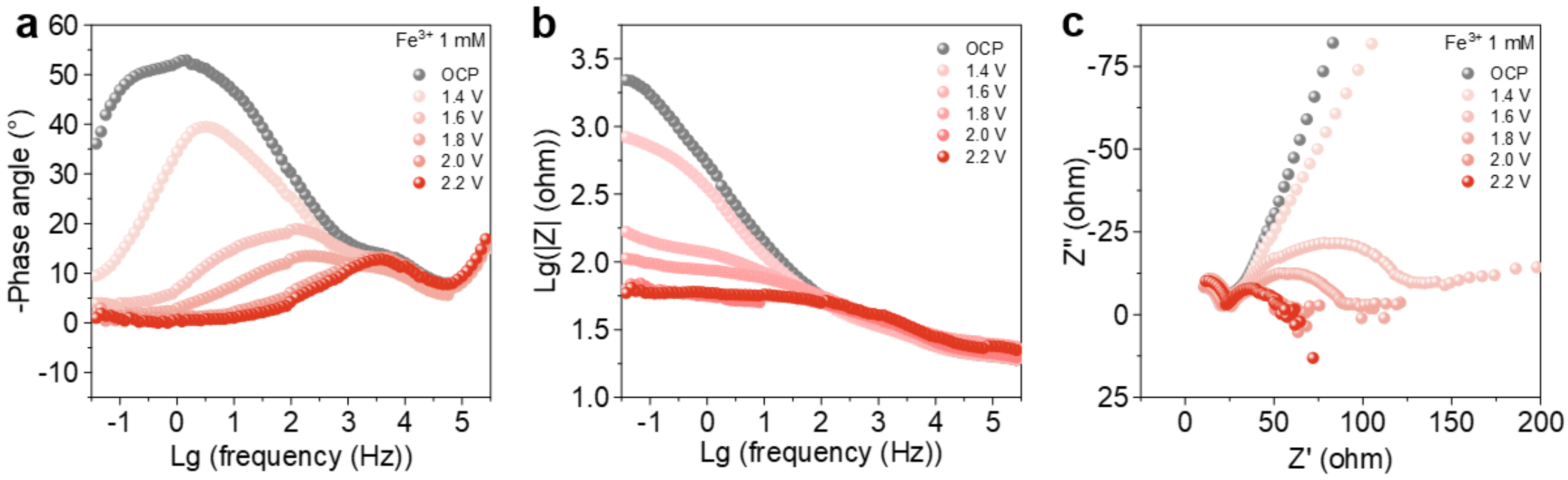

Supplementary Fig. 16. a. b. and c. Impedance Bode phase angle plots, Bode magnitude plots and Nyquist plots at different potentials. Experimental conditions: [Na_2SO_4] = 50 mM, [Fe^{3+}] = 1 mM. Source data are provided as a Source Data file.

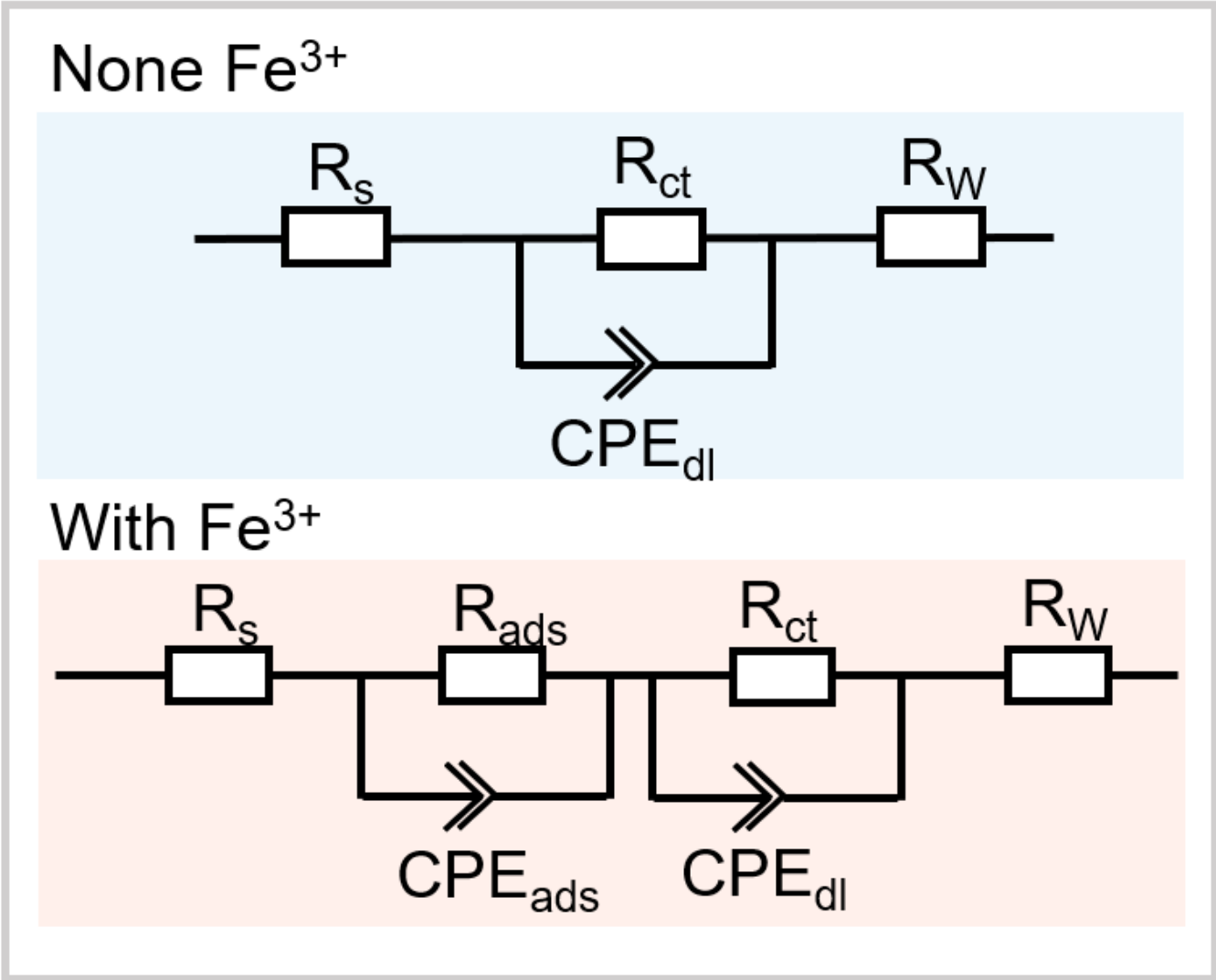

Supplementary Fig. 17. Schematic diagram of the equivalent circuit without Fe^{3+} and with Fe^{3+}. (According to the EIS test results, the equivalent circuit simulated by using ZView2 software.)

Note: The EIS test results were fitted using the Zview2 software, and an equivalent circuit model was obtained. With Fe^{3+} and without Fe^{3+} are two different types of equivalent circuit models.

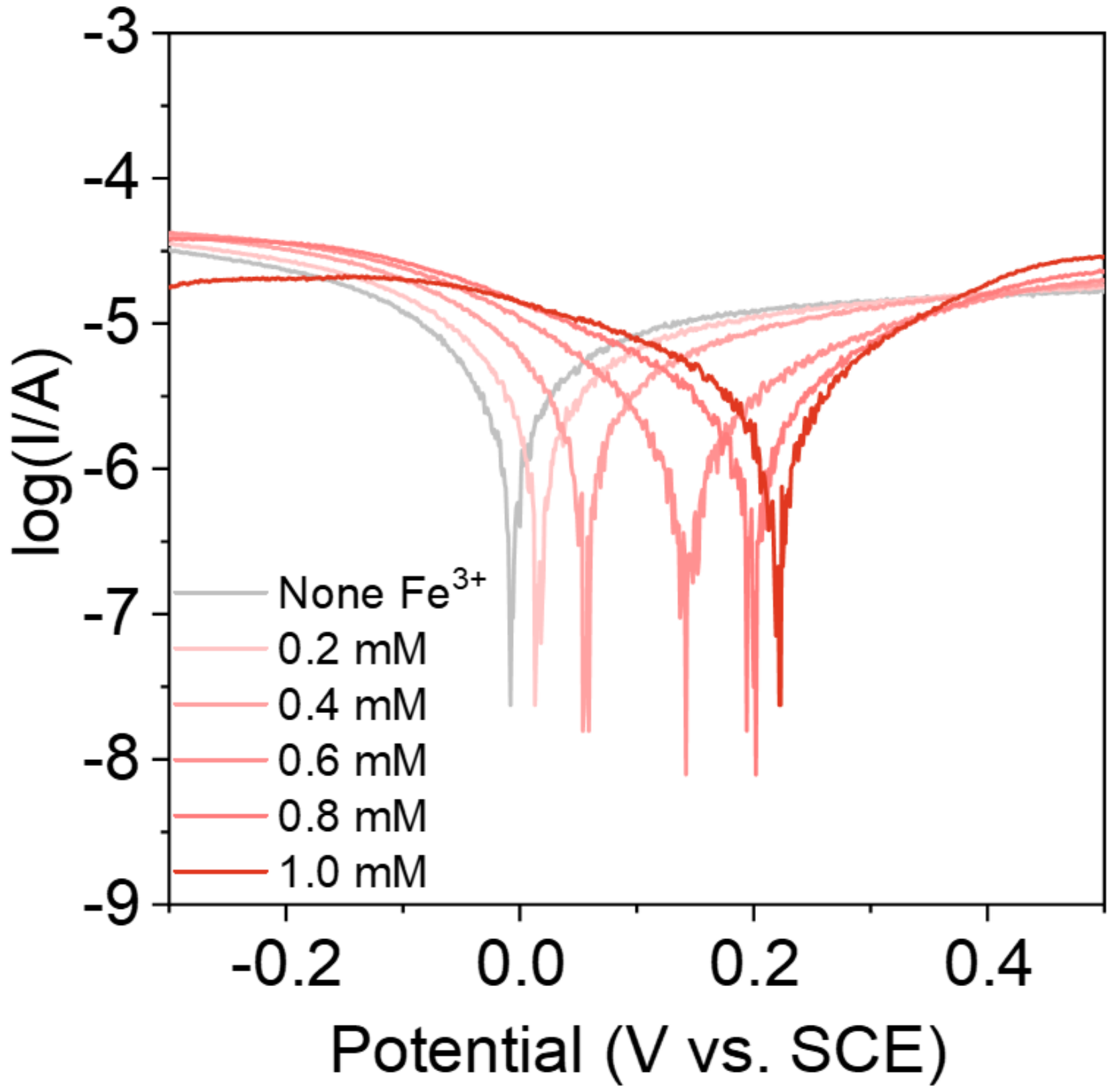

Supplementary Fig. 18. Tafel curves under different iron ion concentrations. Experimental conditions: [Na_2SO_4] = 50 mM. Source data are provided as a Source Data file.

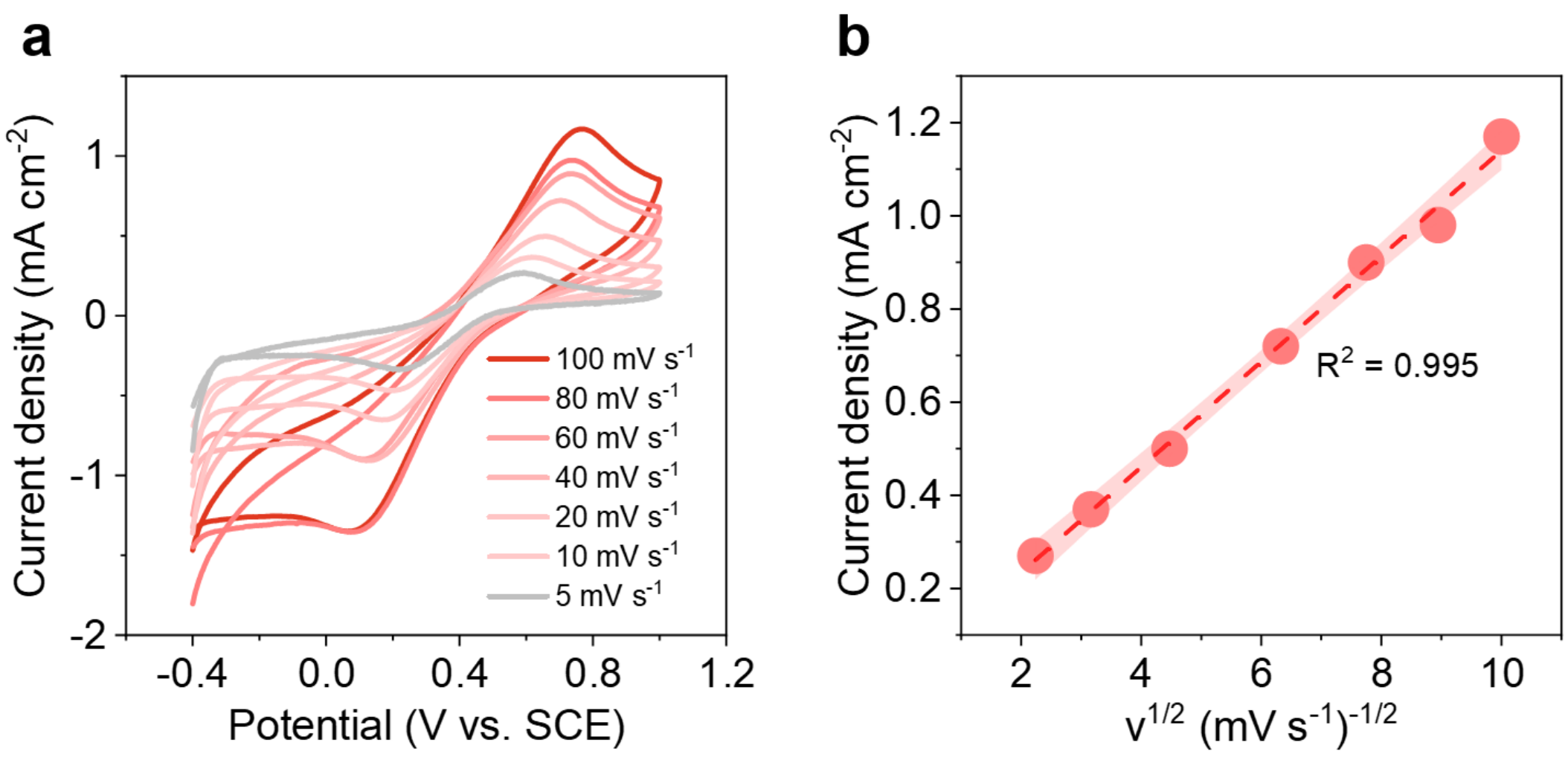

Supplementary Fig. 19. a. Cyclic voltammogram (CV) curves at different scanning rates. **b.** Fitting of the square root of the scanning rate to the current density. Experimental conditions: [Na_2SO_4] = 50 mM, [Fe^{3+}] = 1 mM, current density = 7.5 mA cm^{-2}. Source data are provided as a Source Data file.

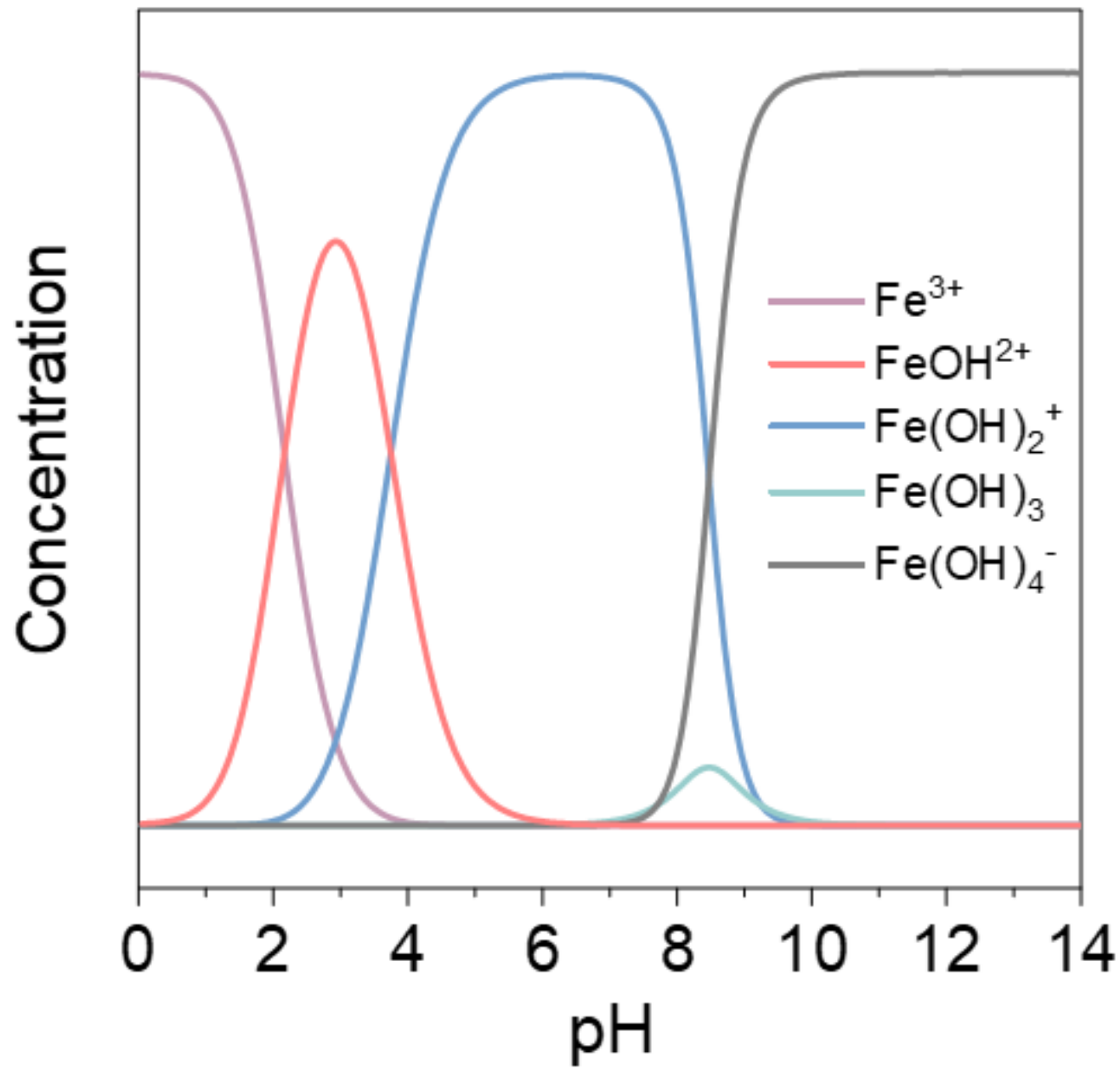

Supplementary Fig. 20. Intricate distribution dynamics of Fe^{3+} across a spectrum of pH levels. Source data are provided as a Source Data file.

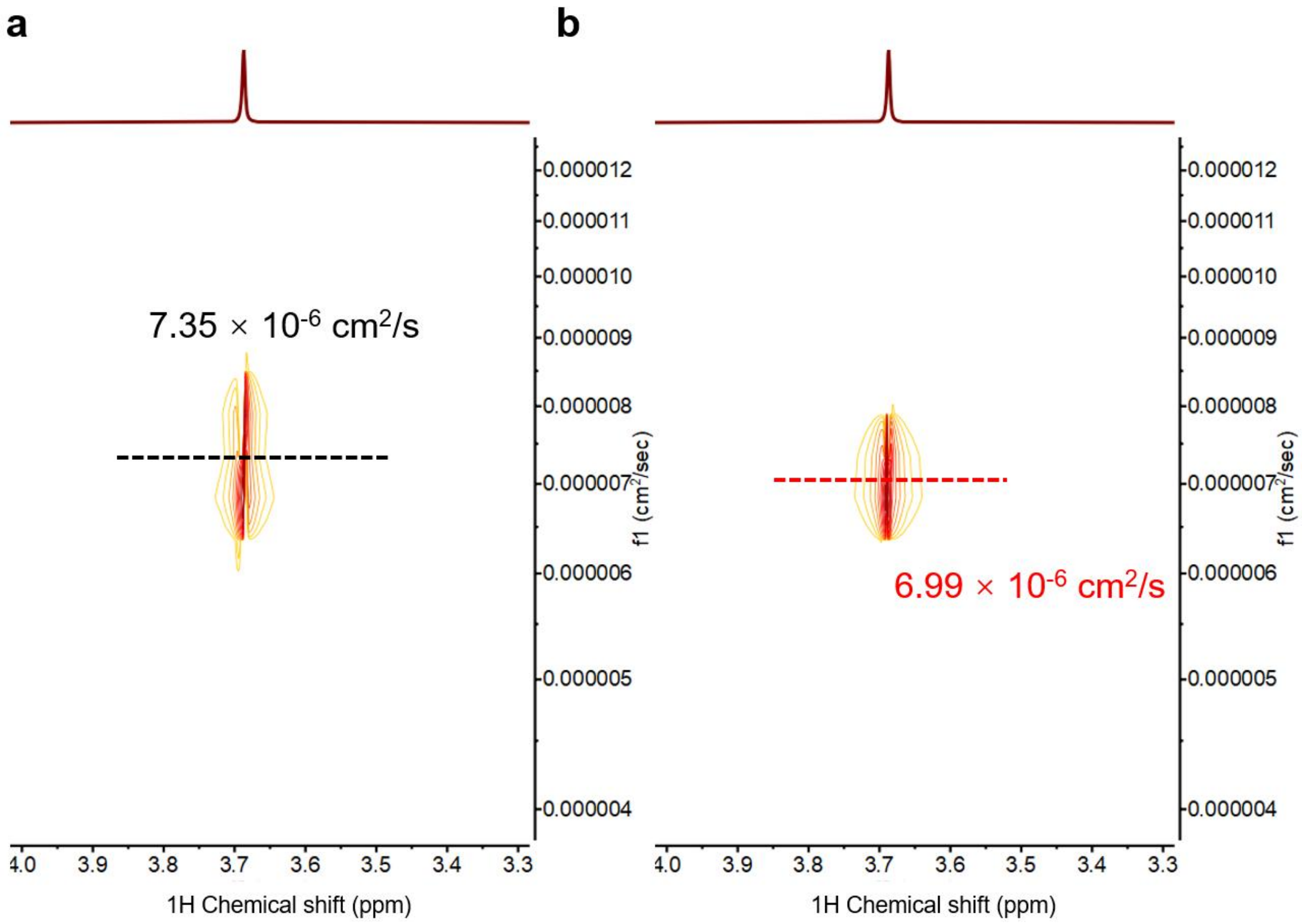

Supplementary Fig. 21. a. and b. 2D DOSY NMR of H_2O/DMSO solutions with and without Fe^{3+} addition.

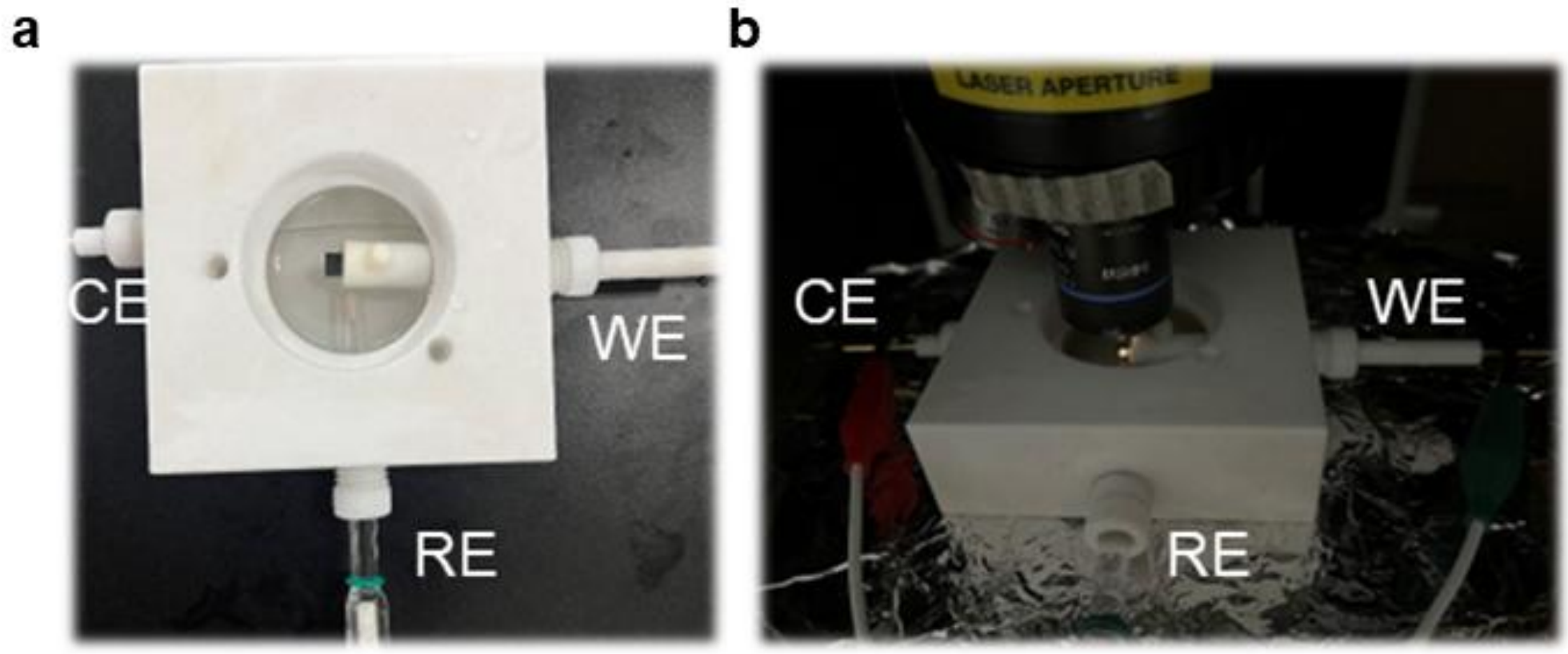

Supplementary Fig. 22. *In situ* Raman spectroscopy test image. **a.** Photograph of the custom-built electrochemical reaction cell used for in-situ Raman measurements. **b.** Photograph showing the real-world experimental testing setup during data acquisition. CE: counter electrode; WE: work electrode; RE: reference electrode.

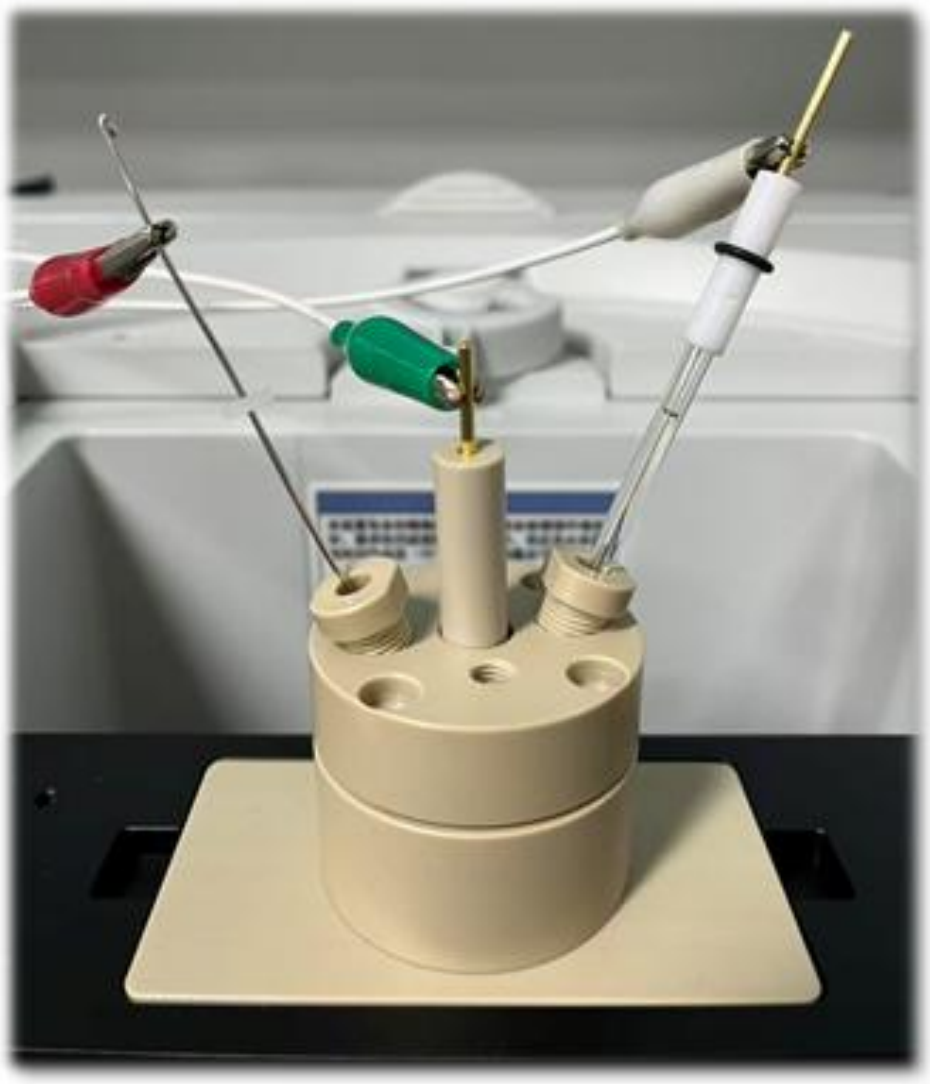

Supplementary Fig. 23. *In situ* FTIR spectroscopy test Image.

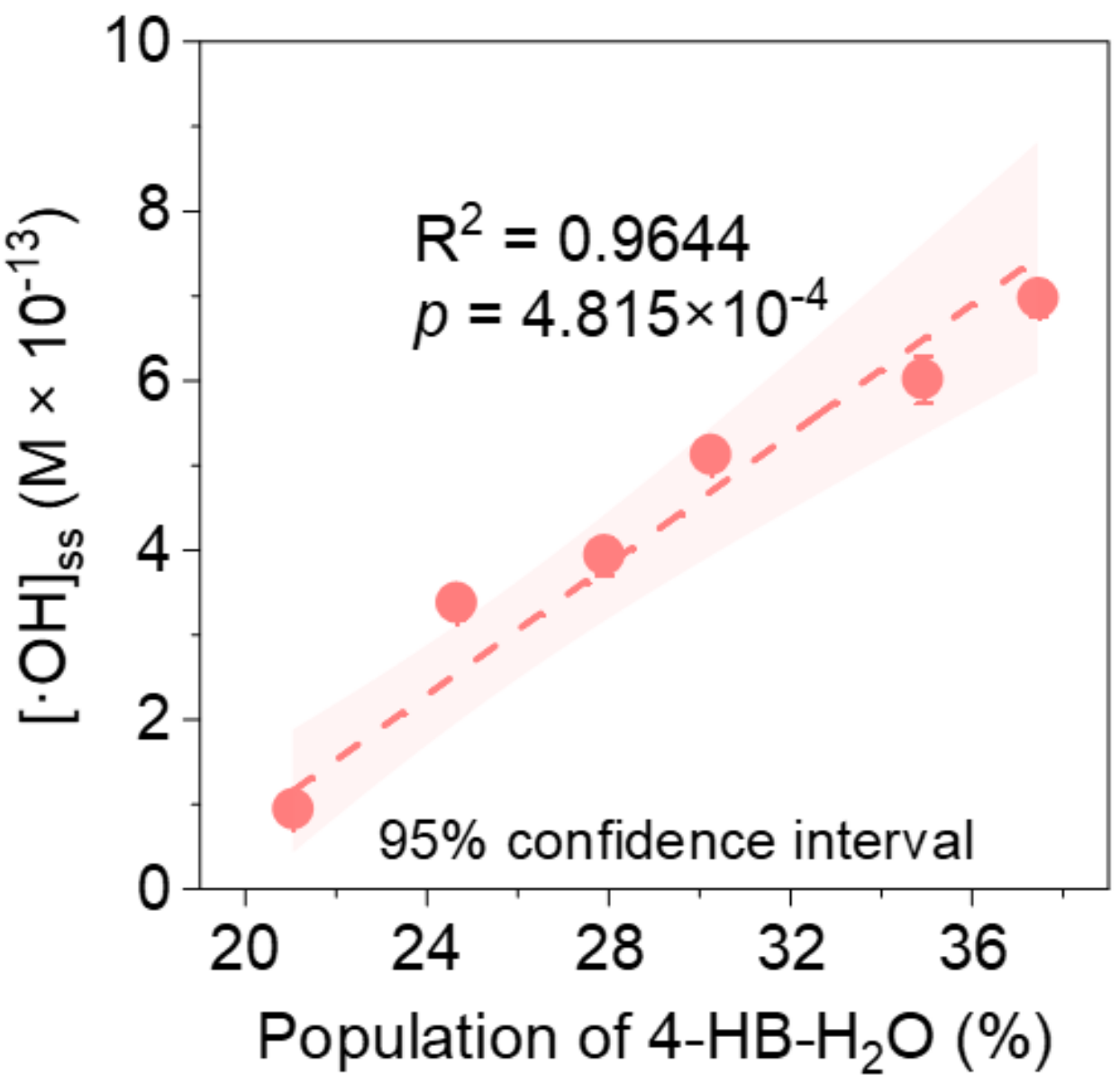

Supplementary Fig. 24. Linear fitting (95% confidence interval) between the proportion of 4-HB-H_2O and the concentration of •OH. Source data are provided as a Source Data file.

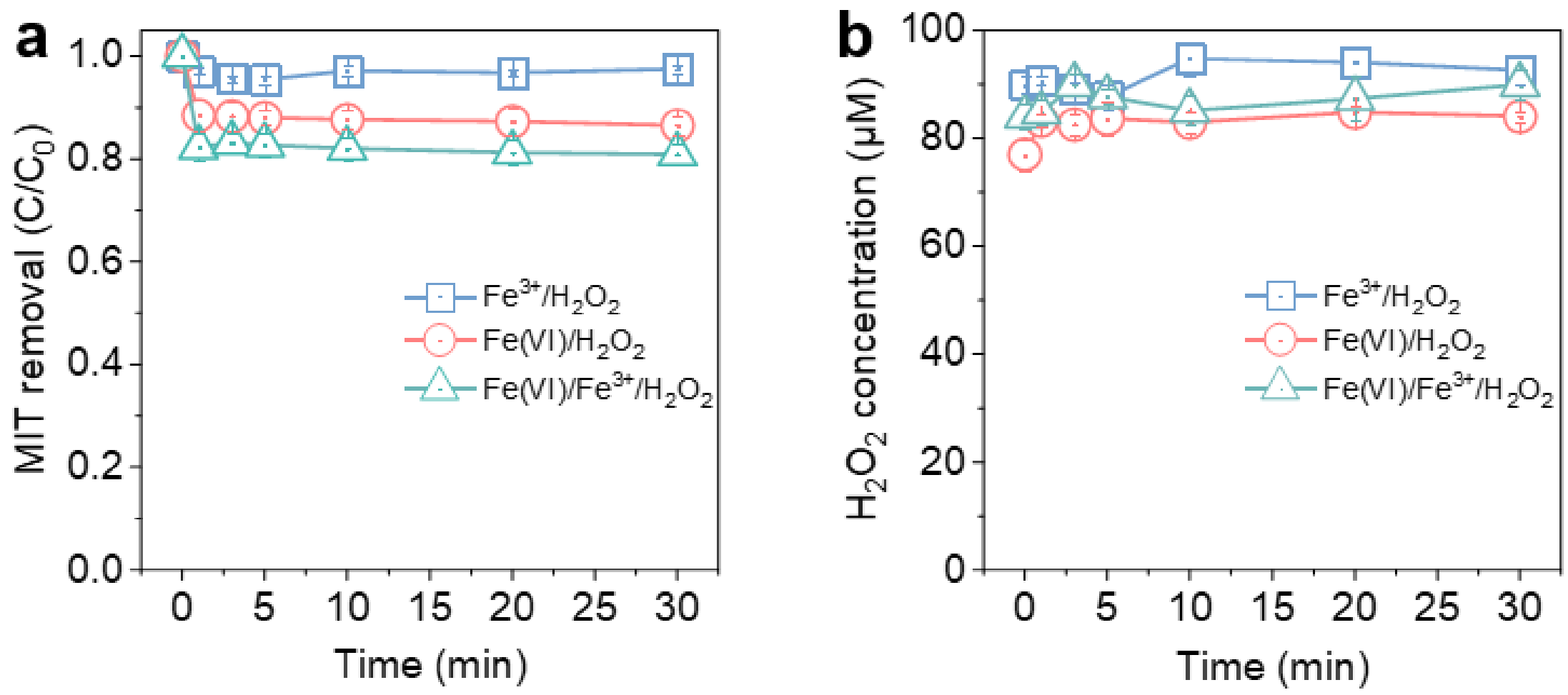

Supplementary Fig. 25. a. The removal effect of MIT by Fe^{3+}/H_2O_2, $Fe(VI)/H_2O_2$ and $Fe(VI)/Fe^{3+}/H_2O_2$, **b.** the variation of H_2O_2 concentration with time in Fe^{3+}/H_2O_2, $Fe(VI)/H_2O_2$ and $Fe(VI)/Fe^{3+}/H_2O_2$ systems. Error bars represent the standard deviation, obtained by repeating the experiment thrice (n = 3). Experimental conditions: [MIT] = 20 μM, $[Fe^{3+}]$ = 0.2 mM, [Fe(VI)] = 2 mg/L, $[H_2O_2]$ = 80 μM, initial pH = 3. Source data are provided as a Source Data file.

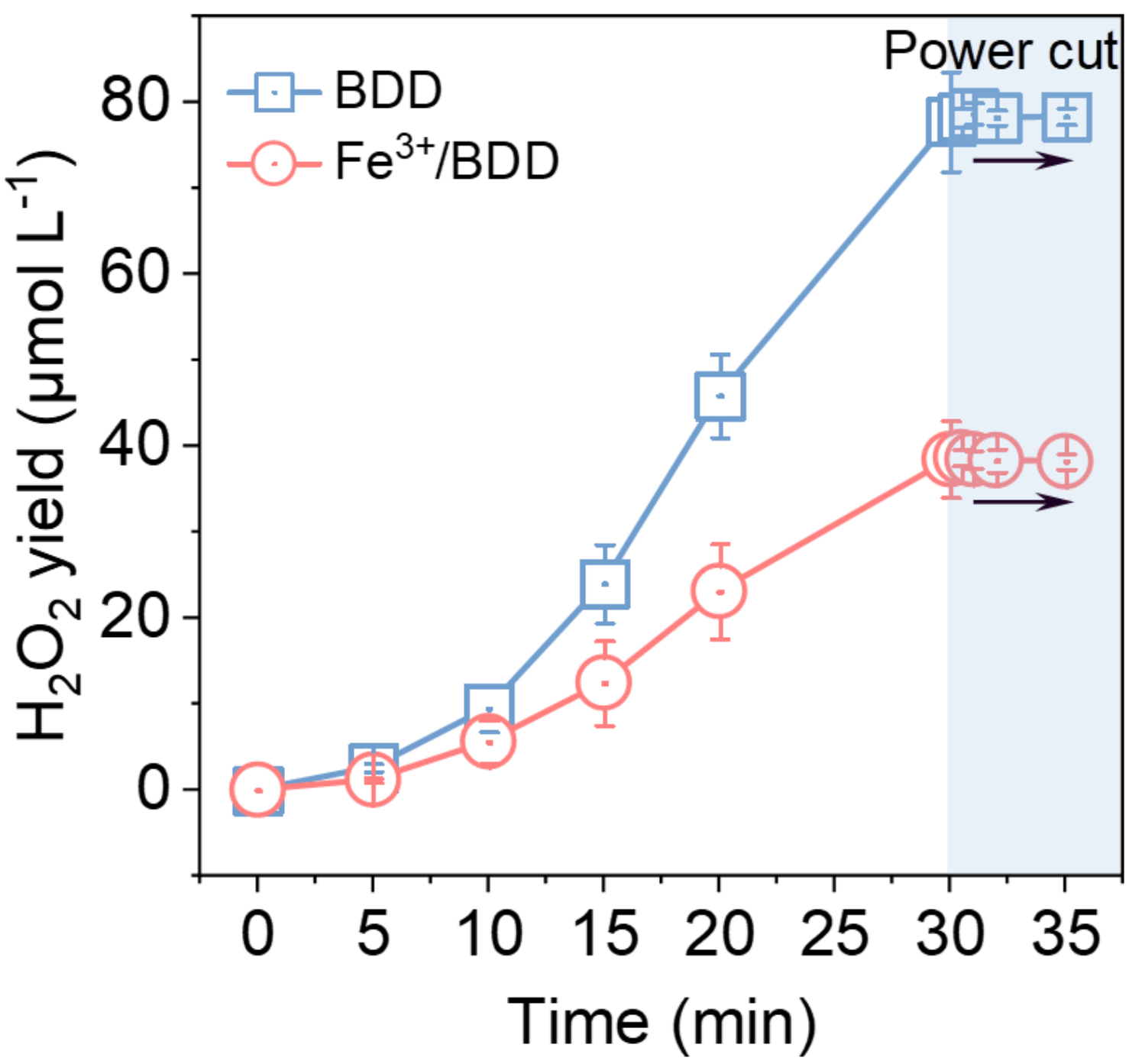

Supplementary Fig. 26. The continuous removal and the changes in H_2O_2 concentration after power cut. Error bars represent the standard deviation, obtained by repeating the experiment thrice (n = 3). Experimental conditions: [Na_2SO_4] = 50 mM, [Fe^{3+}] = 0.2 mM, current density = 7.5 mA cm^{-2}. Source data are provided as a Source Data file.

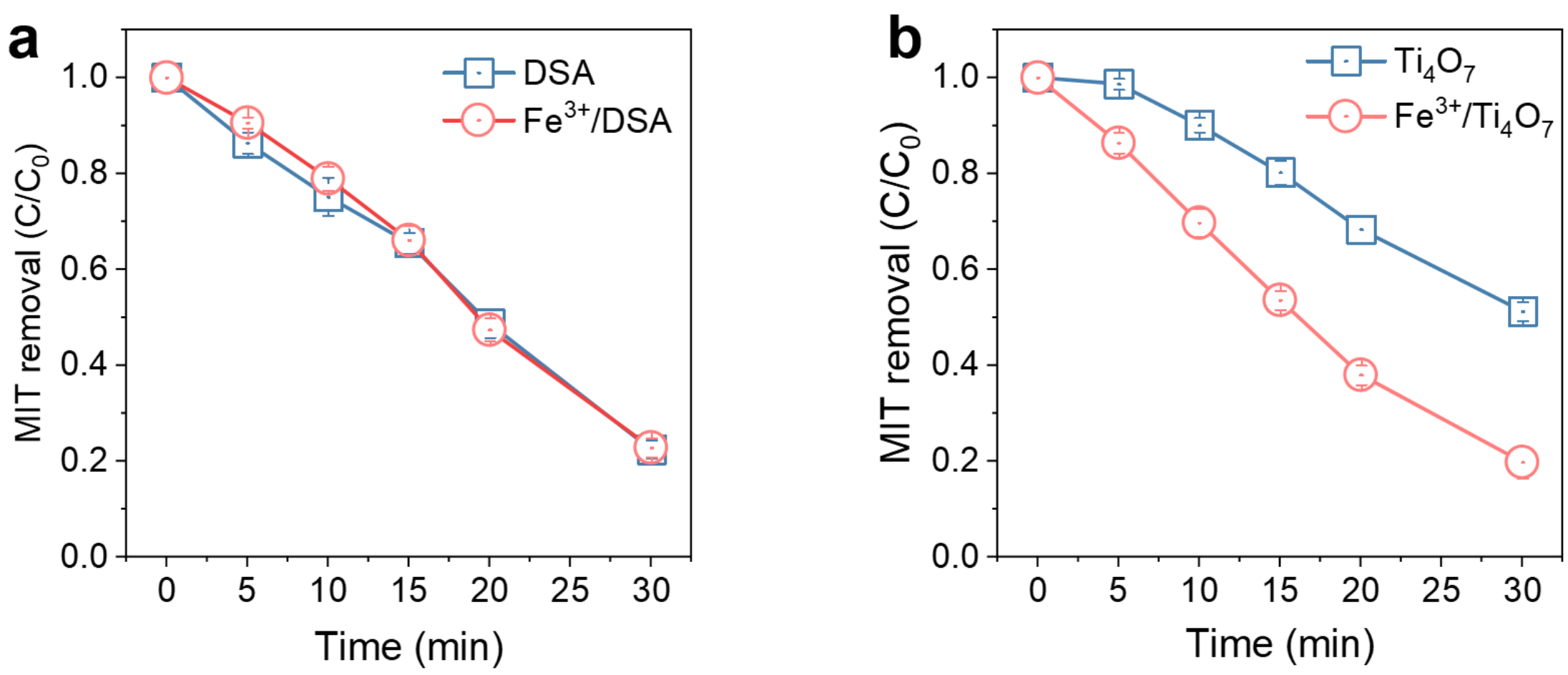

Supplementary Fig. 27. a and b. The removal efficiency of MIT in the DSA, Fe^{3+}/DSA, Ti_4O_7 and Fe^{3+}/Ti_4O_7 systems. Error bars represent the standard deviation, obtained by repeating the experiment thrice (n = 3). Experimental conditions: [Na_2SO_4] = 50 mM, [Fe^{3+}] = 0.2 mM, current density = 7.5 mA cm^{-2}. Source data are provided as a Source Data file.

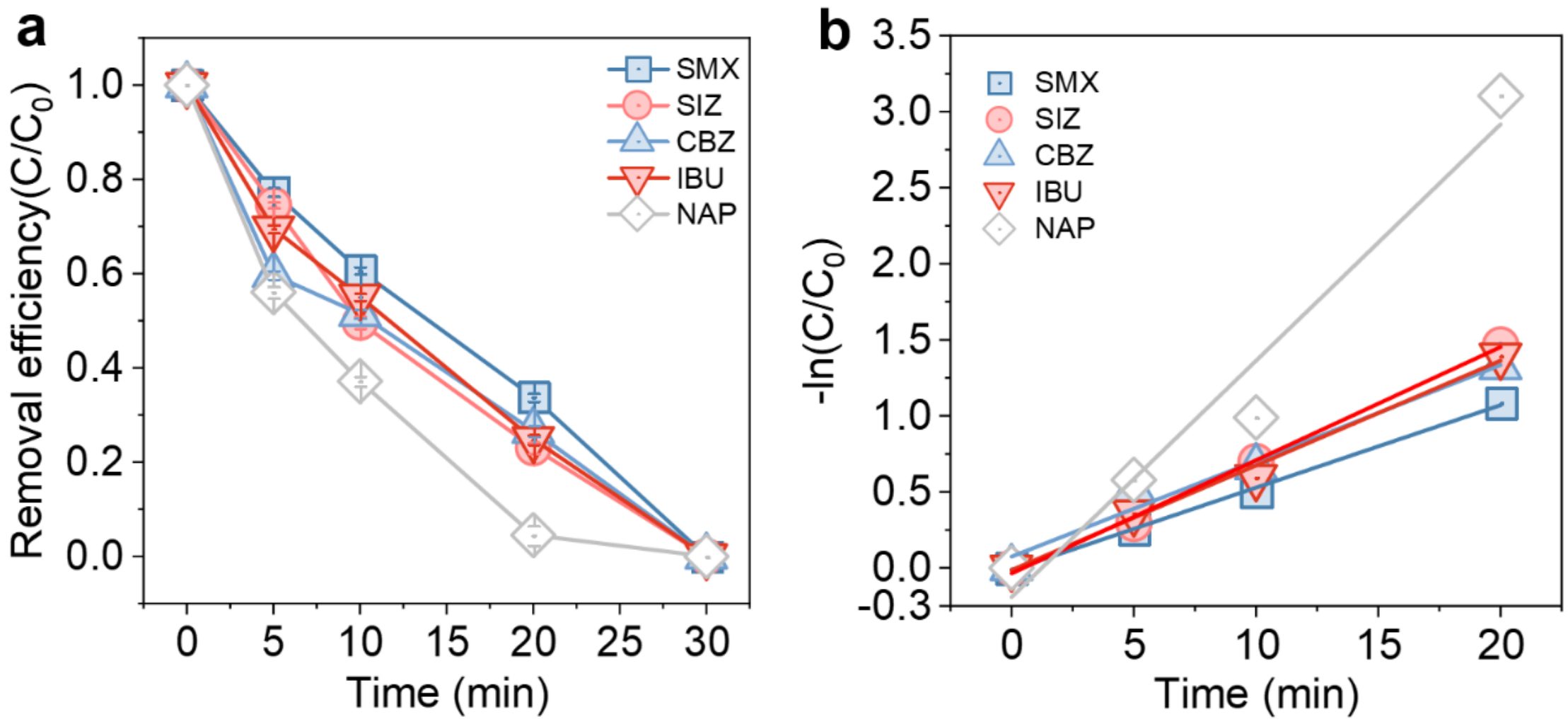

Supplementary Fig. 28. a. The attenuation of SMX, SIZ, CBZ, IBU and NAP **b.** and the corresponding first-order kinetic curves in the Fe^{3+}/BDD system. Error bars represent the standard deviation, obtained by repeating the experiment thrice (n = 3). Experimental conditions: current density = 7.5 mA cm^{-2}, [Na_2SO_4] = 50 mM, [pollution] = 20 μM, [Fe^{3+}] = 0.2 mM. Source data are provided as a Source Data file.

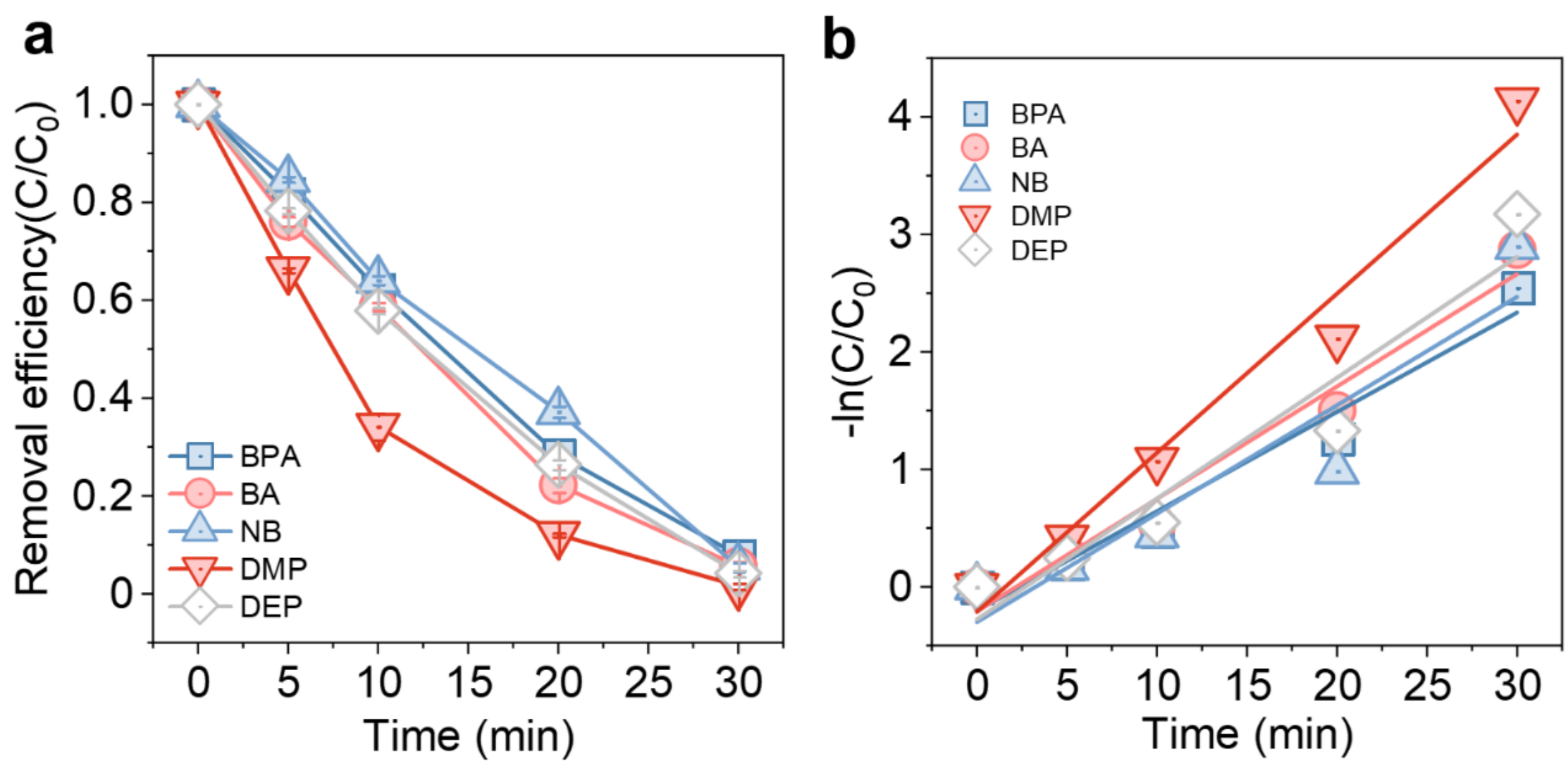

Supplementary Fig. 29. a. The attenuation of BPA, BA, NB, DMP and DEP **b.** and the corresponding first-order kinetic curves in the Fe^{3+}/BDD system. Error bars represent the standard deviation, obtained by repeating the experiment thrice (n = 3). Experimental conditions: current density = 7.5 mA cm^{-2}, [Na_2SO_4] = 50 mM, [pollution] = 20 μM, [Fe^{3+}] = 0.2 mM. Source data are provided as a Source Data file.

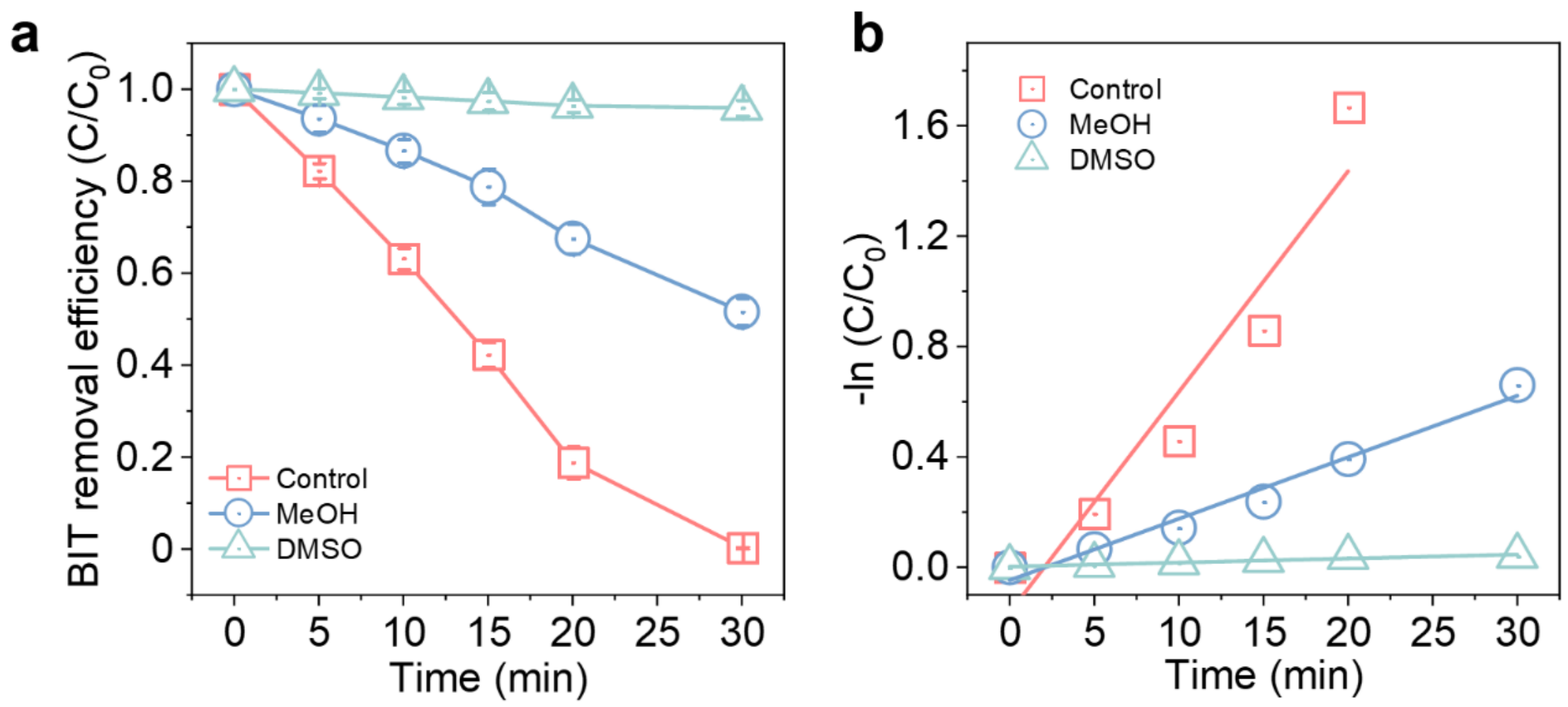

Supplementary Fig. 30. a. and b. Decay of BIT and corresponding first-order kinetic curves of BIT in quenching reaction processes in Fe^{3+}/BDD system. Error bars represent the standard deviation, obtained by repeating the experiment thrice (n = 3). Experimental conditions: [Na_2SO_4] = 50 mM, [BIT] = 20 μM, [Fe^{3+}] = 0.2 mM, [MeOH] = 200 mM, [DMSO] = 50 mM, current density = 7.5 mA cm^{-2}. Source data are provided as a Source Data file.

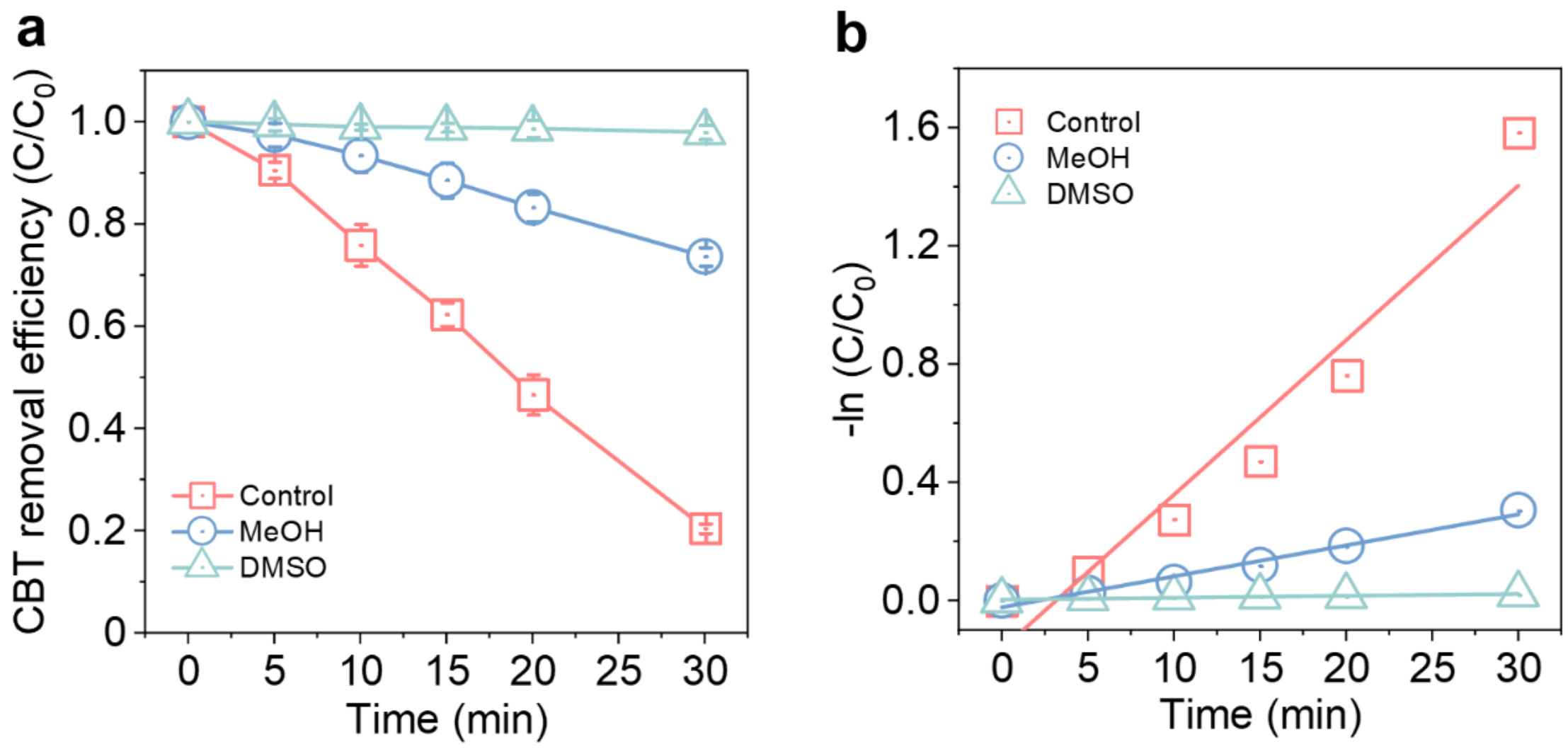

Supplementary Fig. 31. a. and b. Decay of CBT and corresponding first-order kinetic curves of CBT in quenching reaction processes in Fe^{3+}/BDD system. Error bars represent the standard deviation, obtained by repeating the experiment thrice (n = 3). Experimental conditions: [Na_2SO_4] = 50 mM, [CBT] = 20 μM, [Fe^{3+}] = 0.2 mM, [MeOH] = 200 mM, [DMSO] = 50 mM, current density = 7.5 mA cm^{-2}. Source data are provided as a Source Data file.

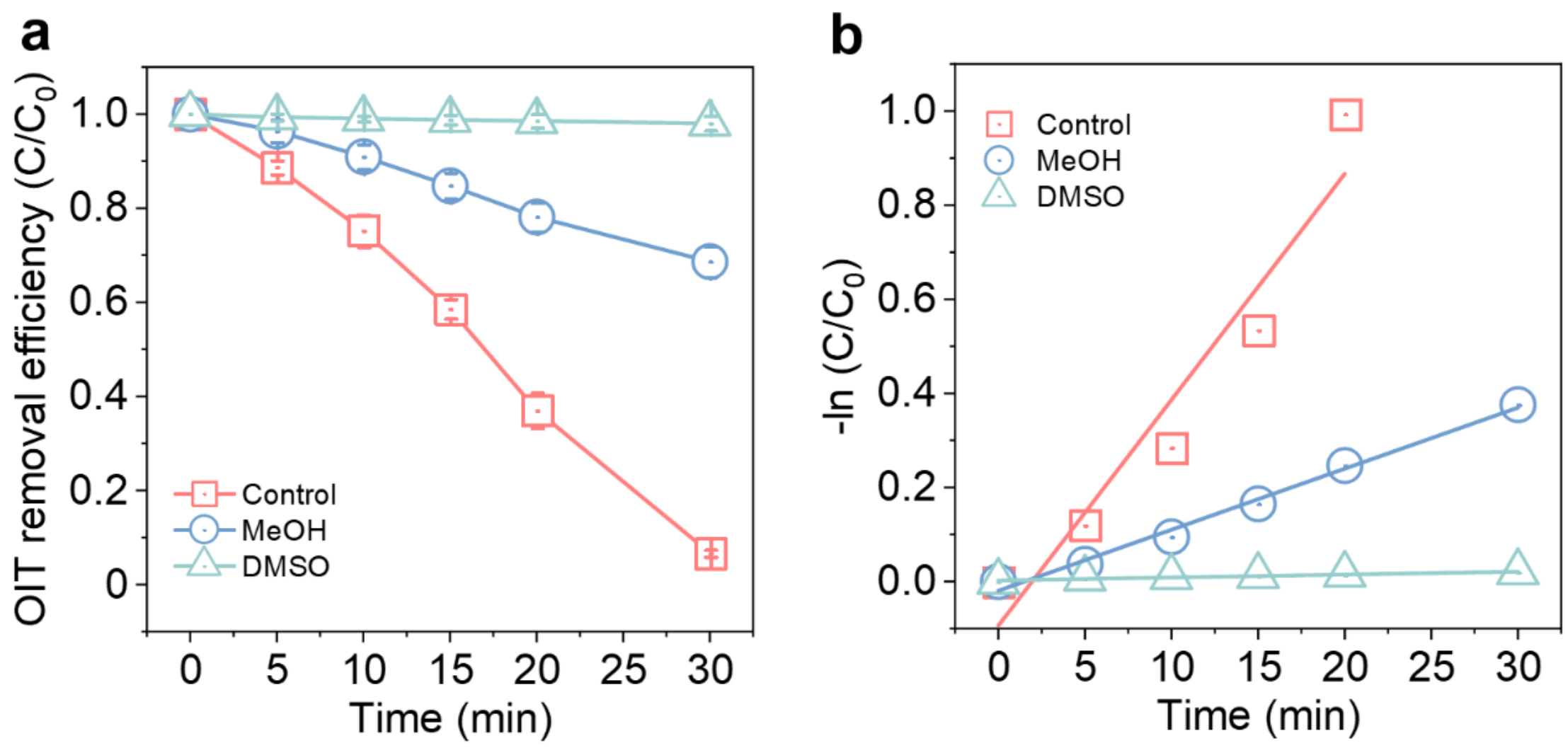

Supplementary Fig. 32. a. and b. Decay of OIT and corresponding first-order kinetic curves of OIT in quenching reaction processes in Fe^{3+}/BDD system. (Note: The -ln(C/C_0) value at 30 minutes in the control group was too high, making it unsuitable for kinetic fitting.) Error bars represent the standard deviation, obtained by repeating the experiment thrice (n = 3). Experimental conditions: [Na_2SO_4] = 50 mM, [OIT] = 20 μM, [Fe^{3+}] = 0.2 mM, [MeOH] = 200 mM, [DMSO] = 50 mM, current density = 7.5 mA cm^{-2}. Source data are provided as a Source Data file.

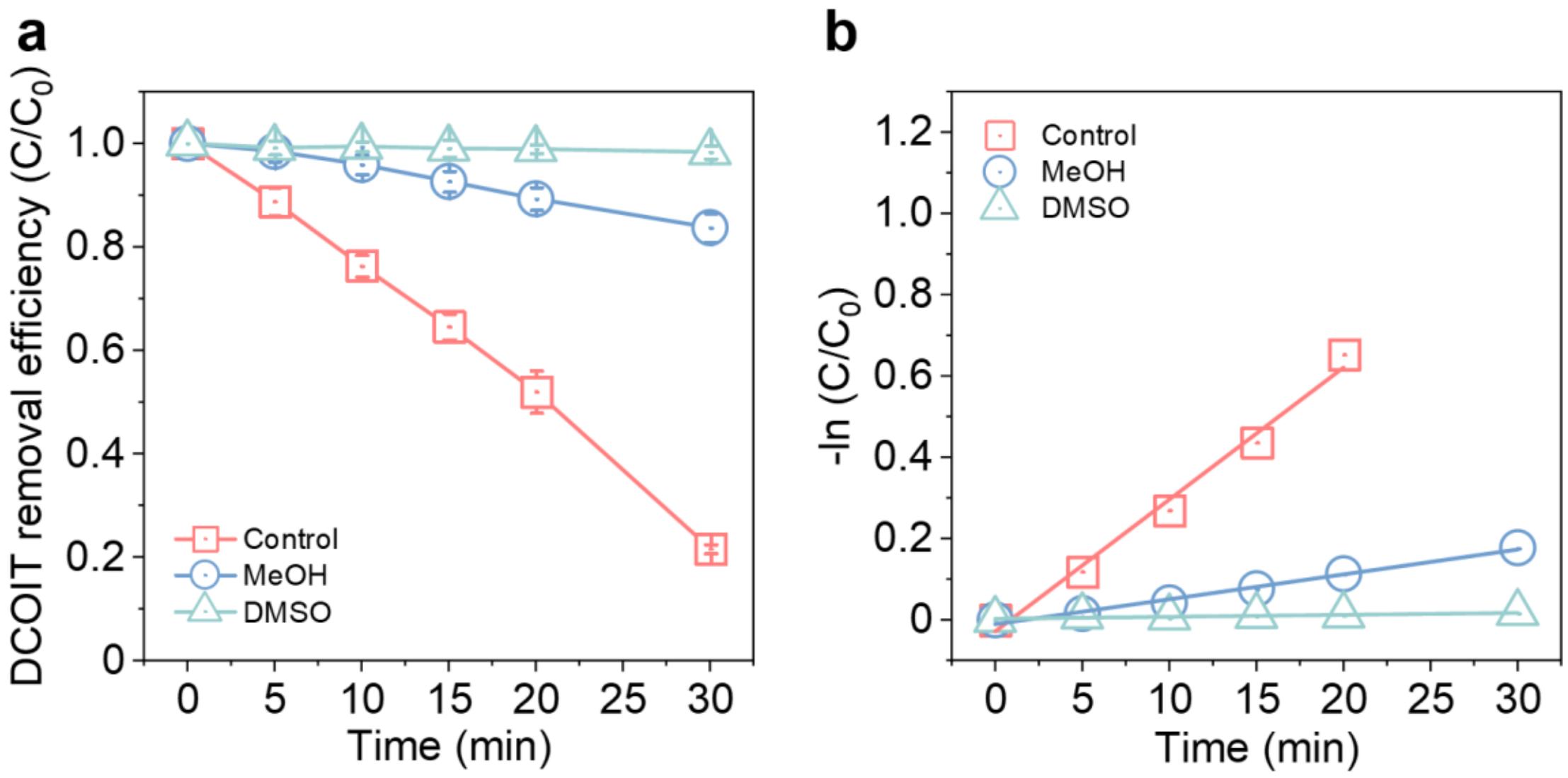

Supplementary Fig. 33. a. and b. Decay of DCOIT and corresponding first-order kinetic curves of DCOIT in quenching reaction processes in Fe^{3+}/BDD system. (Note: The -ln(C/C_0) value at 30 minutes in the control group was too high, making it unsuitable for kinetic fitting.) Error bars represent the standard deviation, obtained by repeating the experiment thrice (n = 3). Experimental conditions: [Na_2SO_4] = 50 mM, [DCOIT] = 20 μM, [Fe^{3+}] = 0.2 mM, [MeOH] = 200 mM, [DMSO] = 50 mM, current density = 7.5 mA cm^{-2}. Source data are provided as a Source Data file.

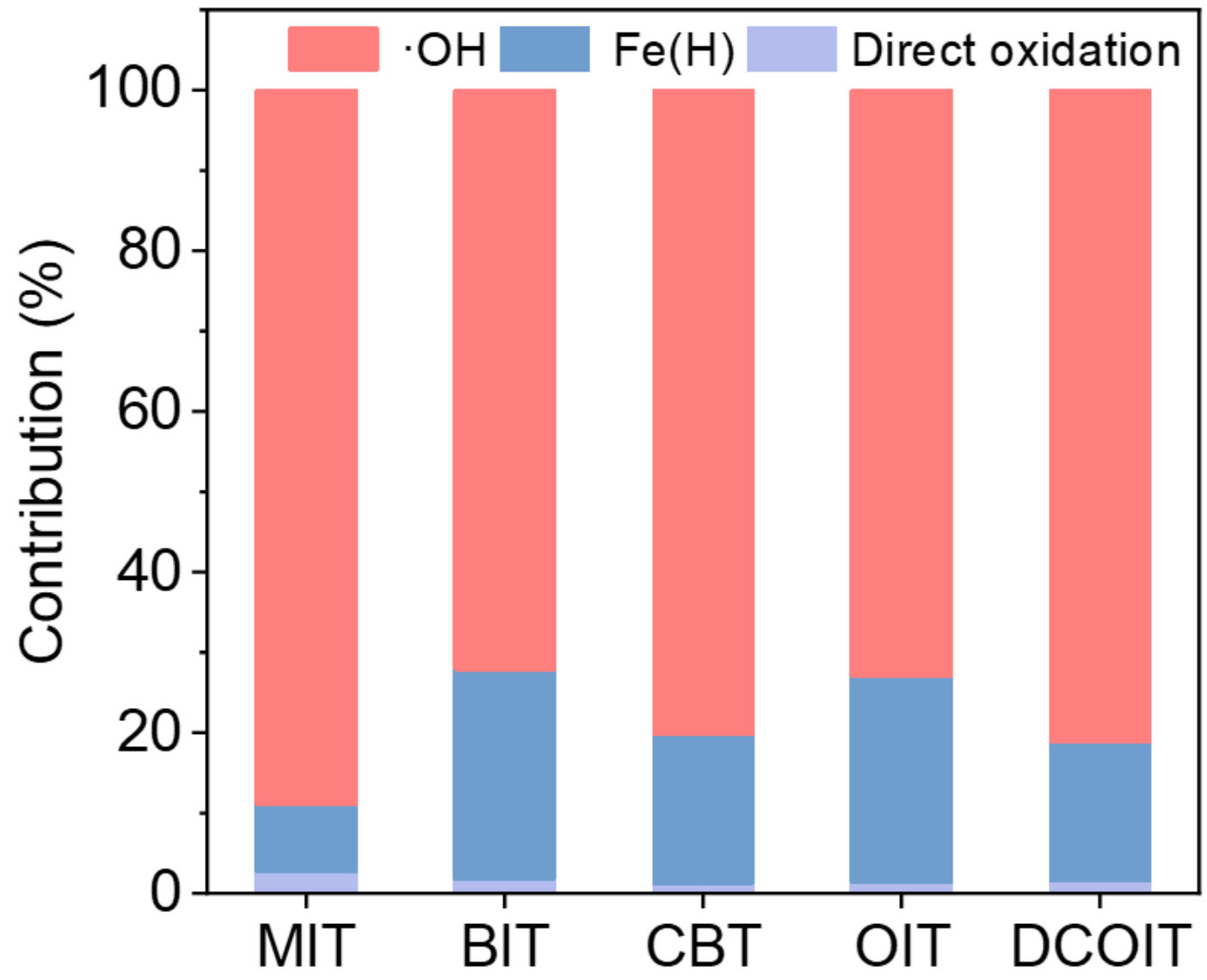

Supplementary Fig. 34. The relative contributions of •OH and Fe(H) to the degradation of MIT, BIT, DCBI, OIT and DCOIT. Experimental conditions: [Na_2SO_4] = 50 mM, [pollution] = 20 μM, [Fe^{3+}] = 0.2 mM, current density = 7.5 mA cm^{-2}. Source data are provided as a Source Data file.

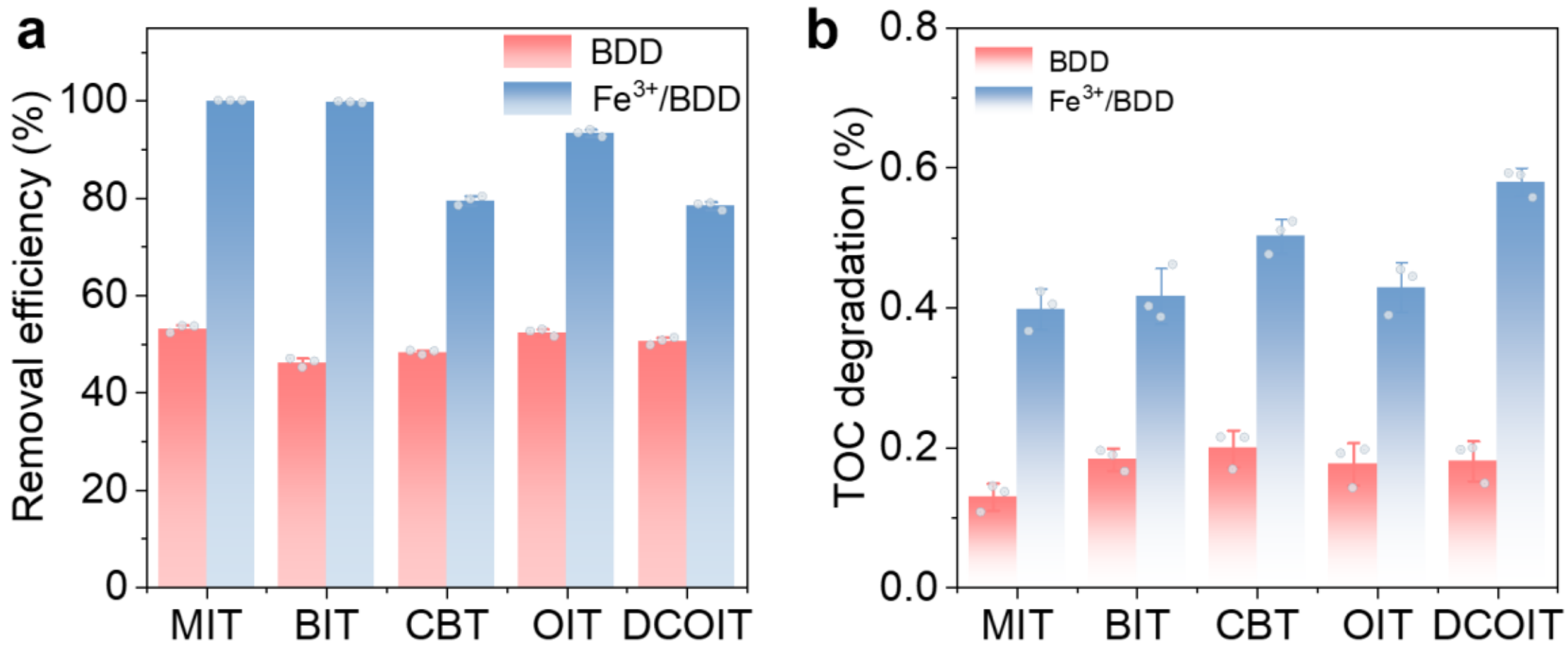

Supplementary Fig. 35. a. Comparison of removal efficiency of MIT, BIT, CBT, OIT and DCOIT **b.** and TOC removal rate in BDD and Fe^{3+}/BDD systems. Error bars represent the standard deviation, obtained by repeating the experiment thrice ($n = 3$). Experimental conditions: current density = 7.5 mA cm^{-2}, [Na_2SO_4] = 50 mM, [pollution] = 20 μM, [Fe^{3+}] = 0.2 mM. Source data are provided as a Source Data file.

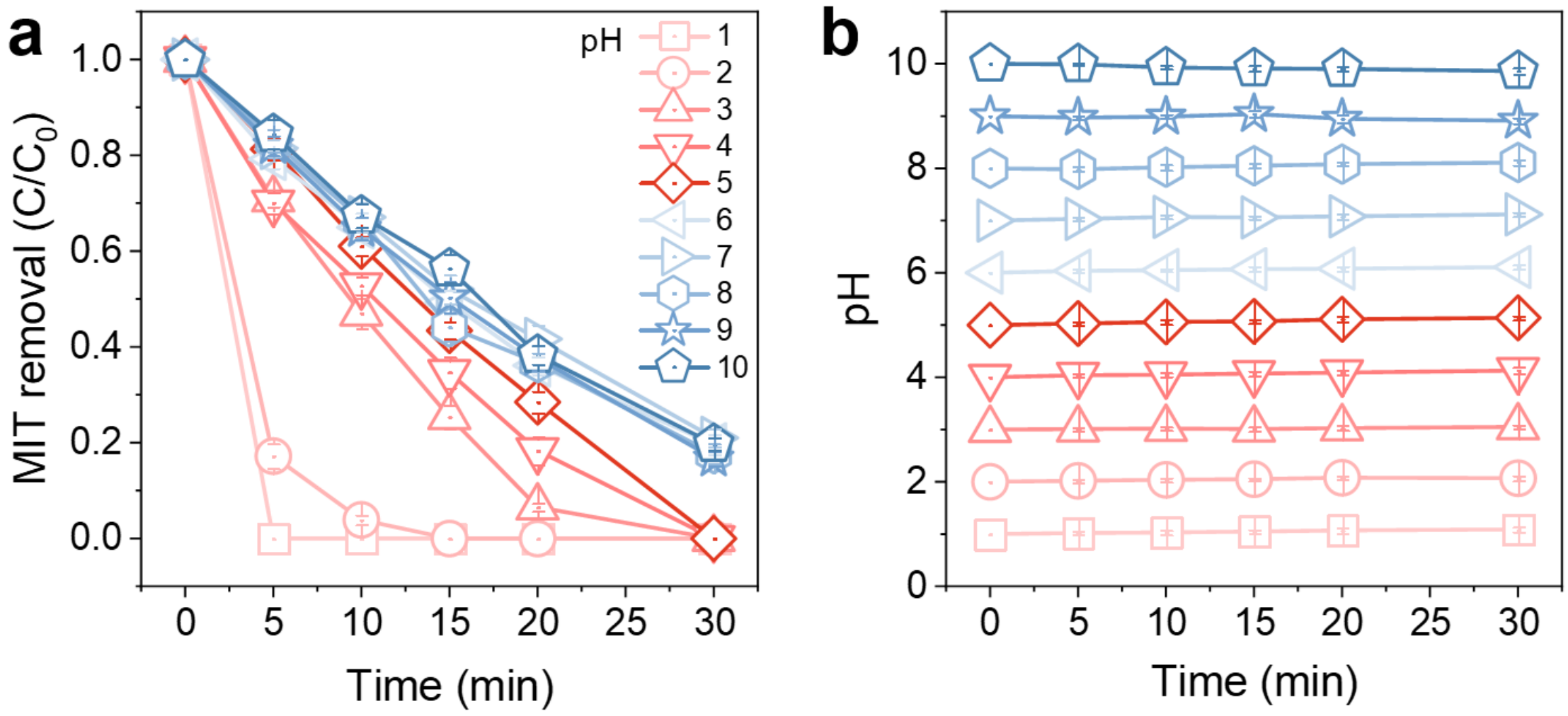

Supplementary Fig. 36. a. Effect of Fe^{3+}/BDD system on the removal efficiency of MIT under different initial pH conditions. **b.** The variation of pH with reaction time. Error bars represent the standard deviation, obtained by repeating the experiment thrice (n = 3). Experimental conditions: [Na_2SO_4] = 50 mM, [Fe^{3+}] = 0.2 mM, current density = 7.5 mA cm^{-2}. Source data are provided as a Source Data file.

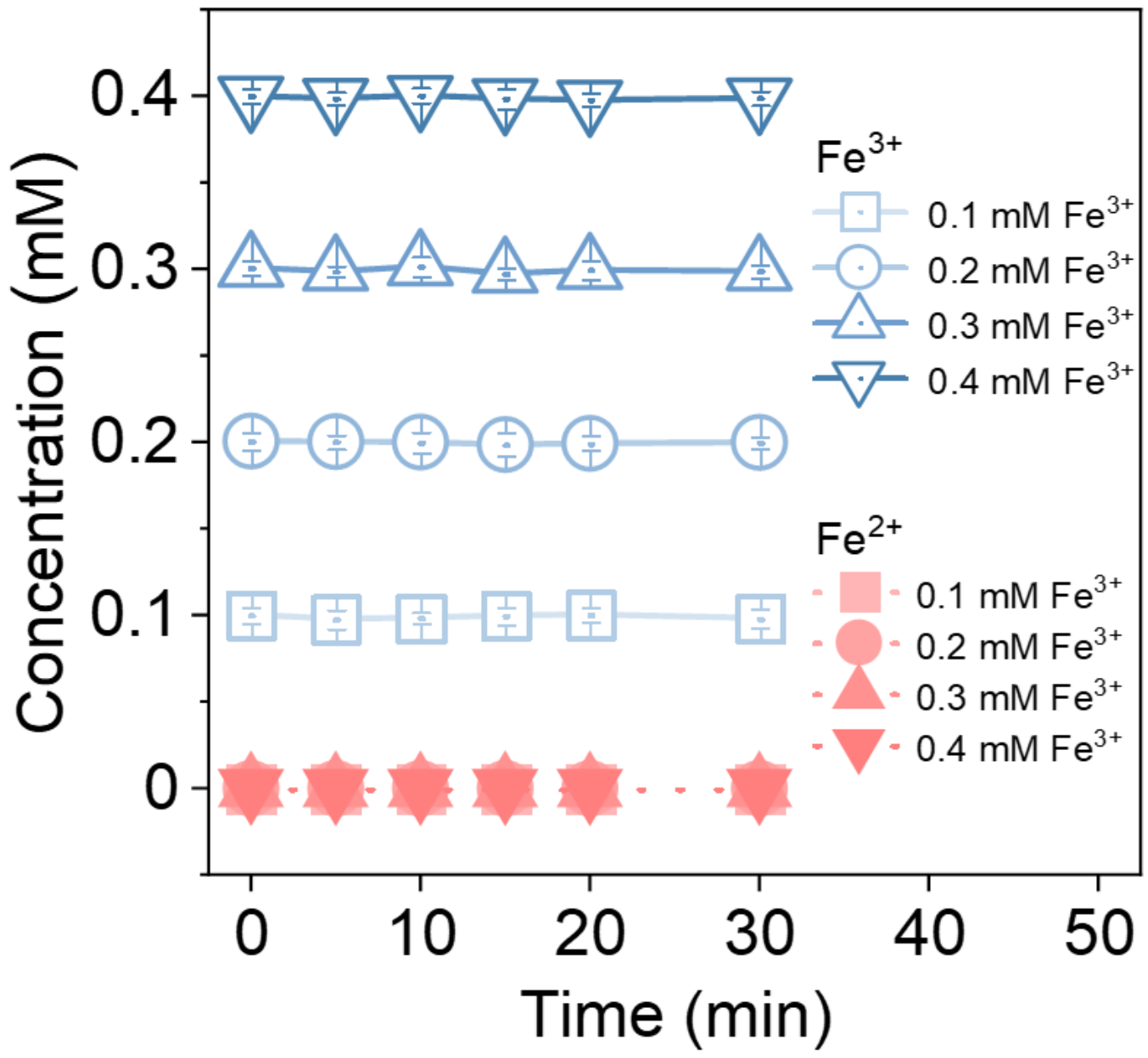

Supplementary Fig. 37. Variation of Fe^{2+} and Fe^{3+} concentrations in the Fe^{3+}/BDD system with reaction time. Error bars represent the standard deviation, obtained by repeating the experiment thrice (n = 3). Experimental conditions: [Na_2SO_4] = 50 mM, current density = 7.5 mA cm^{-2}. Source data are provided as a Source Data file.

Note: No Fe^{2+} was detected in this reaction system. The reduction of H_2O_2 was not attributed to the Fenton reaction.

Supplementary Table S1 UPLC conditions for organic contaminants analysis

Organic contaminants	Mobile phase (v/v)	Wavelength (nm)	Flow rate (mL/min)	Column temperature (°C)
MIT	Water/methanol (85/15)	275	0.8	40
BIT	0.1% Acetic acid/acetonitrile (70/30)	318	0.8	30
CBT	Water/methanol (20/80)	218	1	30
OIT	Water/methanol (20/80)	278	1	40
DCOIT	Water/methanol (60/40)	280	1	30
BA	0.1% Acetic acid/acetonitrile (70/30)	230	1	30
NB	Water/ acetonitrile (50/50)	268	1	30
BPA	Methanol/water (20/80)	250	0.8	30
DMP	Water /acetonitrile (50/50)	228	1	30
DEP	Water /acetonitrile (30/70)	228	1	30
SMX	Water /acetonitrile (50/50)	264	1	30
SIZ	0.1% Acetic	271	1	30

	acid/acetonitrile (60/40)			
CBZ	Water /acetonitrile (40/60)	286	1	30
IBU	0.1% Acetic acid/acetonitrile (20/80)	220	0.8	30
NAP	Methanol/0.1% Acetic acid (80/20)	231	1	30
PMSO<br>$PMSO_2$	0.1% Acetic acid/acetonitrile (70/30)	215	1	30

Supplementary Table S2 Elementary reactions during the Fe^{3+}/BDD system

No.	Reaction equation	E^0 or k	References
1	$BDD + H_2O \rightarrow BDD(\cdot OH) + H^+ + e^-$	E^0 = 1.8-2.7 V vs. RHE	[5]
2	$2BDD(\cdot OH) \rightarrow 2BDD + H_2O_2$	/	[6]
3	$Fe(III) + 2H_2O_2 \rightarrow Fe(II) + 2H_2O + O_2$	$k = 9.1\times10^{-7}\ M^{-1}s^{-1}$	[7]
4	$Fe(II) + 2H_2O_2 \rightarrow Fe(III) + \cdot OH + OH^-$	$k = 40\text{-}80\ M^{-1}s^{-1}$	[7]
5	$Fe(III) + BDD \rightarrow Fe(IV)$	/	[8]
7	$Fe(III) + \cdot OH \rightarrow Fe(IV) + OH^-$		[8]
8	$Fe(IV) + BDD \rightarrow Fe(VI)$		[8]

Note: E^0 represents standard redox potential, k represents reaction rate constant.

Supplementary Table S3 Catalytic performance comparison of recently typical anodic oxidation (AO) systems for pollutant removal

Catalyst/AO type	Current density (mA cm^{-2})	Pollutant concentration (μM)	Removal efficiency (%)	Removal time (min)	k_{obs} (min^{-1})	Ref.
Fe^{3+}/BDD	7.5	20	100 (MIT)	30	0.28	This work
MW/BDD	10	452.5	91.04 (2,4-DCP)	180	0.0133	[9]
BDD/PS	10	5	100 (CBZ)	30	0.059	[10]
UV/BDD	10	50	100 (NB)	60	0.075	[11]
$SnSbO_2/Mn_xCu_{1-x}O$	10	1064	100 (BF)	60	0.038	[12]
Sb-SnO_2-F	20	200	99 (PFOS)	120	0.035	[13]
3D CuNi-ATO	5	20.8	88.4 (TC)	120	0.06	[14]
$MnCuSnSbO_2$	20	55.4	100 (LEV)	30	0.188	[15]
Ti_4O_7	10	2	98.6 (PFOS)	30	0.008	[16]
TiO_2 NCs	2.22 V_{RHE}	155.6	95.6 (4-CP)	180	0.19	[17]
Co_3O_4-TiO_2	0.4 V_{SCE}	21.9	100 (BPA)	60	0.08	[18]
$(Ti_{1-x}Ce_x)_4O_7$	20	20	98.9 (PFOS)	120	0.083	[19]
RuO_2/Fe^{2+}	10	300	100 (MeP)	180	0.016	[20]
DSA/PS	5	5	30 (BPA)	15	0.013	[5]

Supplementary Table 4 List of assumptions and costs for treating 1 m^3 of wastewater using four different advanced oxidation processes (AOPs): anode oxidation (DSA)[21], electro-Fenton[22], and anode oxidation (this work)

AOPs	Cost classification	Cost items	Unit	Amount	Unit price (US$/kg or m^3)	Cost (US$)
**Anode oxidation (DSA)**	Chemical	Na_2SO_4	kg	7	0.129	0.9
	Electrode	Ti/IrO_2-Ta_2O_5 anode	kg	0.003	11000	0.3
		Ti cathode	kg	0.002	6	0.0002
	Electricity	Degradation	kW·h	175	0.07	12.3
						**13.5**
**Electro-Fenton**	Chemical	$Fe_2(SO_4)_3·7H_2O$	kg	9.3	0.097	0.9
		Na_2SO_4	kg	7	0.129	0.9
	Post-treatment	NaOH for pH adjustment	kg	0.02	0.57	0.0114
	Electricity	Degradation	kW·h	78.6	0.07	5.5
						**7.3**
**Anode oxidation (BDD)**	Chemical	Na_2SO_4	kg	7	0.129	0.9
	Electrode	BDD anode	kg	0.00035	1000	0.35
		Stainless steel cathode	kg	0.003	1.847	0.0006
	Electricity	Degradation	kW·h	83	0.07	5.81

						**7.06**
**Anode oxidation (BDD/Fe^{3+})**	Chemical	Na_2SO_4	kg	7.1	0.129	0.9
		$Fe_2(SO_4)_3 \cdot 6H_2O$	kg	0.1	0.097	0.0097
	Electrode	BDD anode	kg	0.0035	1000	0.35
		Stainless steel cathode	kg	0.003	1.847	0.0006
	Electricity	Degradation	kW·h	41	0.07	2.87
	Post-treatment	NaOH for pH adjustment	kg	0.02	0.57	0.0114
						**4.1**

Supplementary References

1. Wu L, Wang L, Liu YL, Zhao XN, Ma J. VUV Activated Fe(VI) by Promoting the Generation of Intermediate Valent Iron and Hydroxyl Radicals. *Environ. Sci. Technol.* **58**, 20256-20266 (2024).
2. Kang Y*, et al.* Unveiling the spatially confined oxidation processes in reactive electrochemical membranes. *Nat. Commun.* **14**, (2023).
3. Liu M*, et al.* Enhanced electrocatalytic CO2 reduction via field-induced reagent concentration. *Nature* **537**, 382-386 (2016).
4. Chen Y, Zhang G, Ji Q, Lan H, Liu H, Qu J. Visualization of Electrochemically Accessible Sites in Flow-through Mode for Maximizing Available Active Area toward Superior Electrocatalytic Ammonia Oxidation. *Environ. Sci. Technol.* **56**, 9722-9731 (2022).
5. Ding J, Bu L, Zhao Q, Kabutey FT, Wei L, Dionysiou DD. Electrochemical activation of persulfate on BDD and DSA anodes: Electrolyte influence, kinetics and mechanisms in the degradation of bisphenol A. *J. Hazard. Mater.* **388**, (2020).
6. Salazar C, Ridruejo C, Brillas E, Yáñez J, Mansilla HD, Sirés I. Abatement of the fluorinated antidepressant fluoxetine (Prozac) and its reaction by-products by electrochemical advanced methods. *Appl. Catal. B-Environ.* **203**, 189-198 (2017).
7. Sun YM*, et al.* Hydrogen Peroxide as an Ideal Electron Donor for Long-Lasting Fenton Chemistry: Strong Enhancement of Fe(III) Activity by Heteroatom-Doped Nanocarbons. *Acs. Catal.* **14**, 6525-6534 (2024).
8. Wang SC, Lin YM, Shao BB, Dong HY, Ma J, Guan XH. Selective Removal of Emerging Organic Contaminants from Water Using Electrogenerated Fe(IV) and Fe(V) under Near-Neutral Conditions. *Environ. Sci. Technol.* **57**, 9332-9341 (2023).
9. Gao J, Zhao G, Shi W, Li D. Microwave activated electrochemical degradation of 2,4-dichlorophenoxyacetic acid at boron-doped diamond electrode. *Chemosphere* **75**, 519-525 (2009).
10. Song HR*, et al.* Electrochemical activation of persulfates at BDD anode: Radical or nonradical oxidation? *Water Res.* **128**, 393-401 (2018).
11. Chen P*, et al.* Shifts of surface-bound •OH to homogeneous •OH in BDD electrochemical system via UV irradiation for enhanced degradation of hydrophilic aromatic compounds. *Chemosphere* **291**, (2022).
12. Wang R*, et al.* Electrochemical system with moderate ozone generation and catalytic enhancement for efficient organic wastewater treatment. *Water Res.* **287**, (2025).
13. Yang B*, et al.* Electrochemical mineralization of perfluorooctane sulfonate by novel F and Sb co-doped Ti/SnO_2 electrode containing Sn-Sb interlayer. *Chem. Eng. J.* **316**, 296-304 (2017).
14. Jin B, Cheng S, Li L, Li H, Zhou Y, Chen H. Self-supporting three-dimensional CuNi–Sb–SnO_2 anode with ultra-long service life for efficient removal of antibiotics in wastewater. *Chemosphere* **365**, (2024).
15. Chen Y*, et al.* Cu/Mn-mediated electron shuttle and high-valent metals enhance hydroxyl radicals production during the electrochemical oxidation on the CuMn-Sb-SnO_2 electrode. *J. Hazard. Mater.* **477**, (2024).

16. Wang L, Lu J, Li L, Wang Y, Huang Q. Effects of chloride on electrochemical degradation of perfluorooctanesulfonate by Magnéli phase Ti_4O_7 and boron doped diamond anodes. *Water Res.* **170**, (2020).
17. Song R*, et al.* Highly Efficient Degradation of Persistent Pollutants with 3D Nanocone TiO_2-Based Photoelectrocatalysis. *J. Am. Chem. Soc.* **143**, 13664-13674 (2021).
18. Hu Y*, et al.* Interface and Defect Engineering in 3D Co_3O_4-O_v/TiO_2 to Boost Simultaneous Removal of BPA and Cr(VI) upon Photoelectrocatalytic/Peroxymonosulfate (PEC/PMS) System. *Adv. Funct. Mater.* **35**, (2024).
19. Lin H*, et al.* Defect Engineering on a Ti_4O_7 Electrode by Ce^{3+} Doping for the Efficient Electrooxidation of Perfluorooctanesulfonate. *Environ. Sci. Technol.* **55**, 2597-2607 (2021).
20. Steter JR, Brillas E, Sirés I. Solar photoelectro-Fenton treatment of a mixture of parabens spiked into secondary treated wastewater effluent at low input current. *Appl. Catal. B-Environ.* **224**, 410-418 (2018).
21. Pontes JPSD, da Costa PRF, da Silva DR, Garcia-Segura S, Martínez-Huitle CA. Methylene Blue decolorization and Mineralization by Means of Electrochemical Technology at Pre-pilot Plant Scale: Role of the Electrode Material and Oxidants. *Int. J. Electrochem. Sc.* **11**, 4878-4891 (2016).
22. Mousset E, Loh WH, Lim WS, Jarry L, Wang Z, Lefebvre O. Cost comparison of advanced oxidation processes for wastewater treatment using accumulated oxygen-equivalent criteria. *Water Res.* **200**, (2021).
