# Supplementary material for: Reconfiguring interfacial hydrogen-bond networks to enhance anodic oxidation for efficient wastewater treatment
Source: Nat Commun. 2026 Oct 5;17:11322. doi: 10.1038/s41467-026-78208-2 (PMC13724709; doi:10.1038/s41467-026-78208-2)
Supplement: Supplementary file 2 [file 41467_2026_78208_MOESM2_ESM.pdf]

nature portfolio

Corresponding author(s): Bo Lai

Last updated by author(s): Sep 5, 2026

Reporting Summary

Nature Portfolio wishes to improve the reproducibility of the work that we publish. This form provides structure for consistency and transparency in reporting. For further information on Nature Portfolio policies, see our Editorial Policies and the Editorial Policy Checklist.

Statistics

For all statistical analyses, confirm that the following items are present in the figure legend, table legend, main text, or Methods section.

n/a	Confirmed	
☐	☒	The exact sample size (n) for each experimental group/condition, given as a discrete number and unit of measurement
☐	☒	A statement on whether measurements were taken from distinct samples or whether the same sample was measured repeatedly
☒	☐	The statistical test(s) used AND whether they are one- or two-sided<br>*Only common tests should be described solely by name; describe more complex techniques in the Methods section.*
☒	☐	A description of all covariates tested
☒	☐	A description of any assumptions or corrections, such as tests of normality and adjustment for multiple comparisons
☐	☒	A full description of the statistical parameters including central tendency (e.g. means) or other basic estimates (e.g. regression coefficient) AND variation (e.g. standard deviation) or associated estimates of uncertainty (e.g. confidence intervals)
☒	☐	For null hypothesis testing, the test statistic (e.g. F, t, r) with confidence intervals, effect sizes, degrees of freedom and P value noted<br>*Give P values as exact values whenever suitable.*
☒	☐	For Bayesian analysis, information on the choice of priors and Markov chain Monte Carlo settings
☒	☐	For hierarchical and complex designs, identification of the appropriate level for tests and full reporting of outcomes
☒	☐	Estimates of effect sizes (e.g. Cohen's d, Pearson's r), indicating how they were calculated

Our web collection on statistics for biologists contains articles on many of the points above.

Software and code

Policy information about availability of computer code

Data collection	COMSOL simulations were performed using COMSOL Multiphysics 6.2, AIMD simulations were carried out with CP2K, and all other data were obtained through direct experiments. No custom code was used.
Data analysis	origin 2021

For manuscripts utilizing custom algorithms or software that are central to the research but not yet described in published literature, software must be made available to editors and reviewers. We strongly encourage code deposition in a community repository (e.g. GitHub). See the Nature Portfolio guidelines for submitting code & software for further information.

Data

Policy information about availability of data

All manuscripts must include a data availability statement. This statement should provide the following information, where applicable:

- Accession codes, unique identifiers, or web links for publicly available datasets
- A description of any restrictions on data availability
- For clinical datasets or third party data, please ensure that the statement adheres to our policy

The data supporting the findings of the study are included in the main text and supplementary information files. Additional data are available from the corresponding author upon request. Source data are provided with this paper.

Research involving human participants, their data, or biological material

Policy information about studies with human participants or human data. See also policy information about sex, gender (identity/presentation), and sexual orientation and race, ethnicity and racism.

Reporting on sex and gender	This study does not involve human participants, their data, or biological material.
Reporting on race, ethnicity, or other socially relevant groupings	This study does not involve human participants, their data, or biological material.
Population characteristics	This study does not involve human participants, their data, or biological material.
Recruitment	This study does not involve human participants, their data, or biological material.
Ethics oversight	This study does not involve human participants, their data, or biological material.

Note that full information on the approval of the study protocol must also be provided in the manuscript.

Field-specific reporting

Please select the one below that is the best fit for your research. If you are not sure, read the appropriate sections before making your selection.

☐ Life sciences ☐ Behavioural & social sciences ☒ Ecological, evolutionary & environmental sciences

For a reference copy of the document with all sections, see nature.com/documents/nr-reporting-summary-flat.pdf

Ecological, evolutionary & environmental sciences study design

All studies must disclose on these points even when the disclosure is negative.

Study description	In this study, we unveil an innovative mechanism where iron ions (Fe3+) act not merely as outer-sphere reaction center, but as "architects" of the interfacial microenvironment. By inducing a massive reconfiguration of the hydrogen-bond (H-bond) network, we successfully lower the energy barrier for water oxidation.
Research sample	The study used boron-doped diamond as the anode and aqueous methylisothiazolinone solution as the model pollutant system, conducting batch degradation experiments with sodium sulfate and iron(III) sulfate as additives.
Sampling strategy	Batch experiments were conducted under controlled conditions using fixed electrodes, constant current density, electrolyte concentration, and solution volume. For comparison, control experiments without ferrous sulfate or other electrodes were also set up, and all experiments were independently repeated to ensure consistency of results.
Data collection	Data Collection:<br>Organic residue concentrations in the reaction solution were determined by HPLC, while reactive oxygen species during the reaction process were measured using EPR. Data on electrode interface reactions and interfacial water structure were obtained through standard characterization techniques including in situ electrochemical testing, in situ Raman spectroscopy, and in situ FTIR spectroscopy. All measurements were conducted according to standard instrument operating procedures, and data were recorded using the respective instrument-specific data acquisition software.
Timing and spatial scale	All experiments were conducted under laboratory conditions at room temperature. Reaction times ranged from minutes to several hours depending on the specific experiment.
Data exclusions	No data were excluded from the analyses.
Reproducibility	Parallel control experiments were conducted, and consistent trends were observed across independent experiments. All experiments were independently repeated at least three times using freshly prepared samples.
Randomization	Randomization was not applicable as experiments were conducted under controlled laboratory conditions with predefined parameters.
Blinding	No binding was used. This work is not a clinical research (or related research) study.

Did the study involve field work? ☐ Yes ☒ No

Reporting for specific materials, systems and methods

We require information from authors about some types of materials, experimental systems and methods used in many studies. Here, indicate whether each material, system or method listed is relevant to your study. If you are not sure if a list item applies to your research, read the appropriate section before selecting a response.

Materials & experimental systems

n/a	Involved in the study
☒	☐ Antibodies
☒	☐ Eukaryotic cell lines
☒	☐ Palaeontology and archaeology
☒	☐ Animals and other organisms
☒	☐ Clinical data
☒	☐ Dual use research of concern
☒	☐ Plants

Methods

n/a	Involved in the study
☒	☐ ChIP-seq
☒	☐ Flow cytometry
☒	☐ MRI-based neuroimaging

Plants

Seed stocks	none
Novel plant genotypes	none
Authentication	none
