# Supplementary material for: Reconfiguring interfacial hydrogen-bond networks to enhance anodic oxidation for efficient wastewater treatment
Source: Nat Commun. 2026 Oct 5;17:11322. doi: 10.1038/s41467-026-78208-2 (PMC13724709; doi:10.1038/s41467-026-78208-2)
Supplement: Supplementary file 3 [file 41467_2026_78208_MOESM3_ESM.pdf]

nature portfolio

Peer Review File

Reconfiguring Interfacial Hydrogen-Bond Networks to Enhance Anodic Oxidation for Efficient Wastewater Treatment

Corresponding Author: Professor Bo Lai

This file contains all reviewer reports in order by version, followed by all author rebuttals in order by version.

Version 0:

Reviewer comments:

Reviewer #1

(Remarks to the Author)
This work reports a Fe^{3+}-mediated strategy to reconfigure the interfacial hydrogen-bond network of water molecules for enhanced anodic oxidation. The study breaks the traditional research paradigm that focuses on electrode material modification, and instead proposes a facile electrolyte engineering approach—simply introducing trace Fe^{3+} to precisely reconfigure the hydrogen-bond network of interfacial water molecules on the BDD anode, which can boost •OH production and induce long-lived high-valent iron species formation, achieving the oxidation kinetics enhancement and oxidation scope expansion. The study is innovative, with in depth mechanistic analysis and promising practical application potential. A few minor revisions and clarifications are suggested to further improve the clarity of the work. Below are some suggestions for the authors' consideration.

1. Abstract: It is suggested to add a brief description of the specific types of emerging contaminants that achieve efficient degradation, such as antimicrobials, industrial chemicals, to make the research achievements more specific and intuitive.
2. Abstract: The manuscript mentions a 500-hour stable long-term flow operation but does not specify the pollutant removal efficiency maintained during this period. It is recommended to add this key performance parameter.
3. Line 98: Fe3+ is oxidized to high-valent iron (Fe(H), including Fe(IV), Fe(V), Fe(VI)). Please unify the notation of high-valent iron throughout the manuscript, and briefly clarify why Fe(VI) is classified into Fe(H) to avoid notation confusion.
4. Figure 2c and Table S3: The manuscript compares the pollutant normalization degradation kinetics with state-of-the-art anodic oxidation studies. It is recommended to give detailed parameters, including current density and pollutant concentration.
5. Line 162: •OH and Fe(H) contribute 83.5% and 13.4% to MIT removal respectively. It is suggested to briefly discuss the reason for the large difference in contribution ratios to deepen the analysis of reactive species action mechanisms.
6. Line 182: The authors describes the COMSOL simulation of the concentration distribution of •OH and Fe(H). It is suggested to supplement the key simulation parameters in the Supporting Information, and briefly explain the selection basis of these parameters.
7. Line 300: The manuscript classifies interfacial water into 4-HB-H_2O, 2-HB-H_2O and free H_2O. It is recommended to explain the Raman characteristic peak positions corresponding to each type of water, to make the spectral analysis more accessible for readers who are not familiar with interfacial water characterization.
8. Line 371: Figure 5f shows that Fe^{3+} inhibits the formation of H_2O_2 from •OH dimerization. It is recommended to add the detection limit of the H_2O_2 test method, to clarify the reliability of the low-concentration H_2O_2 data.
9. Line 418: The authors investigate the effect of typical anions and humic acid on MIT degradation. It is suggested to add the concentration range of this pollutant in actual wastewater in the discussion, to better reflect the practical application value of the Fe^{3+}/BDD system.

Reviewer #2

(Remarks to the Author)
This manuscript presents a thoughtfully designed and conceptually appealing study on reconfiguring interfacial hydrogen-bond (H-bond) networks via Fe^{3+} to enhance anodic oxidation for wastewater treatment. The integration of electrochemical

analysis, in situ spectroscopy, and theoretical simulations provides a relatively comprehensive framework linking electrolyte engineering to catalytic performance. The manuscript is clearly written, logically structured, and the results are, in general, convincing. The proposed strategy offers a potentially impactful paradigm for advancing electrochemical water treatment technologies with practical relevance.

That said, given the growing interest in interfacial water regulation and electrolyte effects in electrocatalysis, several aspects of the mechanistic interpretation would benefit from further clarification or additional discussion to meet the expectations of a high-impact readership. In particular, some key claims rely on indirect evidence and would be strengthened by more rigorous justification. Overall, I recommend minor revision, provided the authors address the following points:

Specific Comments:
1.In the Abstract, the statement that 4-HB-H_2O motifs increase by 16% is central to the mechanistic claim. The authors are encouraged to briefly indicate the baseline value (e.g., initial percentage) to improve clarity and allow immediate contextual interpretation.
2.In the Results and Discussion, 0.2 mM Fe^{3+} is selected as the optimal concentration, although 1 mM exhibits a higher kobs. The authors should elaborate on the practical trade-offs (e.g., chemical consumption, possible sludge formation, or diminishing energy efficiency) that justify this selection.
3.Identification of high-valent iron species is important mechanistic point but not clear in the manuscript. In Fig. 3a and related discussion, the formation of Fe(IV), Fe(V), and Fe(VI) is proposed. Given the central role of these species, the authors should clarify which high-valent iron species predominantly contributes to pollutant oxidation. Additionally, it would strengthen the manuscript to discuss the limitations of current evidence (e.g., Raman assignment, probe reactions) and whether more definitive techniques (e.g., XAS or Mössbauer spectroscopy) could further validate these assignments.
4.In Figure 4a, the non-monotonic chemical shift with increasing Fe^{3+} concentration is interesting. Please clarify why the paramagnetic effect becomes dominant only above ~0.4 mM Fe^{3+}, and whether this threshold is consistent with known Fe^{3+} hydration or clustering behavior.
5.In Figure 5f, the decrease in H_2O_2 concentration is attributed to suppressed •OH dimerization. The authors are encouraged to consider and experimentally evaluate whether Fe^{3+} (or Fe(H) species) could also directly decompose H_2O_2, to exclude alternative pathways.
6.The 500 h continuous operation (Figure 6d) is impressive. It would be valuable to report the Fe concentration (or speciation, if possible) during long-term operation to confirm the stability and recyclability of the Fe catalytic center.
7.The system operates at pH ≈ 3. The authors should briefly discuss the pH tolerance of the system and the implications for practical wastewater treatment, particularly regarding operation under near-neutral conditions.
8.The study focuses on BDD electrodes. The authors are encouraged to comment on whether this Fe^{3+}-mediated H-bond network regulation strategy is transferable to other anodic materials (e.g., DSA, Ti_4O_7), which would enhance the general significance of the work.
9.While the increase in 4-HB-H_2O is correlated with improved performance, the causal relationship remains somewhat indirect. The authors are encouraged to strengthen the discussion by clarifying how changes in H-bond structure quantitatively relate to reaction kinetics or •OH yield, and to acknowledge potential uncertainties in Raman-based deconvolution.
10.Several conclusions regarding interfacial water structure are partially supported by bulk-sensitive techniques (e.g., ^1H NMR). The authors should clarify this limitation and distinguish more explicitly between bulk hydration effects and true interfacial phenomena.
11.Figure presentation and quantitative rigor are required to be strengthened. Some figures (e.g., Fig. 2c, Fig. 6a) would benefit from inclusion of error bars or statistical information to improve quantitative rigor and reproducibility.

Reviewer #3

(Remarks to the Author)
This manuscript reports that the introduction of Fe3+ strengthens the interfacial hydrogen-bond network and thereby enhances both •OH formation and high-valent iron production in a BDD anodic oxidation system. The topic is potentially interesting, and the authors attempt to connect interfacial water structure with anodic oxidation performance. However, in the present form, the mechanistic argument is not sufficiently substantiated, and the central conclusion appears to go beyond what the data can support.

1. The novelty of this work requires further consideration.
In line 90-92 the article stated that its novelty lies in the limited prior exploration of “microscopic regulations of the water environment at the anode solid-liquid interface and increase the production and utilization rate of •OH”.
However, the hydrogen-bond mediated interface electrolyte engineering is a generally used method in regulating activity, selectivity, and durability (https://doi.org/10.1016/j.joule.2025.102306). And regulation of interfacial hydrogen-bond networks through the introduction of functional ligands, molecular additives, or ionic species has already been explored in several studies (https://doi.org/10.1016/j.ijhydene.2025.152286 ; https://doi.org/10.1021/jacs.5c22753 ; https://doi.org/10.1002/adfm.202526825)
Moreover, the increased production of •OH remains insufficiently convincing. Its contribution to overall oxidation enhancement is not convincingly separated from high-valent iron species formation. BDD anode assisted high-valent iron species production in water treatment area, is not conceptually new. (https://doi.org/10.1021/acs.est.2c09237; https://doi.org/10.1021/acs.est.3c01850)

2. The evidence for enhanced •OH production upon Fe^{3+} addition is internally inconsistent.
1) Based on Fig. 2d, the removal ratio quenched by TBA and MeOH in the systems with and without Fe^{3+} do not appear substantially different (100% to 38.7% and 53.2% to 8.4%, respectively). If this interpretation is correct, the contribution of •OH to oxidation may be similar in the two systems, which would weaken the conclusion that Fe^{3+} markedly enhances •OH production.
2) According to the equation S4 provided by the authors, since k_{NB} is constant (3×10^9 M^{-1} s^{-1}), $[•OH]_{ss}$ should be proportional to $k_{obs,NB}$. However, In Fig. 2b, the k_{obs} at 0.4-0.8 mM Fe^{3+} seems nearly unchanged, whereas Fig. 2f shows a clear stepwise increase. The manuscript should explain this inconsistency.
3) The relationship between Fig. 2g and Fig. S8 is confusing. In Fig. 2g, the contribution of Fe(H) is reported to be only 13.41% under 2 mM Fe^{3+}, whereas in Fig. S8 the $PMSO_2$ yield under 1 mM Fe^{3+} exceeds 50%. Since PMSO yield is commonly used as an indicator for high-valent iron contributions, these two results appear difficult to reconcile. The authors need to clearly explain how these values were calculated and why the inferred oxidative contribution of high-valent iron differs substantially between the two datasets.
4) Could the authors add a scale bar to indicate the simulated diffusion layer thickness in Fig. 2h? Moreover, within the same thickness range, the simulated concentration of Fe(H) appears to be approximately twice that of •OH. It is also unclear whether this value is directly calculated from the model or defined based on specific assumptions. In addition, if MIT oxidation mainly occurs at or near the interface, it is not clear whether •OH can still be considered the dominant oxidizing species, as stated in Line 154.

3. The key evidence supporting hydrogen-bond reconfiguration is weak.
1) Line 269-276: The ^1H NMR indicated upfield shift at 0.2–0.4 mM Fe^{3+} addition cannot fully support the claim "forming an extended and strongly hydrogen-bonded hydrated sphere centered on $[Fe(OH)(H_2O)_5]^{2+}$, with an increase in the electron cloud density of the H atoms of water molecules and an enhanced H-bond network."
2) The claim that the peaks in Fig. 4d undergo a pronounced red shift (line 304-306) with increasing Fe^{3+} concentration is not sufficiently supported by the presented data. The 0.6-1.0 mM Fe^{3+} peaks appear to show a blue shift rather than a red shift.
3) line 303-306: the authors state, based on in situ Raman spectroscopy, that with increasing Fe^{3+} concentration, the characteristic band at ~3200 cm^{-1} assigned to 4-HB-H_2O shows enhanced intensity and a pronounced red shift, which is then interpreted as strengthened hydrogen bonding, further weakening and polarization of O–H bonds, and a "pre-activated" water state.
This description needs to be reformulated more rigorously.

4. Important operational parameters are insufficiently monitored or discussed.
For both the MIT oxidation and potential environmental application, the Fe^{3+} concentration and pH are not discussed. The added Fe concentration of from 11.2 to 56 mg/L is not negligible for a water-treatment system and may be strongly influenced by the reaction pH. The authors are therefore encouraged to provide additional data on the evolution of Fe concentration and pH during the reaction.

5. The possibility of an ozone-mediated pathway is not adequately excluded.
This is a major mechanistic concern.
Because the system employs a BDD anode under strongly anodic conditions, the possible formation of ozone should be carefully considered (https://doi.org/10.1016/j.jechem.2025.05.016; https://doi.org/10.1021/acs.est.2c05290). If ozone is generated electrochemically, then Fe species may further activate ozone and produce additional reactive oxygen species, mainly •OH (https://doi.org/10.1016/j.watres.2024.121907). If so, the observed enhancement may rise from an ozone-iron interaction pathway, rather than from hydrogen-bond reconfiguration.

6. In Lines 371-380, the decrease in H_2O_2 concentration (Fig 5f) after Fe^{3+} addition cannot by itself support the conclusion that Fe^{3+} suppresses •OH recombination into H_2O_2.

7. Line 187, "Fig. 1h" should presumably be corrected to "Fig. 2h".

8. Line 213, "This shift confirms that Fe^{3+} significantly the kinetics of interfacial water oxidation on the BDD anode." A missing verb.

Version 1:

Reviewer comments:

Reviewer #1

(Remarks to the Author)
The authors have well-revised the manuscript, it can be accepted for publication.

Reviewer #2

(Remarks to the Author)
The authors have responded to my previous concerns in a thorough and satisfactory manner. The revisions have substantially improved the manuscript. I have no remaining objections and recommend the paper for publication.

Reviewer #3

(Remarks to the Author)
I would like to thank the authors for their careful and thorough revision of the manuscript. The revised version has been substantially improved in both clarity and rigor through the refinement of the discussion and the addition of supporting experiments. In particular, the novelty of the work is now more clearly articulated: Fe^{3+} promotes anodic •OH generation by modulating the interfacial water hydrogen-bond network. Overall, the manuscript is now much stronger than the previous version. However, a few minor issues should still be addressed before publication.

1. In the revised manuscript, Figure 2 experiment condition "[TBA] = [MeOH] = 200 mM" should be revised according to the new data. It is indicated in the response letter that "the data in Figures 2d and Figures 2g of the manuscript (using 500 mM MeOH as the quenching agent)".

2. With the additional experiment in Figure 3a, it is now clearer that •OH makes a greater contribution in the MIT oxidation system because MIT has a much higher reaction rate constant with •OH. To better distinguish the results shown in Figure 3 and Figure S9, the authors are suggested to indicate explicitly that the experiment in Figure 3 was conducted in the presence of MIT.

3. The descriptions on page 16, line 306, such as "appear as dispersed bulk water" and "strong electron-withdrawing effect", seem to require further justification or more precise wording.

4. In the revised manuscript, page 7, line 113, "the Fe^{3+} are oxidized to high-valent iron" should be corrected to "the Fe^{3+} is oxidized to high-valent iron".

5. In the revised manuscript page 8, line 116-120: "In situ electrochemical spectroscopy and ab initio molecular dynamics (AIMD) demonstrates that $[Fe(OH)(H_2O)_5]^{2+}$ enhances the 4-HB-H_2O and H-bond population at the electrode surface (Figure S1)."
The authors may want to reconsider referring to Figure S1 in the Introduction section, as it feels slightly unusual to cite a supplementary figure at this stage. And "demonstrates" should be corrected to "demonstrate".

6. Page 10, line 175: please check the double space in "MeOH and TBA".

7. It is also suggested that the authors check the consistency of tense when presenting the results, as there appears to be a mixture of past and present tense throughout the manuscript.

Open Access This Peer Review File is licensed under a Creative Commons Attribution 4.0 International License, which permits use, sharing, adaptation, distribution and reproduction in any medium or format, as long as you give appropriate credit to the original author(s) and the source, provide a link to the Creative Commons license, and indicate if changes were made.
In cases where reviewers are anonymous, credit should be given to 'Anonymous Referee' and the source.

The images or other third party material in this Peer Review File are included in the article's Creative Commons license, unless indicated otherwise in a credit line to the material. If material is not included in the article's Creative Commons license and your intended use is not permitted by statutory regulation or exceeds the permitted use, you will need to obtain permission directly from the copyright holder.
To view a copy of this license, visit https://creativecommons.org/licenses/by/4.0/

Responses to Reviewers' Comments

Manuscript ID: NCOMMS-26-019794A

Title: "Reconfiguring Interfacial Hydrogen-Bond Networks to Enhance Anodic Oxidation for Efficient Wastewater Treatment"

Author(s): Tong Liu [1], Shuang Zhong [1], Xiaotong Liang, Hongrui Zhu, Ye Du* & Bo Lai*

We would like to thank you for the valuable comments, which have helped us improve the quality and scientific merits of the manuscript. The manuscript has been carefully revised with point-to-point responses to comments as listed below. The revised portions were marked in red in the revised manuscript, and we hope the revision can be satisfactory.

Reviewer #1 (Remarks to the Author):

General comments: This work reports a Fe^{3+}-mediated strategy to reconfigure the interfacial hydrogen-bond network of water molecules for enhanced anodic oxidation. The study breaks the traditional research paradigm that focuses on electrode material modification, and instead proposes a facile electrolyte engineering approach—simply introducing trace Fe^{3+} to precisely reconfigure the hydrogen-bond network of interfacial water molecules on the BDD anode, which can boost •OH production and induce long-lived high-valent iron species formation, achieving the oxidation kinetics enhancement and oxidation scope expansion. The study is innovative, with in depth mechanistic analysis and promising practical application potential. A few minor revisions and clarifications are suggested to further improve the clarity of the work. Below are some suggestions for the authors' consideration.

Response: We express our gratitude to you for the highly positive feedback regarding the novelty and scientific merits of our manuscript. We also thank you for these valuable suggestions, which would be very beneficial to improving the manuscript. We accepted all the suggestions and have revised the manuscript accordingly.

Specific comments:

1. Comment 1: Abstract: It is suggested to add a brief description of the specific types of emerging contaminants that achieve efficient degradation, such as antimicrobials, industrial chemicals, to make the research achievements more specific and intuitive.

Response: We appreciate your valuable suggestions. We have specified in the Abstract that the type of pollutant is "isothiazolinone antimicrobials", making the research achievements more specific and intuitive.

Page 2, line 20: Anodic oxidation is a pivotally electrochemical technology for wastewater treatment containing emerging contaminants such as isothiazolinone antimicrobials, but sluggish interfacial water-splitting kinetics for hydroxyl radical (•OH) formation and a limited oxidation range, constrain its industrial scalability.

2. Comment 2: Abstract: The manuscript mentions a 500-hour stable long-term flow operation but does not specify the pollutant removal efficiency maintained during this period. It is recommended to add this key performance parameter.

Response: We appreciate your valuable suggestions. We agree that pollutant removal efficiency is an important parameter. Our system maintained stable operation for 500 h with approximately 95% degradation efficiency. According to your comments, we have made the following revisions to the Abstract.

Page 2, line 31: Ultimately, Fe^{3+}-mediated precise tuning of the H-bond network of water molecules enables non-selective degradation of diverse emerging contaminants, delivering stable long-term flow operation of 500 hours with ~95% removal efficiency maintained for contaminant.

3. Comment 3: Line 98: Fe^{3+} is oxidized to high-valent iron (Fe(H), including Fe(IV), Fe(V), Fe(VI)). Please unify the notation of high-valent iron throughout the manuscript, and briefly clarify why Fe(VI) is classified into Fe(H) to avoid notation confusion.

Response: Many thanks for your valuable suggestions and kind reminder. Since this system generates Fe(IV), Fe(V), and Fe(VI), to make the expression more concise, we use Fe(H) as a unified representation for high-valent iron species. The definition of high-valent iron is provided on the Page 6, line 113 of the manuscript.

In the *in situ* electrochemical Raman, we conducted tests under operating potentials ranging from 1.6 V vs. SCE to 3.0 V vs. SCE, using potassium ferrate (VI) (Fe(VI)) as a reference standard (Figure S11). Within the effective voltage range, peaks corresponding to the characteristic position of Fe(VI) gradually intensified with increasing voltage (~805 cm^{-1}), confirming the presence of Fe(VI). Therefore, Fe(H) includes Fe(VI) in this system.

Additionally, PMSO is commonly used as a probe for Fe(IV)/Fe(V), which can undergo oxygen transfer with PMSO to generate $PMSO_2$. Therefore, we confirmed the presence of Fe(IV)/Fe(V) in Fe^{3+}/BDD system through PMSO probe experiments (Figure 3a and Figure S9). We demonstrated that Fe(H) in the Fe^{3+}/BDD system include Fe(IV), Fe(V), and Fe(VI) [1, 2]. However, the reaction rates of Fe(IV)/Fe(V) with pollutants are 2 to 6 orders of magnitude higher than those of Fe(VI), making them the

dominant active high-valent iron species [3]. For simplicity, we refer to these high-valent iron species collectively as Fe(H) throughout this paper, representing Fe(IV), Fe(V), and Fe(VI). Nevertheless, for detailed descriptions of specific species, we accurately distinguish between Fe(IV), Fe(V), and Fe(VI) throughout the manuscript.

Page 9, line 174: DMSO, a representative quencher for both •OH and Fe(H)[36], exhibited a slightly stronger inhibitory effect (93.9%) on pollutant degradation than MeOH (79.9%) and TBA (75.8%) in the Fe^{3+}/BDD system, indicating the involvement of Fe(H).

Page 9, line 182: However, the reaction rates of Fe(IV)/Fe(V) with pollutants are 2 to 6 orders of magnitude higher than those of Fe(VI), making them the dominant active Fe(H)[3].

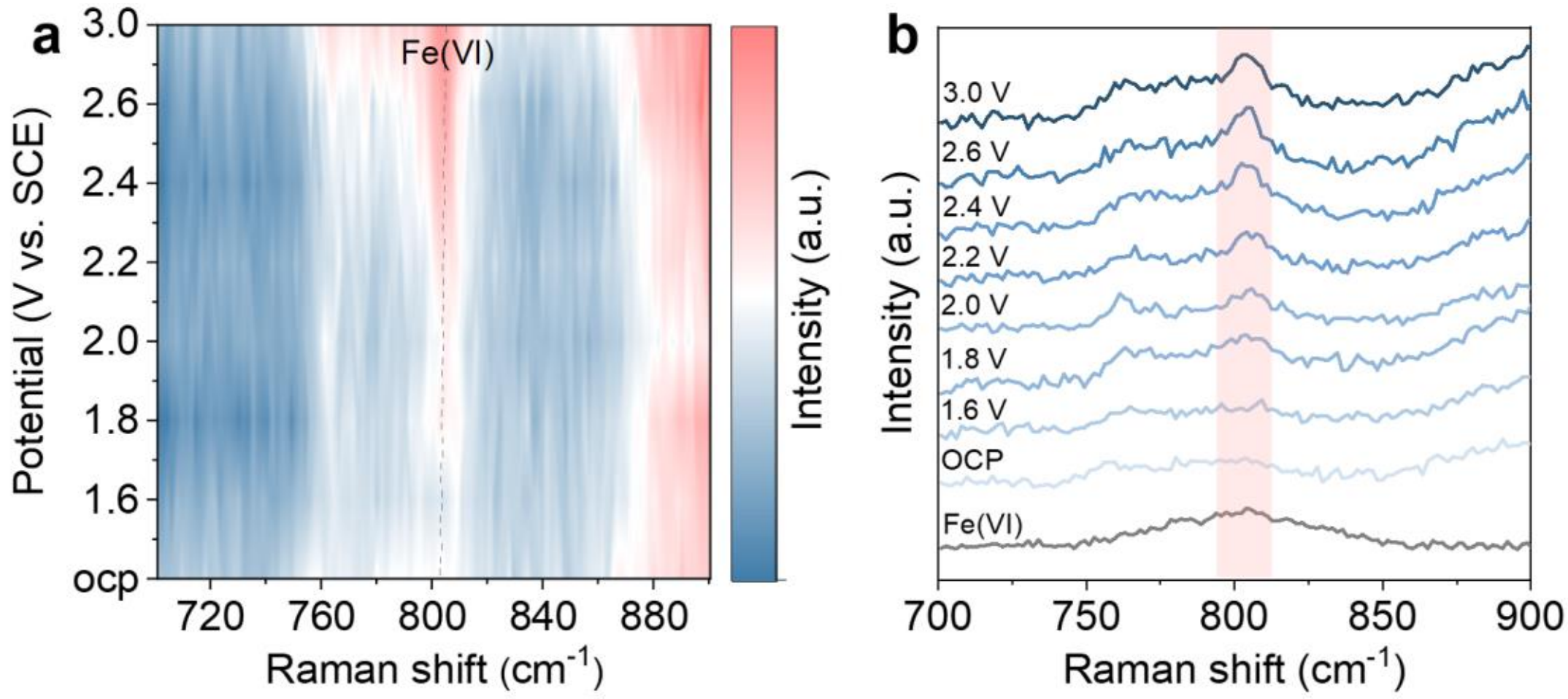

Figure S11. *In situ* electrochemical Raman spectroscopy at different potentials (700 cm^{-1} - 900 cm^{-1}). (Experimental conditions: [Na_2SO_4] = 50 mM, [Fe^{3+}] = 2 mM, [Fe(VI)] = 2 mM)

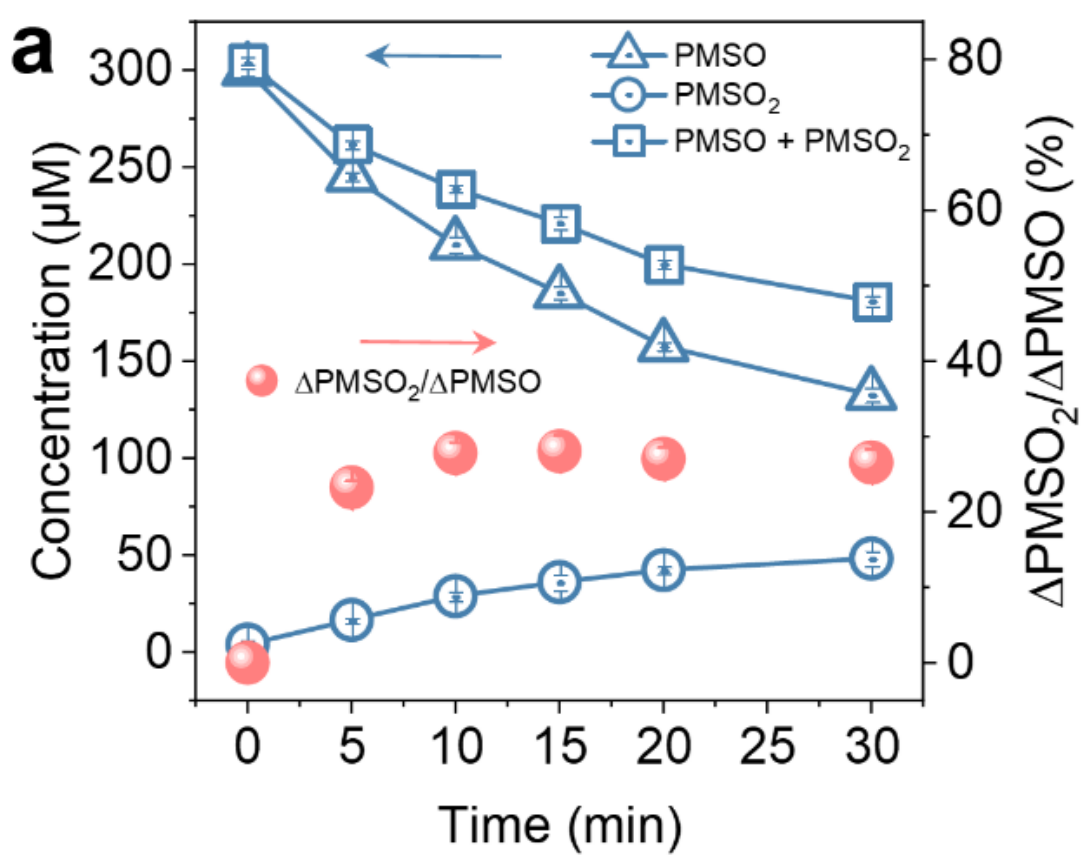

Figure 3. ***Operando* electrochemical characterization to monitor the effect of Fe^{3+} on the anodic oxidation interface. a.** Changes in PMSO consumption, $PMSO_2$ generation, and conversion rate over time.

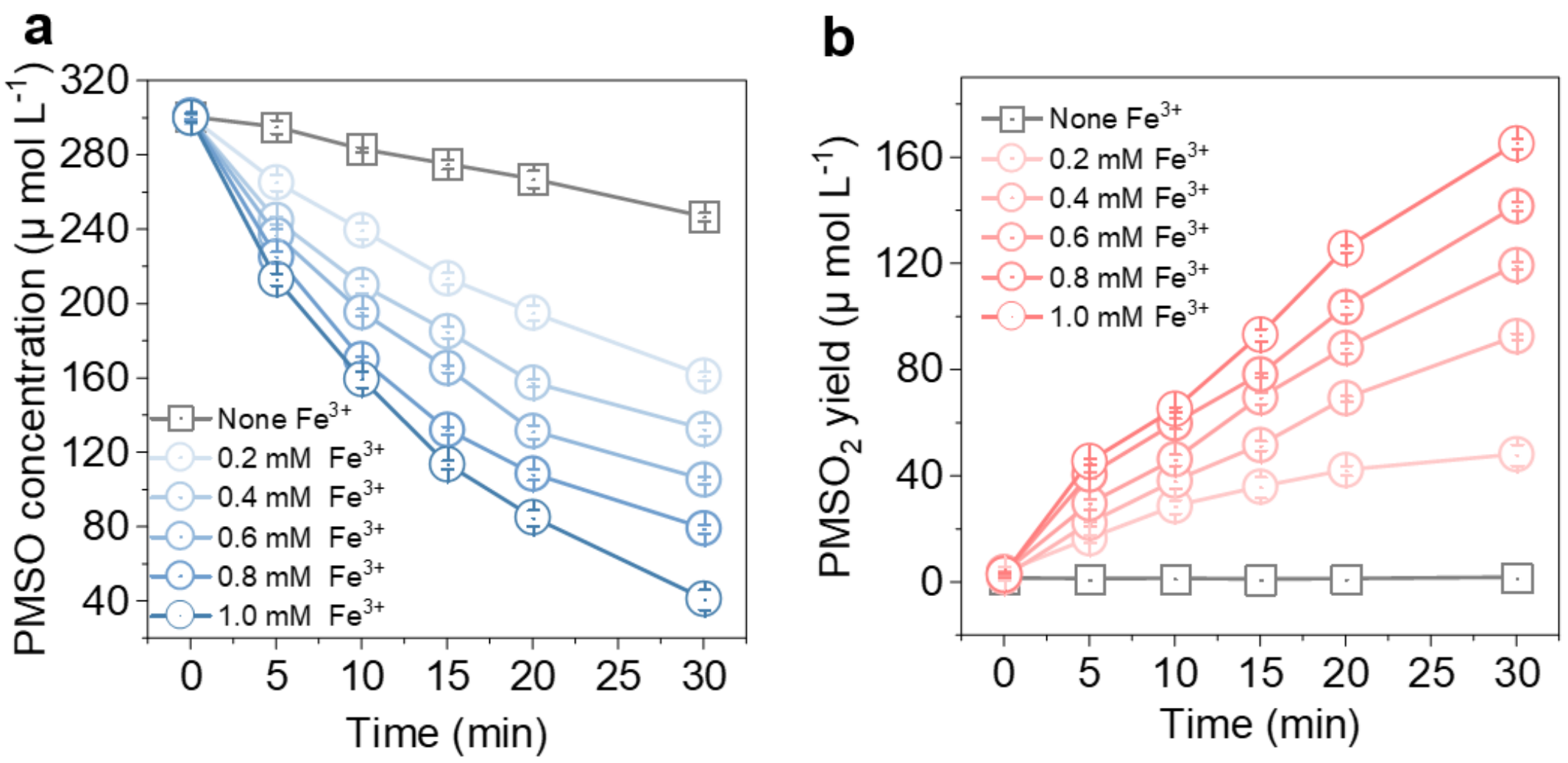

Figure S9. a. b. Effect of different Fe^{3+} concentrations on the consumption amount of PMSO and the generation amount of $PMSO_2$ in the BDD system. (Experimental conditions: [Na_2SO_4] = 50 mM, [PMSO] = 300 μM, current density = 7.5 mA cm^{-2})

4. Comment 4: Figure 2c and Table S3: The manuscript compares the pollutant normalization degradation kinetics with state-of-the-art anodic oxidation studies. It is recommended to give detailed parameters, including current density and pollutant concentration.

Response: Many thanks for your valuable suggestions and kind reminder. We previously compared the removal efficiency, removal time and k_{obs}. According to your suggestion, two more parameters of current density and pollutant concentration have been supplemented to make the comparison more comprehensive. The specific details are shown in Table S3 below.

Table S3 Catalytic performance comparison of recently typical anodic oxidation (AO) systems for pollutant removal

Catalyst/AO type	Current density (mA cm^{-2})	Pollutant concentration (μM)	Removal efficiency (%)	Reaction time (min)	k_{obs} (min^{-1})	Ref.
Fe^{3+}/BDD	7.5	20	100 (MIT)	30	0.28	This work
MW/BDD	10	452.5	91.04 (2,4-DCP)	180	0.0133	[4]
BDD/PS	10	5	100 (CBZ)	30	0.059	[5]
UV/BDD	10	50	100 (NB)	60	0.075	[6]
$SnSbO_2/Mn_xCu_{1-x}O$	10	1064	100 (BF)	60	0.038	[7]
Sb-SnO_2-F	20	200	99 (PFOS)	120	0.035	[8]
3D CuNi-ATO	5	20.8	88.4 (TC)	120	0.06	[9]
$MnCuSnSbO_2$	20	55.4	100 (LEV)	30	0.188	[10]
Ti_4O_7	10	2	98.6 (PFOS)	30	0.008	[11]
TiO_2 NCs	2.22 V_{RHE}	155.6	95.6 (4-CP)	180	0.19	[12]
Co_3O_4-TiO_2	0.4 V_{SCE}	21.9	100 (BPA)	60	0.08	[13]
$(Ti_{1-x}Ce_x)_4O_7$	20	20	98.9 (PFOS)	120	0.083	[14]
RuO_2/Fe^{2+}	10	300	100 (MeP)	180	0.016	[15]

DSA/PS	5	5	30 (BPA)	15	0.013	[16]

5. Comment 5: Line 162: •OH and Fe(H) contribute 83.5% and 13.4% to MIT removal respectively. It is suggested to briefly discuss the reason for the large difference in contribution ratios to deepen the analysis of reactive species action mechanisms.

Response: We extend our heartfelt gratitude for your careful reminder. To explain the substantial difference in removal contributions between •OH and Fe(IV)/Fe(V) during MIT degradation, we conducted a deeper analysis of the current data. Specifically, we evaluated the reaction rate constants and steady-state concentrations of these two dominant reactive species in a combined manner. The result shows that the product ($k_{obs,\ MIT}$, s^{-1}) of the second-order reaction rate constant ($M^{-1}\ s^{-1}$) corresponding to •OH and its concentration (M) is 5.5 times that of Fe(IV)/Fe(V). The detailed discussion is as follows.

(1). Reaction rate

•OH possesses an exceptionally high standard reduction potential (E^0(•OH/OH^-) = 2.70 V vs SHE) [2, 17, 18], which is significantly higher than that of high-valent iron species such as Fe(IV) (E^0(Fe(IV)/Fe(III)) = 2.0 V vs SHE) and Fe(V) (E^0(Fe(V)/Fe(IV)) = 1.8 V vs SHE) [2, 18]. Therefore, the oxidation rate of •OH with pollutants is typically several orders of magnitude higher than that of Fe(IV)/Fe(V). More importantly, the secondary rate constant of •OH with MIT ($k_{\bullet OH,\ MIT}$) is 2.09 × 10^9 M^{-1} s^{-1} [19]. Therefore, we supplemented competitive kinetic experiments on MIT and PMSO after completely quenching •OH by adding excess MeOH in the Fe^{3+}/BDD system, and obtained $k_{Fe(IV)/Fe(V),\ MIT}$ = 1.08 × 10^3 M^{-1} s^{-1} (Text S8 and Figure S14). The results indicate that the $k_{\bullet OH,\ MIT}$ is 6 orders of magnitude higher than $k_{Fe(V)/Fe(IV),\ MIT}$, demonstrating that •OH possesses an absolute kinetic advantage. In addition, the reaction rate constants of Fe(V)/Fe(IV) with many emerging pollutants are approximately 10^1–10^4 M^{-1} s^{-1} [2], and our calculated results fall within a reasonable range.

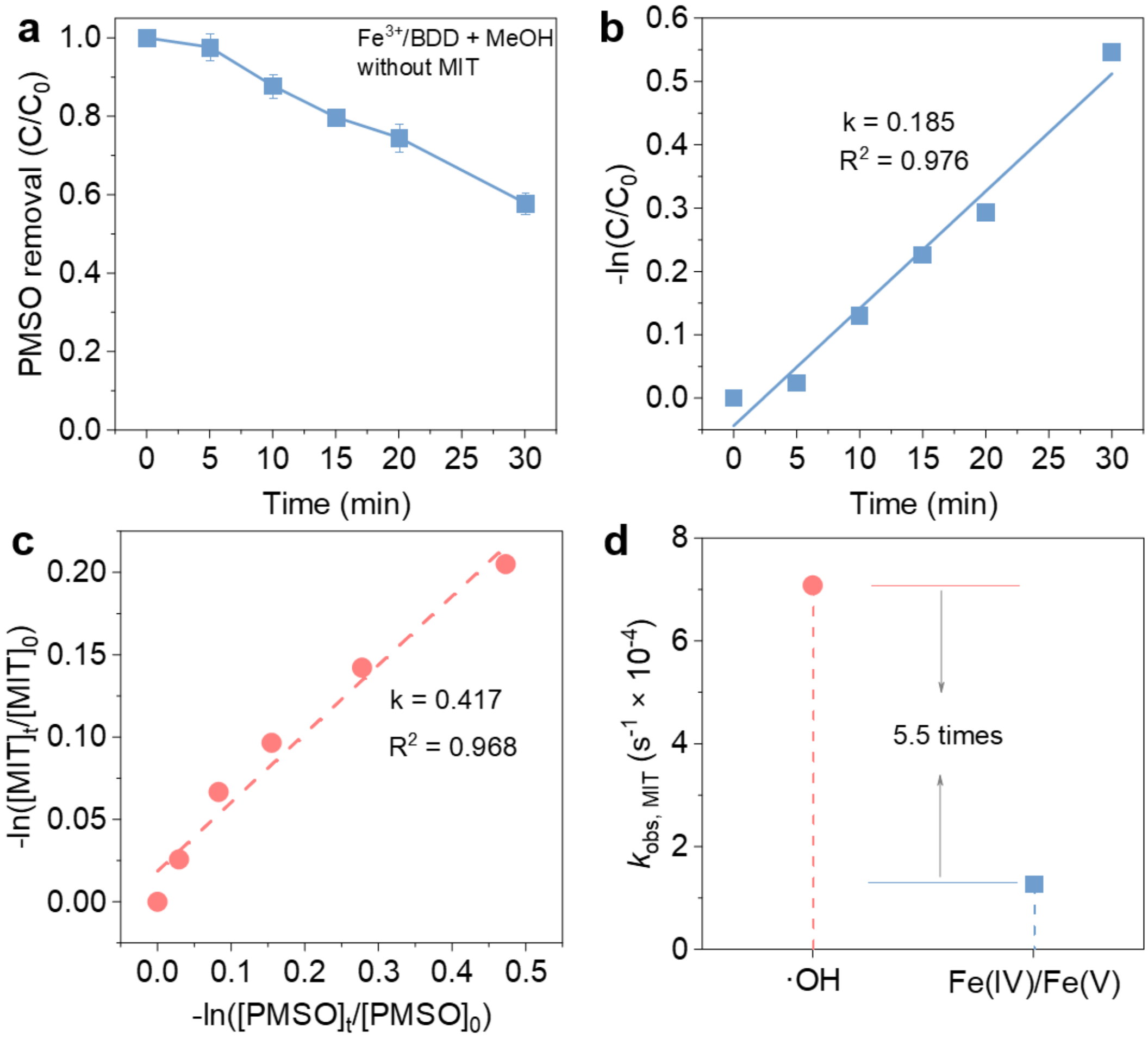

Figure S14. a. and b. Degradation of PMSO in the Fe^{3+}/BDD system during MeOH quenching and its corresponding first-order kinetic curves. **c.** Linear fitting of $-\ln([MIT]_t/[MIT]_0)$ and $-\ln([PMSO]_t/[PMSO]_0)$ obtained from the MeOH quenching process in the Fe^{3+}/BDD system with MIT and PMSO at the same concentration. **d.** The $k_{obs,MIT}$ of •OH and Fe(IV)/Fe(V) in the Fe^{3+}/BDD system. (Experimental conditions: $[Na_2SO_4]$ = 50 mM, [MIT] = [PMSO] =20 μM, $[Fe^{3+}]$ = 0.2 mM, [MeOH] = 1000 mM, current density = 7.5 mA cm^{-2})

(2). Concentration of reactive species

The concentrations of •OH and Fe(IV)/Fe(V) calculated based on the results of the NB probe experiment and the PMSO probe experiment were 3.39×10^{-13} M and 1.20×10^{-7} M, respectively (Figure 2f, Text S7, Text S8 and Figure S14). Due to the extremely short half-life of •OH, capturing it is highly challenging, which may result in relatively low measured concentrations. The •OH captured by NB probe experiments represents the average concentration in the bulk phase, whereas COMSOL simulation

results show local concentrations at the interface (Figure 2h). Therefore, a certain discrepancy between the two is entirely reasonable.

In conclusion, we can obtain the $k_{obs,\ MIT}$ of •OH and Fe(IV)/Fe(V) with MIT by multiplying the second-order rate constants by the concentrations of the active species (Text S9). The results show that the $k_{obs,\ MIT}$ for •OH is 5.5 times higher when reacting with Fe(IV)/Fe(V), which is consistent with their respective contributions to MIT removal (Figure 2g).

To further accurately quantify the contributions of •OH and Fe(IV)/Fe(V), we have supplemented the experiments with a concentration gradient of MeOH and TBA (Figure S5). Using 500 mM MeOH (Figure 2d and g), we found that 500 mM MeOH reduced MIT degradation by the Fe^{3+}/BDD system from 100% to 24.2% and by the BDD-only system from 53.2% to 8.4%. These results confirm that Fe^{3+} enhances •OH generation during BDD anodic oxidation. Quantitative analysis shows •OH contributes 88.93%—the dominant reactive species in the Fe^{3+}/BDD system.

In this system, the introduction of Fe^{3+} significantly accelerates the reaction kinetics of water oxidation at the BDD anode to generate •OH, thereby greatly enhancing the production of •OH. Meanwhile, Fe^{3+}/Fe(H) cycle further promotes the dissociation of interfacial water and generating more Fe(H). The primary degradation pathway remains driven by •OH, while the Fe^{3+}/Fe(H) cycle enhances overall oxidation kinetics and broadens the degradation range. In summary, we have made the following additions in the revised manuscript.

Page 9, line 186: Collectively, •OH and Fe(IV)/Fe(V) are identified as the dominant reactive species in the Fe^{3+}/BDD system.

Page 10, line 188: Quenching analysis and kinetic calculations (Figure 2g, Figure S13, and Text S6) indicates that •OH and Fe(IV)/Fe(V) together dominate pollutant degradation, contributing 88.9% and 8.4%, respectively, whereas direct electron transfer plays a negligible role (2.7%).

Page 10, line 191: According to literature and experimental results, the second-order reaction rate constant of •OH with MIT ($k_{\bullet OH,\ MIT}$ = $2.09 \times 10^{9}\ M^{-1}\ s^{-1}$) is significantly higher than that of Fe(V)/Fe(IV) ($k_{Fe(V)/Fe(IV),\ MIT}$ = $1.08 \times 10^{3}\ M^{-1}\ s^{-1}$) (Text

S8 and Figure S14) [19], but the steady-state concentration of •OH (3.39×10^{-13} M) is lower than that of Fe(V)/Fe(IV) (1.20×10^{-7} M) (Text S7). Therefore, the $k_{obs,\,MIT}$ of •OH is 5.5 times that of Fe(V)/Fe(IV) (Text S9 and Figure S14), consistent with their relative contributions to MIT removal.

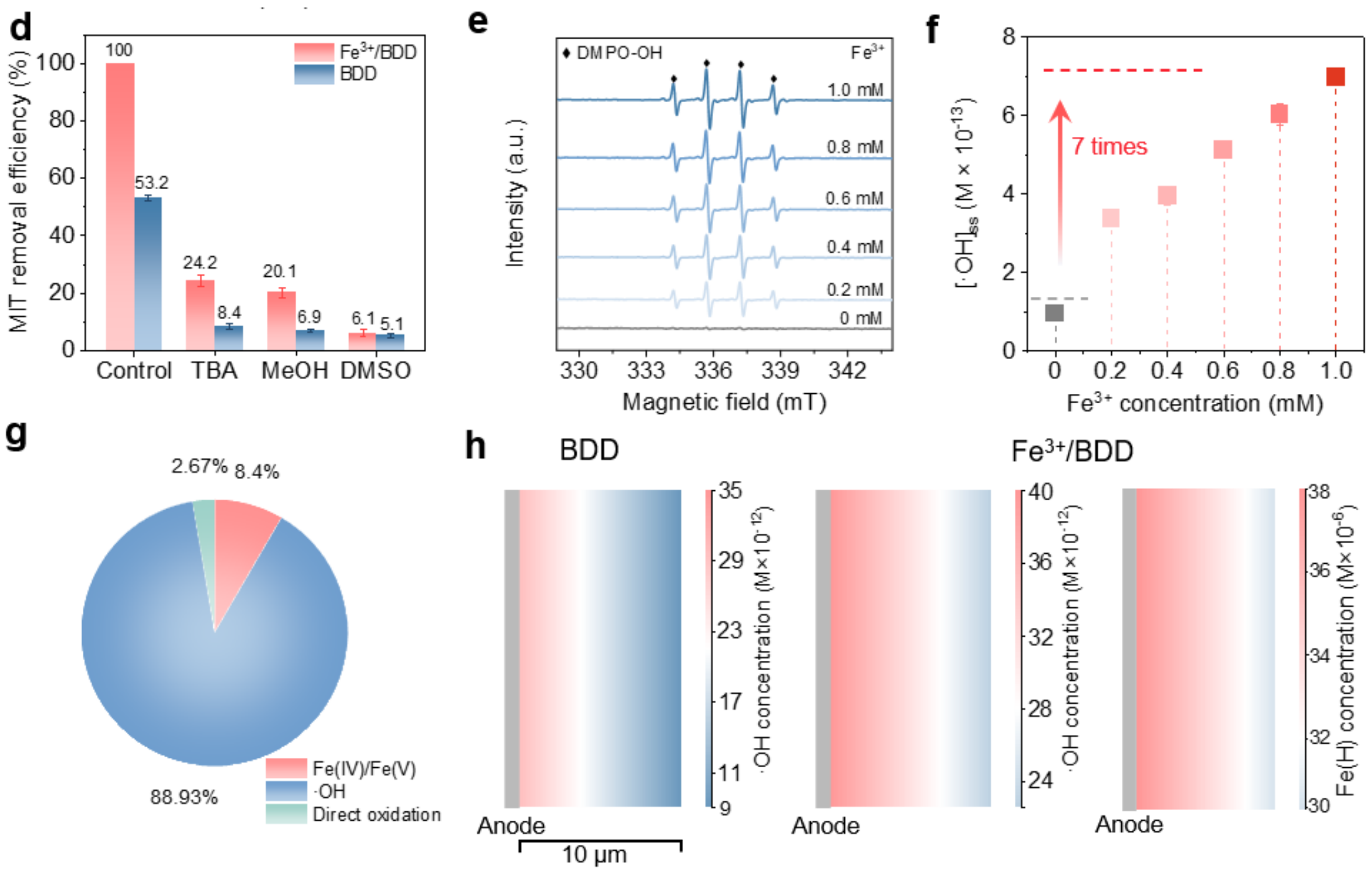

Figure 2. Pollutant degradation and identification of dominant reactive species in the Fe^{3+}/BDD system. d. Effect of scavenger (TBA, MeOH and DMSO) on the removal of MIT in the BDD and Fe^{3+}/BDD systems. **e.** Effect of Fe^{3+} concentration on the DMPO-OH signal intensity in the EPR test. **f.** Effect of Fe^{3+} concentration on the generated concentration of •OH in Fe^{3+}/BDD. **g.** Relative contributions of reactive species to MIT removal in the Fe^{3+}/BDD system. **h.** Concentration distribution of •OH and Fe(IV)/Fe(V) at the anode interface were simulated using COMSOL in BDD and Fe^{3+}/BDD systems. (Experimental conditions: current density = 7.5 mA cm^{-2}, [Na_2SO_4] = 50 mM, [MIT] = 20 μM, [DMPO] = 20 μM, [TBA] = [MeOH] = 200 mM, [DMSO] = 50 mM, [Fe^{3+}] = 0.2 mM)

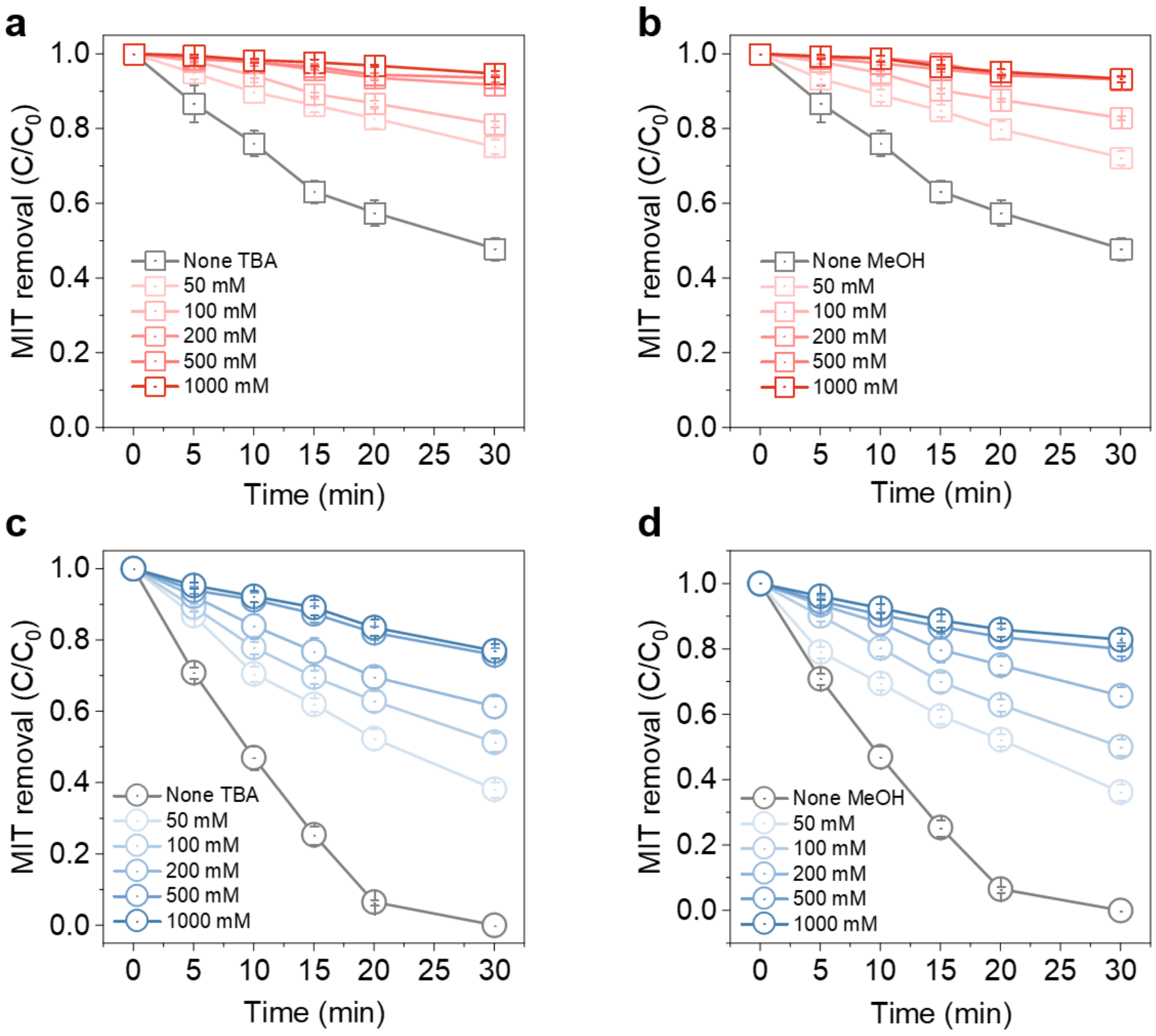

Figure S5. a and b. Effect of different TBA and MeOH concentrations on the attenuation of MIT in the BDD system. **c and d.** Effect of different TBA and MeOH concentrations on the attenuation of MIT in the BDD system. (Experimental conditions: [Na_2SO_4] = 50 mM, [MIT] = 20 μM, [Fe^{3+}] = 0.2 mM, current density = 7.5 mA cm^{-2})

6. Comment 6: Line 182: The authors describe the COMSOL simulation of the concentration distribution of •OH and Fe(H). It is suggested to supplement the key simulation parameters in the Supporting Information, and briefly explain the selection basis of these parameters.

Response: We thank you for the constructive suggestion to provide the key simulation parameters and their selection basis. In the revised manuscript, we have supplemented Text S10 that lists the key parameters for the COMSOL Multiphysics simulations. We supplement the Text S10 of the supporting information as follows.

Text S10: The concentration distribution of •OH and Fe(H) on the surface of the BDD electrode was simulated using COMSOL Multiphysics 6.2 software. A two-dimensional geometry "Transient" module along with a "Cube Current Distribution" module were employed to address the material distribution issue on the BDD electrode surface[20, 21]. The dynamic simulation of the reaction process was conducted utilizing

the Butler-Volmer and Nernst-Planck equations[22]. The electrode double layer thickness was set to approximately 10 μm, which is a typical steady-state Nernst electrical double layer for a non-stirred system. The bulk concentration of Fe^{3+} was set to the experimentally optimized value of 0.2 mM. The current density is set at 7.5 mA cm^{-2}. The diffusion coefficient of •OH in aqueous solution was set to about 2.0×10^{-9} $m^2\ s^{-1}$, which is an approximation commonly adopted in electrochemical models for small molecules. For the high valent iron species, a slightly lower value of about 0.6×10^{-9} $m^2\ s^{-1}$ has been used. This choice accounts for the larger size of the Fe(H) compared to the •OH.

7. Comment 7: Line 300: The manuscript classifies interfacial water into 4-HB-H_2O, 2-HB-H_2O and free H_2O. It is recommended to explain the Raman characteristic peak positions corresponding to each type of water, to make the spectral analysis more accessible for readers who are not familiar with interfacial water characterization.

Response: We extend our heartfelt gratitude for your careful reminder. In Figure 4d, we have marked the corresponding positions for the three water hydrogen bond structures, including 4-HB-H_2O (~3200 cm^{-1}), 2-HB-H_2O (~3400 cm^{-1}) and free H_2O (~3600 cm^{-1}), and provided detailed explanations in the figure caption.

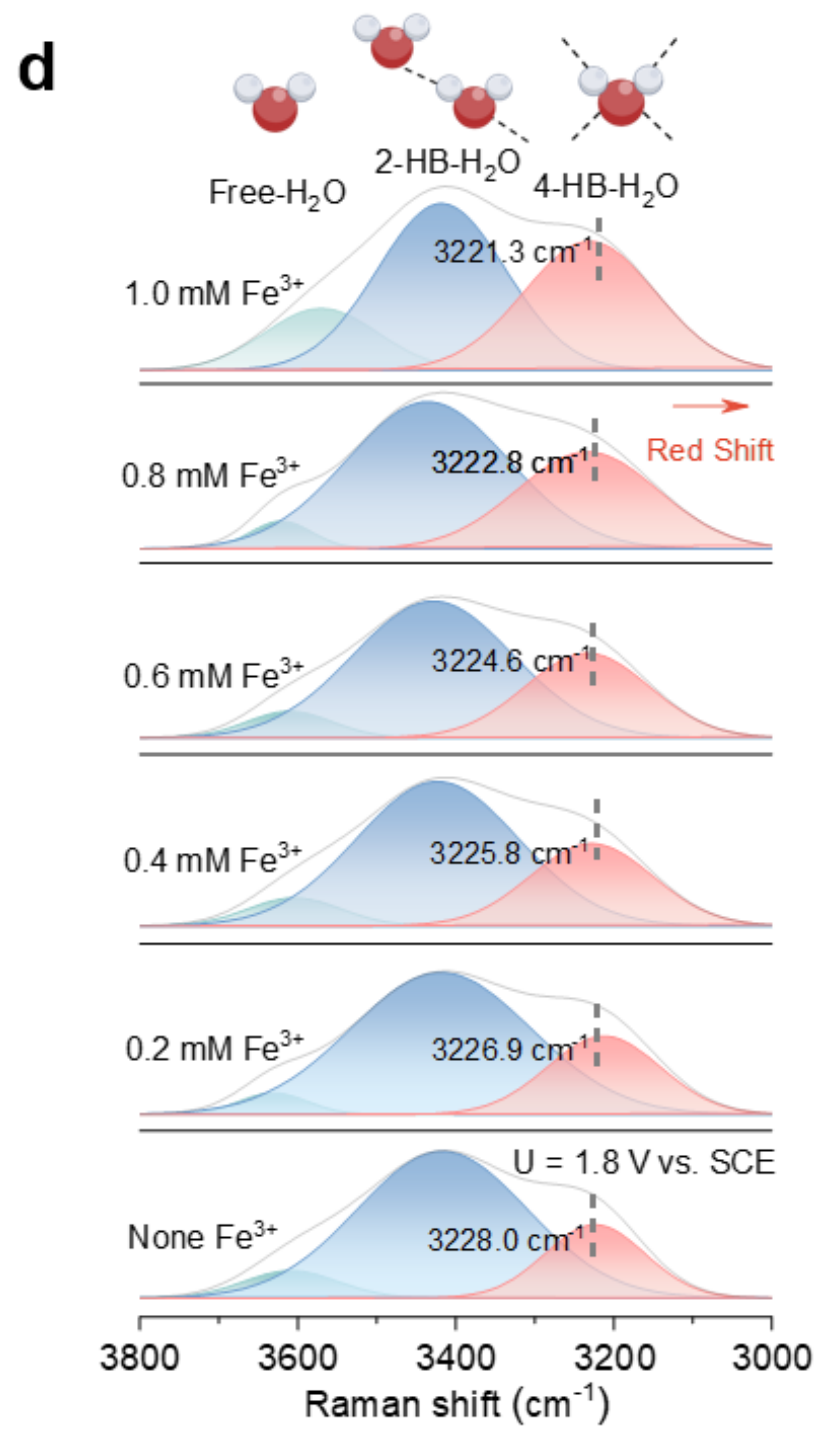

Figure 4. *In situ* spectroscopic analysis of hydrogen-bond network in the Fe^{3+}/BDD system. d. *In situ* Raman spectra at 1.8 V vs. SCE with Fe^{3+} concentrations ranging from 0 to 1 mM. (4-HB-H_2O: ~3200 cm^{-1}, 2-HB-H_2O: ~3400 cm^{-1} free H_2O: ~3600 cm^{-1})

8. Comment 8: Line 371: Figure 5f shows that Fe^{3+} inhibits the formation of H_2O_2 from •OH dimerization. It is recommended to add the detection limit of the H_2O_2 test method, to clarify the reliability of the low-concentration H_2O_2 data.

Response : Thank you for your suggestion. To validate the reliability of the H_2O_2 measurements, we provide the following explanations and supplements.

H_2O_2 was quantified using the potassium titanium(IV) oxalate spectrophotometric method, which is based on the formation of a stable yellow-orange complex (peroxotitanic acid) between Ti(IV) and H_2O_2 in acidic medium, with absorbance measured at 410 nm [23]. This method is widely used for H_2O_2 determination in advanced oxidation systems due to its simplicity, rapidity, and minimal interference from common coexisting species. The detection capability of this method has been well established in the literature.

Following your recommendation, we have supplemented the calibration curve of the potassium titanate oxalate method under specific experimental conditions (Figure R1). We established a primary calibration curve for H_2O_2 (2–100 μM), showing excellent linearity between concentration and UV absorbance ($R^2 > 0.99$, $p < 0.05$), fully covering the experimental range in Figure 5f (2–78 μM). A separate low-concentration curve (0–10 μM) also showed strong linearity ($R^2 > 0.96$, $p < 0.05$). This confirms the reliability of this method for quantifying H_2O_2 within this concentration range. All H_2O_2 concentration measurements were performed in triplicate. The relative standard deviations (RSD) were consistently less than 5%, demonstrating excellent analytical reproducibility. The linear correlation between absorbance and H_2O_2 concentration remained excellent even at the lowest concentrations within our calibration range.

Then, we measured the absorbance of 10 blank samples and calculated the standard deviation (SD_{blank}) as 0.001. Based on the standard curve in Figure R1b and LOD = 3 × SD_{blank}/k, the detection limit of this method was determined to be 2 μM.

Therefore, we measured the H_2O_2 concentration entirely within the effective range of this method.

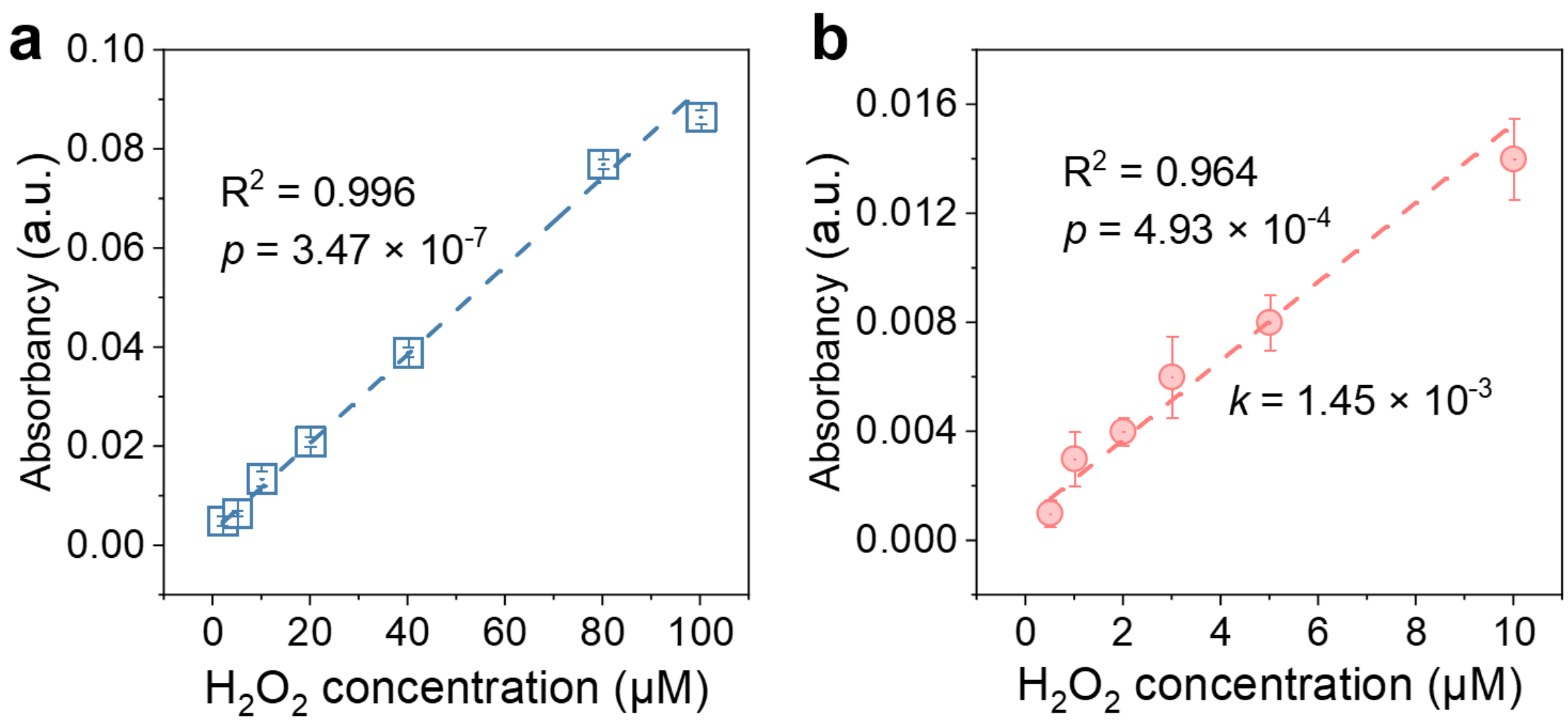

Figure R1. a. Standard curve for the detection of H_2O_2 concentration by potassium titanium oxalate method (2 μM to 100 μM). **b.** Standard curve for the detection of H_2O_2 concentration by potassium titanium oxalate method (0.5 μM to 10 μM).

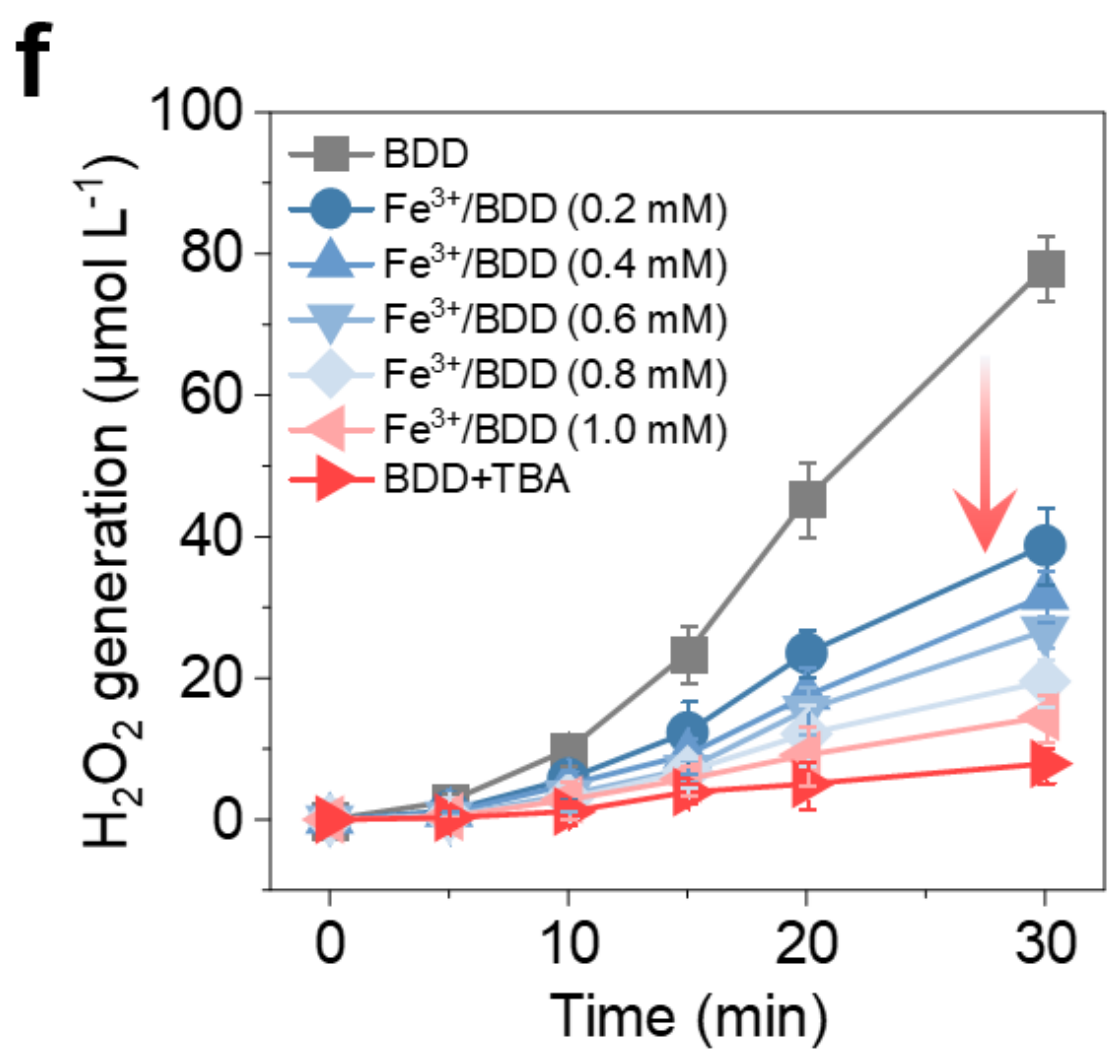

Figure 5. AIMD simulation of H-bond network reconfiguration and anodic oxidation mechanisms in Fe^{3+}/BDD system. f. H_2O_2 concentration during AO processes. (Experimental conditions: current density = 7.5 mA cm^{-2}, [Na_2SO_4] = 50 mM, [TBA] = 200 mM)

9. Comment 9: Line 418: The authors investigate the effect of typical anions and humic acid on MIT degradation. It is suggested to add the concentration range of this pollutant in actual wastewater in the discussion, to better reflect the practical application value of the Fe^{3+}/BDD system.

Response: We appreciate your very thought-provoking questions. We have noted that in the reverse osmosis system of wastewater treatment and reuse processes, 1-10 mg/L of MIT is usually added to prevent membrane fouling [24, 25]. Literature reports indicate that MIT concentrations in industrial wastewater and reverse osmosis concentrate can reach nearly 200 mg·L^{-1}, while concentrations in municipal sewage and surface water may be as low as ~μg·L^{-1} [26, 27, 28]. Therefore, the MIT concentration used in this study was 2.3 mg/L, which was at a moderate level. The Fe^{3+}/BDD system can optimize its treatment performance by adjusting the electrode area, current and Fe^{3+} concentration when treating wastewater from different scenarios. We made the following additions in the manuscript.

Page 28, line 519: The concentration range of MIT in actual sewage is quite wide, ranging from ~μg/L to nearly 200 mg/L [26, 27]. Here, a MIT concentration at a moderate level (2.3 mg/L) was selected.

Reviewer #2 (Remarks to the Author):

General comments: This manuscript presents a thoughtfully designed and conceptually appealing study on reconfiguring interfacial hydrogen-bond (H-bond) networks via Fe^{3+} to enhance anodic oxidation for wastewater treatment. The integration of electrochemical analysis, in situ spectroscopy, and theoretical simulations provides a relatively comprehensive framework linking electrolyte engineering to catalytic performance. The manuscript is clearly written, logically structured, and the results are, in general, convincing. The proposed strategy offers a potentially impactful paradigm for advancing electrochemical water treatment technologies with practical relevance.

That said, given the growing interest in interfacial water regulation and electrolyte effects in electrocatalysis, several aspects of the mechanistic interpretation would benefit from further clarification or additional discussion to meet the expectations of a high-impact readership. In particular, some key claims rely on indirect evidence and would be strengthened by more rigorous justification. Overall, I recommend minor revision, provided the authors address the following points:

Response: We sincerely appreciate your positive comments on the originality, novelty and scientific significance of our manuscript. We supplemented control experiments, different initial pH experiments, 2D DOSY NMR tests, Fe^{3+} concentration monitoring, universality investigations, and established a linear relationship between •OH concentration and 4-HB-H_2O, providing a more detailed explanation of the mechanism by which Fe^{3+} mediate enhanced •OH generation. These kind suggestions are all accepted and carefully addressed as below. The corresponding revisions have also been made in the manuscript accordingly.

Specific Comments:

1. Comment 1: In the Abstract, the statement that 4-HB-H_2O motifs increase by 16% is central to the mechanistic claim. The authors are encouraged to briefly indicate the baseline value (e.g., initial percentage) to improve clarity and allow immediate contextual interpretation.

Response: We extend our heartfelt gratitude to you for the very considerate comment. We have revised the Abstract to explicitly state the baseline value of the 4-HB-H_2O motifs. Specifically, the original statement "4-HB-H_2O motifs increase by 16%" has been modified to "4-HB-H_2O motifs increase from 21% to 37%". This provides immediate contextual interpretation without ambiguity. The corresponding sentence in the Abstract has been updated accordingly.

Page 2, line 24: This study leverages Fe^{3+} to strengthen interfacial water hydrogen bond, accelerating •OH formation and high-valent iron production, thus enhancing oxidation kinetics and scope. $[Fe(OH)(H_2O)_5]^{2+}$ enhances the hydrogen-bond (H-bond) population at the electrode surface—particularly 4-HB-H_2O motifs increase from 21% to 37% and weakens O–H bonds, due to its strong hydration and polarization ability.

2. Comment 2: In the Results and Discussion, 0.2 mM Fe^{3+} is selected as the optimal concentration, although 1 mM exhibits a higher k_{obs}. The authors should elaborate on the practical trade-offs (e.g., chemical consumption, possible sludge formation, or diminishing energy efficiency) that justify this selection.

Response: We extend our heartfelt gratitude to you for the very considerate comment. After the addition of 0.2 mM Fe^{3+}, the k_{obs} for the removal of MIT increased by 3.5 times and it could be completely removed within 30 minutes. Although the addition of 0.2 mM Fe^{3+} increased the chemical reagent cost by 0.0097 US$/$m^3$ and the post-treatment cost by 0.0114 US$/$m^3$, the electricity cost was reduced from 5.81 US$/$m^3$ to 2.87 US$/$m^3$. Overall, this significantly reduced the total cost of sewage treatment by 2.94 US$/$m^3$. Finally, considering the balance of performance, energy consumption and cost, we ultimately chose a Fe^{3+} dosage of 0.2 mM. We have supplemented the following explanation in the revised manuscript.

Page 8, line 140: The 0.2 mM Fe^{3+} is selected over 1 mM to enhance degradation kinetics while reducing chemical use and iron sludge—avoiding reduced energy efficiency and secondary pollution at higher concentrations.

3. Comment 3: Identification of high-valent iron species is important mechanistic point but not clear in the manuscript. In Fig. 3a and related discussion, the formation of Fe(IV), Fe(V), and Fe(VI) is proposed. Given the central role of these species, the

authors should clarify which high-valent iron species predominantly contributes to pollutant oxidation. Additionally, it would strengthen the manuscript to discuss the limitations of current evidence (e.g., Raman assignment, probe reactions) and whether more definitive techniques (e.g., XAS or Mössbauer spectroscopy) could further validate these assignments.

Response: We appreciate your constructive suggestion. We have conducted a detailed analysis of the evidence and roles of Fe(IV), Fe(V), and Fe(VI) as follows.

(1). Identification and roles of high-valent iron species

Based on the PMSO probe experiment (Figure 3a, Figure S9 and S10), we confirmed the presence of Fe(IV)/Fe(V). And based on *in situ* Raman (Figure S11), we confirmed the existence of Fe(VI). All these were directly or indirectly proved through experiments that the Fe(IV), Fe(V), and Fe(VI) all exist in the system. However, due to the limitations of existing probe methods, it is still difficult to quantitatively distinguish the contributions of each. Therefore, we assess their effects from the perspective of reaction kinetics. Existing literature indicates that the reaction rates of Fe(IV) and Fe(V) with organic pollutants are typically 2 to 6 orders of magnitude higher than that of Fe(VI) [3]. Thus, Fe(IV)/Fe(V) are more likely to be the main high-valent iron active species in the oxidation process of pollutants [1].

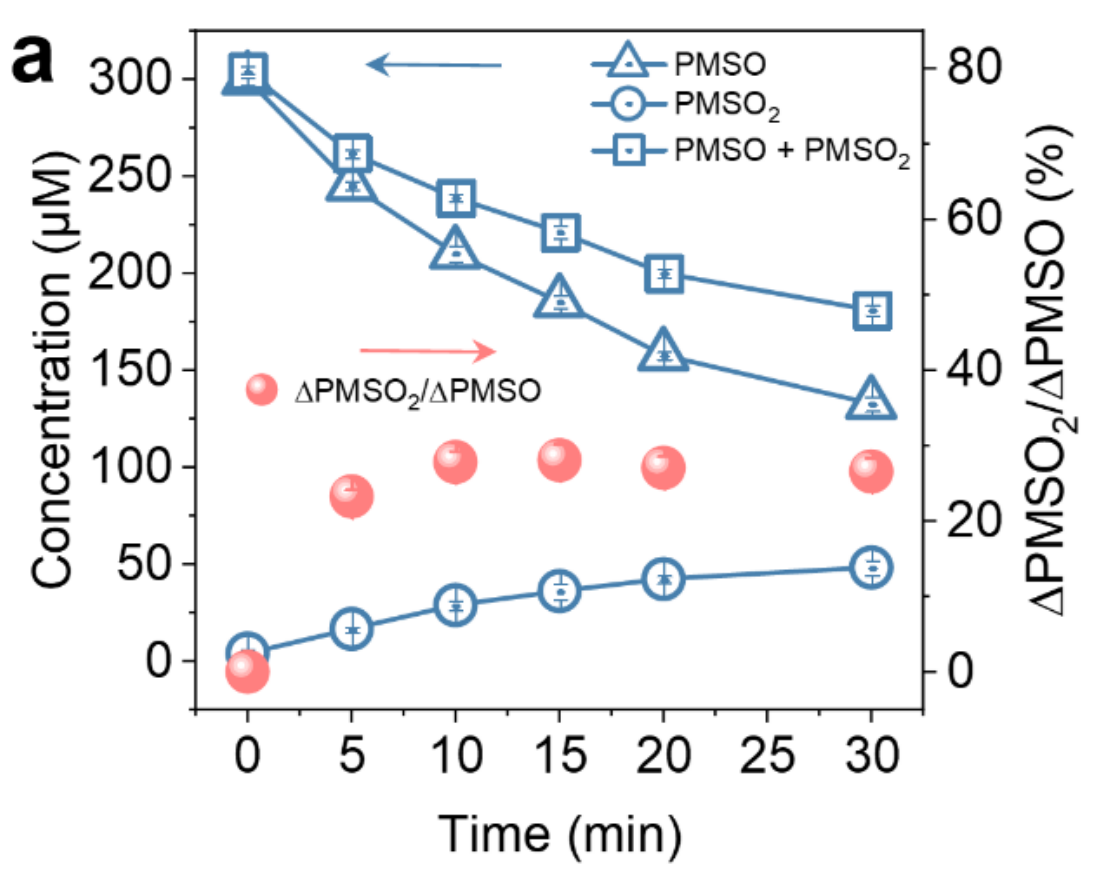

Figure 3. ***Operando*** **electrochemical characterization to monitor the effect of** $\mathbf{Fe^{3+}}$ **on the anodic oxidation interface. a.** Changes in PMSO consumption, $PMSO_2$ generation, and conversion rate over time.

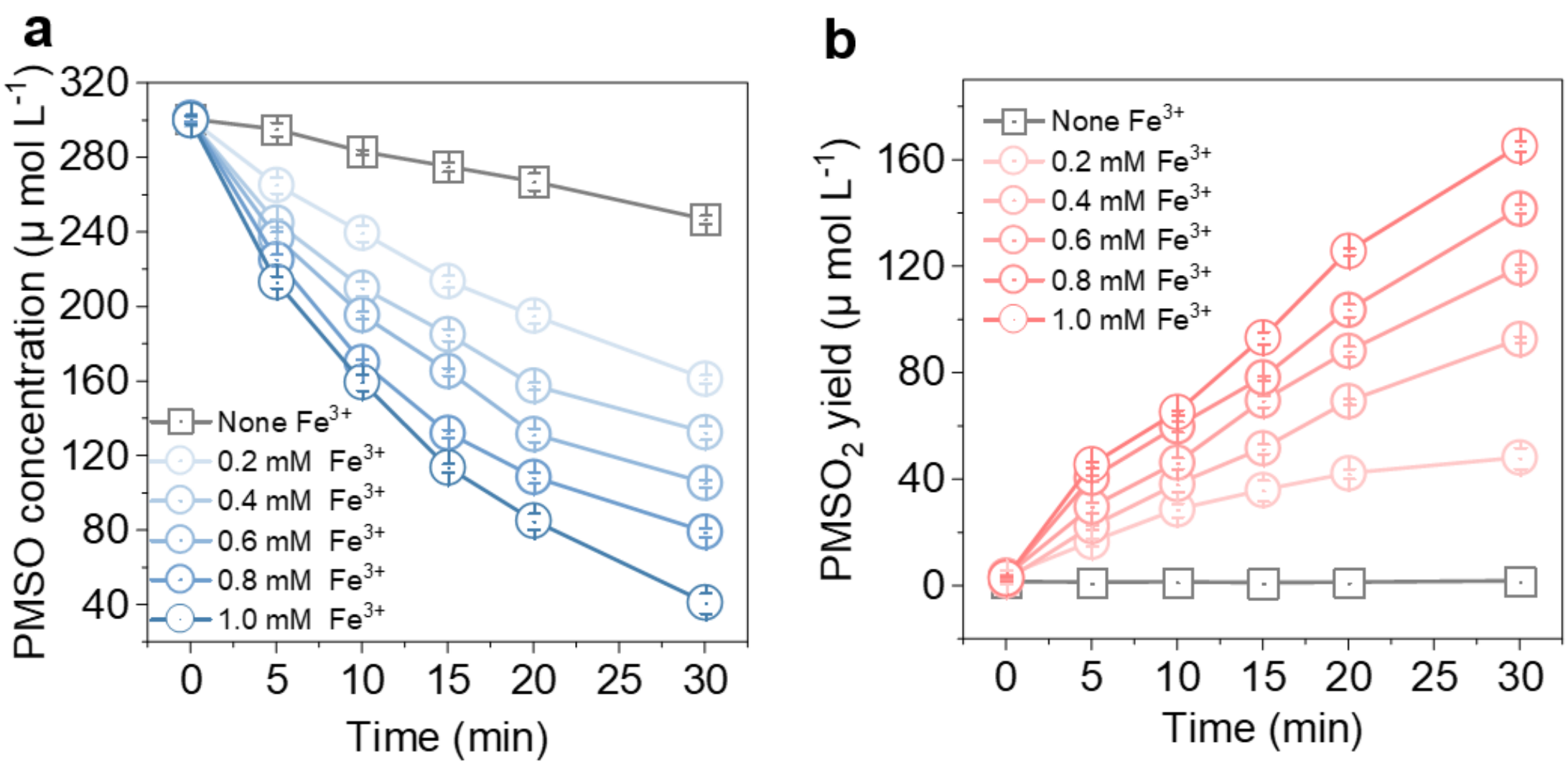

Figure S9. a. b. Effect of different Fe^{3+} concentrations on the consumption amount of PMSO and the generation amount of $PMSO_2$ in the BDD system. (Experimental conditions: [Na_2SO_4] = 50 mM, [PMSO] = 300 μM, current density = 7.5 mA cm^{-2})

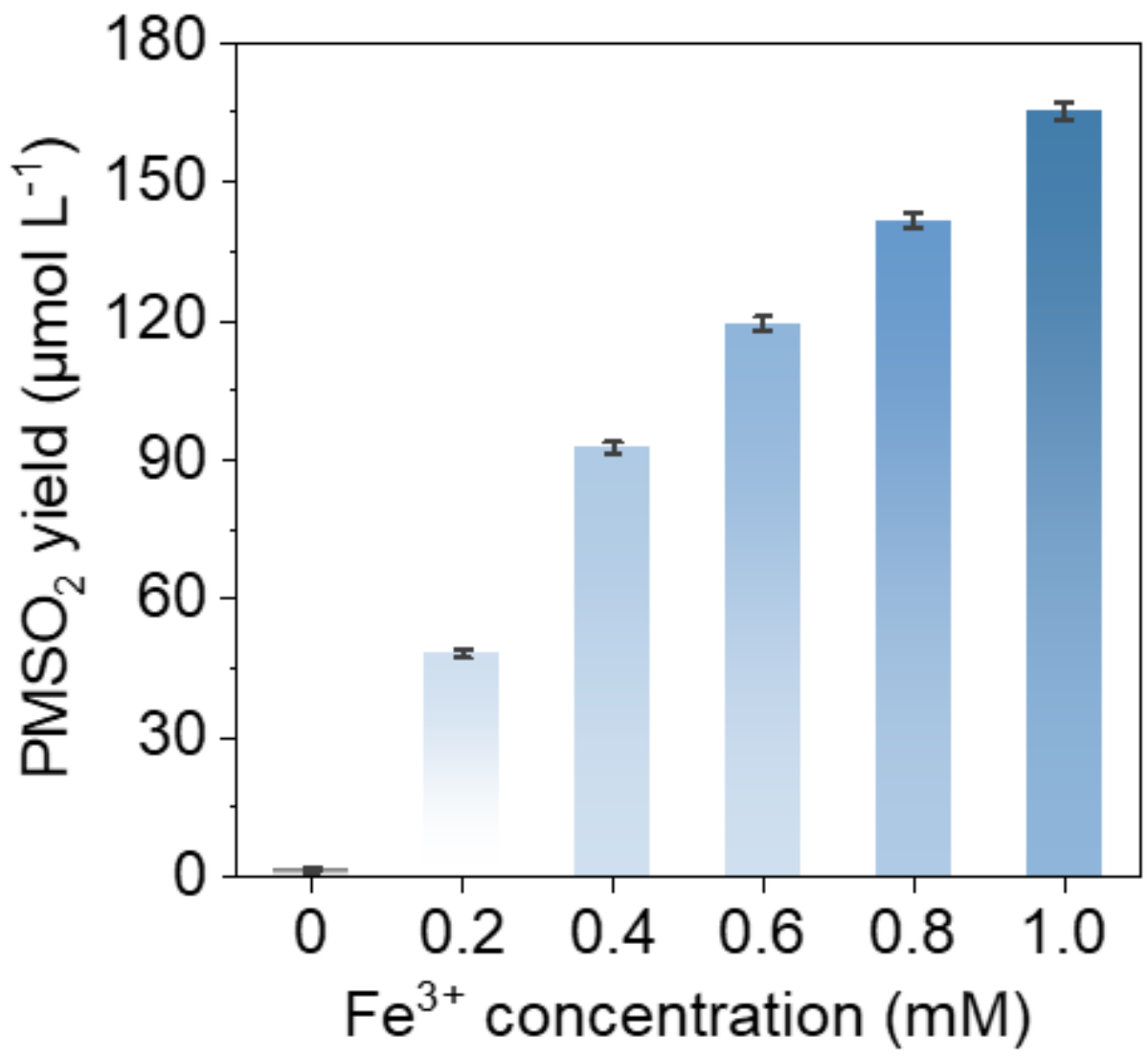

Figure S10. The amount of $PMSO_2$ generated corresponding to different Fe^{3+} concentrations. (Experimental conditions: [Na_2SO_4] = 50 mM, [PMSO] = 300 μM, current density = 7.5 mA cm^{-2})

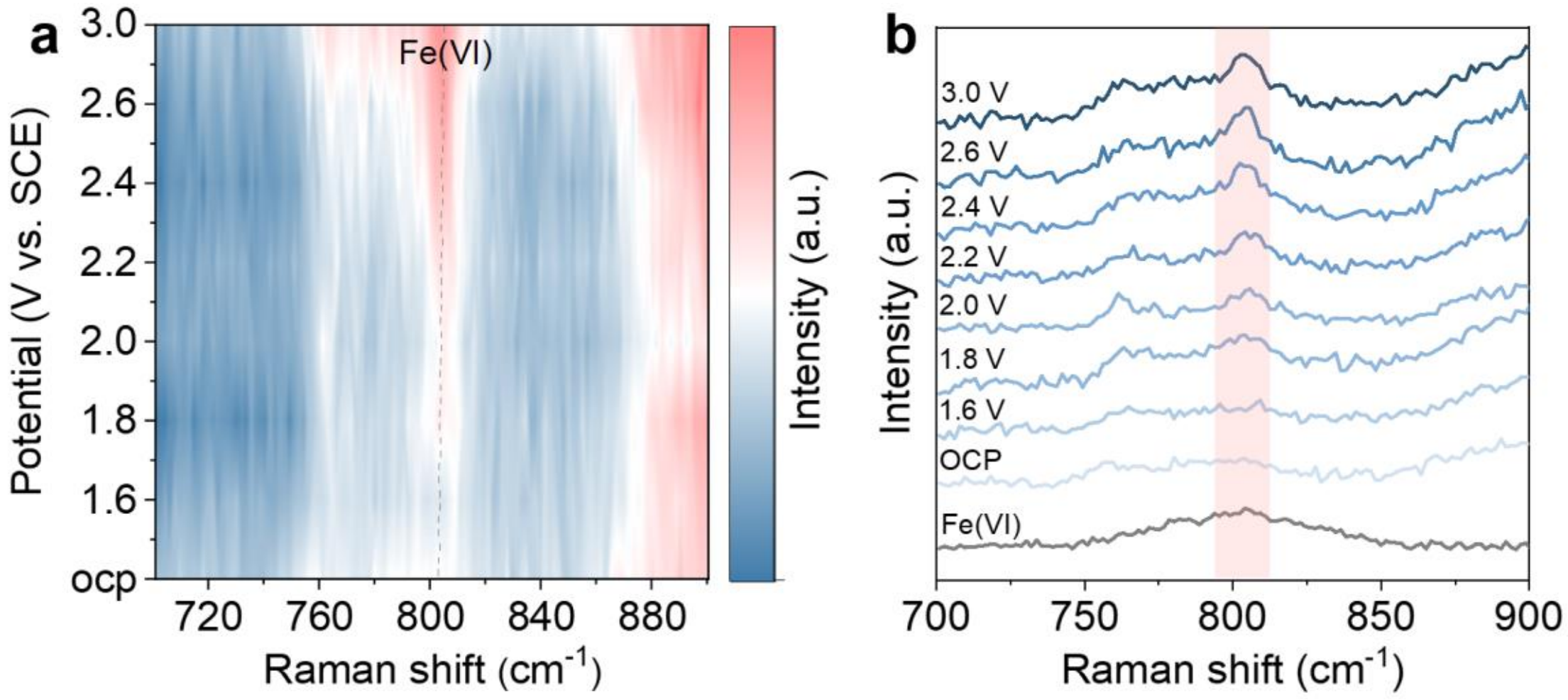

Figure S11. *In situ* electrochemical Raman spectroscopy at different potentials (700 cm^{-1} - 900 cm^{-1}). (Experimental conditions: [Na_2SO_4] = 50 mM, [Fe^{3+}] = 2 mM, [Fe(VI)] = 2 mM)

(2). XAS and Mössbauer

We appreciate your suggestion. The XAS or Mössbauer spectroscopy can indeed provide more direct evidence for identifying heterogeneous high-valent iron species. However, in this system, Fe(IV), Fe(V), and Fe(VI) are difficult to be directly captured under the current conditions. It should be noted that the core mechanism of this study is not high-valent iron-dominated oxidation, but rather the Fe^{3+}-induced reconstruction of the interfacial H-bond network, which promotes water oxidation to generate •OH, and it is •OH that mainly drives the MIT degradation. In contrast, the contribution of high-valent iron species to MIT removal is only about 8.7%, playing an auxiliary role. Although *in situ* Raman identification and probe reactions have limitations, they remain the most commonly used and effective methods currently available. In our future research, we will focus on attempting to characterize the formation and transformation of high-valent iron species using XAS or Mössbauer spectroscopy.

4. Comment 4: In Figure 4a, the non-monotonic chemical shift with increasing Fe^{3+} concentration is interesting. Please clarify why the paramagnetic effect becomes dominant only above ~0.4 mM Fe^{3+}, and whether this threshold is consistent with known Fe^{3+} hydration or clustering behavior.

Response: Many thanks for your valuable suggestions and kind reminder. We have reclarified the relationship between hydrogen bonding effects and paramagnetic effects on the shielding and deshielding of hydrogen nuclei, and supplemented the 2D DOSY NMR data. The changes in the DOSY diffusion coefficients further support the formation of a more tightly packed H-bond network around water molecules coordinated to Fe^{3+}. We have made detailed additions and revised the manuscript accordingly.

Page 16, line 306: First, low concentrations of Fe^{3+} forcibly strip water molecules to form the local hydration shells (0.2–0.4 mM). Although local hydrogen bonds are formed, this solvation process disrupts the original bulk water H-bond network. The strong local H-bond network formed by the Fe^{3+} hydrated sphere ($[Fe(OH)(H_2O)_5]^{2+}$) is masked by paramagnetic effects, causing the overall NMR signal to appear as dispersed bulk water shifted toward upfield. As concentration increases (0.4–2 mM), more Fe^{3+} hydrated spheres form, and the strong electron-withdrawing effect within these spheres propagates through chemical bonds to the surface coordinated water molecules, weakening their O-H bonds. This allows the coordinated water molecules to form tighter H-bond network with outer water molecules, shifting the NMR signal toward downfield. Additionally, due to the paramagnetism of Fe^{3+}, water exchange within the hydrated spheres becomes slow, and multiple Fe^{3+} paramagnetic centers accumulate under the strong H-bond network. According to nuclear magnetic relaxation theory, this leads to a shortened transverse relaxation time, resulting in broadening of the NMR peak shapes.

Additionally, 2D DOSY NMR also indicates the rigidity of this strong H-bond network (Figure S21). After adding Fe^{3+}, the diffusion coefficient decreases, indicating slower water exchange and the formation of a more tightly bound H-bond network [29].

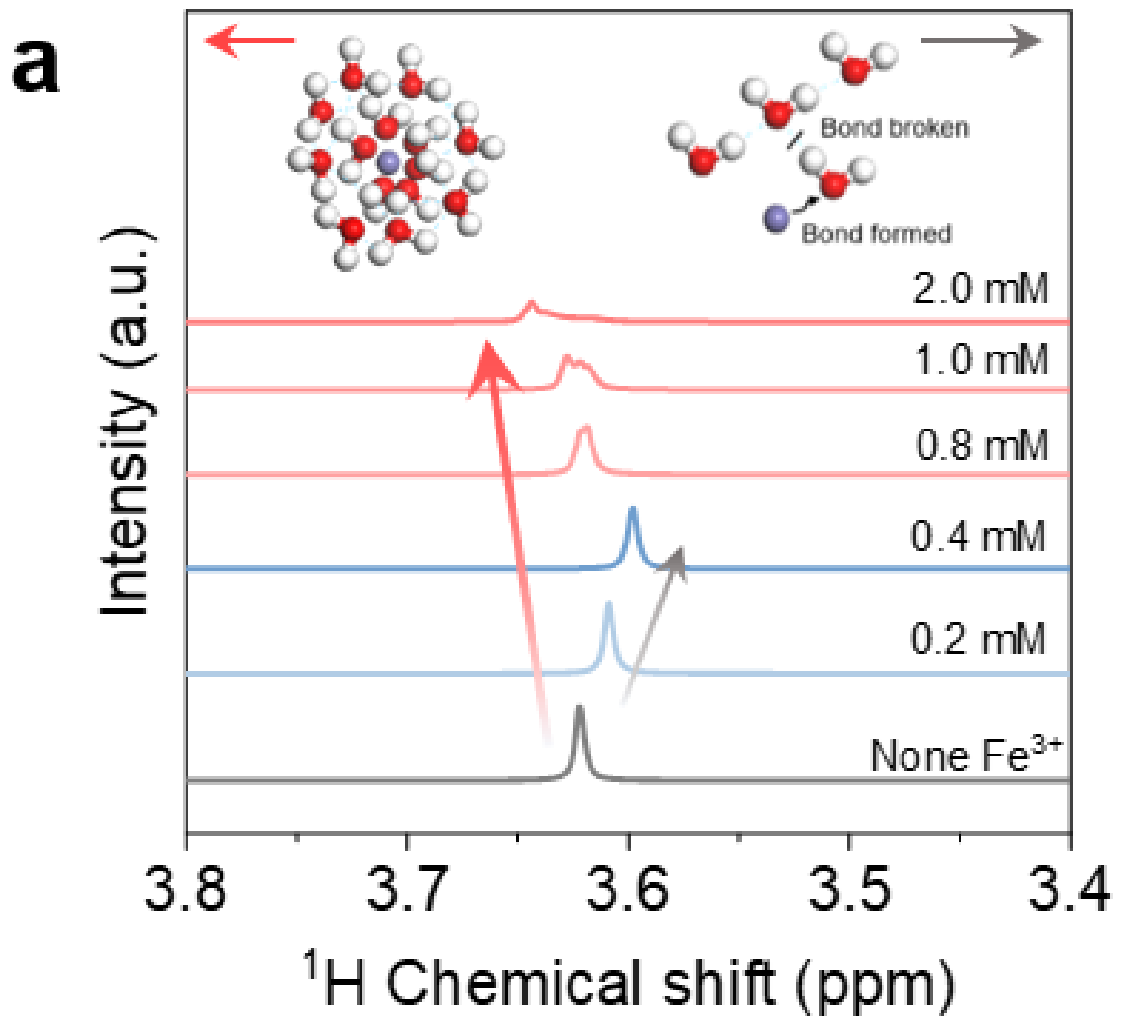

Figure 4. *In situ* spectroscopic analysis of hydrogen-bond network in the Fe^{3+}/BDD system. a. ^{1}H NMR chemical shifts for water at different Fe^{3+} concentration.

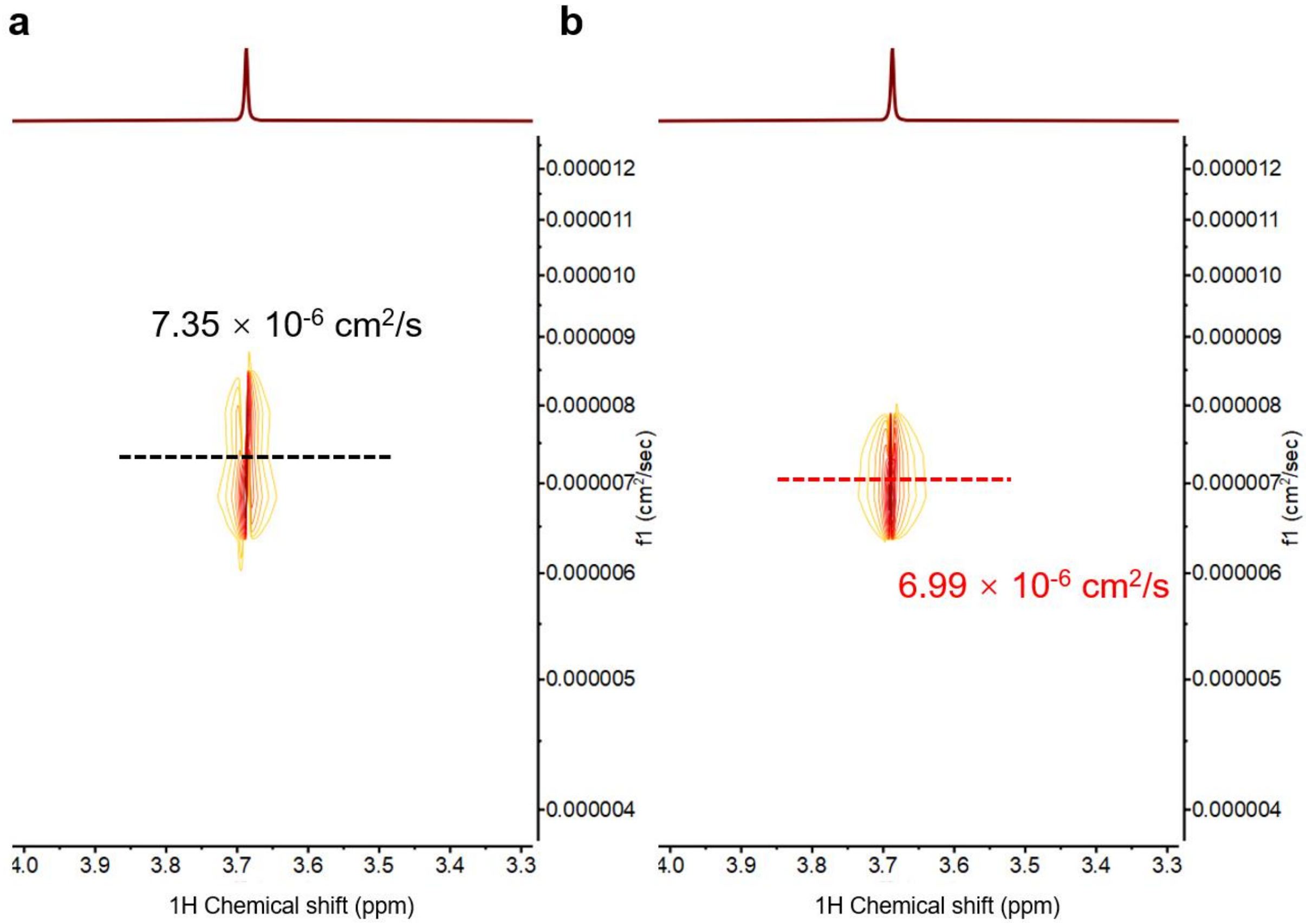

Figure S21. a. and b. 2D DOSY NMR of H_2O/DMSO solutions without and with Fe^{3+} addition.

5. Comment 5: In Figure 5f, the decrease in H_2O_2 concentration is attributed to suppressed •OH dimerization. The authors are encouraged to consider and

experimentally evaluate whether Fe^{3+} (or Fe(H) species) could also directly decompose H_2O_2, to exclude alternative pathways.

Response: We are grateful for your detailed suggestions. We conducted corresponding control experiments of Fe^{3+}/H_2O_2, Fe(VI)/H_2O_2 and Fe(VI)/Fe^{3+}/H_2O_2. The results proved that under the conditions of this study, neither Fe^{3+} nor Fe(VI) decomposes H_2O_2, further confirming our proposed viewpoint that the decrease in H_2O_2 concentration is attributed to the suppression of •OH dimerization. The specific evidence and explanations are as follows.

The result presented in detail as Figure S25, a control experiment was conducted at an initial pH of approximately 3 without an applied potential. In this experiment, the concentration of Fe^{3+} was 0.2 mM, the addition of Fe(VI) was 2 mg/L, and the concentration of H_2O_2 was 0.08 mM (consistent with the highest H_2O_2 concentration in Figure 5f). Within 30 minutes, among the three systems, only the system with Fe(VI) added showed a degradation effect of less than 20% on MIT. Moreover, the concentration of H_2O_2 remained largely unchanged in all three systems. This evidence is sufficient to demonstrate that Fe^{3+} and high-valent iron species cannot generate active oxidants that can significantly decompose H_2O_2 (Figure S25b).

These results collectively demonstrate that the decrease in H_2O_2 concentration observed in Figure 5f is primarily attributable to suppressed •OH dimerization rather than direct decomposition by Fe^{3+} or Fe(H). We add the following content to the manuscript.

Page 24, line 457: When the pH is approximately 3, Fe^{3+} and Fe(H) cannot effectively activate H_2O_2 to generate strong oxidizing active species. The removal efficiencies of MIT in the Fe^{3+}/H_2O_2, Fe(VI)/H_2O_2 and Fe(VI)/Fe^{3+}/H_2O_2 systems are all less than 20%, and the H_2O_2 concentration did not decline within 30 minutes (Figure S25).

Page 24, line 461: In addition, during the degradation of MIT by the Fe^{3+}/BDD system, no significant consumption of the by-product H_2O_2 was observed within 5 minutes after the power supply was cut off (Figure S26). This indicates that in Fe^{3+}/BDD system, there are no other pathways for the consumption of H_2O_2.

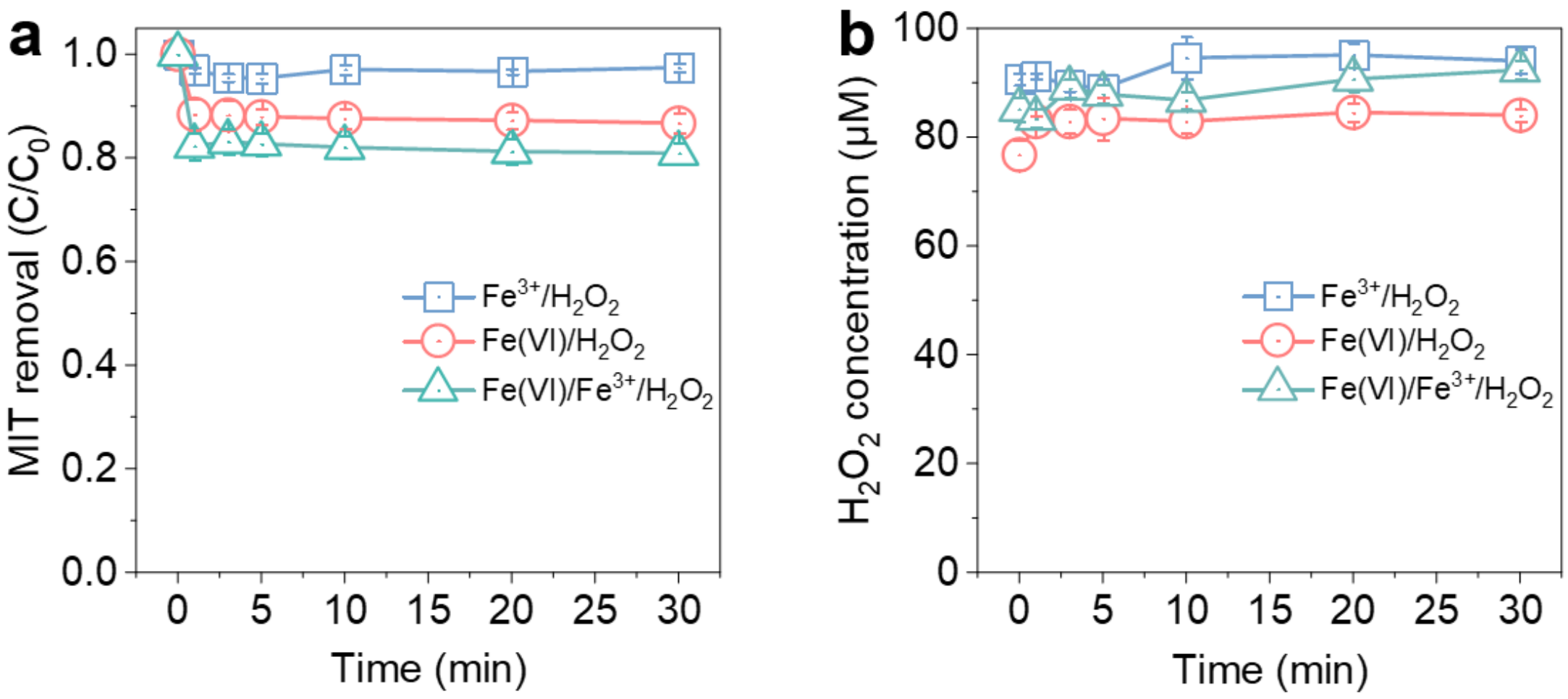

Figure S25. a. The removal effect of MIT by Fe^{3+}/H_2O_2, $Fe(VI)/H_2O_2$ and $Fe(VI)/Fe^{3+}/H_2O_2$, **b.** the variation of H_2O_2 concentration with time in Fe^{3+}/H_2O_2, $Fe(VI)/H_2O_2$ and $Fe(VI)/Fe^{3+}/H_2O_2$ systems. (Experimental conditions: [MIT] = 20 μM, [Fe^{3+}] = 0.2 mM, [Fe(VI)] = 2 mg/L, [H_2O_2] = 80 μM, initial pH = 3)

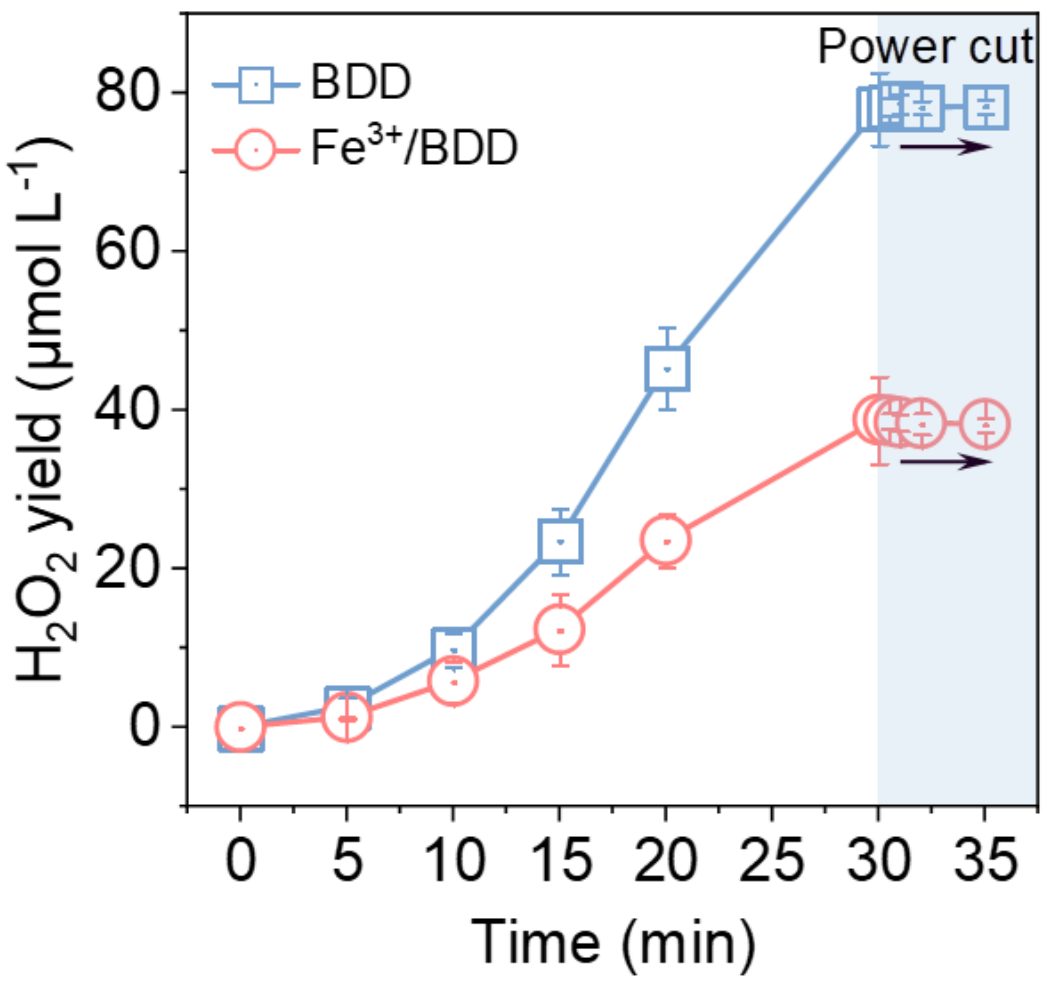

Figure S26. The continuous removal and the changes in H_2O_2 concentration after power cut. (Experimental conditions: [Na_2SO_4] = 50 mM, [Fe^{3+}] = 0.2 mM, current density = 7.5 mA cm^{-2})

6. Comment 6: The 500-h continuous operation (Figure 6d) is impressive. It would be valuable to report the Fe concentration (or speciation, if possible) during long-term operation to confirm the stability and recyclability of the Fe catalytic center.

Response: We sincerely appreciate the positive comments from you on the 500-hour continuous operation (Figure 6d). According to your suggestions, we conducted

additional experiments to evaluate the stability and recyclability of Fe^{3+}. Specifically, we monitored the total iron concentration during the long-term operation, and designed a Fe^{3+} recycle experiment to directly demonstrate the recyclability of Fe^{3+}.

(1). Stability of iron concentration during continuous operation for 500 hours

We monitored the concentration changes of Fe^{3+} under continuous flow conditions. The results shown in the Figure 6d indicate that the total iron concentration remained very stable throughout the 500-hour long-term performance, decreasing by less than 5% from the initial 0.2 mM (11.2 mg/L). This high stability is attributed to two factors: the acidic pH of the system (approximately 3), iron was mainly present in the form of $Fe(OH)^{2+}$ (Figure S1), which inhibits the hydrolysis and precipitation; and Fe^{3+} is continuously regenerated through the cycle of Fe^{3+}-Fe(H)-Fe^{3+}. Importantly, the stable iron concentration is correlated with the consistently high pollutant removal efficiency (approximately 95% throughout the 500 hours), confirming that Fe^{3+} remain active during long-term operation. We have made supplements in the revised manuscript.

Page 27, line 523: After the Fe^{3+} is oxidized to Fe(H) at the anode, its reduction product is Fe^{3+}, enabling a rapid Fe^{3+}-Fe(H)-Fe^{3+} cycle (Figure S37).

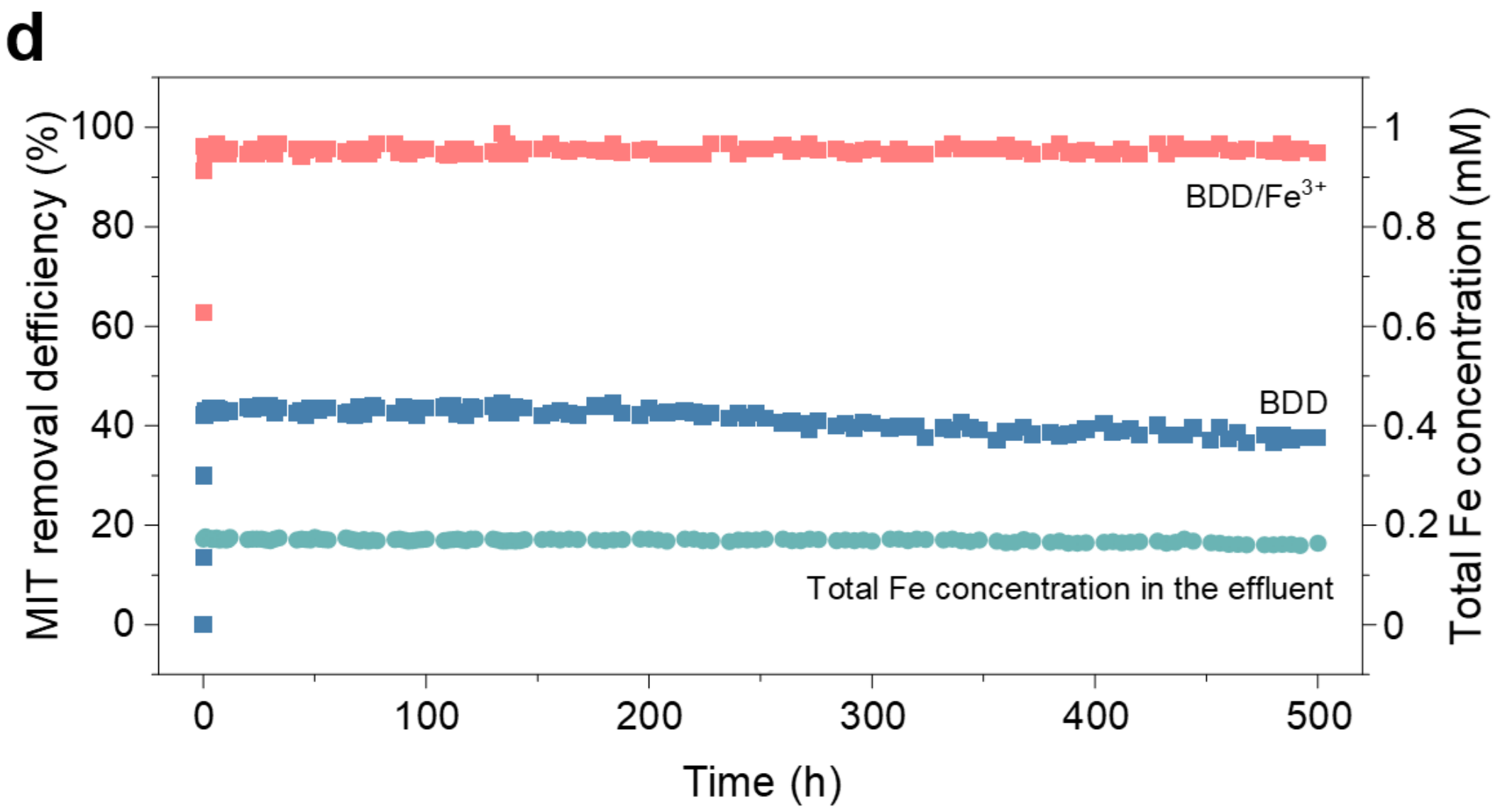

Figure 6. Practical application investigations of Fe^{3+}/BDD system. d. Stability tests of different systems.

(2). Demonstration of iron recyclability through a Fe^{3+} recycle experiment

To directly confirm that Fe^{3+} is indeed recyclable rather than continuously consumed, both fresh Fe^{3+}/BDD and recycled Fe^{3+}/BDD systems were continuously operated for 7 days (168 hours). The results (Figure R2) indicated that the recycled Fe^{3+}/BDD system maintained almost the same MIT removal efficiency throughout the 7-day period. This experiment directly indicates that Fe^{3+} is not consumed during the degradation process, continuously cycling between Fe^{3+} and Fe(H) and then returning to the initial oxidation state. The high reproducibility of the Fe^{3+} recycle experiment further confirms that Fe^{3+} remains fully active throughout the process without the need for supplementation, which is a key characteristic for practical applications.

The recyclability of Fe^{3+} demonstrated in the recycle experiment, and the 500-hour continuous operation (with approximately 95% of the contaminant removed throughout the process) collectively prove that Fe^{3+} plays a sustainable and long-lasting role in the Fe^{3+}/BDD system.

We once again thank you for this valuable suggestion, which enables us to more comprehensively demonstrate the practical feasibility of our system.

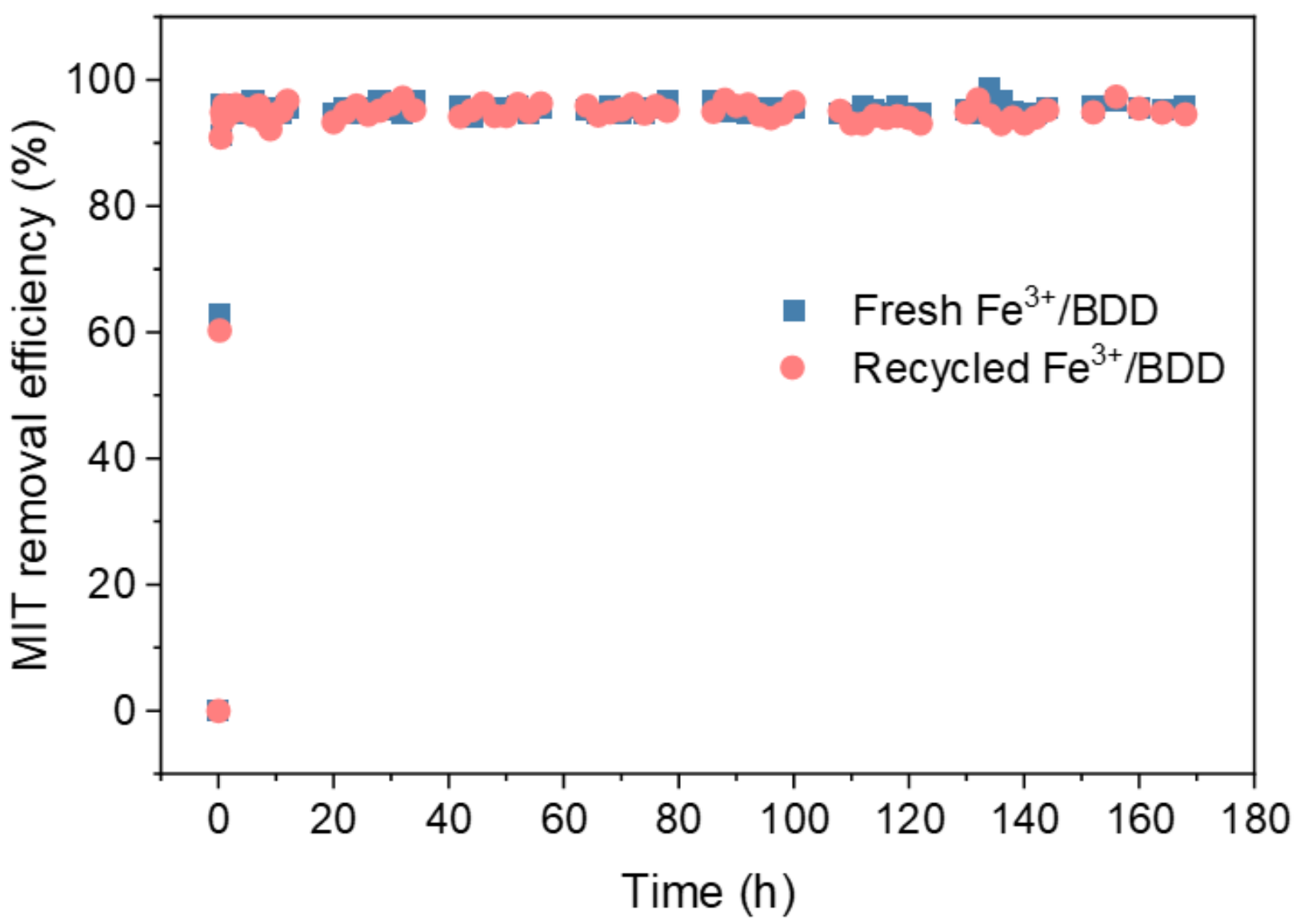

Figure R2. Stability tests of fresh Fe^{3+}/BDD systems and recycled Fe^{3+}/BDD.

7. Comment 7: The system operates at pH ≈ 3. The authors should briefly discuss the pH tolerance of the system and the implications for practical wastewater treatment, particularly regarding operation under near-neutral conditions.

Response: We appreciate for your valuable suggestion. Following your suggestion, we conducted systematic pH-dependent degradation experiments over the range of initial pH 1–10 (Figure S33). We provide the following explanation for the supplementary experiments.

The MIT degradation efficiency of the Fe^{3+}/BDD system declined progressively with increasing initial pH (1-5). At pH 1, complete degradation was achieved within 5 minutes, whereas at pH 5, complete removal was obtained after 30 min. Across this range, Fe^{3+} exists in hydrated forms such as $[Fe(H_2O)_6]^{3+}$ and $[Fe(OH)(H_2O)_5]^{2+}$. On one hand, Fe^{3+} reconstructs the H-bond network of interfacial water molecules through strong hydration, promoting the water decomposition reaction on the BDD surface to generate more •OH. On the other hand, it generates high-valent iron species under BDD anodic oxidation, providing an additional oxidation pathway. The removal rate can still maintain about 80% within 30 minutes at pH 6, which is the result of the synergistic effect of these two pathways.

Within the initial pH range of 6–10, MIT removal efficiency reached approximately 80% within 30 minutes, and the removal rate no longer decreased with increasing pH. The synergistic effect of •OH and Fe(H) enables the Fe^{3+}/BDD system to efficiently remove MIT across a wide pH range of 1–10. Within the typical pH range of most municipal wastewater and industrial effluents (pH 6–10), the degradation efficiency of the Fe^{3+}/BDD system remains at a high level, indicating that strict pH pretreatment is unnecessary. The Fe^{3+}/BDD system is a pH-tolerant technology suitable for direct treatment of real wastewater, effectively degrading MIT and other emerging organic pollutants without prior acidification. We have made supplements in the revised manuscript.

Page 26, line 510: The Fe^{3+}/BDD system achieves stable MIT degradation efficiency (more than 80% in 30 min) across pH 1–10 (Figure S36), demonstrating broad pH tolerance.

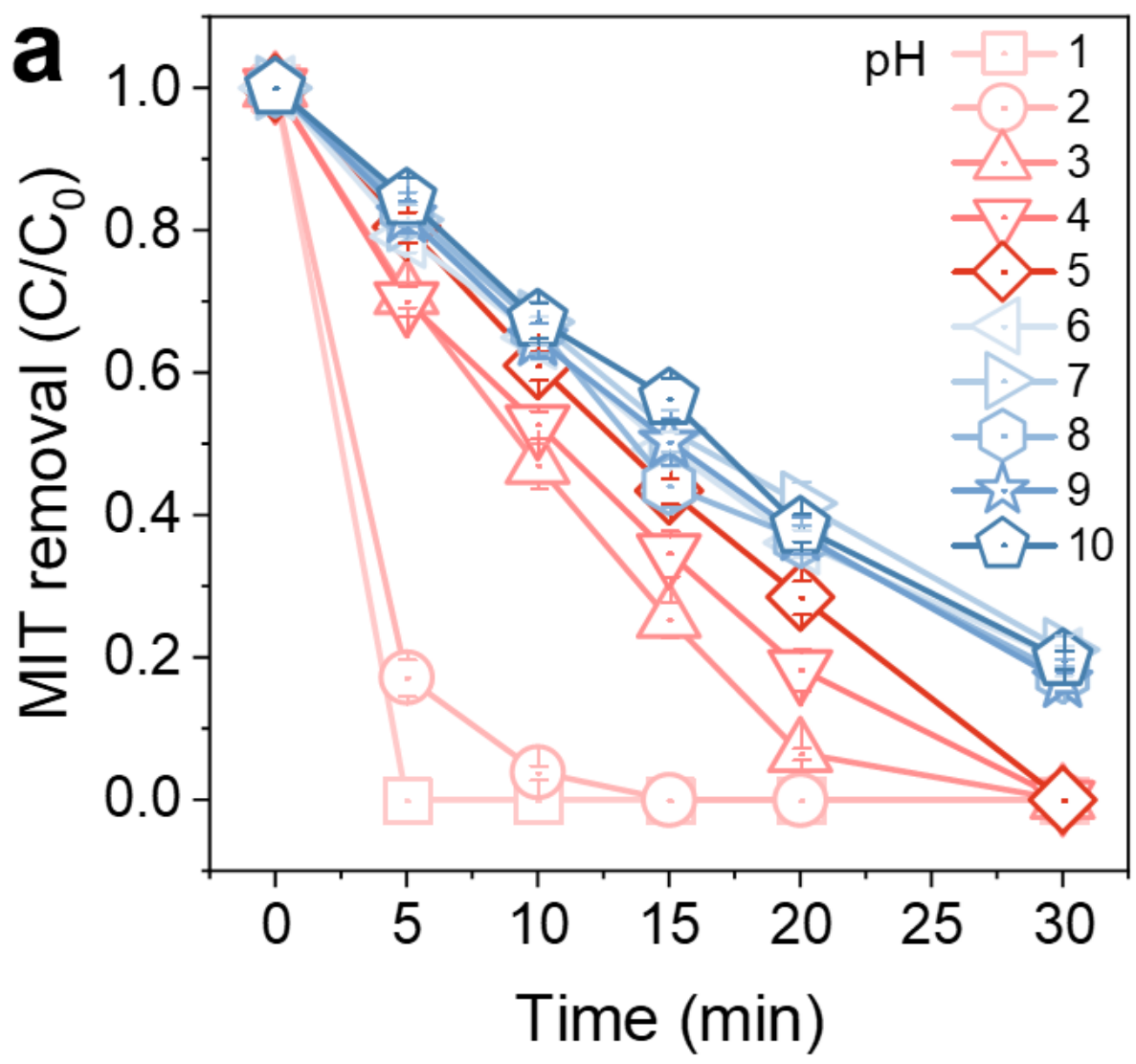

Figure S36. a. Effect of Fe^{3+}/BDD system on the removal efficiency of MIT under different initial pH conditions. (Experimental conditions: [Na_2SO_4] = 50 mM, [Fe^{3+}] = 0.2 mM, current density = 7.5 mA cm^{-2})

8. Comment 8: The study focuses on BDD electrodes. The authors are encouraged to comment on whether this Fe^{3+}-mediated H-bond network regulation strategy is transferable to other anodic materials (e.g., DSA, Ti_4O_7), which would enhance the general significance of the work.

Response: We thank you for this valuable suggestion to explore the transferability of our Fe^{3+}-mediated H-bond network regulation strategy to other anodic materials. Following your advice, we have performed additional experiments using two representative anode materials (DSA and Ti_4O_7). The results indicate that this Fe^{3+}-mediated H-bond network regulation strategy holds promise for application to other inactive anodes (such as Ti_4O_7), which has universal significance, but is ineffective on active anodes (such as DSA). The specific experimental results are supplemented as follows.

(1). DSA anode. No significant enhancement in MIT degradation was observed upon Fe^{3+} addition; performance remained comparable to that of the Fe^{3+}-free control. DSA is an active anode. On DSA, water oxidation proceeds via the formation of surface-bound oxides (e.g., IrO_x or RuO_x) that promote direct two-electron/four-electron water oxidation to O_2, and the surface-mediated mechanism leads to a very short lifetime of any •OH species and a low steady-state

concentration of free •OH. Moreover, the strong adsorption of water and intermediates on DSA surfaces likely hinders the effective oxidation of Fe^{3+} to high-valent iron species because the available active sites are occupied by water oxidation intermediates. Consequently, the Fe^{3+}-mediated H-bond regulation and high-valent iron formation are suppressed, resulting in no observable enhancement.

(2). Ti_4O_7 anode. Addition of Fe^{3+} significantly enhanced MIT degradation (similar to BDD) (49% to 81%), indicating that the Fe^{3+}-mediated strategy is effective on Ti_4O_7. Both BDD and Ti_4O_7 are classified as inactive anodes. This property favors the direct electrochemical generation of •OH and allows the oxidation of Fe^{3+} to high-valent iron species. The strong hydration shell of Fe^{3+} can restructure the H-bond network of interfacial water molecules and promote the oxidation of H_2O to generate •OH. Therefore, the Fe^{3+}-mediated strategy works effectively on these inactive anodes. In conclusion, we have made supplements in the revised manuscript.

Page 25, line 482: More importantly, the addition of Fe^{3+} significantly enhances the pollutant removal performance of inactive anodes (such as Ti_4O_7), but is ineffective on active anodes (such as DSA). This suggests that the Fe^{3+}-mediated H-bond network modulation strategy may also be effective for other inactive anodes, indicating potential universality (Figure S27).

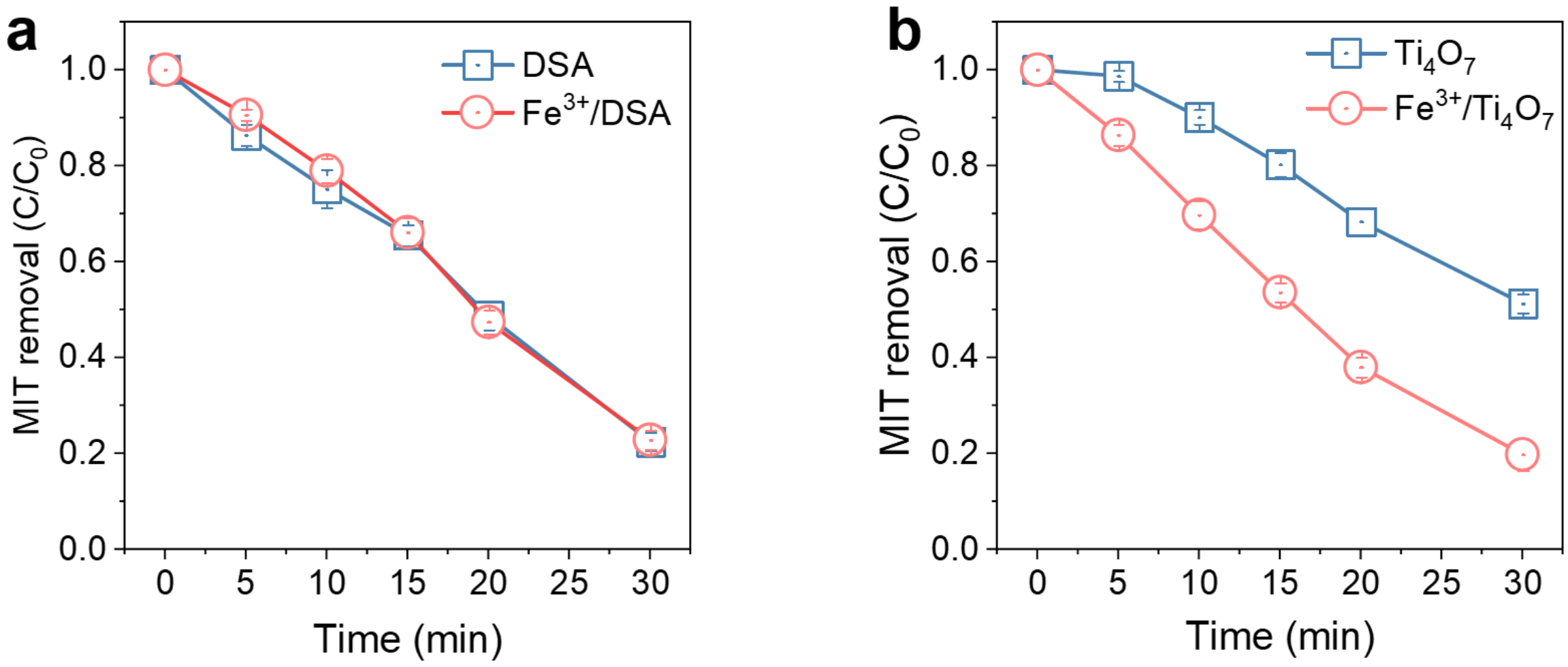

Figure S27. a and b. The removal efficiency of MIT in the DSA, Fe^{3+}/DSA, Ti_4O_7 and Fe^{3+}/Ti_4O_7 systems. (Experimental conditions: $[Na_2SO_4]$ = 50 mM, $[Fe^{3+}]$ = 0.2 mM, current density = 7.5 mA cm^{-2})

9. Comment 9: While the increase in 4-HB-H_2O is correlated with improved performance, the causal relationship remains somewhat indirect. The authors are encouraged to strengthen the discussion by clarifying how changes in H-bond structure quantitatively relate to reaction kinetics or •OH yield, and to acknowledge potential uncertainties in Raman-based fitting.

Response: Thank you for your reminder. We conducted a series of linear relationship fittings between the relative contents of different water structures and the •OH concentration. We found that 4-HB-H_2O has a linear relationship with •OH concentration (Figure S24).

Primarily, we extracted the 4-HB-H_2O population data from our *in situ* Raman analysis (Figure 4b) and the corresponding •OH concentrations were obtained through probe experiments and calculations. A linear fitting was performed with the 4-HB-H_2O content as the independent variable and the •OH concentration as the dependent variable. This high correlation coefficient ($R^2 > 0.96$) quantitatively demonstrates that strengthening the interfacial H-bond network (characterized by the 16%-increase in 4-HB-H_2O) directly correlates with enhanced •OH generation. This result establishes a quantitative link between the microscopic water structure and the macroscopic oxidation performance.

Secondly, the linear correlation described above is underpinned by well-established physical chemistry principles. The O–H bond length in a H-bond water complex increases and the stretching vibration exhibits a red shift as the hydrogen bond strengthens——this bond elongation reduces the activation barrier for water dissociation. In our system, the 4-HB-H_2O enhancement directly modifies the local electronic structure of interfacial water molecules, accelerating the kinetics of water decomposition to generate •OH. Consequently, the Introduction of Fe^{3+} leads to a 7-fold increase in the •OH concentration and a proportional acceleration of the pollutant degradation kinetics. This structure-thermodynamics-dynamics correlation (the increase of 4-HB-H_2O, the weakening of O-H bonds, the reduction of activation energy, the increase of •OH yield, and the acceleration of MIT degradation rate) establishes a clear and quantitative causal link from the microscopic water structure to the macroscopic oxidation performance.

We minimize these uncertainties in the fitting analysis. The same fitting model (number of peaks, initial peak centres, and constraints on the full width at half maximum) is used for all samples to ensure consistency in relative comparisons. We verify the peak assignments by comparing the fitting procedures with previous literature on the fitting of Raman spectra [30, 31]. The monotonically increasing trend of the observed 4-HB-H_2O population with Fe^{3+} concentration and its high correlation (R^2 = 0.96) with the •OH yield is intrinsically consistent, indicating that fitting artifacts do not dominate the conclusion (Figure S24). In summary, this indicates that during the water oxidation process at the BDD anode, the amount of •OH generated is correlated with 4-HB-H_2O.

We believe this response adequately addresses your concern regarding the potential uncertainties in Raman-based fitting. Thank you again for this valuable suggestion, which has improved the transparency and rigor of our work. The fitting data have been included as Figure S24, and the analysis has been incorporated into the revised manuscript.

Page 18, line 359: A linear relationship (R^2 > 0.96) between the content of 4-HB-H_2O and the concentration of •OH was established through quantitative analysis (Figure S24), thereby clarifying the connection between the structural changes of hydrogen bonds and the reaction kinetics.

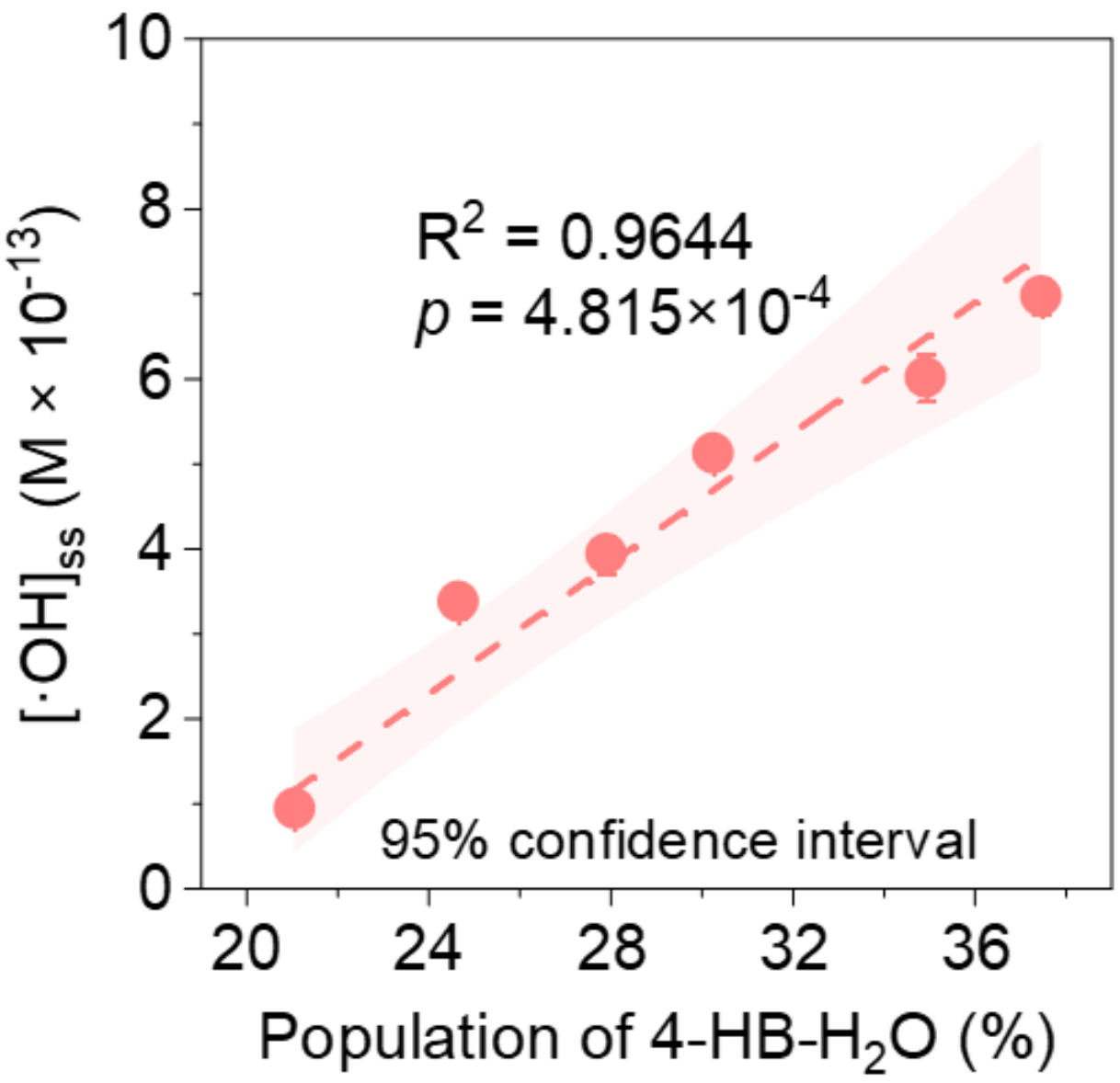

Figure S24. Linear fitting (95% confidence interval) between the proportion of 4-HB-H_2O and the concentration of •OH.

10. Comment 10: Several conclusions regarding interfacial water structure are partially supported by bulk-sensitive techniques (e.g., ^{1}H NMR). The authors should clarify this limitation and distinguish more explicitly between bulk hydration effects and true interfacial phenomena.

Response: We appreciate your suggestions. We agree that techniques such as ^{1}H NMR mainly reflect the hydration environment and hydrogen bonding characteristics of bulk water and cannot be used alone as direct evidence of changes in the structure of interfacial water. Our purpose in using this result was to illustrate that the addition of Fe^{3+} would alter the overall hydration/hydrogen bonding environment. Further combined with *in situ* Raman/FTIR and other interfacial-related evidence, we believe that Fe^{3+} can induce the reconstruction of the interfacial H-bond network and further affect anodic water oxidation and the generation of •OH. We have revised the relevant expressions in the revised manuscript to clearly distinguish the bulk hydration effect from the changes in the interfacial water structure, avoiding over-interpretation. To address this concern, we have supplemented the manuscript as follows.

Page17, line 333: However, the ^{1}H NMR spectrum is an exogenous characterization and cannot represent the conditions at the electrode interface and the working potential. Therefore, to elucidate this effect at the molecular level, interfacial water microenvironmental changes were probed by electrochemical *in situ* Raman and FTIR spectroscopy (Figures 4b–c and Figures S22–S23).

11. Comment 11: Figure presentation and quantitative rigor are required to be strengthened. Some figures (e.g., Fig. 2c, Fig. 6a) would benefit from inclusion of error bars or statistical information to improve quantitative rigor and reproducibility.

Response: Thanks for your insightful and constructive feedback concerning figure presentation and quantitative rigor. In response, we have thoroughly revised Figure 2c and Figure 6a, as well as all the figures in the manuscript, to enhance data transparency, visual clarity and consistency.

(1). For Figure 2c: The literature data cited in this figure were extracted from published sources which did not provide individual replicate data or error bars. Therefore, it is not possible to add error bars to those literature points. However, we have now added error bars for our own experimental data presented in Figure 2c. The revised figure clearly distinguishes between literature values (no error bars) and our measured data (with error bars). This presentation improves transparency while respecting the limitations of previously published data.

(2). For Figure 6a: All data points in Figure 6a represent the average of three independent replicate experiments. For data points that are not 100% removal, the standard deviations were calculated and are now shown as error bars. We note that in the original figure these error bars were very small and thus not clearly visible. In the revised figure, we have enlarged the symbol size and adjusted the error bar styling to make them discernible. For the data points at 100% removal, the standard deviation is zero because all three replicates achieved complete removal at the same sampling time. Therefore, no error bars are shown for these points.

We believe these revisions fully address your concern regarding quantitative rigor and reproducibility. All changes have been highlighted in the revised manuscript.

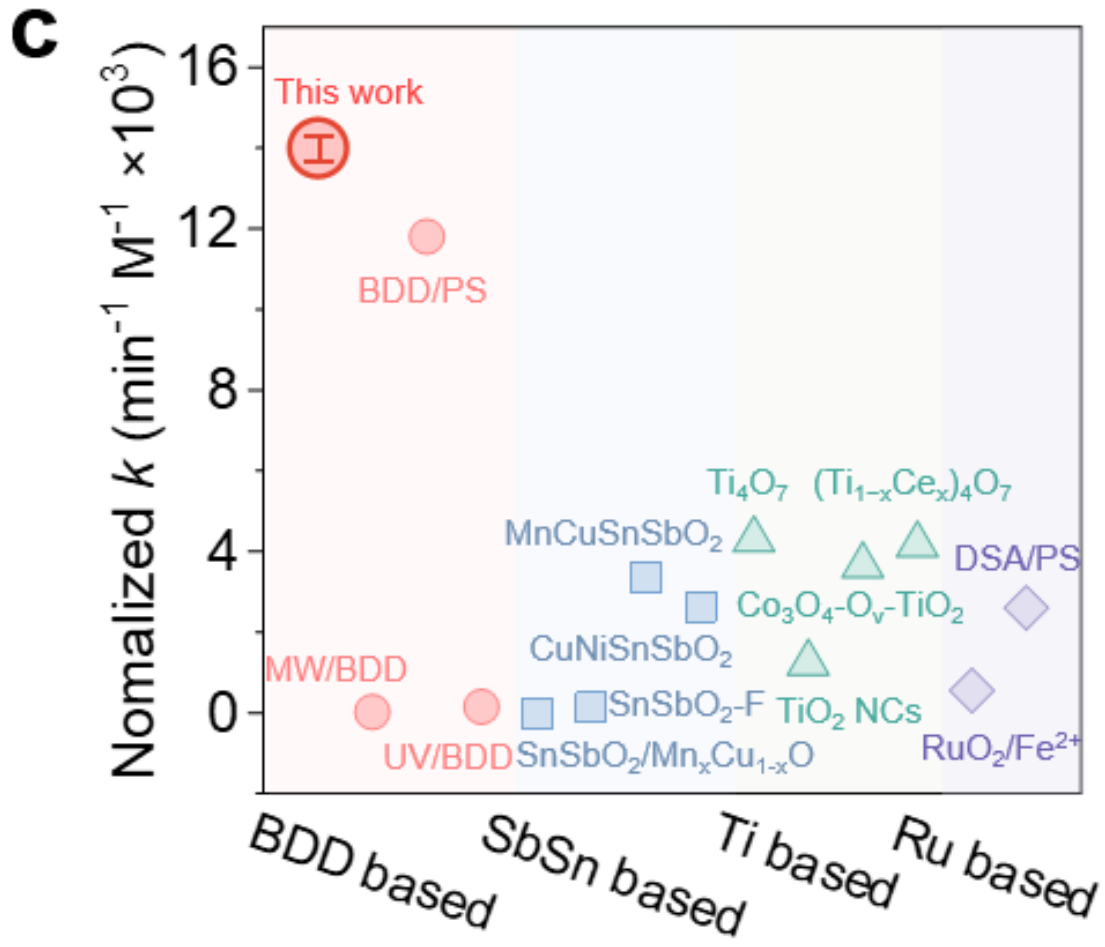

Figure 2. Pollutant degradation and identification of dominant reactive species in the Fe^{3+}/BDD system. c. Comparison of the pollutant normalization degradation kinetics in state-of-the-art anodic oxidation studies.

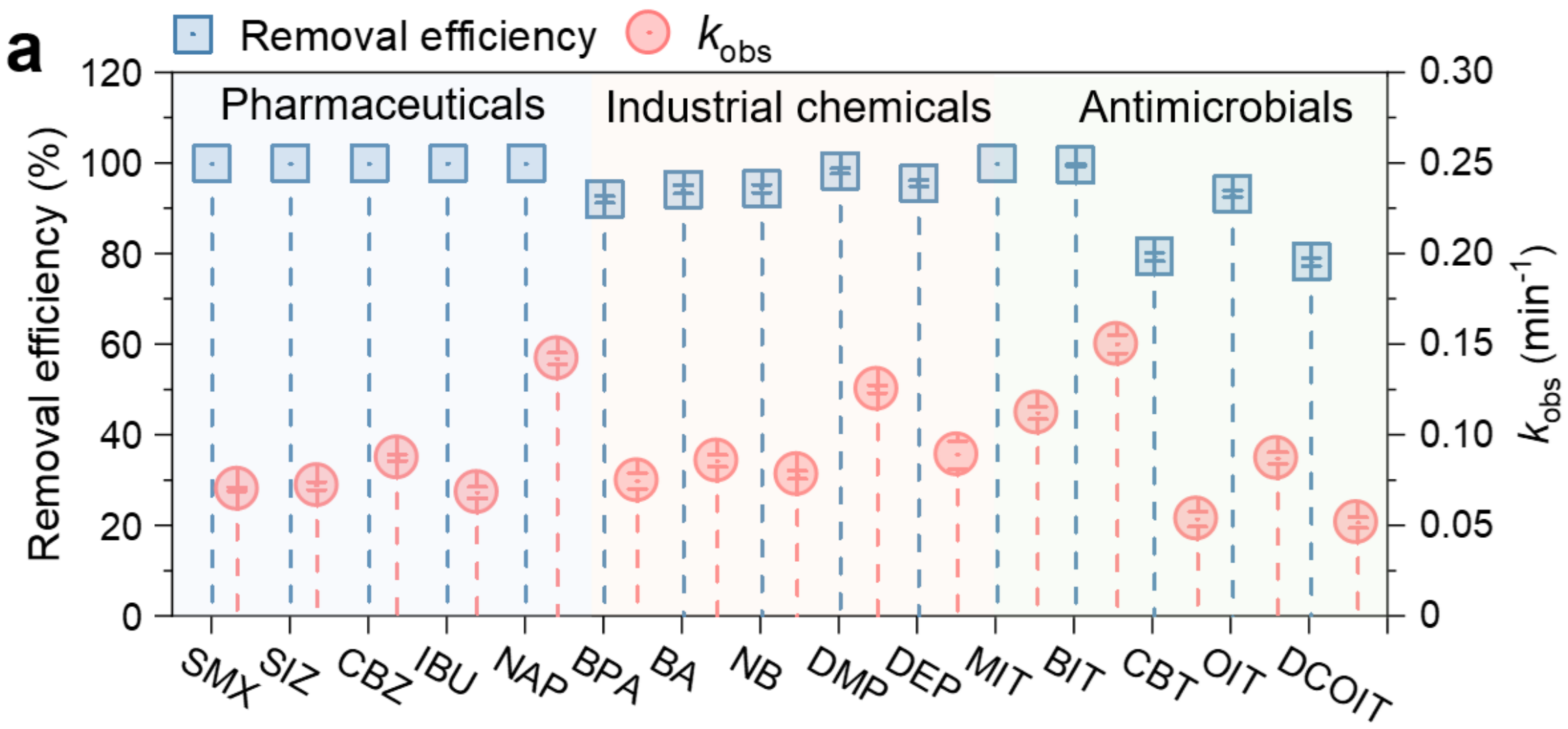

Figure 6. Practical application investigations of Fe^{3+}/BDD system. a. A comparison of removal efficiency and observed rate constant (k_{obs}) for different types of pollutant in the Fe^{3+}/BDD system.(SMX, sulfamethoxazole; SIZ, Sulfanilamide; CBZ, carbamazepine; IBU, ibuprofen; NAP, naproxen; BPA, bisphenol A; BA, benzoic acid; NB, nitrobenzene; DMP, dimethyl phthalate; DEP, diethyl phthalate; MIT, methyl isothiazolinone; BIT, benzothiazolinone; CBT, chlorinated benzothiazolinone; OIT, 2-octyl-isothiazolinone; DCOIT, dichloro-octyl isothiazolinone.)

Reviewer #3 (Remarks to the Author):

This manuscript reports that the introduction of Fe^{3+} strengthens the interfacial hydrogen-bond network and thereby enhances both •OH formation and high-valent iron production in a BDD anodic oxidation system. The topic is potentially interesting, and the authors attempt to connect interfacial water structure with anodic oxidation performance. However, in the present form, the mechanistic argument is not sufficiently substantiated, and the central conclusion appears to go beyond what the data can support.

Response: Thank you very much for your thoughtful and constructive comments on our manuscript. We sincerely appreciate your recognition and endorsement of our research topic. We explored the underlying mechanism from a brand-new perspective of how the hydrogen bond network of water molecules at the interface is restructured by Fe^{3+} to promote the generation of •OH during anodic oxidation. The strong hydration and polarization of Fe^{3+} increase the amount of 4-HB-H_2O at the interface, weaken the O-H bonds of water molecules, and thereby accelerate the kinetics of anode water oxidation. This significantly promotes the generation of •OH through anodization, while also generating high-valent iron species to expand the anodization range. This mechanism has been fully demonstrated through *in situ* electrochemical tests, ^{1}H NMR, *in situ* electrochemical Raman/FTIR, and AIMD.

According to your suggestion, we have supplemented the following additional experiments to further fully demonstrate the mechanism. This includes adding control experiments, probe experiments, quenching experiments, calculations of $k_{obs,\ MIT}$, 2D DOSY NMR analysis, and revised certain strong statements. Through these revisions, we aim to make the mechanism analysis more rigorous and the evidence chain more complete, thereby enhancing the scientific validity and reliability of this work.

1. Comment 1: The novelty of this work requires further consideration. In line 90-92 the article stated that its novelty lies in the limited prior exploration of “microscopic regulations of the water environment at the anode solid-liquid interface and increase the production and utilization rate of •OH”. However, the hydrogen-bond mediated interface electrolyte engineering is a generally used method in regulating activity, selectivity, and durability (https://doi.org/10.1016/j.joule.2025.102306). And regulation

of interfacial hydrogen-bond networks through the introduction of functional ligands, molecular additives, or ionic species has already been explored in several studies (https://doi.org/10.1016/j.ijhydene.2025.152286; https://doi.org/10.1021/jacs.5c22753; https://doi.org/10.1002/adfm.202526825)

Moreover, the increased production of •OH remains insufficiently convincing. Its contribution to overall oxidation enhancement is not convincingly separated from high-valent iron species formation. BDD anode assisted high-valent iron species production in water treatment area, is not conceptually new. (https://doi.org/10.1021/acs.est.2c09237; https://doi.org/10.1021/acs.est.3c01850)

Response: We thank you for this detailed and constructive comment regarding the novelty of our work. We greatly appreciate the literature provided, which we have carefully examined. Following your suggestion, we will present a detailed discussion to clarify the innovative aspects of this study.

(1). Innovation and significance of this study

Although previous reports have indicated hydrogen-bond (H-bond) mediated interfacial electrolyte regulation and BDD anode-assisted generation of high-valent iron species [32, 33, 34, 35, 36, 37]. However, the innovation of this study lies not in simply repeating these concepts, but in elucidating how Fe^{3+} enhances the BDD anode oxidation process by modulating the interfacial water structure. We investigated the interfacial mechanism by which Fe^{3+} enhances the oxidation capacity of BDD anode systems from a new perspective—namely, the role of interfacial water, and provided insights for studying other inactive anode systems (e.g., Ti_4O_7, Figure S27).

Specifically, unlike other BDD-based systems assisted by high-valent iron species, this system is dominated by •OH as the primary active species in electrochemical water treatment. From the perspective of ionic hydration and interfacial H-bond network modulation, we demonstrate that the introduction of Fe^{3+} can reconstruct the local aqueous environment, promote anodic water activation and •OH generation, and improve the effective utilization of •OH. Furthermore, the high-valent iron species generated through further anodic oxidation of Fe^{3+} serve as an auxiliary oxidation pathway, thereby expanding the oxidative capacity and broadening its pollutant removal range. This study proposed a new paradigm for regulating the hydrogen bond

network of water molecules at the interface, opening up new ideas for addressing the inherent limitations in the anodic oxidation process.

(2). Differences with the provided literature

We recognize that H-bond mediated interfacial electrolyte engineering has been explored in fields such as electrocatalytic hydrogen evolution (HER), oxygen evolution (OER), and carbon dioxide reduction reactions (CO_2RR) (https://doi.org/10.1016/j.joule.2025.102306)[32]. However, there remains a significant gap in understanding how H-bond regulates the anodic water oxidation process to generate •OH. From a novel perspective, we propose that Fe^{3+} can modulate the H-bond network of interfacial water molecules at the anode, thereby accelerating the kinetic process of homolytic cleavage of water molecules to produce •OH. Furthermore, our research focuses on electrochemical active oxidants in wastewater treatment. Previous studies did not provide a clear understanding of the structure of interface water and the generation of electrochemical active oxidants. Therefore, establishing a reliable mechanism framework that can link the structure of interface water with the generation of •OH remains a key challenge that has not been resolved in this field.

To overcome this, we adopted a comprehensive multi-technique strategy combining (Figure 4 and Figure 5): ^{1}H NMR as a bulk-phase probe of water H-bond network perturbations caused by hydrated Fe^{3+} species; *In situ* Raman/FTIR spectroscopy to monitor near-electrode water structure under applied voltage; AIMD to visualize the process of Fe^{3+} in the changes of water structure. Quantitative analysis revealed a 16% increase in the abundance of the 4-HB-H_2O motif (Figure 4d and Figure 4e) and a sevenfold increase in •OH concentration (Figure 2f). And a linear connection between the microstructure of water and the macroscopic oxidation performance was established (Figure S24). This multidimensional characterization strategy—^{1}H NMR, *in situ* Raman/FTIR spectroscopy, and AIMD analysis—provides a new paradigm for understanding the mechanism by which Fe^{3+} enhances BDD anode oxidation to generate •OH and modulates the restructuring of interfacial water H-bond networks. Furthermore, we demonstrated that this enhancement effect is broadly applicable to other inactive anodes (such as Ti_4O_7), but not to active anodes (such as DSA) (Figure S27), confirming the potential universality of this principle.

Recent studies on Fe(IV)/Fe(V) generation in BDD-based systems (https://doi.org/10.1021/acs.est.2c09237; https://doi.org/10.1021/acs.est.3c01850) [33, 34], have primarily explored the feasibility of producing Fe(IV)/Fe(V) under near-neutral conditions and highlighted their contribution to pollutant oxidation. However, the influence of Fe^{3+} with strong local electric field, strong polarization effect and strong hydration effect on the oxidation process of BDD anode is by no means limited to providing high-valent iron species, its impact on water molecular structure is highly significant. In contrast, our study approaches the system from a fundamentally different angle: we aim to uncover how Fe^{3+} precisely regulates the interfacial water H-bond network to enhance •OH generation and overall oxidation kinetics, before evaluating its synergistic role with high-valent iron species.

Meanwhile, as you pointed out, the approach of regulating the interfacial hydrogen bond network by introducing functional ligands, molecular additives or ionic substances has been explored in numerous studies (https://doi.org/10.1016/j.ijhydene.2025.152286; https://doi.org/10.1021/jacs.5c22753; https://doi.org/10.1002/adfm.202526825)[35, 36, 37]. We have discovered that the unique properties of Fe^{3+} can significantly influence the water molecules H-bond at the interface, facilitating the process of water oxidation at the anode to produce •OH. Therefore, we conducted a thorough investigation base on sufficient evidence, linking the H-bond structure of microscopic water molecules with the kinetics of •OH generation.

(3). Evidence of •OH increase and clarification of the contribution from reactive species

Our study focuses on how the strong polarization effect and hydration ability of Fe^{3+} alter the microstructure of water and promote water oxidation on BDD anode. Through EPR measurements, we demonstrated that •OH increase with rising Fe^{3+} dosage (Figure 2e). After quenching with MeOH, the MIT removal rate in the Fe^{3+}/BDD system decreased by 75.8%, whereas that of BDD alone decreased by only 44.8%. This 31% difference is attributed to the enhanced generation of •OH resulting from the introduction of Fe^{3+} (Figure 2d). The NB probe experiment and kinetic calculations showed that adding 0.2 mM Fe^{3+} increased the •OH concentration from 9.5×10^{-14} M to 3.39×10^{-13} M (Figure 2f and Figure S7). In addition, quenching experiments and

kinetic calculations further indicated that •OH contributes dominantly (up to 88.9%) to MIT removal (Figure 2g and Text S6), with Fe^{3+}-accelerated water decomposition generating •OH being the primary oxidation mechanism, while Fe(IV)/Fe(V) plays a supporting role.

Thus, our study not only proposes a novel paradigm in which Fe^{3+} reconstructs the hydrogen bonding network of water molecules to enhance •OH generation via BDD anodic oxidation, but also establishes a clear mechanism framework based on interfacial hydrogen bond engineering for understanding and designing EAOPs.

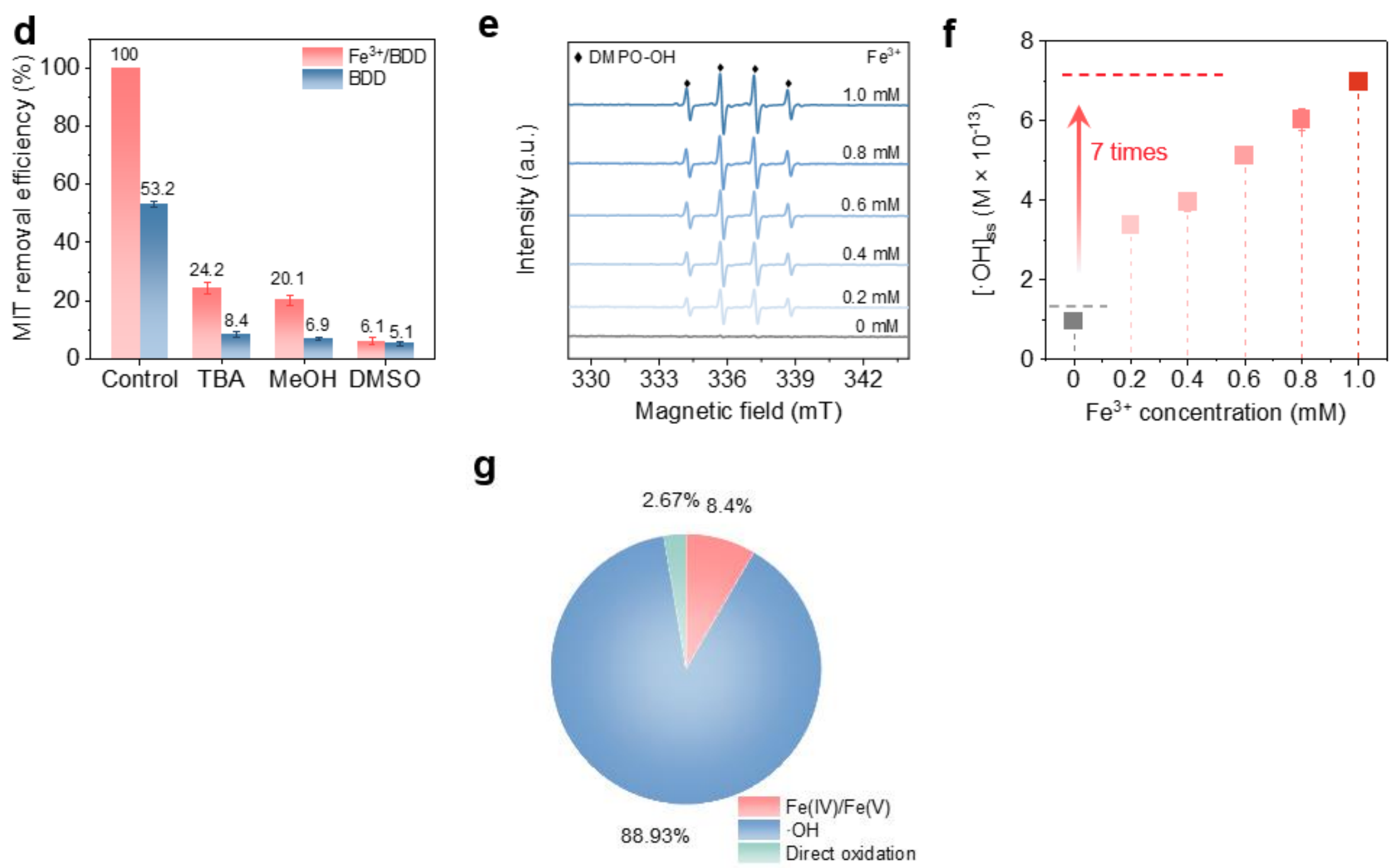

Figure 2. Pollutant degradation and identification of dominant reactive species in the Fe^{3+}/BDD system. d. Effect of scavenger (TBA, MeOH and DMSO) on the removal of MIT in the BDD and Fe^{3+}/BDD systems. **e.** Effect of Fe^{3+} concentration on the DMPO-OH signal intensity in the EPR test. **f.** Effect of Fe^{3+} concentration on the generated concentration of •OH in Fe^{3+}/BDD. **g.** Relative contributions of reactive species to MIT removal in the Fe^{3+}/BDD system. (Experimental conditions: current density = 7.5 mA cm^{-2}, [Na_2SO_4] = 50 mM, [MIT] = 20 μM, [DMPO] = 20 μM, [TBA] = [MeOH] = 200 mM, [DMSO] = 50 mM, [Fe^{3+}] = 0.2 mM)

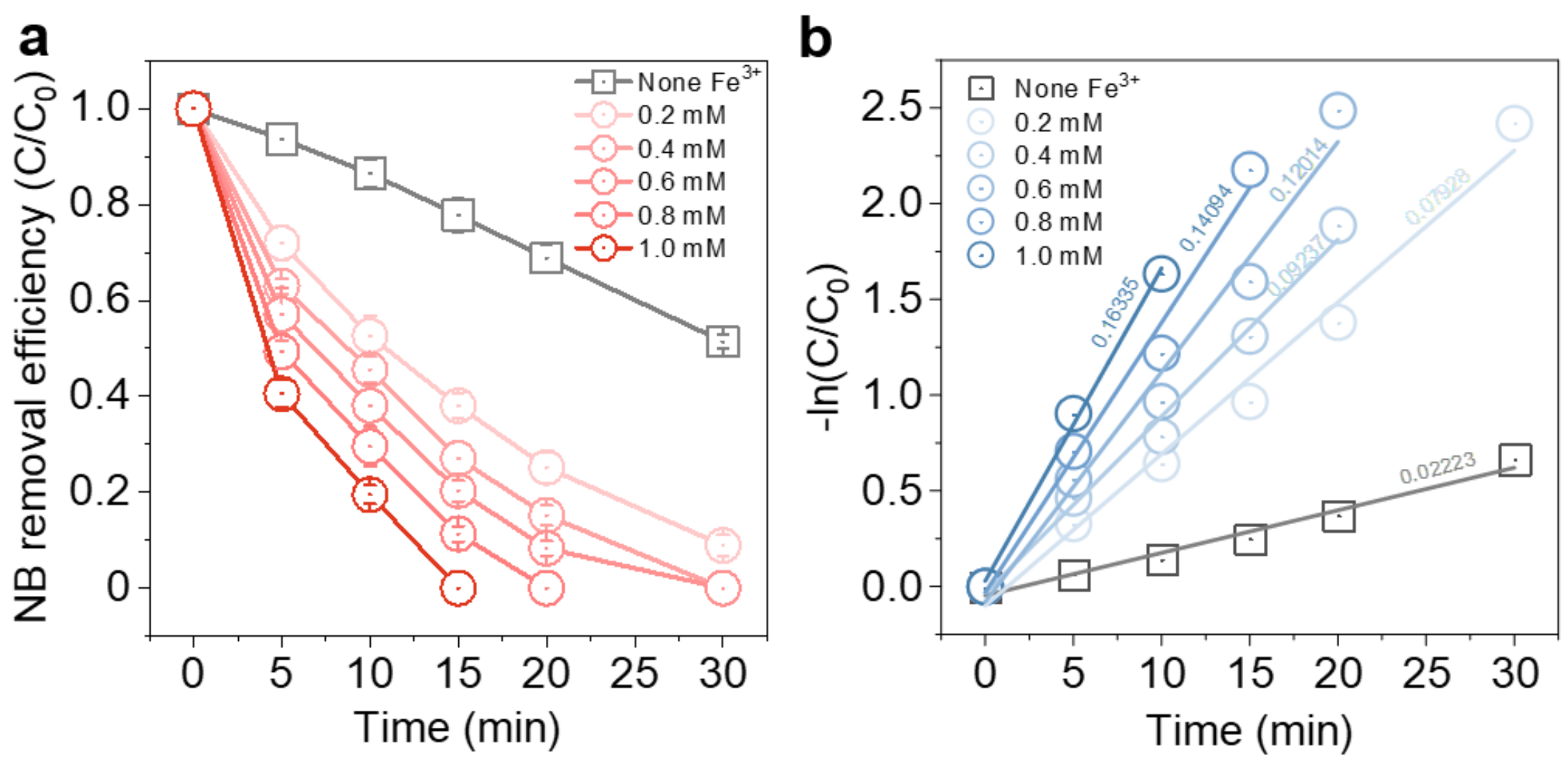

Figure S7. Probe experiments of NB as an •OH probe in Fe^{3+}/BDD system with different Fe^{3+} concentrations: **a.** the decay situation of the probe compound, **b.** the corresponding first-order kinetic curve. (Experimental conditions: [Na_2SO_4] = [$KClO_4$] =50 mM, [NB] = 20 μM, current density = 7.5 mA cm^{-2})

2. Comment 2: The evidence for enhanced •OH production upon Fe^{3+} addition is internally inconsistent.

Response: Thank you for this helpful comment. In the revised manuscript, we have supplemented additional experiments based on the existing evidence to further verify the enhanced •OH generation after Fe^{3+} addition. The specific revisions are as follows.

2.1 Based on Fig. 2d, the removal ratio quenched by TBA and MeOH in the systems with and without Fe^{3+} do not appear substantially different (100% to 38.7% and 53.2% to 8.4%, respectively). If this interpretation is correct, the contribution of •OH to oxidation may be similar in the two systems, which would weaken the conclusion that Fe^{3+} markedly enhances •OH production.

Response: We thank you for this careful observation. In the initial manuscript, we provided direct and indirect evidence for the enhancement of BDD anodic oxidation to generate •OH by adding Fe^{3+} through NB probe experiments and EPR tests (Figure 2e and Figure 2f). To further clarify the accuracy of the quenching experiments more

accurately, we have supplemented the following experiments with a concentration gradient of the quenching agent.

We conducted a series of quenching experiments using a gradient concentration of TBA/MeOH (0 - 1000 mM) under consistent experimental conditions (Figure S5). At low concentrations of TBA and MeOH, the removal rate of MIT in the Fe^{3+} system was significantly higher than that in the system without Fe^{3+}, which is consistent with the higher availability of •OH. However, we found that the concentration of quencher required to completely inhibit the removal rate was significantly different. The 200 mM MeOH or TBA we previously selected was insufficient to completely quench the •OH in the Fe^{3+}/BDD system. In the BDD system, 200 mM TBA or MeOH could almost completely inhibit the removal of MIT. But in the Fe^{3+}/BDD system, the concentration of TBA or MeOH had to be increased to 500 mM for the removal rate of MIT to drop below 20%, and further increasing the quencher concentration did not further inhibit. These results indicate that the introduction of Fe^{3+} indeed significantly enhanced the generation of •OH. We regret the oversight in failing to account for the effect of quencher concentration, which resulted in an underestimation of the contribution of •OH generation to the observed phenomenon.

Based on the above, we have already carried out the calibration. We have corrected the data in Figures 2d and Figures 2g of the manuscript (using 500 mM MeOH as the quenching agent). The results of the calibration show that 500 mM MeOH can reduce the degradation of MIT by the Fe^{3+}/BDD system from 100% to 24.2% (a decrease of 75.8%). Meanwhile, 500 mM MeOH can reduce the degradation of MIT by the BDD system from 53.2% to 8.4% (a decrease of 44.8%). The quenching experiment results conclusively demonstrate that Fe^{3+} promotes •OH generation during BDD anode oxidation. Furthermore, quantitative contribution analysis of the reactive species reveals that •OH accounts for 88.9% (Figure 2g), establishing it as the dominant reactive species in the Fe^{3+}/BDD system. This revision has been incorporated at line 188 of the manuscript.

In original manuscript, the quantitative determination of •OH concentration by the NB probe experiment indicated that the •OH concentration increased with the addition of Fe^{3+} (Figure 2e). The EPR test results show that the signal of DMPO-OH increases with the increase of the Fe^{3+} concentration (Figure 2f). In conclusion, the

supplementary quenching experiments, probe experiments and EPR tests provide compelling evidence that the addition of Fe^{3+} in the BDD anodic oxidation system significantly increases the production of •OH.

In summary, after quenching with MeOH, the MIT removal rate in the Fe^{3+}/BDD system decreased by 75.8%, whereas that of BDD alone decreased by only 44.8%. This 31% difference is attributed to the enhanced generation of •OH resulting from the introduction of Fe^{3+}. In this system, the introduction of Fe^{3+} significantly accelerates the reaction kinetics of water oxidation at the BDD anode to generate •OH, thereby greatly enhancing the production of •OH. Meanwhile, Fe^{3+}/Fe(H) cycle further promotes the dissociation of interfacial water and generating more Fe(H). The primary degradation pathway remains driven by •OH, while the Fe^{3+}/Fe(H) cycle enhances overall oxidation kinetics and broadens the degradation range. In summary, we have made the following additions in the revised manuscript.

Page 8, line 160: After quenching with MeOH, the MIT removal rate in the Fe^{3+}/BDD system decreased by 75.8%, whereas that of BDD alone decreased by only 44.8%. This 31% difference is attributed to the enhanced generation of •OH resulting from the introduction of Fe^{3+}.

Page 9, line 188: Quenching analysis and kinetic calculations (Figure 2g, Figure S13, and Text S6) indicates that •OH and Fe(IV)/Fe(V) together dominate pollutant degradation, contributing 88.9% and 8.4%, respectively, whereas direct electron transfer plays a negligible role (2.7%).

Page 10, line 191: According to literature and experimental results, the second-order reaction rate constant of •OH with MIT ($k_{\bullet OH,MIT}$ = 2.09 × 10^9 M^{-1} s^{-1}) is significantly higher than that of Fe(V)/Fe(IV) ($k_{Fe(V)/Fe(IV),MIT}$ = 1.08 × 10^3 M^{-1} s^{-1}) [19] (Text S8 and Figure S14), but the steady-state concentration of •OH (3.39×10^{-13} M) is lower than that of Fe(V)/Fe(IV) (1.20×10^{-7} M) (Text S7). Therefore, the $k_{obs,MIT}$ of •OH is 5.5 times that of Fe(V)/Fe(IV) (Text S9 and Figure S14), consistent with their relative contributions to MIT removal.

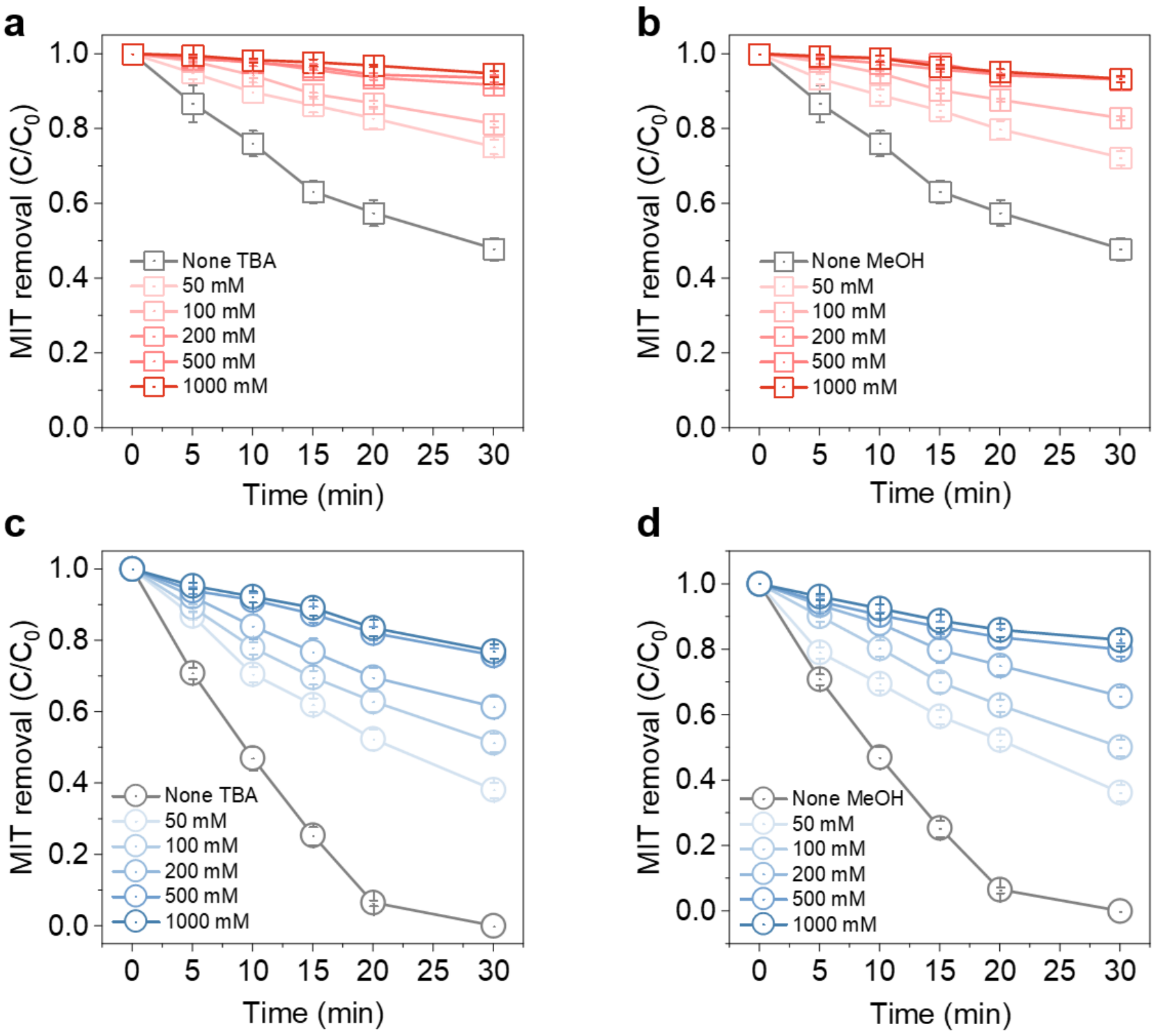

Figure S5. a and b. Effect of different TBA and MeOH concentrations on the attenuation of MIT in the BDD system. **c and d.** Effect of different TBA and MeOH concentrations on the attenuation of MIT in the BDD system. (Experimental conditions: [Na_2SO_4] = 50 mM, [MIT] = 20 μM, [Fe^{3+}] = 0.2 mM, current density = 7.5 mA cm^{-2})

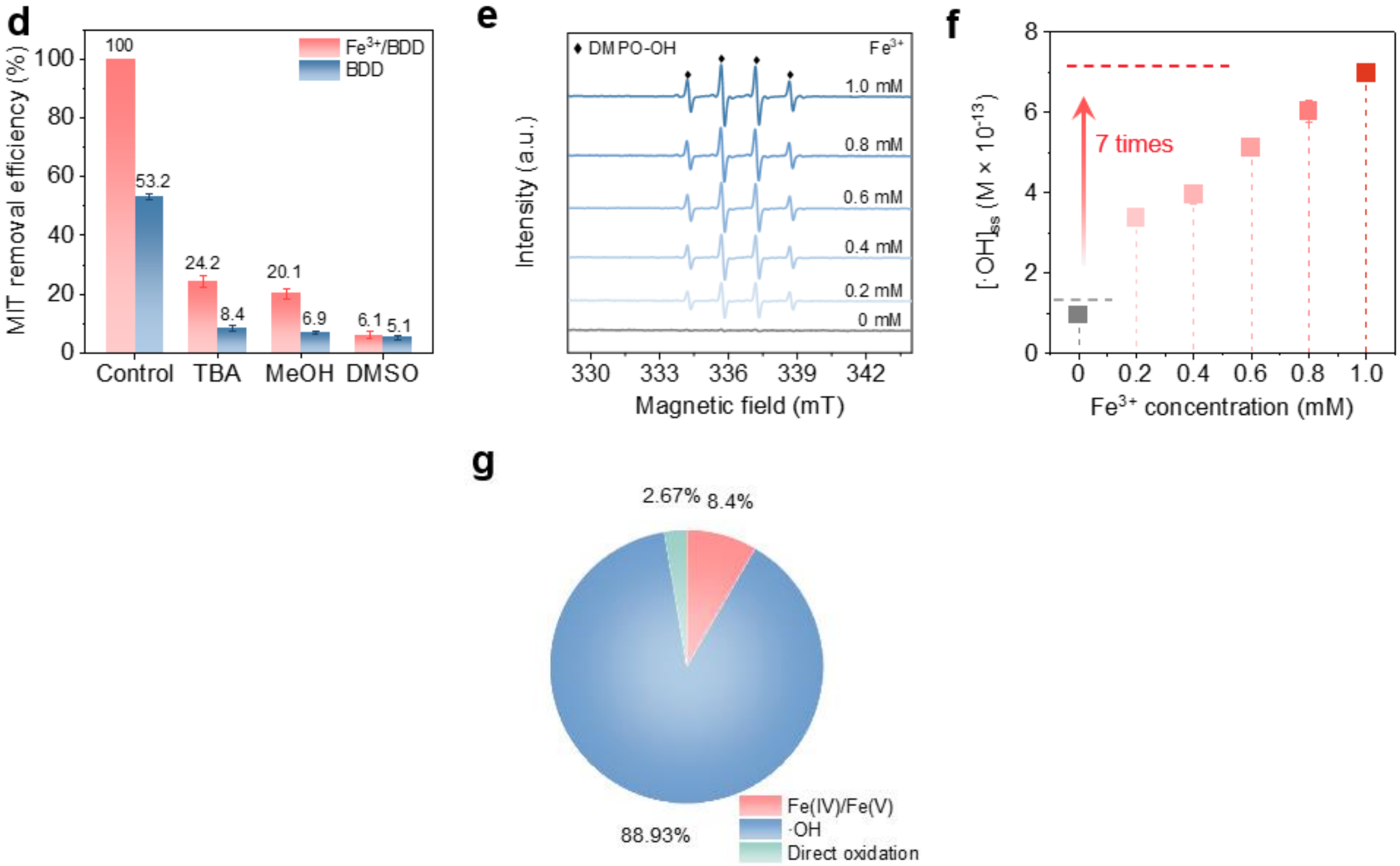

Figure 2. Pollutant degradation and identification of dominant reactive species in the Fe^{3+}/BDD system. d. Effect of scavenger (TBA, MeOH and DMSO) on the removal of MIT in the BDD and Fe^{3+}/BDD systems. **e.** Effect of Fe^{3+} concentration on the DMPO-OH signal intensity in the EPR test. **f.** Effect of Fe^{3+} concentration on the generated concentration of •OH in Fe^{3+}/BDD. **g.** Relative contributions of reactive species to MIT removal in the Fe^{3+}/BDD system. (Experimental conditions: current density = 7.5 mA cm^{-2}, [Na_2SO_4] = 50 mM, [MIT] = 20 μM, [DMPO] = 20 μM, [TBA] = [MeOH] = 200 mM, [DMSO] = 50 mM, [Fe^{3+}] = 0.2 mM)

2.2 According to the equation S4 provided by the authors, since k_{NB} is constant (3 × 10^9 M^{-1} s^{-1}), $[•OH]_{ss}$ should be proportional to $k_{obs, NB}$. However, In Fig. 2b, the k_{obs} at 0.4-0.8 mM Fe^{3+} seems nearly unchanged, whereas Fig. 2f shows a clear stepwise increase. The manuscript should explain this inconsistency.

Response: We thank you for this critical observation. We appreciate the opportunity to clarify the apparent inconsistency between the $k_{obs, NB}$ data (Figure S7) and the $k_{obs, MIT}$ data (Figure 2b). We clarify the $k_{obs, MIT}$ data as follows.

The key point to recognize is that NB is highly reactive toward •OH but exhibits negligible reactivity toward high-valence iron species. Numerous studies have reported that the second-order rate constant for the reaction of Fe(VI) with NB is around 96-127 $M^{-1}·s^{-1}$ [38]. By contrast, the reaction between MIT and •OH proceeds with a second-order rate constant of 2.09 × 10^9 $M^{-1}·s^{-1}$, while the rate constant for Fe(VI) with MIT is even slower. Consequently, $k_{obs, NB}$ reflects solely the •OH contribution, whereas $k_{obs, MIT}$ reflects the combined contributions of both •OH and high-valence iron species. This selective reactivity explains the observed divergence in trends.

Secondly, we sincerely apologize for the fitting deviations of the data points of $k_{obs,MIT}$ in Figure 2b. Due to our previous oversight, we used 15-minute and 20-minute points for the results of 0.2 mM and 0.4 mM Fe^{3+} addition, which led to an excessive deviation of $k_{obs, MIT}$. And when fitting with 0.6 mM, 0.8 mM and 1.0 mM Fe^{3+} addition, we took fewer sampling points, resulting in an excessive deviation of $k_{obs, MIT}$. After careful consideration, we added 1-minute, 3-minute and 7-minute sampling points to the MIT degradation process in Figure 2a and re-performed the fitting of -ln(C/C_0). We

supplemented the data in Figure 2a and modified the corresponding $k_{obs, MIT}$ in Figure 2b.

We are once again deeply sorry for our mistake. We have re-examined all the data in the manuscript strictly to ensure the authenticity and scientific nature of all the data.

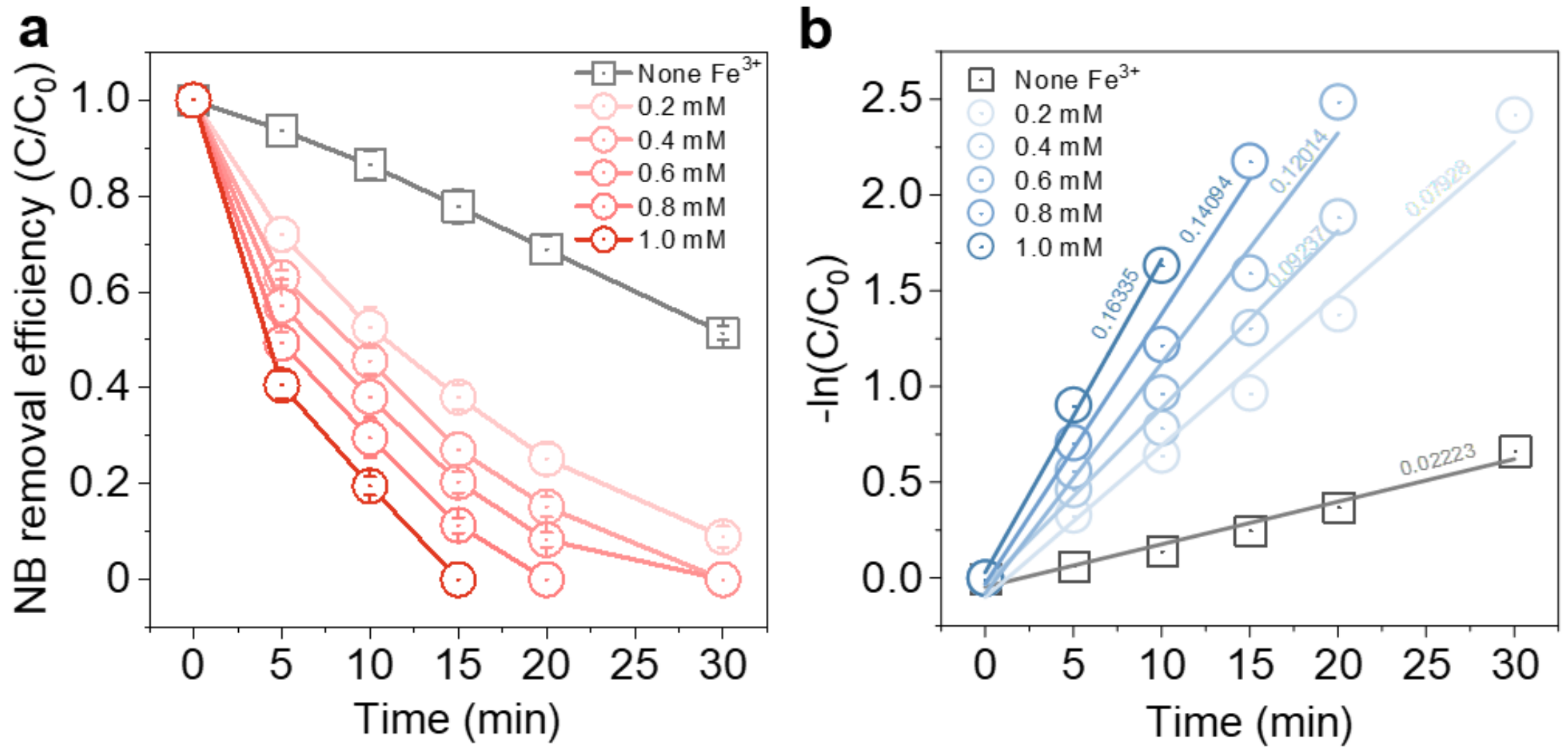

Figure S7. Probe experiments of NB as an •OH probe in Fe^{3+}/BDD system with different Fe^{3+} concentrations: **a.** the decay situation of the probe compound, **b.** the corresponding first-order kinetic curve. (Experimental conditions: [Na_2SO_4] = [$KClO_4$] =50 mM, [NB] = 20 μM, current density = 7.5 mA cm^{-2})

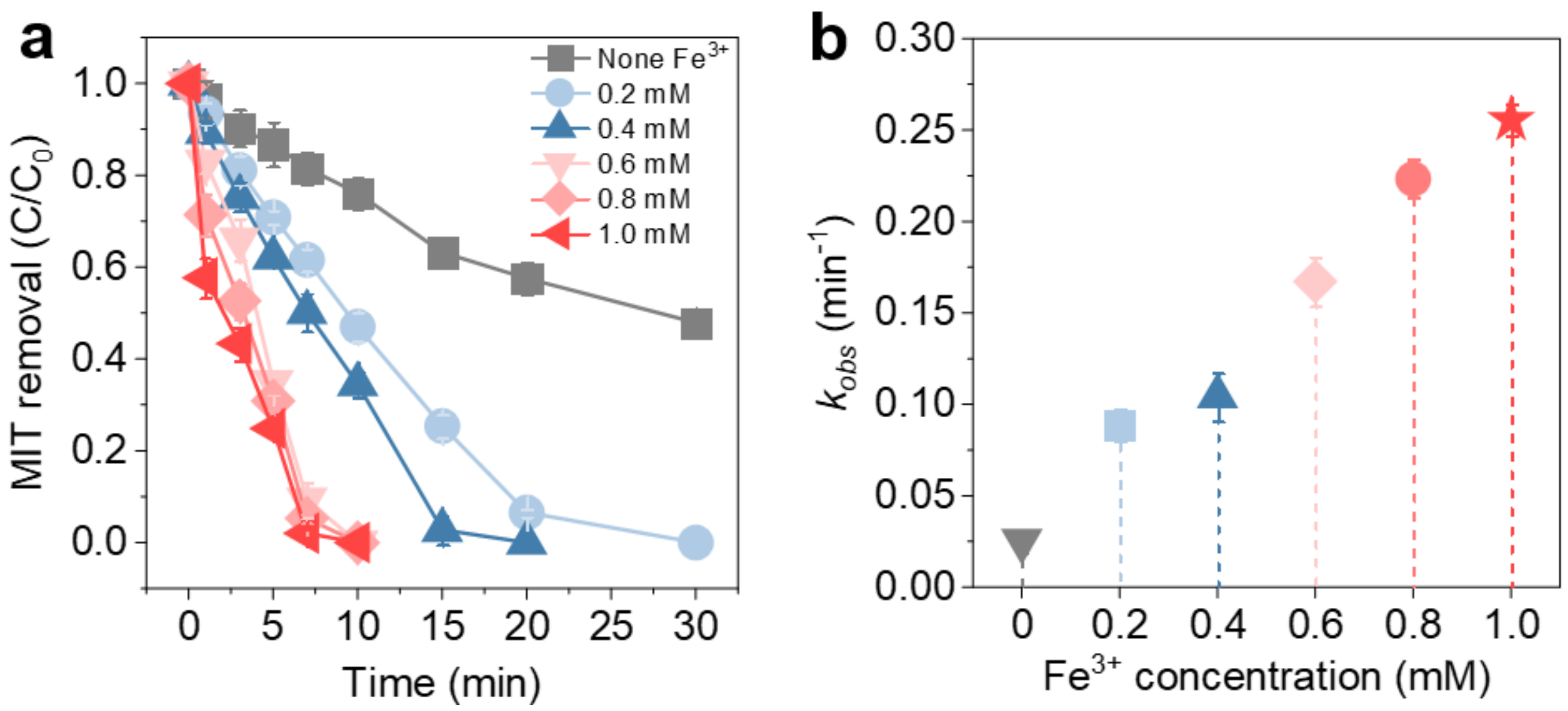

Figure 2. Pollutant degradation and identification of dominant reactive species in the Fe^{3+}/BDD system. a and b. Effect of Fe^{3+} concentration on the removal efficiency of MIT and the observed rate constant (k_{obs}) in Fe^{3+}/BDD system.

2.3 The relationship between Fig. 2g and Fig. S8 is confusing. In Fig. 2g, the contribution of Fe(H) is reported to be only 13.41% under 0.2 mM Fe^{3+}, whereas in Fig. S8 the $PMSO_2$ yield under 1 mM Fe^{3+} exceeds 50%. Since PMSO yield is commonly used as an indicator for high-valent iron contributions, these two results appear difficult to reconcile. The authors need to clearly explain how these values were calculated and why the inferred oxidative contribution of high-valent iron differs substantially between the two datasets.

Response: Thank you for your detailed suggestions. PMSO is a probe that proves the presence of Fe(IV)/Fe(V) in the system (Figure 3a, Figure S9 and Figure S10), and the contribution rate of Fe(IV)/Fe(V) to MIT removal was determined to be 8.4% (revised) (Figure 2g), based on quenching experiments and kinetic calculations. Below we explain the fundamental differences between the two measurements and why they are not directly comparable.

PMSO is a probe molecule that undergoes characteristic transformation to $PMSO_2$ upon reaction with Fe(IV)/Fe(V). A high $PMSO_2$ yield indicates that Fe(IV)/Fe(V) are present and capable of oxidizing PMSO. However, this value does not represent the fractional contribution of Fe(IV)/Fe(V) to the degradation of a different target pollutant (MIT). The $PMSO_2$ yield is a probe-specific, molecule-dependent parameter that reflects the relative reactivity of the coexisting oxidants toward PMSO, not their absolute or relative contributions to MIT oxidation. To avoid any possible confusion, we have provided a clear explanation to clearly distinguish between these two.

In this manuscript, the PMSO probe experiment is only used as evidence for the existence of Fe(IV)/Fe(V). The oxidation contributions of •OH and Fe(IV)/Fe(V) to MIT were calculated based on the results of quenching experiments (Figure 2g and Text S6). We thank you for this insightful comment, which has allowed us to clarify a subtle but important point in interpreting probe-based measurements in multi-oxidant systems.

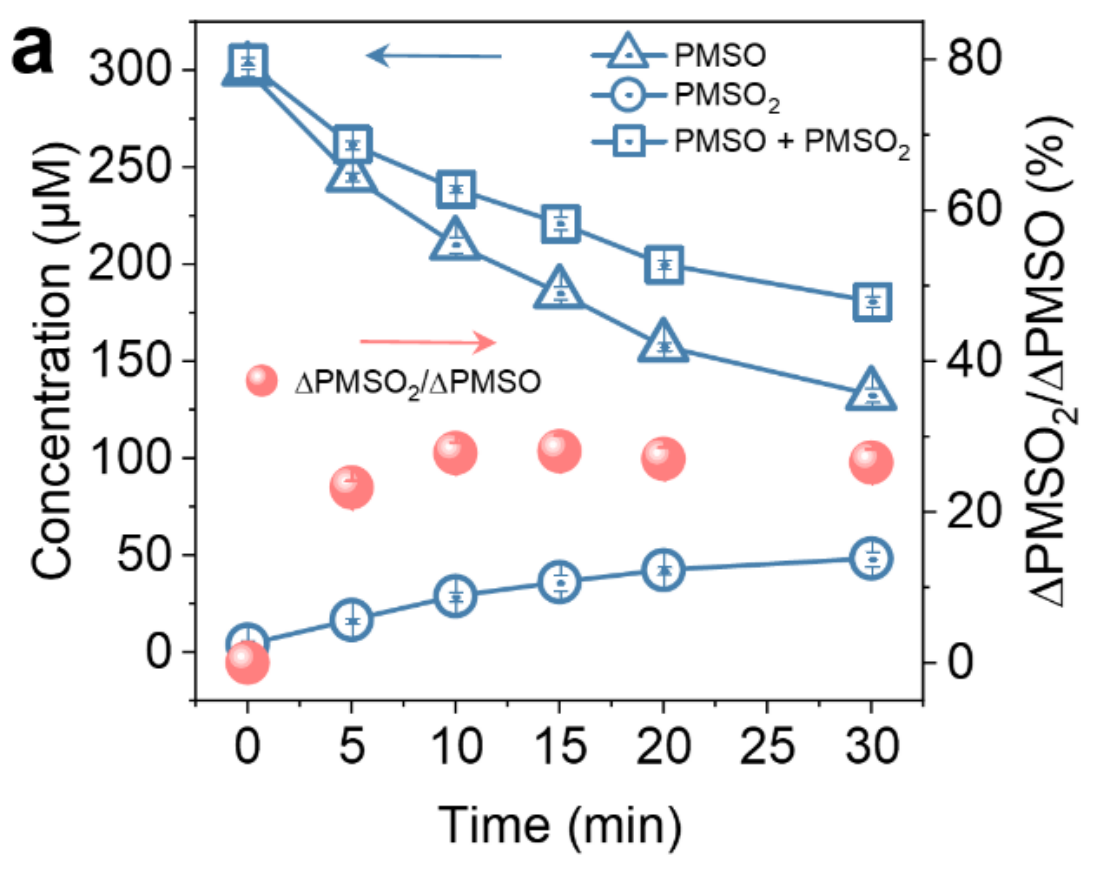

Figure 3. ***Operando*** **electrochemical characterization to monitor the effect of Fe^{3+} on the anodic oxidation interface. a.** Changes in PMSO consumption, $PMSO_2$ generation, and conversion rate over time.

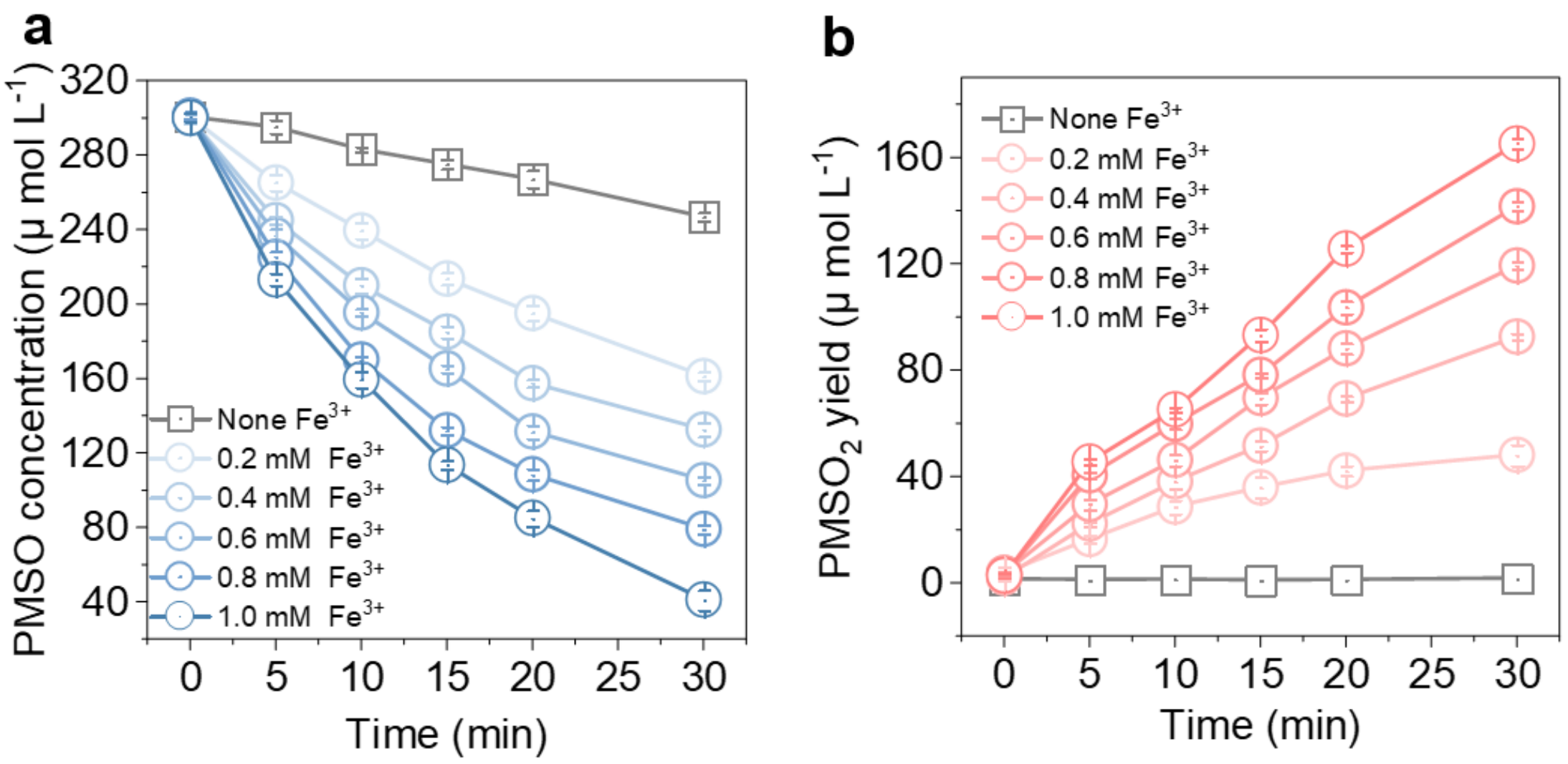

Figure S9. a. b. Effect of different Fe^{3+} concentrations on the consumption amount of PMSO and the generation amount of $PMSO_2$ in the BDD system. (Experimental conditions: [Na_2SO_4] = 50 mM, [PMSO] = 300 μM, current density = 7.5 mA cm^{-2})

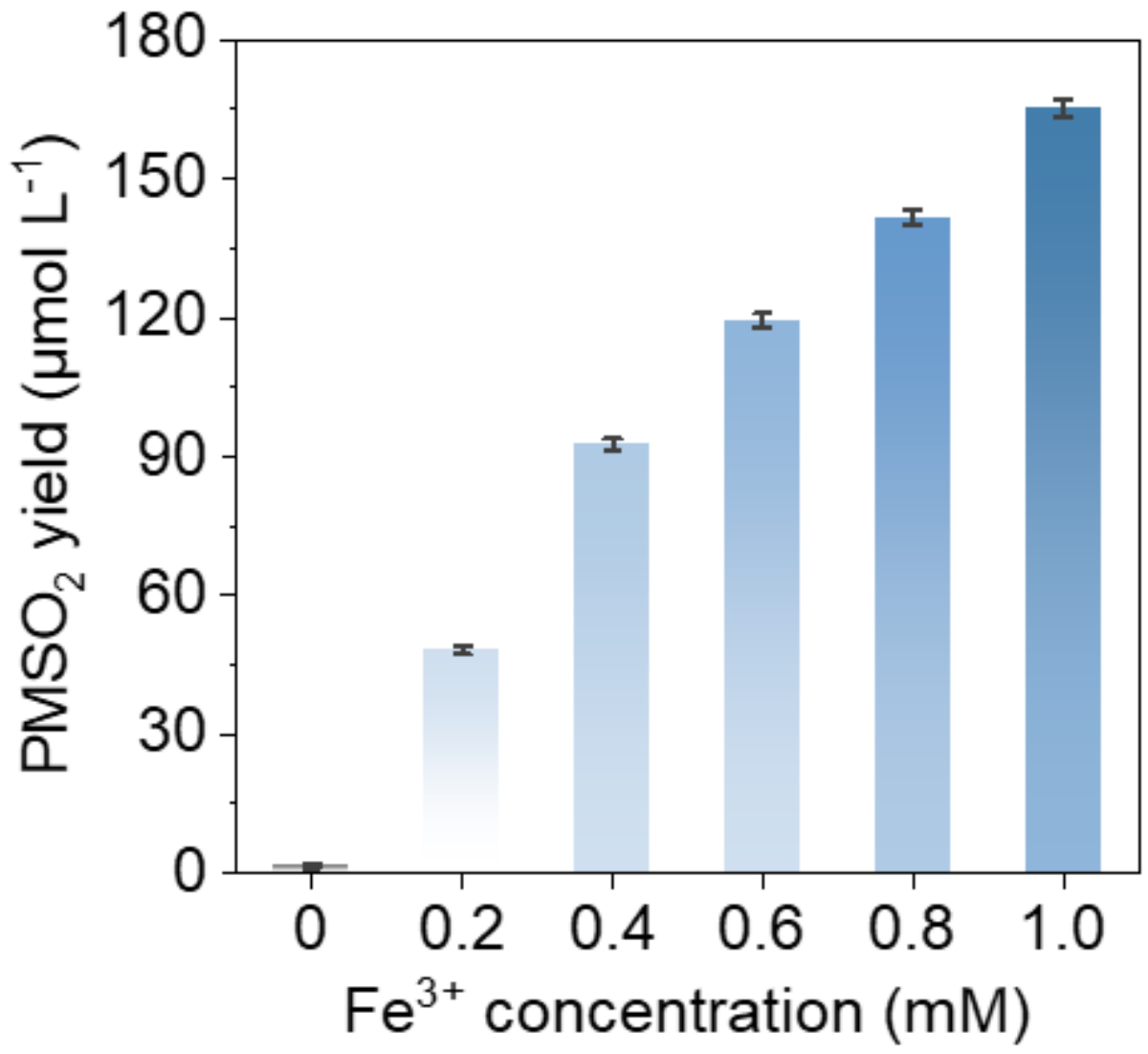

Figure S10. The amount of $PMSO_2$ generated corresponding to different Fe^{3+} concentrations. (Experimental conditions: [Na_2SO_4] = 50 mM, [PMSO] = 300 μM, current density = 7.5 mA cm^{-2})

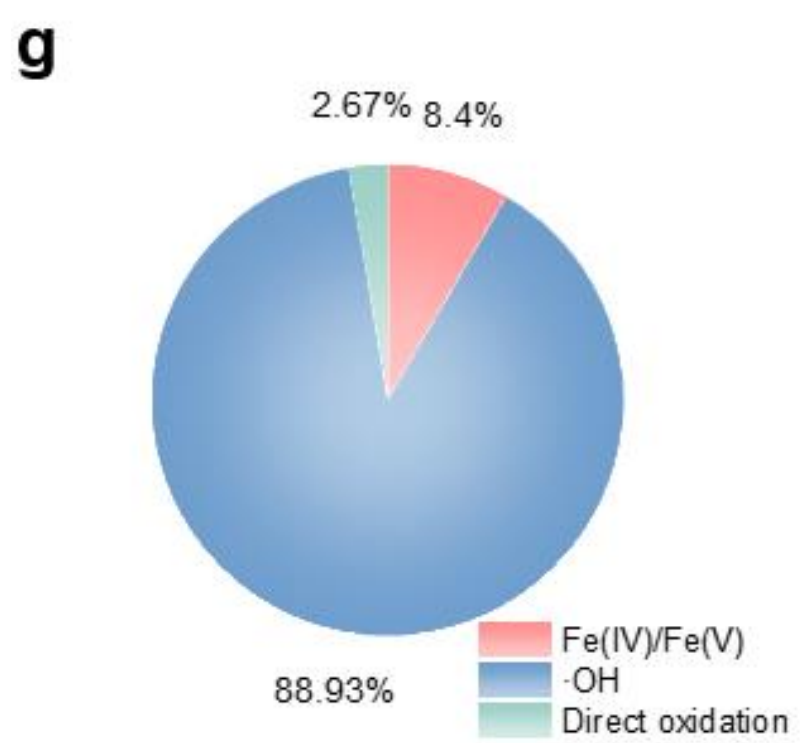

Figure 2. Pollutant degradation and identification of dominant reactive species in the Fe^{3+}/BDD system. g. Relative contributions of reactive species to MIT removal in the Fe^{3+}/BDD system. (Experimental conditions: current density = 7.5 mA cm^{-2}, [Na_2SO_4] = 50 mM, [MIT] = 20 μM, [TBA] = [MeOH] = 500 mM, [DMSO] = 50 mM, [Fe^{3+}] = 0.2 mM)

2.4 Could the authors add a scale bar to indicate the simulated diffusion layer thickness in Fig. 2h? Moreover, within the same thickness range, the simulated concentration of Fe(H) appears to be approximately twice that of •OH. It is also unclear whether this value is directly calculated from the model or defined based on specific assumptions. In addition, if MIT oxidation mainly occurs at or near the interface, it is

not clear whether •OH can still be considered the dominant oxidizing species, as stated in Line 154.

Response: Thanks so much for your very insightful comments. We will address each point below.

(1). Scale bar in Figure 2h. We agree to add a simulated thickness of the diffused active species in Figure 2h, which will help improve clarity. In the revised manuscript, we will add a scale bar to the figure in Figure 2h.

(2). Simulation details. The simulated concentrations are directly calculated from the COMSOL model using the reaction kinetics and boundary conditions described in the manuscript. The specific simulation parameters are as follows.

Text S10: **Finite Element Analysis (FEA) Simulation**. The concentration distribution of •OH and Fe(H) on the surface of the BDD electrode was simulated using COMSOL Multiphysics 6.2 software. A two-dimensional geometry "Transient" module along with a "Cube Current Distribution" module were employed to address the material distribution issue on the BDD electrode surface[20, 21]. The dynamic simulation of the reaction process was conducted utilizing the Butler-Volmer and Nernst-Planck equations[22]. The electrode double layer thickness was set to approximately 10 μm, which is a typical steady-state Nernst electrical double layer for a non-stirred system. The bulk concentration of Fe^{3+} was set to the experimentally optimized value of 0.2 mM. The current density is set at 7.5 mA cm^{-2}. The diffusion coefficient of •OH in aqueous solution was set to about 2.0×10^{-9} m^2 s^{-1}, which is an approximation commonly adopted in electrochemical models for small molecules. For the high valent iron species, a slightly lower value of about 0.6×10^{-9} m^2 s^{-1} has been used, which is comparable to the reported diffusion coefficients of iron cyanide complexes in aqueous electrolytes. This choice accounts for the larger size of the Fe(H) compared to the •OH.

(3). Whether •OH can still be considered the dominant oxidizing species given that MIT oxidation mainly occurs near the interface and [Fe(H)] is higher than [•OH]. The concentrations of •OH and Fe(IV)/Fe(V) calculated based on the results of the NB probe experiment and the PMSO probe experiment were 3.39×10^{-13} M and 1.20×10^{-7} M respectively. Due to the extremely short half-life of •OH, their capture is very difficult, which may result in a relatively low concentration of •OH

obtained. The •OH captured by NB probe experiments represents the average concentration in the bulk phase, whereas COMSOL simulation results show local concentrations at the interface (Figure 2h). Therefore, a certain discrepancy between the two is entirely reasonable.

Although the steady-state concentration of Fe(H) is indeed higher than that of •OH, the kinetic contribution to pollutant degradation depends on both concentration and reaction rate constants. The secondary rate constant of •OH with MIT is 2.09×10^{9} M^{-1} s^{-1} [19]. In contrast, the reaction rate constants of Fe(V)/Fe(IV) with many emerging pollutants are approximately 10^{1}–10^{4} M^{-1} s^{-1} [2], while the rate constant of Fe(V)/Fe(IV) is usually several orders of magnitude lower than •OH. However, there is no literature reporting the reaction rate constant of Fe(V)/Fe(IV) with MIT. We conducted competitive kinetic experiments on MIT and PMSO after completely quenching •OH by adding excess MeOH in the Fe^{3+}/BDD system, and obtained $k_{Fe(V)/Fe(IV),\ MIT} = 1.08 \times 10^{3}$ M^{-1} s^{-1}. Therefore, •OH has an absolute advantage in kinetics compared to Fe(V)/Fe(IV). In conclusion, we can obtain the actual reaction rate of •OH and Fe(IV)/Fe(V) with MIT by multiplying the reaction rate constant and the concentration of active species. The results show that the $k_{obs,MIT}$ for •OH is 5.5 times higher when reacting with Fe(IV)/Fe(V), which is consistent with their respective contributions to MIT removal (Figure 2g).

In this system, the introduction of Fe^{3+} significantly accelerates the kinetics of •OH generation via BDD anode oxidation of water, leading to a substantial increase in •OH production. Meanwhile, Fe^{3+}/Fe(H) cycle further promotes the dissociation of interfacial water and generating more Fe(H). The primary degradation pathway remains driven by •OH, while the Fe^{3+}/Fe(VI) cycle enhances overall oxidation kinetics and broadens the degradation range. We have made additions in the manuscript.

Page 10, line 191: According to literature and experimental results, the second-order reaction rate constant of •OH with MIT ($k_{\bullet OH,MIT} = 2.09 \times 10^{9}$ M^{-1} s^{-1}) is significantly higher than that of Fe(V)/Fe(IV) ($k_{Fe(V)/Fe(IV),MIT} = 1.08 \times 10^{3}$ M^{-1} s^{-1})[19] (Text S8 and Figure S14), but the steady-state concentration of •OH (3.39×10^{-13} M) is lower than that of Fe(V)/Fe(IV) (1.20×10^{-7} M) (Text S7). Therefore, the $k_{obs,MIT}$ of •OH is 5.5 times that of Fe(V)/Fe(IV) (Text S9 and Figure S14), consistent with their relative contributions to MIT removal.

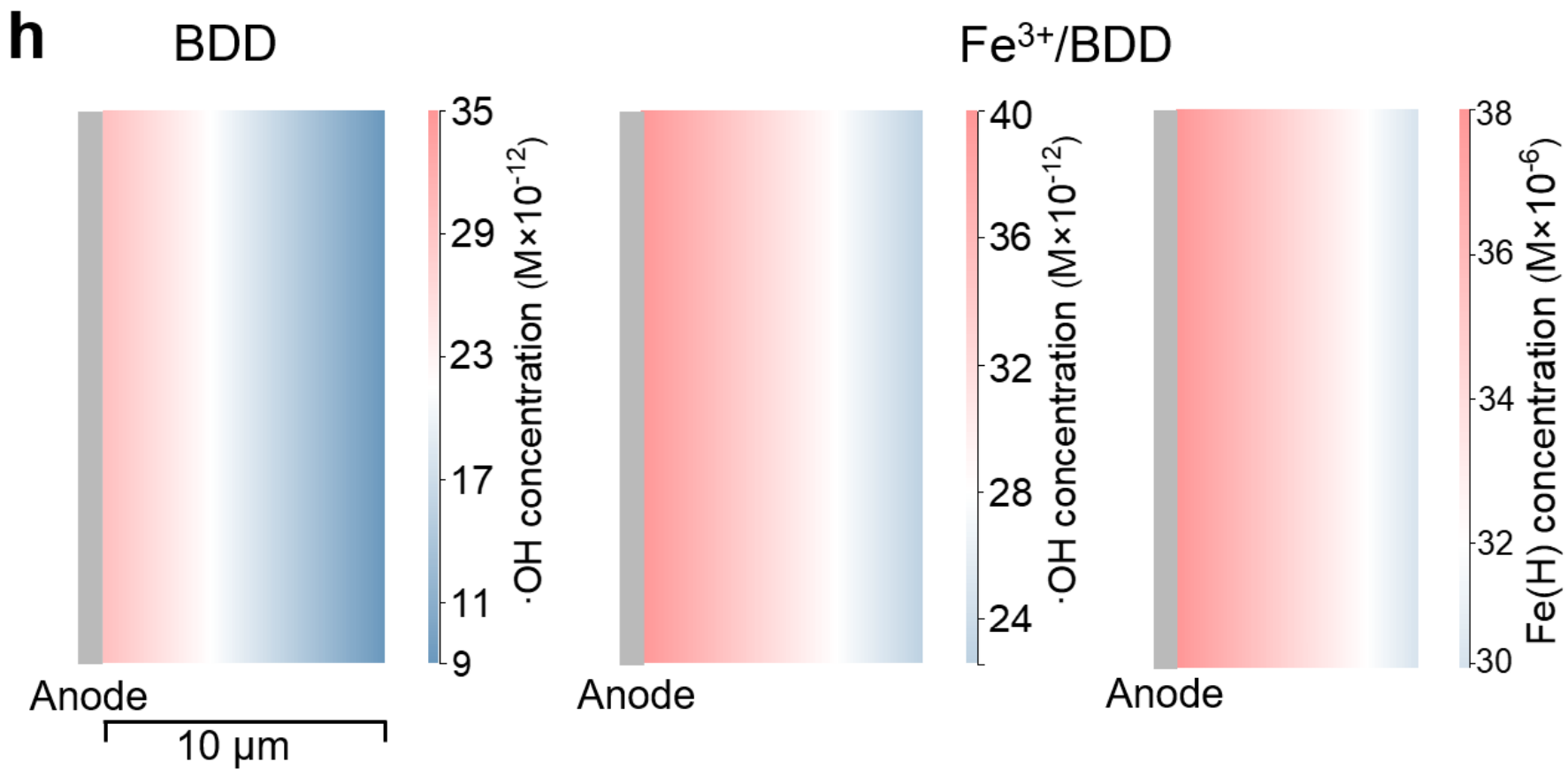

Figure 2. Pollutant degradation and identification of dominant reactive species in the Fe^{3+}/BDD system. h. Concentration distribution of •OH and Fe(H) at the anode interface were simulated using COMSOL in BDD and Fe^{3+}/BDD systems.

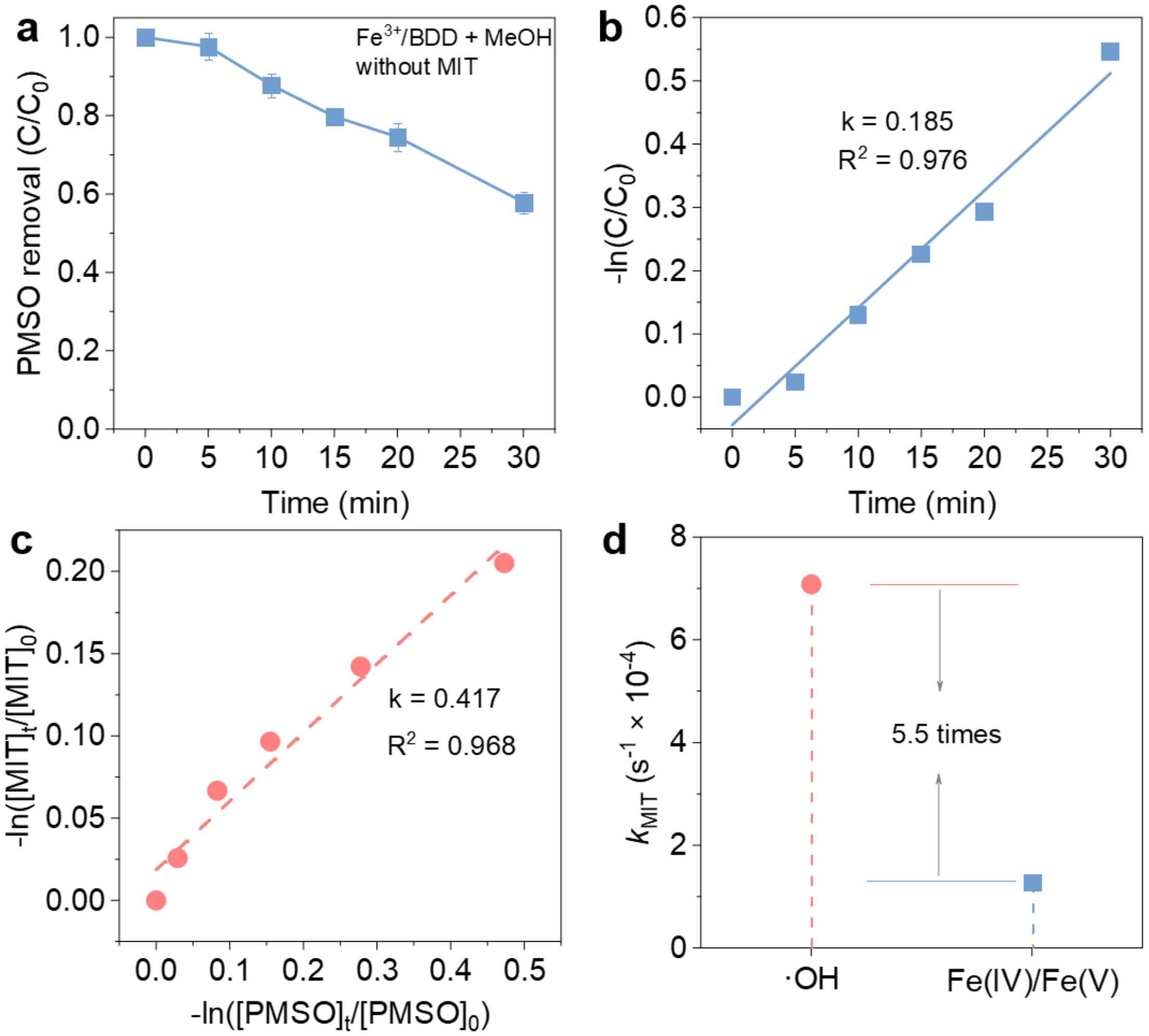

Figure S14. a. and b. Degradation of PMSO in the Fe^{3+}/BDD system during MeOH quenching and its corresponding first-order kinetic curves. **c.** Linear fitting of -

ln($[MIT]_t/[MIT]_0$) and -ln($[PMSO]_t/[PMSO]_0$) obtained from the MeOH quenching process in the Fe^{3+}/BDD system with MIT and PMSO at the same concentration. **d.** the actual reaction rates of MIT with •OH and Fe(IV)/Fe(IV) in the Fe^{3+}/BDD system. (Experimental conditions: [Na_2SO_4] = 50 mM, [MIT] = [PMSO] =20 μM, [Fe^{3+}] = 0.2 mM, [MeOH] = 1000 mM, current density = 7.5 mA cm^{-2})

3. Comment 3: The key evidence supporting hydrogen-bond reconfiguration is weak.

3.1 Line 269-276: The ^{1}H NMR indicated upfield shift at 0.2–0.4 mM Fe^{3+} addition cannot fully support the claim "forming an extended and strongly hydrogen-bonded hydrated sphere centered on $[Fe(OH)(H_2O)_5]^{2+}$, with an increase in the electron cloud density of the H atoms of water molecules and an enhanced H-bond network."

Response: Many thanks for your valuable suggestions and kind reminder. We have reclarified the relationship between hydrogen bonding effects and paramagnetic effects on the shielding and deshielding of hydrogen nuclei, and supplemented the 2D DOSY NMR. The changes in the DOSY diffusion coefficients further support the formation of a more tightly packed hydrogen-bonding network around water molecules coordinated to Fe^{3+}. We have made detailed additions and revised the manuscript accordingly.

Fe^{3+} is a paramagnetic ion (S = 5/2), and its NMR chemical shift is dominated by contact and pseudocontact shifts, rather than the conventional diamagnetic hydrogen-bonding effects. Upfield shifts typically indicate that the unpaired electron spin density is transferred through chemical bonds to protons, causing the protons to experience a negative Fermi contact term. Therefore, the chemical shifts in aqueous Fe^{3+} solutions result from a combined effect of hydrogen bonding and paramagnetic interactions. We have carefully reconsidered and revised the description of the ^{1}H NMR chemical shifts. In addition, we performed 2D DOSY NMR, which showed a decrease in the DOSY diffusion coefficient upon addition of Fe^{3+}, indicating that Fe^{3+} induces water molecule aggregation into hydrated clusters. We have carefully revised and supplemented our discussion of the ^{1}H NMR as follows.

Page 16, line 306: First, low concentrations of Fe^{3+} forcibly strip water molecules to form the local hydration shells (0.2–0.4 mM). Although local hydrogen bonds are formed, this solvation process disrupts the original bulk water H-bond network. The

strong local H-bond network formed by the Fe^{3+} hydrated sphere ($[Fe(OH)(H_2O)_5]^{2+}$) is masked by paramagnetic effects, causing the overall NMR signal to appear as dispersed bulk water shifted toward upfield. As concentration increases (0.4–2 mM), more Fe^{3+} hydrated spheres form, and the strong electron-withdrawing effect within these spheres propagates through chemical bonds to the surface coordinated water molecules, weakening their O-H bonds. This allows the coordinated water molecules to form tighter H-bond network with outer water molecules, shifting the NMR signal toward downfield. Additionally, due to the paramagnetism of Fe^{3+}, water exchange within the hydrated spheres becomes slow, and multiple Fe^{3+} paramagnetic centers accumulate under the strong H-bond network. According to nuclear magnetic relaxation theory, this leads to a shortened transverse relaxation time, resulting in broadening of the NMR peak shapes.

Additionally, 2D DOSY NMR also indicates the rigidity of this strong H-bond network (Figure S21). After adding Fe^{3+}, the diffusion coefficient decreases, indicating slower water exchange and the formation of a more tightly bound H-bond network [29].

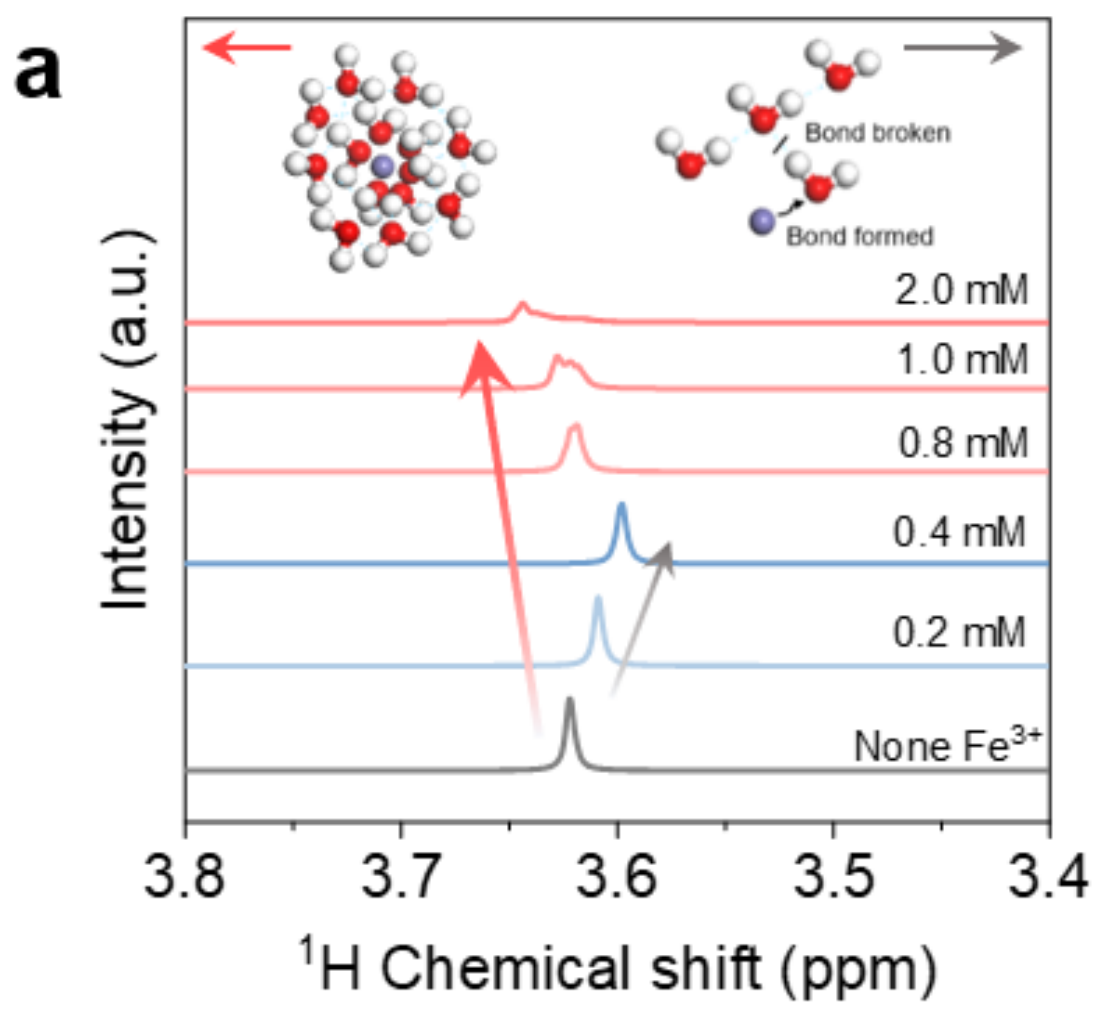

Figure 4. *In situ* spectroscopic analysis of hydrogen-bond network in the Fe^{3+}/BDD system. a. ^1H NMR chemical shifts for water at different Fe^{3+} concentration.

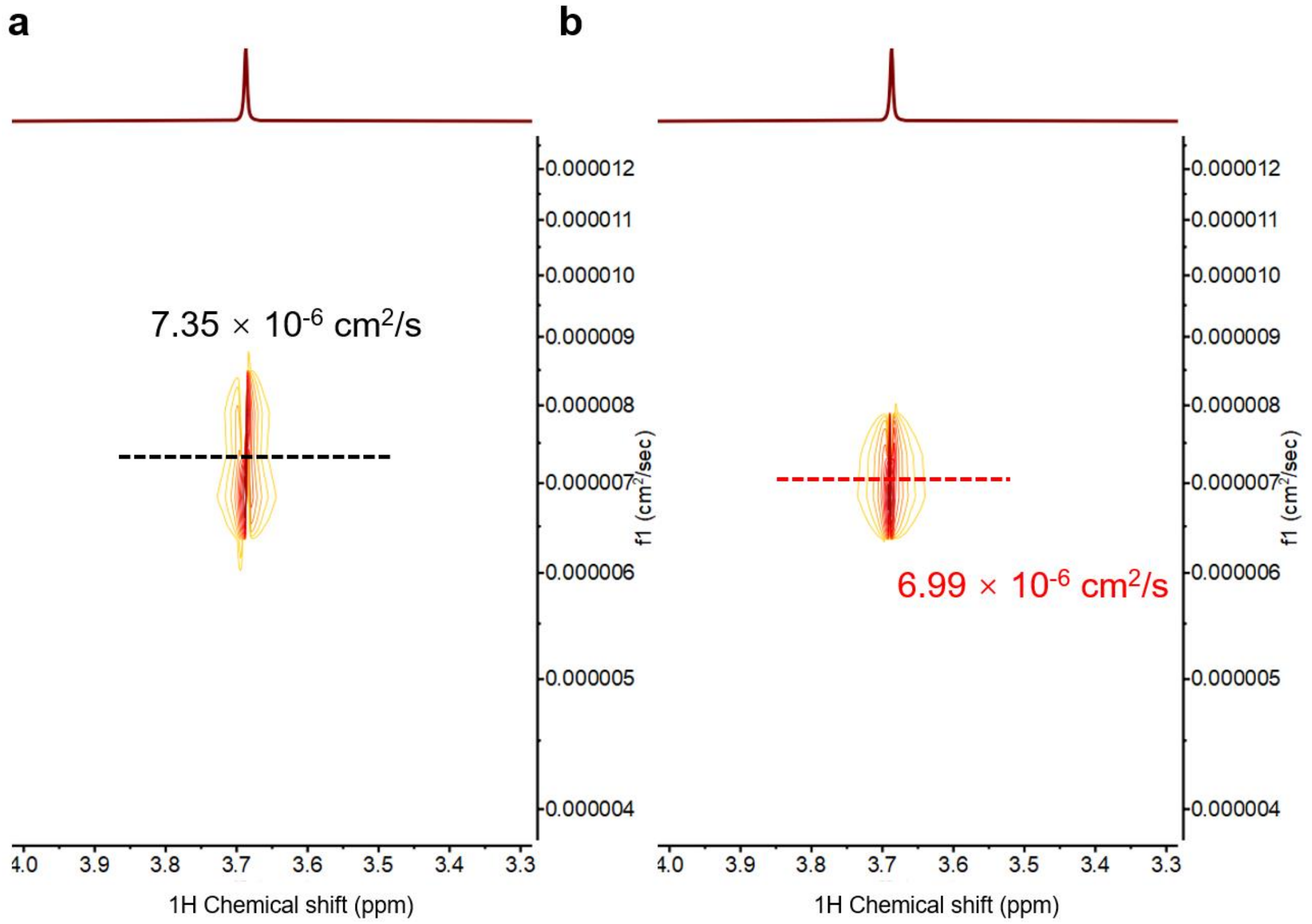

Figure S21. a. and b. 2D DOSY NMR of H_2O/DMSO solutions without and with Fe^{3+} addition.

3.2 The claim that the peaks in Fig. 4d undergo a pronounced red shift (line 304-306) with increasing Fe^{3+} concentration is not sufficiently supported by the presented data. The 0.6-1.0 mM Fe^{3+} peaks appear to show a blue shift rather than a red shift.

Response: We extend our heartfelt gratitude to you for the valuable suggestion. We appreciate the opportunity to clarify the spectral shift behavior across the entire Fe^{3+} concentration range.

Regarding the redshift phenomenon of the 4-HB-H_2O peak at ~3200 cm^{-1} in Figure 4d with the increase of Fe^{3+} concentration, we re-examined the original Raman spectral data and the peak position fitting results. The clarification is as follows.

Redshift trend across the entire concentration range: We used Gaussian multi-peak fitting to precisely extract the peak positions of the ~3200 cm^{-1} water peak (4-HB-H_2O) at Fe^{3+} concentrations from 0 to 1.0 mM. The results show that from 0 mM to 1.0 mM, the peak positions continuously shift to lower wavenumbers (e.g.,

approximately 3228.0 cm^{-1} at 0 mM, 3224.6 cm^{-1} at 0.6 mM, 3222.8 cm^{-1} at 0.8 mM, and 3221.3 cm^{-1} at 1.0 mM), indicating a redshift.

Due to its high charge density and strong hydration ability, Fe^{3+} exerts intense electrostatic interactions with surrounding H_2O. The H_2O around it become polarized and form a H-bond network that is stronger and more ordered than typical H_2O–H_2O interactions. As the hydrogen bonds between water molecules strengthen, the O–H bond length increases, thereby reducing the force constant of the O–H bond. This weakening of the O–H bond lowers the energy required for its stretching vibration, resulting in a decrease in vibrational frequency, which manifests in the Raman spectrum as a redshift of the characteristic peak. Thus, the redshift of the ~3200 cm^{-1} 4-HB-H_2O peak is supported both by experimental data and theoretical principles. To address this, we have revised the manuscript to state more precisely.

Page 17, line 341: From Figure 4d, with increasing Fe^{3+} concentration, the characteristic band at ~3200 cm^{-1} assigned to 4-HB-H_2O exhibits both enhanced intensity and a pronounced red shift (from 3228.0 cm^{-1} at 0 mM to 3221.3 cm^{-1} at 1.0 mM), indicating strengthened hydrogen bonding.

Thank you again for your detailed review. We believe that the supplementary data and explanations provided above can fully address your concerns.

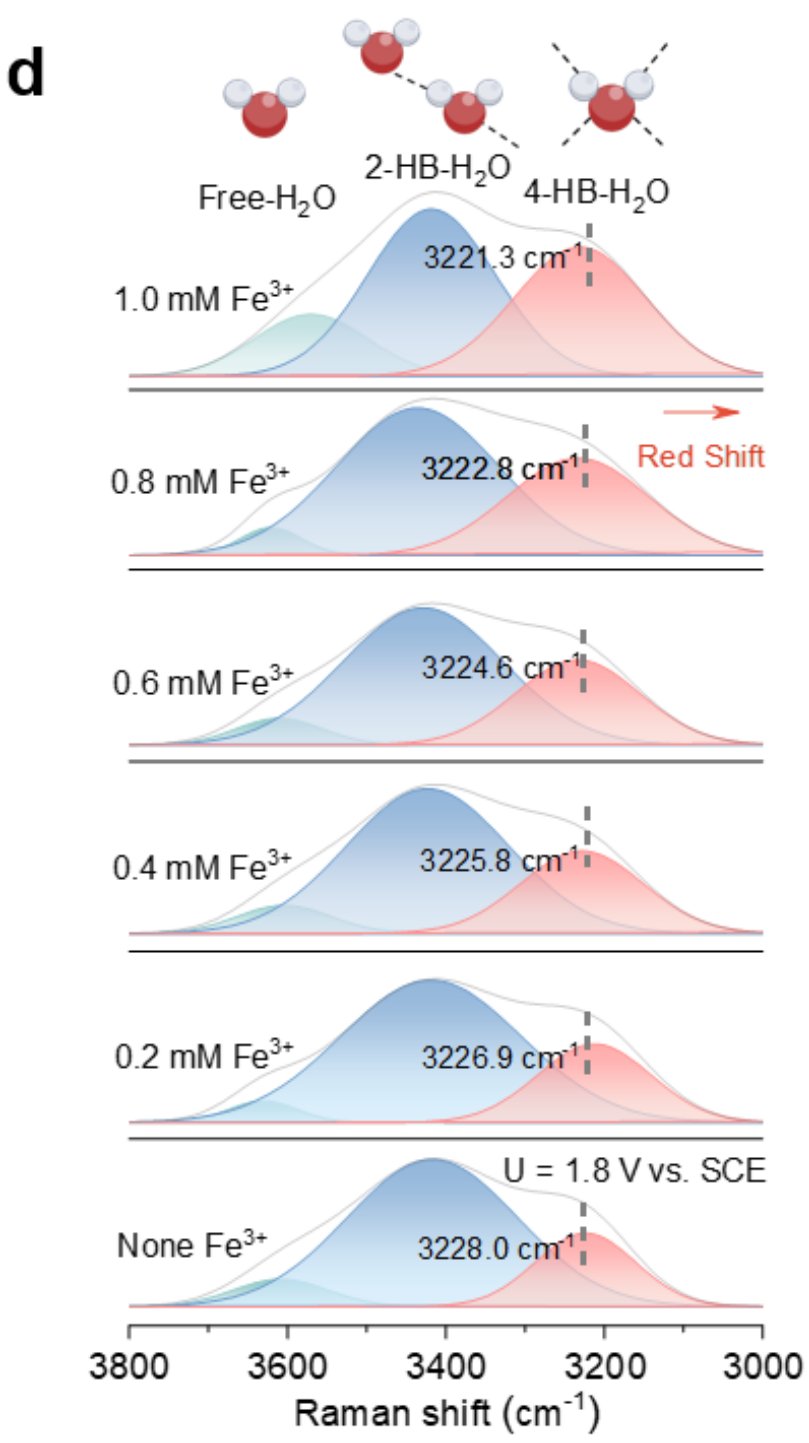

Figure 4. *In situ* spectroscopic analysis of hydrogen-bond network in the Fe^{3+}/BDD system. d. *In situ* Raman spectra at 1.8 V vs. SCE with Fe^{3+} concentrations ranging from 0 to 1 mM. (4-HB-H_2O: ~3200 cm^{-1}, 2-HB-H_2O: ~3400 cm^{-1} free H_2O: ~3600 cm^{-1})

3.3 line 303-306: the authors state, based on in situ Raman spectroscopy, that with increasing Fe^{3+} concentration, the characteristic band at ~3200 cm^{-1} assigned to 4-HB-H_2O shows enhanced intensity and a pronounced red shift, which is then interpreted as strengthened hydrogen bonding, further weakening and polarization of O–H bonds, and a “pre-activated” water state.

This description needs to be reformulated more rigorously.

Response: We sincerely appreciate your constructive suggestion. Based on the results obtained from *in situ* electrochemical Raman spectroscopy and Gaussian fitting, the proportion of 4-HB-H_2O has increased, indicating an enhancement of hydrogen bonds between water molecules; the fitting peak of 4-HB-H_2O has redshifted, suggesting that the O-H bonds of water molecules have been weakened. Therefore, we have carefully revised the relevant paragraphs (original line 303-306 and their context), and the revised content is as follows.

Page 18, line 345: In general, a redshift of the O–H stretching band is associated with a longer and weaker O–H bond, which often results from stronger hydrogen bonding interactions [39]. Meanwhile, the increased intensity of this band suggests a higher population of the 4-HB-H_2O species. Therefore, the observed spectral changes are consistent with a strengthening of the interfacial H-bond network and a weakening of the O–H covalent bonds induced by the presence of Fe^{3+} species. This weakening and polarization of O–H bonds may facilitate subsequent bond cleavage, i.e., a “pre-activated” state of water.

We more rigorously establish the correlation between spectral changes and the strength of hydrogen bonds/O–H bonds, clearly stating that "redshift is usually associated with longer and weaker O–H bonds, which often results from stronger hydrogen bonding", avoiding direct causal leaps. Ultimately, we retain the core conclusion but soften the tone: changing "place water molecules in a pre-activated

state favorable for bond cleavage" to "may facilitate subsequent bond cleavage, i.e., a 'pre-activated' state of water. "

We believe that the above-mentioned modifications are more in line with the rigorous interpretation norms of spectral data, while still providing a strong argument for the core scientific phenomenon. Thank you again for your meticulous and professional review of this article.

4. Comment 4: Important operational parameters are insufficiently monitored or discussed. For both the MIT oxidation and potential environmental application, the Fe^{3+} concentration and pH are not discussed. The added Fe concentration of from 11.2 to 56 mg/L is not negligible for a water-treatment system and may be strongly influenced by the reaction pH. The authors are therefore encouraged to provide additional data on the evolution of Fe concentration and pH during the reaction.

Response: We extend our heartfelt gratitude to you for the very considerate comment. Your mention of the changes in Fe^{3+} concentration and the influence of pH is indeed key issues that is given close attention in actual sewage treatment. We have supplemented additional experiments on the change of Fe^{3+} concentration over reaction time and the effect of different pH on MIT degradation in the Fe^{3+}/BDD system. The specific revisions are as follows.

(1). The concentration of Fe^{3+} during the reaction process.

During the 30-minute beaker experiment and throughout the 500-hour continuous flow operation, we monitored the Fe^{3+} and total Fe concentration (by the o-phenanthroline colorimetric method). The Fe^{3+} concentration remained stable throughout the 30-minute reaction process in the beaker experiment (Figure S37). During the entire 500-hour continuous operation, the total Fe concentration remained stable, with a decrease of less than 5% from the initial 0.2 mM (11.2 mg/L) (Figure 6d). This minor loss was mainly due to physical entrainment rather than chemical precipitation, confirming that Fe^{3+} was not consumed during the degradation process but rather acted as a cyclic substance, promoting interfacial water dissociation and the generation of high-valent iron species. We have made supplements in the revised manuscript.

Page 27, line 523: After the Fe^{3+} is oxidized to Fe(H) at the anode, its reduction product is Fe^{3+}, enabling a rapid Fe^{3+}-Fe(H)-Fe^{3+} cycle (Figure S37).

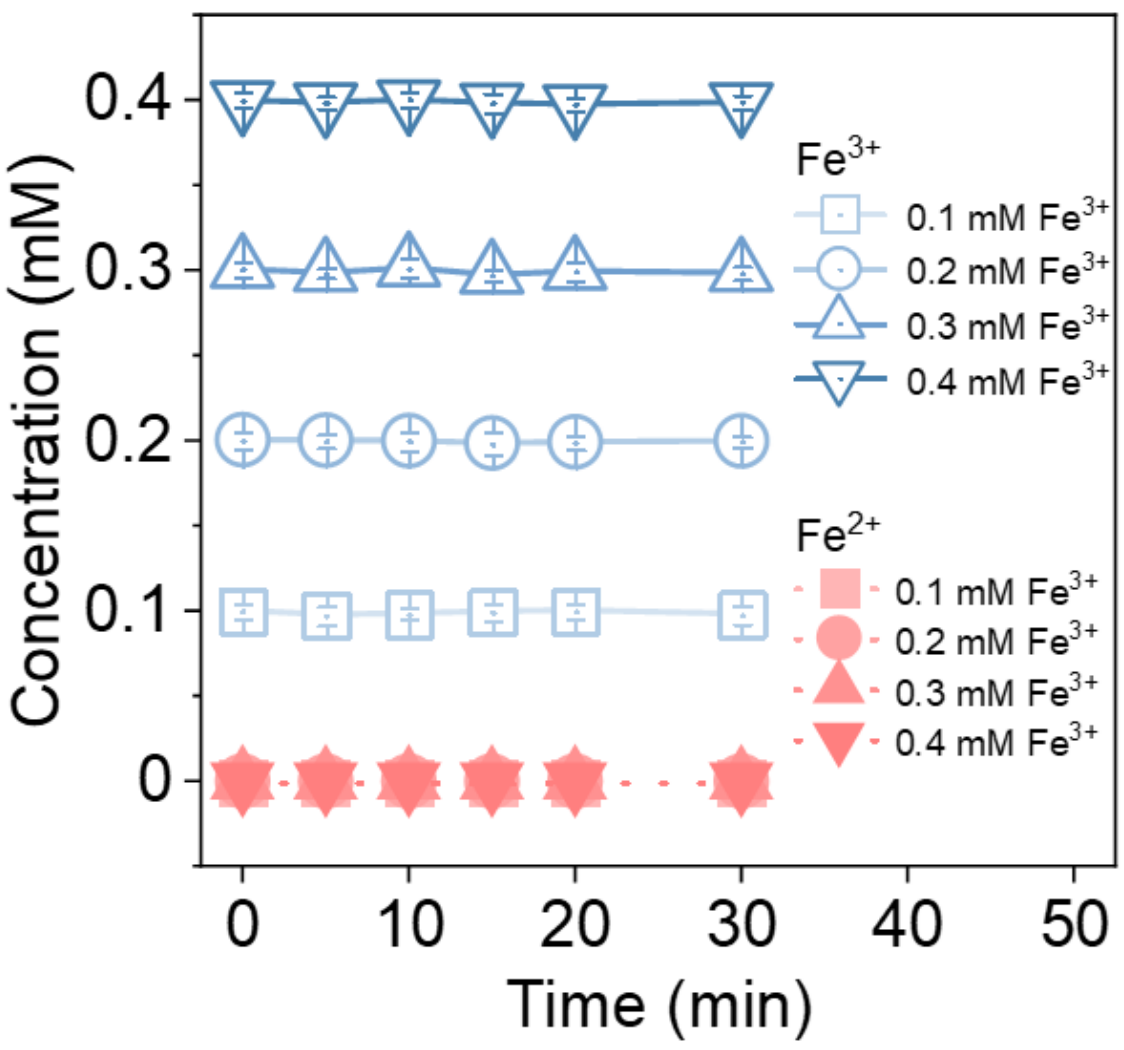

Figure S37. Variation of Fe^{2+} and Fe^{3+} concentrations in the Fe^{3+}/BDD system with reaction time. (Experimental conditions: [Na_2SO_4] = 50 mM, current density = 7.5 mA cm^{-2}.)

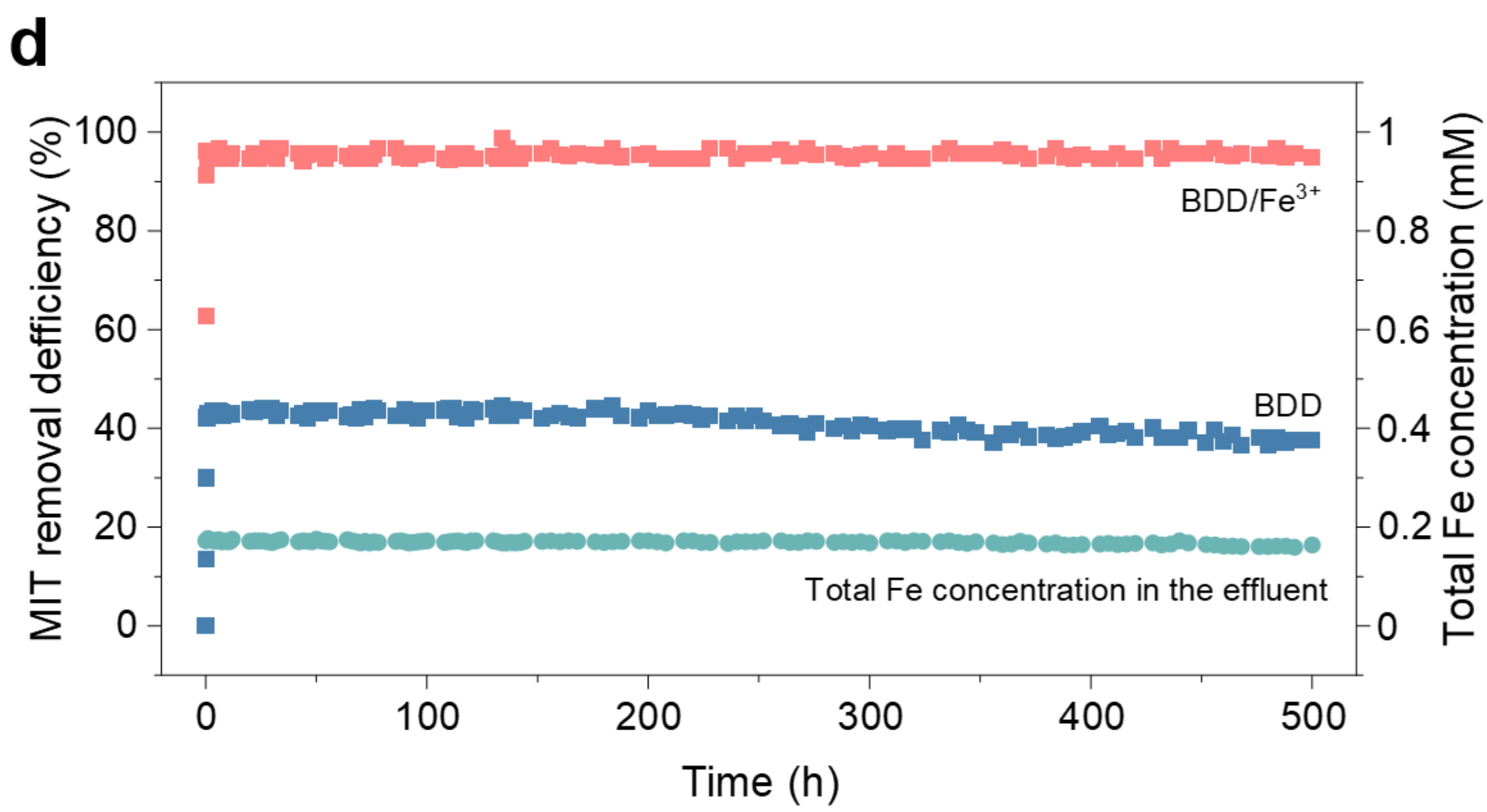

Figure 6. Practical application investigations of Fe^{3+}/BDD system. d. Stability tests of different systems.

(2). The influence of the initial pH and the monitoring of pH during the reaction process.

The MIT degradation efficiency of the Fe^{3+}/BDD system declined progressively with increasing pH (1-5). At pH 1, complete degradation was achieved within 5 minutes, whereas at pH 5, complete removal was obtained after 30 min. Across this range, Fe^{3+} exists in hydrated forms such as $[Fe(H_2O)_6]^{3+}$ and $[Fe(OH)(H_2O)_5]^{2+}$. On one hand, Fe^{3+} reconstructs the hydrogen bond network of interfacial water molecules through strong hydration, promoting the water decomposition reaction on the BDD surface to generate more •OH. On the other hand, it generates high-valent iron species under BDD anodic oxidation, providing an additional oxidation pathway.

Within the pH range of 6–10, MIT removal efficiency reached approximately 80% within 30 minutes, and the removal rate no longer decreased with increasing pH. The synergistic effect of •OH and Fe(H) enables the Fe^{3+}/BDD system to efficiently remove MIT across a wide pH range of 1–10. Within the typical pH range of most municipal wastewater and industrial effluents (pH 6–10), the degradation efficiency of the Fe^{3+}/BDD system remains at a high level, indicating that strict pH pretreatment is unnecessary. The Fe^{3+}/BDD system is a pH-tolerant technology suitable for direct treatment of real wastewater, effectively degrading MIT and other emerging organic pollutants. We have made supplements in the revised manuscript.

Page 26, line 510: The Fe^{3+}/BDD system achieves stable MIT degradation efficiency (more than 80% in 30 min) across pH 1–10 (Figure S36), demonstrating broad pH tolerance.

We thank you again for these insightful suggestions, which have significantly improved the practical relevance and completeness of our manuscript.

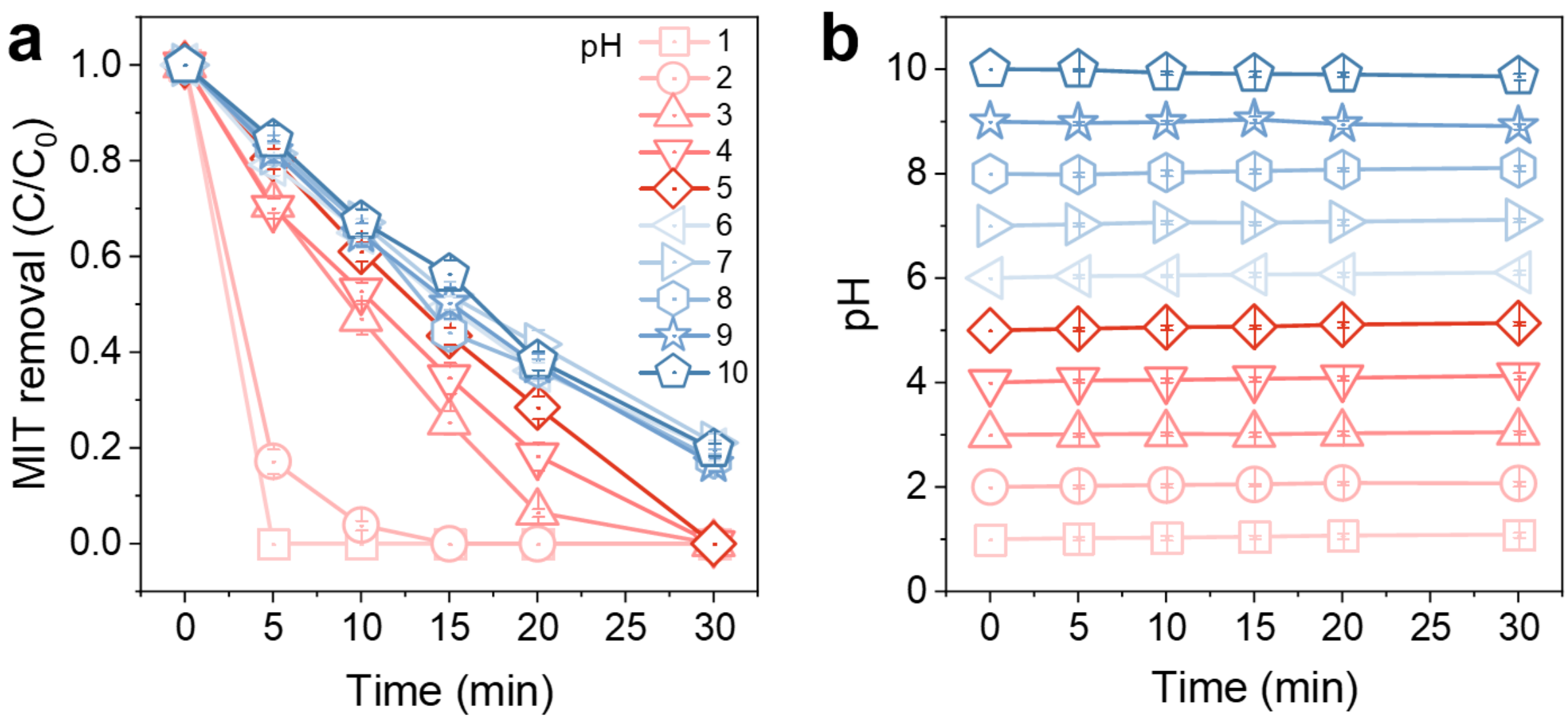

Figure S36. a. Effect of Fe^{3+}/BDD system on the removal efficiency of MIT under different initial pH conditions. **b.** The variation of pH with reaction time. (Experimental conditions: [Na_2SO_4] = 50 mM, [Fe^{3+}] = 0.2 mM, current density = 7.5 mA cm^{-2})

5. Comment 5: The possibility of an ozone-mediated pathway is not adequately excluded. This is a major mechanistic concern. Because the system employs a BDD anode under strongly anodic conditions, the possible formation of ozone should be carefully considered (https://doi.org/10.1016/j.jechem.2025.05.016; https://doi.org/10.1021/acs.est.2c05290). If ozone is generated electrochemically, then Fe species may further activate ozone and produce additional reactive oxygen species, mainly •OH (https://doi.org/10.1016/j.watres.2024.121907). If so, the observed enhancement may rise from an ozone-iron interaction pathway, rather than from hydrogen-bond reconfiguration.

Response: We thank you for raising this important mechanism concern. It is necessary to rule out the possibility of ozone-mediated pathways. While BDD anode can indeed generate ozone under specific conditions, and if ozone is present, Fe^{3+} might catalyze its decomposition to produce additional •OH, potentially interfering with our mechanism conclusions regarding the reconstruction of interfacial H-bond networks. Therefore, we supplemented a series of ozone detection experiments and provide the following detailed evidence indicating that almost no ozone was detected under the experimental conditions of this study. The results indicate that any pathways involving interactions between ozone and Fe^{3+} can be excluded.

Ozone Detection Method. We employed the indigo disulfonate (IDS) spectrophotometric method, which is widely recognized as a standard approach for trace ozone quantification[40]. This method is based on the rapid reaction between ozone and IDS at 610 nm. The IDS method has been successfully used in previous studies on BDD-based electrochemical ozone generation[41].

Results – less than 0.01 mg/L ozone (Figure S12). At 610 nm, all samples showed absorbance identical to the IDS-only blank—no decrease was observed at any time point (5–30 min). This confirms undetectable ozone (< 0.01 mg/L), because under our conditions (7.5 mA cm^{-2}, 50 mM Na_2SO_4), anodic water oxidation favors •OH generation over ozone. Ozone generation via BDD occurs only under specific conditions and is competitive with very low Faradaic efficiency. Our system uses 50 mM Na_2SO_4, which is a common supporting electrolyte and does not have a promoting effect on ozone generation. The reported rate constant for Fe^{3+} catalyzing the decomposition of ozone into •OH indicates that when the ozone concentration is below 0.1 mg/L, the •OH concentration produced is insignificant compared to the direct electrochemical generation of •OH.

We thank you again for this insightful suggestion, which has allowed us to further strengthen the mechanism rigor of our study. We have made the following additions in the revised manuscript.

Page 10, line 184: Moreover, no ozone was detected in the BDD anode oxidation system under our experimental conditions (Figure S12).

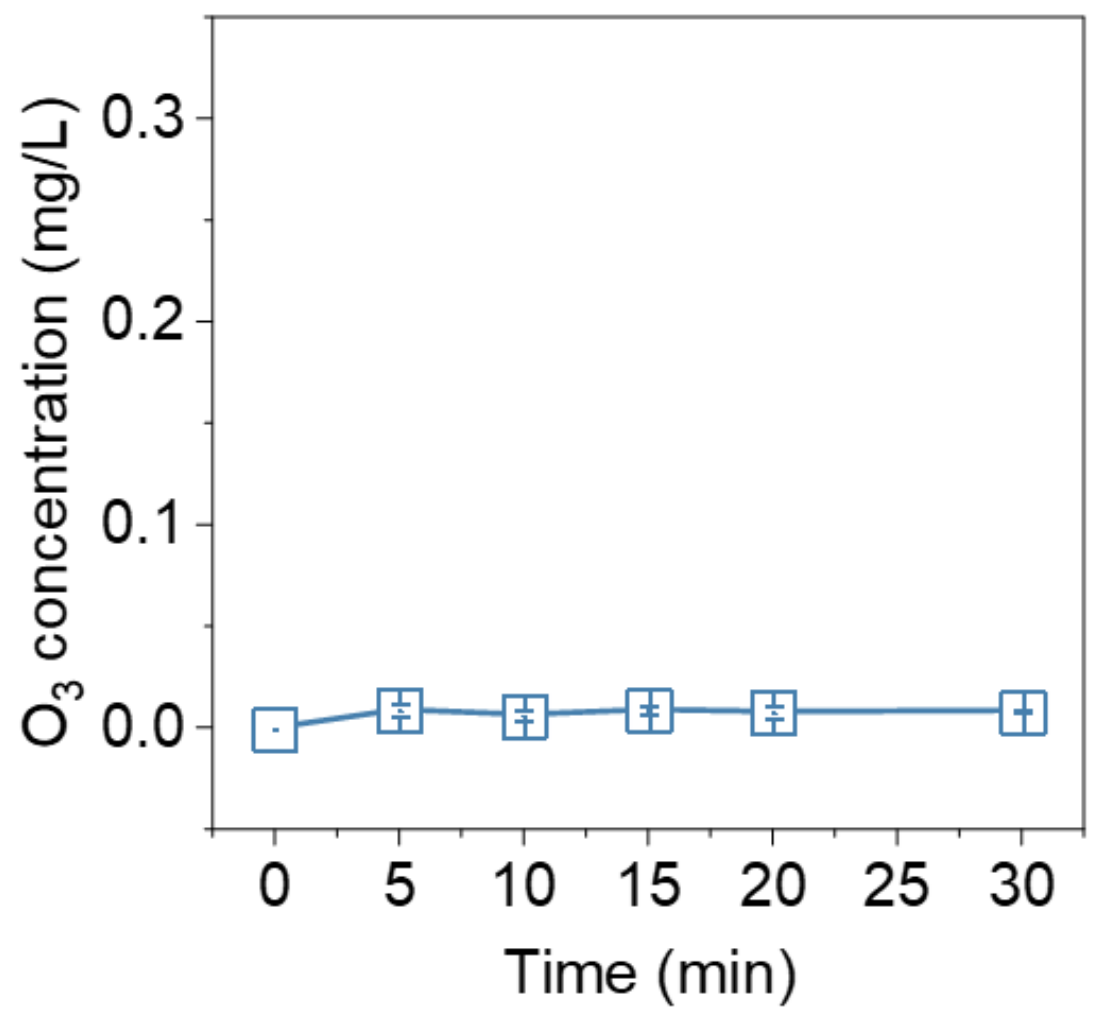

Figure S12. The variation of ozone generation concentration in the BDD system with reaction time was detected by the indigo method. (Experimental conditions: [Na_2SO_4] = 50 mM, current density = 7.5 mA cm^{-2})

6. Comment 6: In Lines 371-380, the decrease in H_2O_2 concentration (Fig 5f) after Fe^{3+} addition cannot by itself support the conclusion that Fe^{3+} suppresses •OH recombination into H_2O_2.

Response: We sincerely appreciate your great suggestion. We conducted corresponding control experiments of Fe^{3+}/H_2O_2, Fe(VI)/H_2O_2 and Fe(VI)/Fe^{3+}/H_2O_2. The results proved that under the conditions of this study, neither Fe^{3+} nor Fe(VI) decomposes H_2O_2, further confirming our proposed viewpoint that the decrease in H_2O_2 concentration is attributed to the suppression of •OH dimerization. The specific evidence and explanations are as follows.

(1). Direct control experiments exclude alternative H_2O_2 consumption pathways.

The result presented in detail as Figure S25, a control experiment was conducted at an initial pH of approximately 3 without an applied potential. In this experiment, the concentration of Fe^{3+} was 0.2 mM, the addition of Fe(VI) was 2 mg/L, and the concentration of H_2O_2 was 0.08 mM (consistent with the highest H_2O_2 concentration in Figure 5f). Within 30 minutes, among the three systems, only the system with Fe(VI) added showed a degradation effect of less than 20% on MIT. Moreover, the concentration of H_2O_2 remained largely unchanged in all three systems. This evidence is sufficient to demonstrate that Fe^{3+} and high-valent iron species cannot generate active oxidants that can significantly decompose H_2O_2 (Figure S25b).

Thus, direct chemical decomposition of H_2O_2 by Fe^{3+} or high-valent iron species is negligible. The observed H_2O_2 decrease in Figure 5f cannot be attributed to these pathways. We add the following content to the manuscript.

Furthermore, during the degradation of MIT by the Fe^{3+}/BDD system, no significant consumption of the by-product H_2O_2 was observed within 5 minutes after the power was cut off (Figure S26). This further indicates that there are no other pathways for the consumption of H_2O_2 in this system.

Page 24, line 457: When the pH is approximately 3, Fe^{3+} and Fe(H) cannot effectively activate H_2O_2 to generate strong oxidizing active species. The removal rates of MIT in the Fe^{3+}/H_2O_2, Fe(VI)/H_2O_2 and Fe(VI)/Fe^{3+}/H_2O_2 systems are all less than 20% (Figure S25).

Page 24, line 461: In addition, during the degradation of MIT by the Fe^{3+}/BDD system, no significant consumption of the by-product H_2O_2 was observed within 5 minutes after the power supply was cut off (Figure S26).

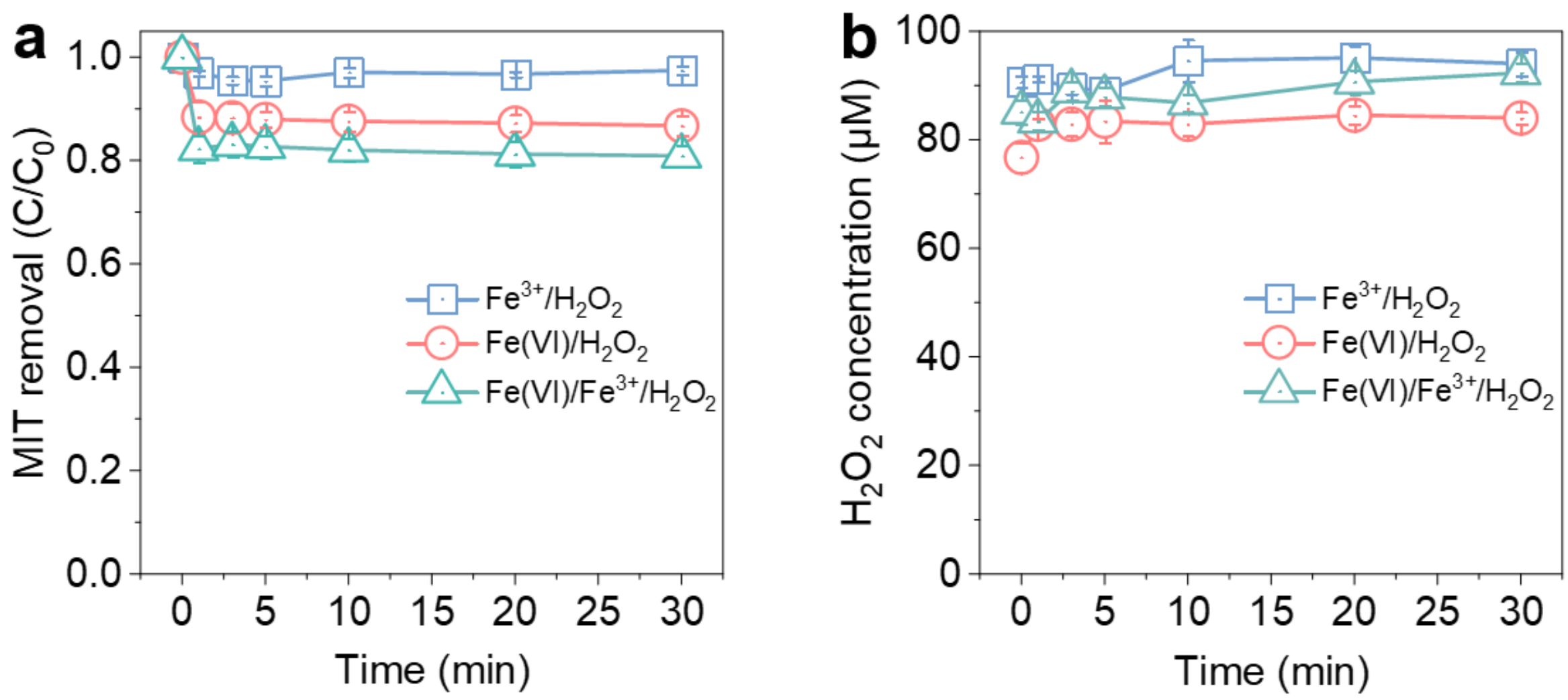

Figure S25. a. The removal effect of MIT by Fe^{3+}/H_2O_2, Fe(VI)/H_2O_2 and Fe(VI)/Fe^{3+}/H_2O_2, **b.** the variation of H_2O_2 concentration with time in Fe^{3+}/H_2O_2, Fe(VI)/H_2O_2 and Fe(VI)/Fe^{3+}/H_2O_2 systems. (Experimental conditions: [MIT] = 20 μM, [Fe^{3+}] = 0.2 mM, [Fe(VI)] = 2 mg/L, [H_2O_2] = 80 μM, initial pH = 3)

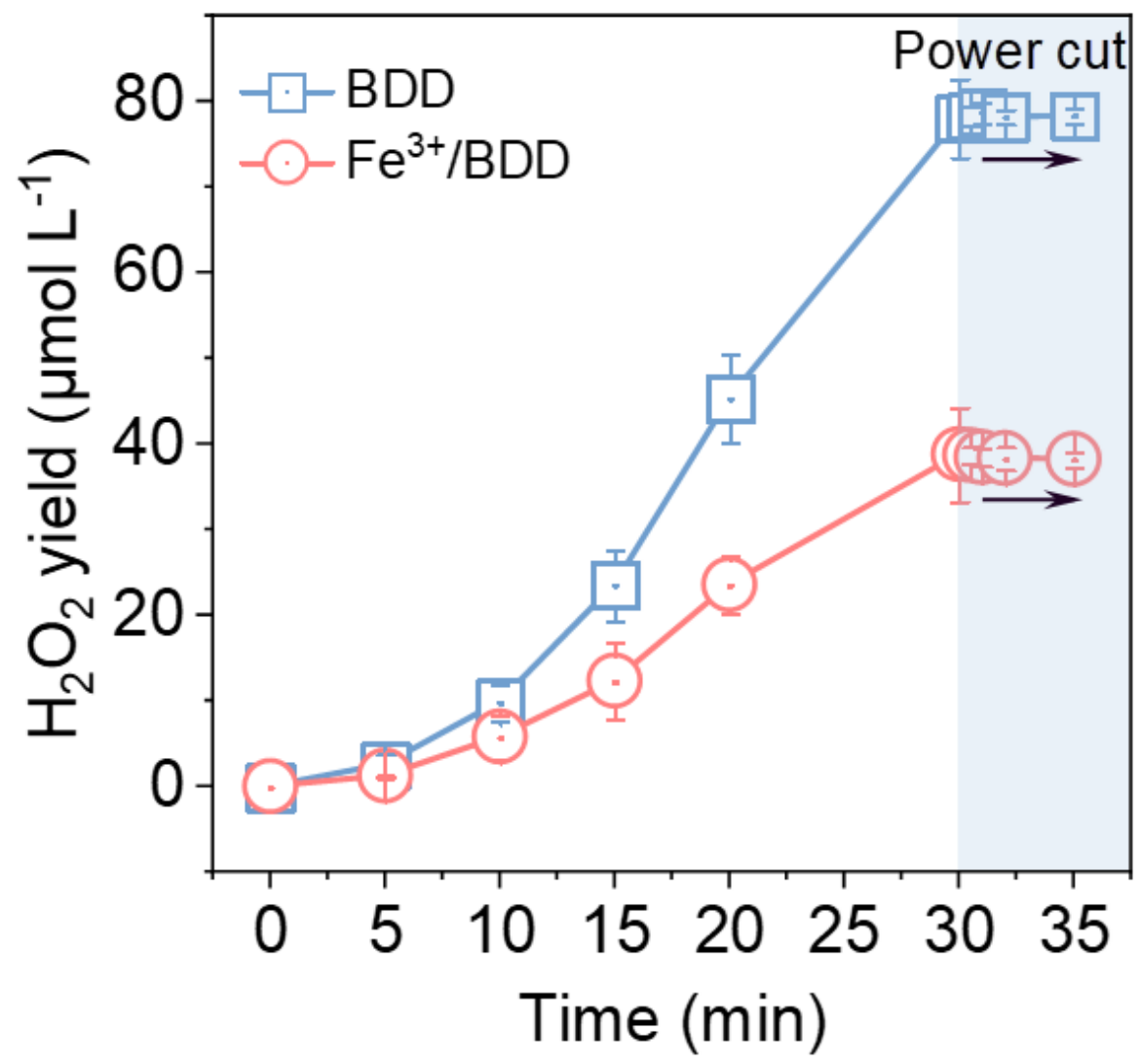

Figure S26. The continuous removal and the changes in H_2O_2 concentration after power cut. (Experimental conditions: [Na_2SO_4] = 50 mM, [Fe^{3+}] = 0.2 mM, current density = 7.5 mA cm^{-2})

(2). Inverse correlation between •OH concentration and H_2O_2 level.

Using the NB probe method, we found that Fe^{3+} increased steady-state •OH concentration sevenfold—but H_2O_2 decreased markedly. Since •OH recombination is the main pathway for H_2O_2 formation, this inverse response indicates Fe^{3+} directly inhibits recombination, not just •OH generation. Even with sevenfold higher •OH, H_2O_2 should rise if recombination were unaffected, its decline proves recombination is blocked. Under these Fe^{3+}/BDD conditions, no other pathways significantly consume H_2O_2. We thank the reviewer for the valuable comment, which helped us clarify the mechanism.

7. Comment 7: Line 187, "Fig. 1h" should presumably be corrected to "Fig. 2h".

Response: We sincerely appreciate you for catching this error. We have corrected "Figure 1h" to "Figure 2h" in Line 205 of the revised manuscript. The correction has been highlighted. We apologize for any confusion this may have caused. The revised content is as follows.

Page 10, Line 205: Through finite element simulation to model the concentration distribution of •OH and Fe(H) within the micron of the interface[20], COMSOL simulation visualizes the mass transfer of active species at the anode interface (Figure 2h and Text S10)[42].

8. Comment 8: Line 213, "This shift confirms that Fe^{3+} significantly the kinetics of interfacial water oxidation on the BDD anode." A missing verb.

Response: We appreciate you for carefully reading the manuscript and identifying the missing verb in Line 213. The correction has been made in the revised manuscript (Line 245) and is highlighted for easy reference. We apologize for this oversight.

Page 13, Line 245: This shift confirms that Fe^{3+} significantly expedites the kinetics of interfacial water oxidation on the BDD anode.

References

1. Zhu J*, et al.* Overlooked Role of Fe(IV) and Fe(V) in Organic Contaminant Oxidation by Fe(VI). *Environ. Sci. Technol.* **54**, 9702-9710 (2020).
2. Wang S*, et al.* High-Valent Iron Species (Fe(IV)/Fe(V)) for Selective Oxidation of Emerging Contaminants: Mechanisms, Applications, and Future Directions. *Environ. Sci. Technol.* **59**, 23005-23023 (2025).
3. Wang S, Deng Y, Shao B, Zhu J, Hu Z, Guan X. Three Kinetic Patterns for the Oxidation of Emerging Organic Contaminants by Fe(VI): The Critical Roles of Fe(V) and Fe(IV). *Environ. Sci. Technol.* **55**, 11338-11347 (2021).
4. Gao J, Zhao G, Shi W, Li D. Microwave activated electrochemical degradation of 2,4-dichlorophenoxyacetic acid at boron-doped diamond electrode. *Chemosphere* **75**, 519-525 (2009).
5. Song HR*, et al.* Electrochemical activation of persulfates at BDD anode: Radical or nonradical oxidation? *Water Res.* **128**, 393-401 (2018).
6. Chen P*, et al.* Shifts of surface-bound •OH to homogeneous •OH in BDD electrochemical system via UV irradiation for enhanced degradation of hydrophilic aromatic compounds. *Chemosphere* **291**, (2022).
7. Wang R*, et al.* Electrochemical system with moderate ozone generation and catalytic enhancement for efficient organic wastewater treatment. *Water Res.* **287**, (2025).
8. Yang B*, et al.* Electrochemical mineralization of perfluorooctane sulfonate by novel F and Sb co-doped Ti/SnO_2 electrode containing Sn-Sb interlayer. *Chem. Eng. J.* **316**, 296-304 (2017).
9. Jin B, Cheng S, Li L, Li H, Zhou Y, Chen H. Self-supporting three-dimensional CuNi–Sb–SnO_2 anode with ultra-long service life for efficient removal of antibiotics in wastewater. *Chemosphere* **365**, (2024).
10. Chen Y*, et al.* Cu/Mn-mediated electron shuttle and high-valent metals enhance hydroxyl radicals production during the electrochemical oxidation on the CuMn-Sb-SnO_2 electrode. *J. Hazard. Mater.* **477**, (2024).
11. Wang L, Lu J, Li L, Wang Y, Huang Q. Effects of chloride on electrochemical degradation of perfluorooctanesulfonate by Magnéli phase Ti_4O_7 and boron doped diamond anodes. *Water Res.* **170**, (2020).
12. Song R*, et al.* Highly Efficient Degradation of Persistent Pollutants with 3D Nanocone TiO_2-Based Photoelectrocatalysis. *J. Am. Chem. Soc.* **143**, 13664-13674 (2021).
13. Hu Y*, et al.* Interface and Defect Engineering in 3D Co_3O_4-O_V/TiO_2 to Boost Simultaneous Removal of BPA and Cr(VI) upon Photoelectrocatalytic/Peroxymonosulfate (PEC/PMS) System. *Adv. Funct. Mater.* **35**, (2024).
14. Lin H*, et al.* Defect Engineering on a Ti_4O_7 Electrode by Ce^{3+} Doping for the Efficient Electrooxidation of Perfluorooctanesulfonate. *Environ. Sci. Technol.* **55**, 2597-2607 (2021).
15. Steter JR, Brillas E, Sirés I. Solar photoelectro-Fenton treatment of a mixture of parabens spiked into secondary treated wastewater effluent at low input current. *Appl. Catal. B-Environ.* **224**, 410-418 (2018).
16. Ding J, Bu L, Zhao Q, Kabutey FT, Wei L, Dionysiou DD. Electrochemical activation of persulfate on BDD and DSA anodes: Electrolyte influence, kinetics and mechanisms in the degradation of bisphenol A. *J. Hazard. Mater.* **388**, (2020).
17. Buxton GV, Greenstock CL, Helman WP, Ross AB. Critical Review of rate constants for reactions of hydrated electrons, hydrogen atoms and hydroxyl radicals (·OH/·O^- in Aqueous Solution. *J. Phys. Chem. Ref. Data* **17**, 513-886 (1988).
18. Li N*, et al.* A critical review on correlating active sites, oxidative species and degradation routes with persulfate-based antibiotics oxidation. *Water Res.* **235**, (2023).
19. Russo D*, et al.* Ultrafast photodegradation of isoxazole and isothiazolinones by UV_{254} and UV_{254}/H_2O_2 photolysis in a microcapillary reactor. *Water Res.* **169**, (2020).
20. Kang Y*, et al.* Unveiling the spatially confined oxidation processes in reactive electrochemical membranes. *Nat. Commun.* **14**, (2023).
21. Liu M*, et al.* Enhanced electrocatalytic CO_2 reduction via field-induced reagent concentration. *Nature* **537**, 382-386 (2016).

22. Chen Y, Zhang G, Ji Q, Lan H, Liu H, Qu J. Visualization of Electrochemically Accessible Sites in Flow-through Mode for Maximizing Available Active Area toward Superior Electrocatalytic Ammonia Oxidation. *Environmental Science & Technology* **56**, 9722-9731 (2022).
23. Central Electricity Generating Board BNL, Berkeley, Gloucestershtre, GL 13 9PB. Spectrophotometric Determination of Hydrogen Peroxide Using Potassium Titanium(IV) Oxalate. *Analyst* **105**, 5 (1980).
24. Huang N*, et al.* Removal of methylisothiazolinone biocide from wastewater by VUV/UV advanced oxidation process: Kinetics, mechanisms and toxicity. *J Environ Manage* **315**, (2022).
25. Li B-T*, et al.* Adsorption of Isothiazolone Biocides in Textile Reverse Osmosis Concentrate by Powdered Activated Carbon. *Water* **10**, (2018).
26. Chen M*, et al.* Kinetic, mechanism and mass transfer impact on electrochemical oxidation of MIT using Ti-enhanced nanotube arrays/SnO2-Sb anode. *Electrochimica Acta* **323**, (2019).
27. Nowak M, Zawadzka K, Lisowska K. Occurrence of methylisothiazolinone in water and soil samples in Poland and its biodegradation by Phanerochaete chrysosporium. *Chemosphere* **254**, (2020).
28. Paijens C*, et al.* Urban pathways of biocides towards surface waters during dry and wet weathers: Assessment at the Paris conurbation scale. *Journal of Hazardous Materials* **402**, (2021).
29. Ma K, Zhao L. The Opposite Effect of Metal Ions on Short-/Long-Range Water Structure: A Multiple Characterization Study. *International Journal of Molecular Sciences* **17**, (2016).
30. Gomes RJ, Kumar R, Fejzić H, Sarkar B, Roy I, Amanchukwu CV. Modulating water hydrogen bonding within a non-aqueous environment controls its reactivity in electrochemical transformations. *Nature Catalysis* **7**, 689-701 (2024).
31. Rui Zhao QW, Yancai Yao, Jie Dai, Xiang--Kui Gu, Lizhi Zhang. Pd single atoms guided proton transfer along an interfacial hydrogen bond network for efficient electrochemical hydrogenation. *Science Advances*, (2025).
32. An W, Zhong Y, Luo L, Zhou D, Sun X. Electrolyte engineering for hydrogen energy. *Joule* **10**, (2026).
33. McBeath ST, Zhang Y, Hoffmann MR. Novel Synthesis Pathways for Highly Oxidative Iron Species: Generation, Stability, and Treatment Applications of Ferrate(IV/V/VI). *Environ Sci Technol* **57**, 18700-18709 (2023).
34. Wang S, Lin Y, Shao B, Dong H, Ma J, Guan X. Selective Removal of Emerging Organic Contaminants from Water Using Electrogenerated Fe(IV) and Fe(V) under Near-Neutral Conditions. *Environmental Science & Technology* **57**, 9332-9341 (2023).
35. Kong D*, et al.* Inhibition of hydrogen evolution reaction during iron electrodeposition from sulfate system by reducing hydrogen bonding activity in water. *International Journal of Hydrogen Energy* **191**, (2025).
36. Sun Z*, et al.* Reconstructing Short-Hydrogen-Bond Network via Hexametaphosphate Boosts Chloride-Ion Shielding Effect Enhancement in Seawater Electrolysis. *Advanced Functional Materials* **36**, (2025).
37. Luo W*, et al.* Bioinspired Catalyst/Electrolyte Interfacial Hydrogen-Bond Network Engineering toward Proton Transfer Acceleration for Glycerol Electrooxidation. *Journal of the American Chemical Society* **148**, 11099-11111 (2026).
38. Qi X, Ding L, Jian C, Liu R, Liu N, Qu D. Ferrate(VI) oxidation of substituted nitrobenzene compounds: Kinetics, degradation, and oxidized products. *Chemical Engineering Journal* **488**, (2024).
39. Kolesov B. Hydrogen Bonds: Raman Spectroscopic Study. *International Journal of Molecular Sciences* **22**, (2021).
40. HOIGNI~ HBaJ. DETERMINATION OF OZONE IN WATER BY THE INDIGO METHOD. *Water Res* **15**, 7 (1981).
41. Lee W, Kim S, Cho K. Quantitative Evaluations on Ozone Evolution Electrocatalysts by Scanning Electrochemical Microscopy for Oxidative Water Treatment. *Environmental Science & Technology* **57**, 18529-18537 (2022).
42. Chen M, Zhang X, Qu J. Unveiling Scale-Design Principle at Electrical Confinement

Materials for Water Purification. *Advanced Materials* **37**, (2025).

Responses to Reviewers' Comments

Manuscript ID: NCOMMS-26-019794A

Title: "Reconfiguring Interfacial Hydrogen-Bond Networks to Enhance Anodic Oxidation for Efficient Wastewater Treatment"

Author(s): Tong Liu[#], Shuang Zhong[#], Xiaotong Liang, Hongrui Zhu, Ye Du* & Bo Lai*

We would like to thank you for the valuable comments, which have helped us improve the quality and rigor of the manuscript. The manuscript has been carefully revised with point-to-point responses to comments as listed below. The revised portions were marked in red in the revised manuscript, and we hope the revision can be satisfactory.

<u>Reviewer #3</u> (Remarks to the Author):

I would like to thank the authors for their careful and thorough revision of the manuscript. The revised version has been substantially improved in both clarity and rigor through the refinement of the discussion and the addition of supporting experiments. In particular, the novelty of the work is now more clearly articulated: Fe3+ promotes anodic •OH generation by modulating the interfacial water hydrogen-bond network. Overall, the manuscript is now much stronger than the previous version. However, a few minor issues should still be addressed before publication.

Response: We sincerely appreciate the highly positive feedback regarding the novelty and scientific merits of our manuscript. We also thank you for these valuable suggestions, which would be very beneficial to improving the manuscript. We accepted all the suggestions and have revised the manuscript accordingly.

1. In the revised manuscript, Figure 2 experiment condition "[TBA] = [MeOH] = 200 mM" should be revised according to the new data. It is indicated in the response letter that "the data in Figures 2d and Figures 2g of the manuscript (using 500 mM MeOH as the quenching agent)".

Response: Thank you for your reminder. We have changed "[TBA] = [MeOH] = 200 mM" in the manuscript to "[TBA] = [MeOH] = 500 mM". We have revised the following in the revised manuscript.

Figure 2: "Experimental conditions: current density = 7.5 mA cm^{-2}, [Na_2SO_4] = 50 mM, [MIT] = 20 µM, [DMPO] = 20 µM, [TBA] = [MeOH] = 500 mM, [DMSO] = 50 mM, [Fe^{3+}] = 0.2 mM."

2. With the additional experiment in Figure 3a, it is now clearer that •OH makes a greater contribution in the MIT oxidation system because MIT has a much higher reaction rate constant with •OH. To better distinguish the results shown in Figure 3 and Figure S9, the authors are suggested to indicate explicitly that the experiment in Figure 3 was conducted in the presence of MIT.

Response: Many thanks for your valuable suggestions. Figure 3a showed the summary of PMSO attenuation, $PMSO_2$ generation, and PMSO/$PMSO_2$ conversion

rate under the condition of 0.2 mM Fe^{3+} addition. While Figure S8 (after revised) compared the PMSO attenuation and $PMSO_2$ generation under different Fe^{3+} addition amounts. No MIT was added during the experiments shown in Figure 3a and Figure S8 (after revised). The presentation form of Figure 3a can more clearly show the difference in the contribution of •OH and Fe(H).

3. The descriptions on page 16, line 306, such as “appear as dispersed bulk water” and “strong electron-withdrawing effect”, seem to require further justification or more precise wording.

Response: We sincerely thank you for the rigorous scrutiny. We have therefore rephrased “appear as dispersed bulk water” and “strong electron-withdrawing effect” to “appear as weakly H-bond water” and “strong local polarization induced by Fe^{3+}”. We have revised the following in the revised manuscript.

Page 14, line 287: “The strong local H-bond network formed by the Fe^{3+} hydrated sphere ($[Fe(OH)(H_2O)_5]^{2+}$) was masked by paramagnetic effects, causing the overall NMR signal to appear as weakly H-bond water shifted toward upfield. As concentration increases (0.4–2 mM), more Fe^{3+} hydrated spheres formed, and the strong local polarization induced by Fe^{3+} within these spheres propagates through chemical bonds to the surface coordinated water molecules, weakening their O-H bonds.”

4. In the revised manuscript, page 7, line 113, “the Fe3+ are oxidized to high-valent iron” should be corrected to “the Fe3+ is oxidized to high-valent iron”.

Response: We extend our heartfelt gratitude to you for the very considerate comment. We have changed “the Fe^{3+} are oxidized to high-valent iron” to “the Fe^{3+} is oxidized to high-valent iron” in the revised manuscript. The revised content is as follows.

Page 7, line 107: “At the same time, the Fe^{3+} is oxidized to high-valent iron (Fe(H), including Fe(IV), Fe(V), and Fe(VI)), thereby expanding the oxidation range of the system.”

5. In the revised manuscript page 8, line 116-120: “In situ electrochemical spectroscopy and ab initio molecular dynamics (AIMD) demonstrates that

[Fe(OH)(H2O)5]2+ enhances the 4-HB-H2O and H-bond population at the electrode surface (Figure S1)."

The authors may want to reconsider referring to Figure S1 in the Introduction section, as it feels slightly unusual to cite a supplementary figure at this stage. And "demonstrates" should be corrected to "demonstrate".

Response: We appreciate your constructive suggestion. We have deleted "Figure S1" from the introduction in the revised manuscript. In addition, we have corrected "demonstrates" to "demonstrate" in the revised manuscript. We have revised the following in the revised manuscript.

Page 6, line 111: "*In situ* electrochemical spectroscopy and ab initio molecular dynamics (AIMD) demonstrate that $[Fe(OH)(H_2O)_5]^{2+}$ enhances the 4-HB-H_2O and H-bond population at the electrode surface[32], thereby guiding the reaction path towards a lower energy barrier[24, 33, 34]."

6. Page 10, line 175: please check the double space in "MeOH and TBA".

Response: Many thanks for your kind reminder. We have removed the unnecessary spaces in "MeOH and TBA". We have revised the following in the revised manuscript.

Page 9, line 172: "DMSO, a representative quencher for both •OH and Fe(H)[36], exhibited a slightly stronger inhibitory effect on pollutant degradation than MeOH and TBA in the Fe^{3+}/BDD system, indicating the involvement of Fe(H)."

7. It is also suggested that the authors check the consistency of tense when presenting the results, as there appears to be a mixture of past and present tense throughout the manuscript.

Response: We thank you for this careful observation. We have carefully reviewed and standardized the tense throughout the revised manuscript. Specific revision examples in the revised manuscript are as follows.

Page 7, line 134: "The 0.2 mM Fe^{3+} was selected over 1 mM to enhance degradation kinetics while reducing chemical use and iron sludge—avoiding reduced energy efficiency and secondary pollution at higher concentrations."

Page 8, line 159: "This 31% difference was attributed to the enhanced generation of •OH resulting from the introduction of Fe^{3+}. Comparisons with a sodium perchlorate electrolyte ruled out the contribution of sulfate radicals ($SO_4^{\bullet-}$) to pollutant removal (Supplementary Fig. 5)."

Page 9, line 176: "*In situ* electrochemical Raman spectroscopy showed that the characteristic signals of Fe(VI) increased with anodic potential, confirming Fe(VI) as the electrochemically generated species during the reaction (Supplementary Fig. 10)."

Page 9, line 186: "Quenching analysis and kinetic calculations (Fig. 2g, Supplementary Fig. 12, and Supplementary Text 6) indicated that •OH and Fe(IV)/Fe(V) together dominated pollutant degradation, contributing 88.9% and 8.4%, respectively, whereas direct electron transfer played a negligible role (2.7%)."

Page 10, line 205: "Finite element simulation were conducted to model the interfacial concentration distribution of •OH and Fe(H) at the micrometer scale[12]. COMSOL simulation visualized the mass transfer of active species at the anode interface (Fig. 2h and Supplementary Text 10)[40]."

Page 11, line 227: "As the Fe^{3+} concentration increased from 0 to 1 mM, the high-frequency characteristic peak shifted from lg 3.3 to lg 3.6 (Fig. 3c and Supplementary Fig. 15), indicating a shorter relaxation time and accelerated interfacial charge-transfer kinetics."

Page 18, line 383: "In the BDD system without Fe^{3+}, water molecules at the surface of the BDD electrode surface exhibited preferential orientation, but the H-bond network remained relatively loose and dynamic disorder."

Page 20, line 428: "The $[Fe(OH)(H_2O)_5]^{2+}$ regulated the interfacial reaction at the OHP site of the BDD anode through hydrogen bonding and long-range electrostatic interactions."
